# New Diarylamine K_V_10.1 Inhibitors and Their Anticancer Potential

**DOI:** 10.3390/pharmaceutics14091963

**Published:** 2022-09-17

**Authors:** Špela Gubič, Žan Toplak, Xiaoyi Shi, Jaka Dernovšek, Louise Antonia Hendrickx, Ernesto Lopes Pinheiro-Junior, Steve Peigneur, Jan Tytgat, Luis A. Pardo, Lucija Peterlin Mašič, Tihomir Tomašič

**Affiliations:** 1 Faculty of Pharmacy, University of Ljubljana, Aškerčeva cesta 7, 1000 Ljubljana, Slovenia; 2 AG Oncophysiology, Max-Planck Institute for Experimental Medicine, Hermann-Rein-Str. 3, 37075 Göttingen, Germany; 3 Toxicology and Pharmacology, Campus Gasthuisberg, University of Leuven, Onderwijs en Navorsing 2, Herestraat 49, 3000 Leuven, Belgium

**Keywords:** K_V_10.1, ion channels, hERG, SAR, antiproliferative activity

## Abstract

Expression of the voltage-gated potassium channel K_V_10.1 (Eag1) has been detected in over 70% of human cancers, making the channel a promising new target for new anticancer drug discovery. A new structural class of K_V_10.1 inhibitors was prepared by structural optimisation and exploration of the structure–activity relationship of the previously published hit compound ZVS-08 (**1**) and its optimised analogue **2**. The potency and selectivity of the new inhibitors between K_V_10.1 and hERG were investigated using whole-cell patch-clamp experiments. We obtained two new optimised K_V_10.1 inhibitors, **17a** and **18b**, with improved nanomolar IC_50_ values of 568 nM and 214 nM, respectively. Compound **17a** exhibited better ratio between IC_50_ values for hEAG1 and hERG than previously published diarylamine inhibitors. Compounds **17a** and **18b** moderately inhibited the growth of the K_V_10.1-expressing cell line MCF-7 in two independent assays. In addition, **17a** and **18b** also inhibited the growth of hERG-expressing Panc-1 cells with higher potency compared with MCF-7 cells. The main obstacle for newly developed diarylamine K_V_10.1 inhibitors remains the selectivity toward the hERG channel, which needs to be addressed with targeted drug design strategies in the future.

## 1. Introduction

The voltage-gated potassium channel K_V_10.1 (Eag1) is one of the eight members of the ether-à-go-go family, which is itself subdivided into three subfamilies (Eag, Erg, and Elk) [1,2]. Analogous to other K_V_ channels, Eag1 is formed by four subunits, with each monomer consisting of six transmembrane segments (S1–S6). The voltage-sensor domain (VSD), which consists of segments S1–S4, detects changes in membrane potential and triggers conformational changes, leading to pore opening. Segments S5 and S6 (pore domain, PD) of each monomer join together to form the ion-conducting pore, which includes the water-filled cavity and the selectivity filter (SF). The second part represents the narrowest part of the pore, which is lined with oxygen atoms that coordinate the movement of potassium ions during permeation [3].

The channel K_V_10.1 has emerged as a very attractive oncological target in the last two decades. The channel is mainly expressed in healthy tissues of the central nervous system, while it is barely detectable in peripheral tissues, with the exception of testis and adrenal gland [4,5]. Aberrant K_V_10.1 expression has been detected in over 70% of human cancers, regardless of tumour type, which is why the channel is considered a nearly universal tumour marker [6]. K_V_10.1 activity is involved in important processes for cancer cell growth and spread, such as cell cycle control, proliferation, migration, angiogenesis, and resistance to hypoxia [7]. The mechanisms of the channel’s involvement in cancer proliferation appear to be related to alterations in membrane potential control and non-canonical effects of overexpressed K_V_10.1, affecting Ca^2+^ signalling and microtubule dynamics [8]. Previous in vitro and in vivo studies have shown that specific inhibition of K_V_10.1 can successfully reduce tumour progression [9,10,11,12,13]. On this basis, the channel is a promising target for new anticancer drug discovery. Novel selective K_V_10.1 inhibitors could, therefore, prolong the survival of patients with fibrosarcoma [14], ovarian cancer [15], glioblastoma [16], acute lymphoid leukaemia [17], gastric [18], head and neck [19], and colon cancer [20], in which the expression of the channel is significantly higher than in normal tissues. Moreover, in a retrospective study, K_V_10.1 inhibitors have already shown an effective improvement in survival in a group of patients with brain metastases compared to an untreated control group [16]. To date, no specific and selective small-molecule K_V_10.1 inhibitors have been reported because they all exhibited significant inhibition of the cardiac hERG channel, which raised major concerns about adverse cardiac side effects [21]. Recent studies have pointed to a particular structural difference between K_V_10.1 and hERG channels that has profound functional implications and suggests the possibility of selective inhibition of K_V_10.1 in cancer therapies [22].

**Figure 1:**
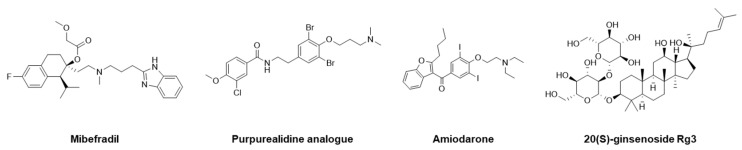
Structures of known ligands binding to the voltage-sensor domain of K_V_10.1.

To date, only a very small number of compounds has been described to act on K_V_10.1 [21]. Most small molecules were identified by studying K_V_10.1 inhibition of the previously described antitumour agents and hERG inhibitors, as these channels share structural similarities. K_V_10.1 inhibitors can interact with the channel at multiple binding sites, including VSD and PD. Since K_V_10.1 and hERG share a high structural similarity in the central cavities, designing a selective small molecule that blocks the pore is a major challenge [23]. In contrast, a more promising approach is to target the VSD domain. Small molecules, such as mibefradil, purpurealidin I analogues, amiodarone, and 20(*S*)-ginsenoside Rg3 (Figure 1), bind to VSD and act as gating modifiers [24,25]. The exact binding sites of these inhibitors are not yet known, but new cryo-electron microscopy structures of K_V_10.1 and hERG may contribute to the development of new specific inhibitors that bind to VSD [3,23].

**Figure 2:**
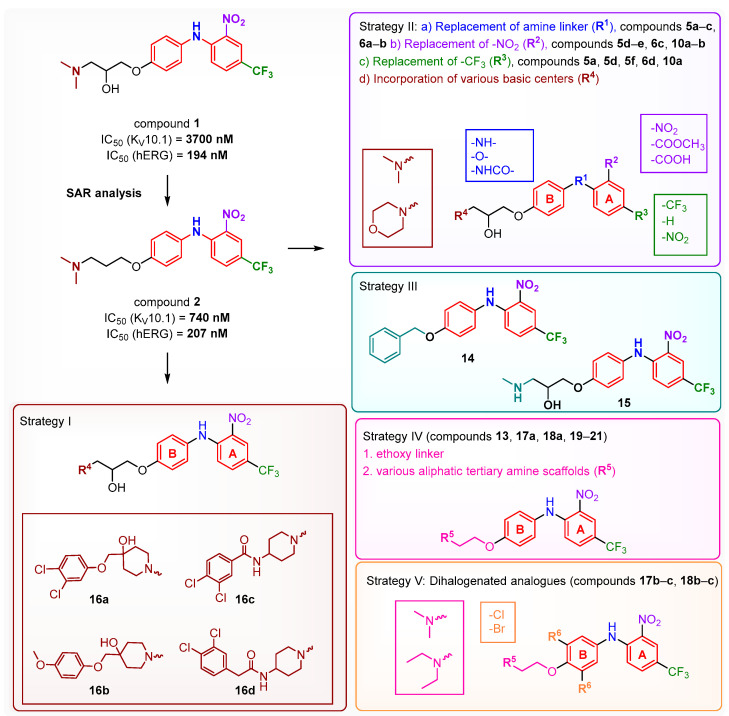
Design of the hit compound ZVS-08 (**1**) analogues (Strategies I–V).

Our research group recently published a novel structural class of K_V_10.1 inhibitors discovered by a unique ligand-based drug design strategy [26]. Compound ZVS-08 (**1**, Figure 2) with a diarylamine structure was identified as a hit compound in a computational 3D pharmacophore model. In biological assays, it inhibited the K_V_10.1 channel with the effect of the gating modifier (IC_50_ = 3.70 µM). The even more potent K_V_10.1 inhibitor **2** (IC_50_ = 740 nM, Figure 2) was obtained by structure–activity relationship studies. Compound **2** was appropriately selective for some K_V_ and Na_V_ channels, but also showed significant hERG inhibition (IC_50_ = 207 nM). Growth of the breast cancer cell line MCF-7, which has high K_V_10.1 levels and low hERG levels, was more strongly inhibited by compound **2** than the growth of Panc-1 cells, which have low K_V_10.1 levels and high hERG levels. Compound **2** also induced significant apoptosis in tumour spheroids of Colo357 cells. Here, we present a new series of potent nanomolar K_V_10.1 inhibitors obtained by structural optimisation of previously published compounds **1** and **2** We also demonstrated the potential of this new structural class of Kv10.1 inhibitors for antiproliferative activity in MCF-7 cancer cell models.

## 2. Materials and Methods

### 2.1. Synthetic Procedures and Analytical Data

Reagents and solvents for the syntheses were from Fluorochem Ltd. (Derbyshire, UK), Apollo Scientific Ltd. (Stockport, UK), Sigma-Aldrich (St. Louis, MO, USA), Enamine Ltd. (Kiev, Ukraine), and TCI (Tokyo, Japan), and were not further purified. Analytical thin-layer chromatography was performed on silica gel aluminium plates (0.20 mm; 60 F254; Merck, Darmstadt, Germany). Silica gel 60 (particle size 230–400 mesh) was used for column chromatography. Reversed-phase column chromatography (RP-CC) was performed on the Isolera Biotage One Flash Chromatography system (SNAP Biotage KP-C18-HS column, 12 g, Biotage, Uppsala, Sweden) using a gradient of 0.1% TFA in deionised water and MeCN as eluent (gradient 10–100% MeCN in 15 column volumes (300 mL); 100% MeCN for 5 column volumes (100 mL)). ^1^H and ^13^C NMR spectra were recorded using a 400 MHz NMR spectrometer (Bruker Advance 3, Bruker, Billerica, MA, USA). The splitting patterns were designated as follows: s, singlet; d, doublet; dd, double doublet; td, triple doublet; t, triplet; dt, double triplet; ddd, double of doublet of doublet; ddt, doublet of doublet of triplets; q, quartet; and m, multiplet. The purity of the prepared compounds was verified by liquid chromatography-mass spectrometry according to method A (see below) on a Thermo Scientific Dionex UltiMate 3000 modular system (Thermo Fisher Scientific Inc., Waltham, MA, USA). HRMS measurements were performed on an LC–MS/MS system (Exactive Plus Orbitrap mass spectrometer; Thermo Scientific Inc., Waltham, MA, USA). The high-resolution mass spectra (HRMS) used optical rotation detection at λ = 589 nm (Polarimeter model 241; Perkin Elmer, Boston, MA, USA).

Analytical reversed-phase UPLC analyses were performed using a modular system (Thermo Scientific Dionex UltiMate 3000 modular system; Thermo Fisher Scientific Inc., MA, USA). Method: Waters Acquity UPLC^®^ HSS C18 SB column (2.1 × 50 mm, 1.8 μm), T = 40 °C; injection volume = 5 µL; flow rate = 0.4 mL/min; detector λ = 254 nm; mobile phase A (0.1% TFA [*v*/*v*] in water), mobile phase B (MeCN). Gradient: 0–2 min, 10% B; 2–10 min, 10–90% B; 10–12 min, 90% B. The purity of the tested compounds was determined to be ≥95% by UPLC at 254 nm.

Detailed chemical synthesis procedures and chemical analysis results of all intermediates and final compounds **3a**–**b**, **4a**–**g**, **5a**–**f**, **6a**–**d**, **7**, **8a**–**b**, **9a**–**b**, **10a**–**b**, **11**, **12a**–**d**, **13**, **14**, **15**, **16a**–**d**, **17a**–**c**, **18a**–**c**, **19**, **20**, and **21** are described in Appendix A and Supplementary Materials.

### 2.2. Electrophysiological Recordings on HEK-293 Cells

For whole-cell patch-clamp measurements, stably transfected HEK-293 cells expressing K_V_10.1 [26,27,28] were grown in DMEM with 10% FCS and 300 µg/mL Zeocin on glass coverslips coated with fibronectin. All experiments were conducted at room temperature under perfusion by gravity with extracellular solution (160 mM NaCl, 2.5 mM KCl, 2 mM CaCl_2_, 1 mM MgCl_2_, 10 mM Hepes pH 7.4 with NaOH, 8 mM glucose). Pipettes were made from #1 glass capillaries (WPI) and had 1–3 MΩ resistance. The intracellular solution contained 100 mM KCl, 45 mM *N*-methyl-D-glucamine, 10 mM 1,2-bis(*o*-aminophenoxy)ethane-*N*,*N*,*N*′,*N*′-tetraacetic acid (BAPTA), 10 mM Hepes pH 7.35. Stimulation was applied and data were acquired with an EPC10 Plus amplifier (HEKA Elektronik) controlled with Patch Master software. Compounds were applied with an 8-channel fast solution exchanger (ALA). Compounds were prepared to a stock concentration of 10 mM in DMSO. The control solution contained 0.5% DMSO. Stimuli consisted of 500 ms depolarizations to +40 mV.

### 2.3. Electrophysiological Recordings on Xenopus laevis Oocytes

Stage V–VI oocytes were isolated by partial ovariectomy from *Xenopus laevis* frogs (African clawed frog) [26]. Mature female frogs were purchased from CRB Xénopes (Rennes, France) and were housed in the Aquatic Facility (KU Leuven) in compliance with the regulations of the European Union (EU) concerning the welfare of laboratory animals as declared in Directive 2010/63/EU. The use of *Xenopus laevis* was approved by the Animal Ethics Committee of the KU Leuven (Project nr. P186/2019). After anesthetising the frogs by a 15 min submersion in 0.1% tricaine methanesulphonate (pH 7.0), the oocytes were collected. The isolated oocytes were then washed with a 1.5 mg/mL collagenase solution for 2 h to remove the follicle layer.

Ion channels (K_V_10.1 and hERG) were expressed in *Xenopus laevis* oocytes, by linearisation of the plasmids and subsequent in vitro transcription using a commercial T7 or SP6 mMESSAGE mMACHINE transcription kit (Ambion, Carlsbad, CA, USA). Defolliculated *Xenopus* oocytes were then injected with 20–50 nL of the cRNA at a concentration of 1 ng/nL using a micro-injector (Drummond Scientific1, Broomall, PA, USA). The oocytes were incubated in a solution containing (in mM): NaCl, 96; KCl, 2; CaCl_2_, 1.8; MgCl_2_, 2 and HEPES, 5 (pH 7.4), supplemented with 50 mg/L gentamycin sulphate and 90 mg theophylline. After ex vivo translation, the ion channels are correctly inserted in the cell membrane of the oocytes.

Two-electrode voltage-clamp recordings were performed at room temperature (18–22 °C) using a Geneclamp 500 amplifier (Molecular Devices, San Jose, CA, USA) controlled by a pClamp data acquisition system (Axon Instruments1, Union City, CA, USA) and using an integrated digital TEVC amplifier controlled by HiClamp, an automated Voltage-Clamp Screening System (Multi Channel Systems MCS GmbH, Reutlingen, Germany). Whole-cell currents from oocytes were recorded 1–7 days after injection. The bath solution composition was ND96 (in mM): NaCl, 96; KCl, 2; CaCl_2_, 1.8; MgCl_2_, 2 and HEPES, 5 (pH 7.5). Voltage and current electrodes were filled with a 3 M solution of KCl in H_2_O. Resistances of both electrodes were kept between 0.5 and 1.5 MΩ. The elicited Kv10.1 and hERG currents were filtered at 0.5 kHz and sampled at 2 kHz using a four-pole low-pass Bessel filter. Leak subtraction was performed using a p/4 protocol. Starting from a holding potential of −90 mV, currents for Kv10.1 were evoked by 1 s depolarizing pulses to 0 mV and currents for hERG were evoked by 2.5 s depolarizing prepulses to +40 mV, followed by hyperpolarizing pulses to −120 mV for 2.5 s. The concentration dependency of all compounds was assessed by measuring the current inhibition in the presence of increasing compound concentrations. To this end, a stock solution of the compounds was prepared in 100% DMSO for the sake of solubility. From this stock solution, adequate dilutions with a maximum of 0.5% DMSO were made for testing. The data of the concentration–response curves were fitted with the Hill equation: y = 100/{1 + (IC_50_/[compound])^h^}, where y is the amplitude of the compound- induced effect, IC_50_ is the compound concentration at half-maximal efficacy, (compound) is the compound concentration, and h is the Hill coefficient.

### 2.4. Statistical Analysis

All electrophysiological data are presented as means ± SEM of n ≥ 3 independent experiments unless otherwise indicated. Oocyte data were analysed using pClamp Clampfit 10.4 (Molecular Devices, Downingtown, PA, USA) and OriginPro 9 (Originlab, Northampton, MA, USA) or GraphPad Prism 8 software (GraphPad Software, Inc., San Diego, CA, USA). Patch-clamp data analysis was performed using FitMaster (HEKA Electronics), IgorPro (Wavemetrics, Inc., Lake Oswego, OR, USA) and GraphPad Prism 9 (GraphPad Software, Inc., San Diego, CA, USA). The Dunnett test and one-way ANOVA were performed to calculate the significance of the induced inhibition compared to the control (*p* < 0.05).

### 2.5. Cell Culture

MCF-7 (DSMZ ACC 115) and PANC-1 (DSMZ ACC 783) cells were grown as recommended by the cell supplier at 5% CO_2_ and 37 °C in a humidified atmosphere. For determining cell growth, cells were seeded in 96-well culture plates (Corning, Corning, NY, USA) at a density of 10^4^ cells/well. After 24 h, the compounds were added diluted in medium. Six wells per condition were used. Four images of each well were collected at 1 h intervals for 48–72 h. Two biological replicates were performed. Images were acquired using an Incucyte device (Sartorius/Essen biosciences). Cell confluence was calculated using the Incucyte software on the phase contrast images. The results are expressed as percent confluence per well normalised to the value with no compounds added.

### 2.6. MTS Assay

The antiproliferative activities of the compounds were evaluated on the breast cancer cell line MCF-7 (ATCC HTB-22, adherent cells isolated from a 69-year-old white woman), using an MTS (Promega, Madison, WI, USA) assay according to the manufacturer’s instructions [29]. The MCF-7 cell line was obtained from the American Type Culture Collection (ATCC, Manassas, VA, USA). Cells were cultured in low-glucose Dulbecco’s Modified Eagle’s Medium (DMEM) (Sigma-Aldrich, St. Louis, MO, USA), supplemented with 10% fetal bovine serum (Gibco, Thermo Fisher Scientific, Waltham, MA, USA), 100 U/mL penicillin (Sigma-Aldrich, St. Louis, MO, USA), 100 µg/mL streptomycin (Sigma-Aldrich, St. Louis, MO, USA), and 2 mM l-glutamine (Sigma-Aldrich, St. Louis, MO, USA). The cell line was incubated in a 5% CO_2_ atmosphere at 37 °C. Cells were plated out in 96-well plates at a density of 2000 cells per well and incubated overnight. The next day, cells were treated with the indicated compounds, positive control (17-DMAG), or vehicle control (0.5% DMSO) and incubated for 72 h. Then CellTiter96 Aqueous One Solution Reagent (10 µL; Promega, Madison, WI, USA) was added to each well and cells were incubated for an additional 3 h. Absorbance was measured at 492 nm using a microplate reader (Synergy 4 Hybrid; BioTek, Winooski, VT, USA). Independent experiments were repeated twice, each time in triplicate. Statistical significance (*p* < 0.05) was calculated using a two-tailed Welch’s *t*-test between treated groups and DMSO. IC_50_ values are given as averages of the independent measurements and were calculated using GraphPad Prism 8.0 software (San Diego, CA, USA). IC_50_ values represent the concentration at which a compound elicits a half-maximal response.

## 3. Results

### 3.1. Design

New K_V_10.1 inhibitors were designed and synthesised to increase the potency of published compounds **1** (ZVS-08) and **2** against K_V_10.1 and selectivity against hERG, and to investigate structure–activity relationships (SAR) important for K_V_10.1 inhibition. Compound **1** (Figure 2) with diarylamine structure and 1-(dimethylamino)-propan-2-ol segment attached to aromatic moiety B was modified in the different parts (R^1^-R^6^) of the molecule to obtain a focused library of compounds screened for K_V_10.1 inhibition (Figure 2, strategies I-V). In strategy I, the basic dimethylamino centre of ZVS-08 (**1**) was extended to different cyclic amines bearing aromatic rings (R^4^, Figure 2) to obtain new analogues with three aromatic scaffolds. The aim of strategy II (Figure 2) was to investigate the importance of the amine linker (R^1^) and the electron-withdrawing nitro (R^2^) and trifluoromethyl (R^3^) groups of the hit compound ZVS-08 (**1**) and compound **2** for K_V_10.1 inhibition. As a result, several new analogues with modifications at four different positions (R^1^-R^4^) were developed. The new analogues in strategy II contained the cationic dimethylamino centre of hit compound **1** or was replaced by a morpholino group (R^4^) to maintain the basic character of the compound but further increase its polarity. Using strategy III (Figure 2), we tested whether the basic dimethylamino centre was required for the inhibition of K_V_10.1. Therefore, two new analogues with modifications of this segment were developed. Since the hydroxyl group of ZVS-08 (**1**) was found not to be an important structural component for the inhibition of K_V_10.1 (based on the comparison between **1** and **2**, Figure 2), the new analogues of strategy IV contained different aliphatic tertiary amine basic centres connected to the aromatic part B via a shorter ethoxy linker. The new dihalogenated analogues (strategy V, Figure 2) contained additional chloride or bromide atoms in aromatic part B and dimethylamino- or diethylamino-ethoxy groups.

### 3.2. Synthesis

**Scheme 1:**
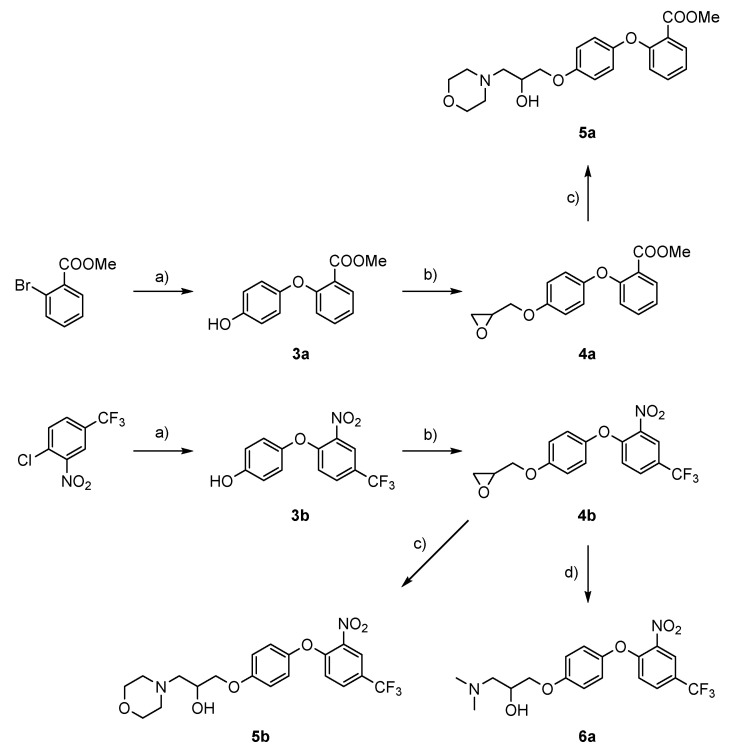
Reagents, solvents, and conditions: (**a**) hydroquinone, K_2_CO_3_, CuO, pyridine, 110 °C, 72 h; (**b**) (±)-epichlorohydrin, MeCN, K_2_CO_3_, 80 °C, 16 h; (**c**) morpholine, acetonitrile, r.t., 72 h; (**d**) Me_2_NH × (**e**) HCl, K_2_CO_3_, MeCN, 70 °C, 24 h.

**Scheme 2:**
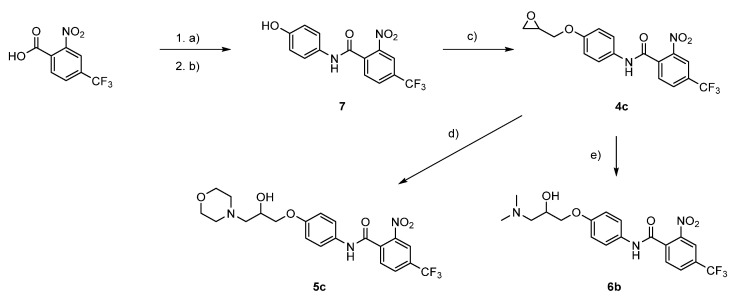
Reagents, solvents, and conditions: (**a**) Oxalyl chloride, 2 drops of DMF, dichloromethane, 40 °C, 24 h; (**b**) 4-aminophenol, triethylamine (TEA), MeCN, reflux, 24 h; (**c**) (±)-epichlorohydrin, MeCN, K_2_CO_3_, 80 °C, 16 h; (**d**) morpholine, MeCN, r.t., 72 h; Me_2_NH × HCl, MeCN, K_2_CO_3_, 70 °C, 24 h.

**Scheme 3:**
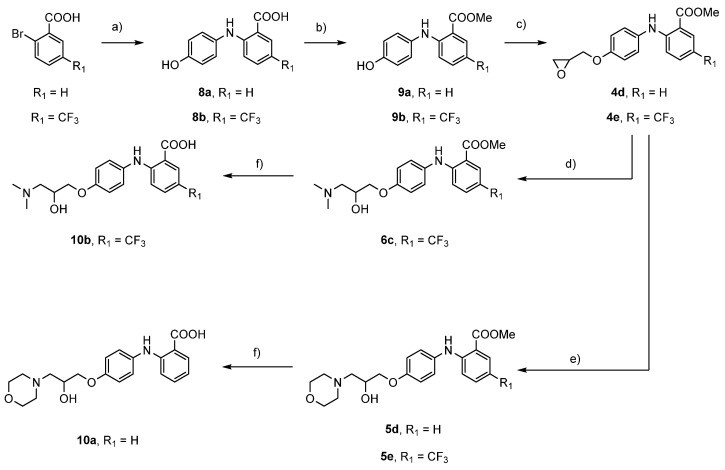
Reagents, solvents, and conditions: (**a**) 4-aminophenol, K_2_CO_3_, CuO, Cu, 2-ethoxyethanol, 130 °C, 16 h; (**b**) thionyl chloride, anhydrous MeOH, 70 °C, overnight; (**c**) (±)-epichlorohydrin, MeCN, K_2_CO_3_, 80 °C, 16 h; (**d**) Me_2_NH × HCl, acetonitrile, K_2_CO_3_, 70 °C, 24 h; (**e**) morpholine, dichloromethane, r.t., 72 h; (**f**) NaOH, EtOH, water, r.t., 72 h.

**Scheme 4:**
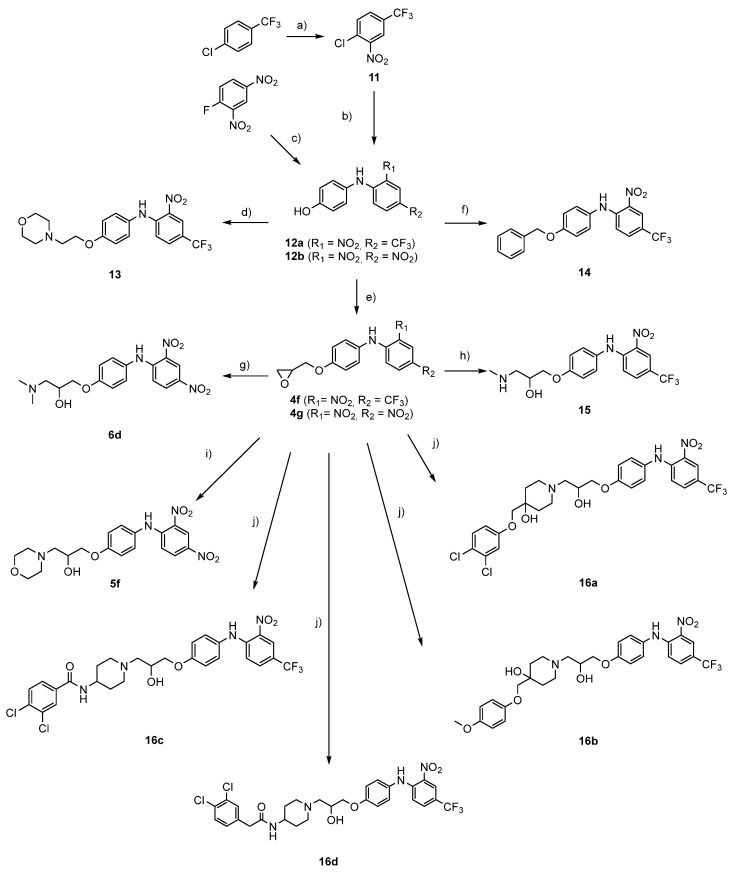
Reagents, solvents, and conditions: (**a**) H_2_SO_4_, HNO_3_, 80 °C, 1 week; (**b**) 4-aminophenol, NaHCO_3_, MeCN, 90 °C, 24 h; (**c**) 4-aminophenol, NaHCO_3_, MeCN, 90 °C, 24 h; (**d**) 4-(2-chloroethyl)morpholine, KOH, EtOH, 100 °C, overnight; (**e**) (±)-epichlorohydrin, MeCN, K_2_CO_3_, 80 °C, 16 h; (**f**) 4-benzyloxy aniline, NaHCO_3_, toluene, 100 °C, 24 h; (**g**) Me_2_NH × HCl, MeCN, 70 °C, 24 h; (**h**) MeNH_2_, K_2_CO_3_, MeCN, 70 °C, 24 h; (**i**) morpholine, dichloromethane, r.t., 72 h; (**j**) appropriate amine, K_2_CO_3_, MeCN, 70 °C, 24 h.

**Scheme 5:**
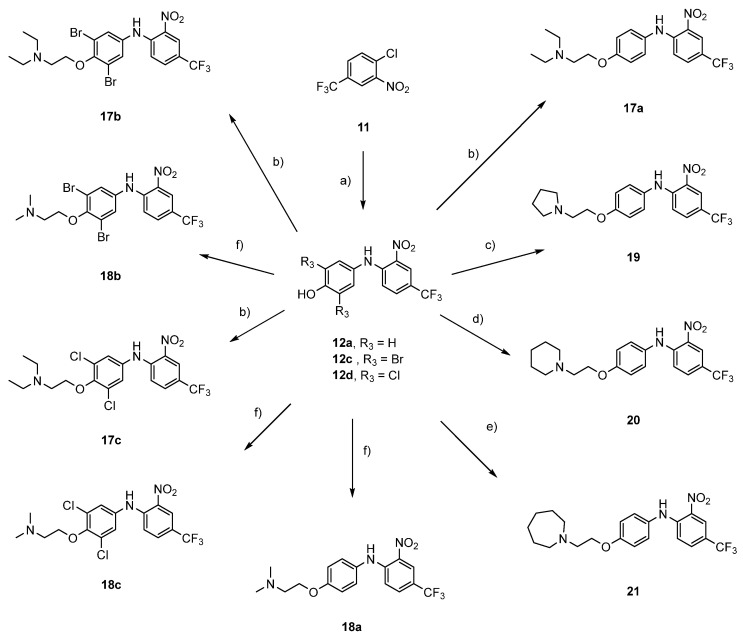
Reagents, solvents, and conditions: (**a**) 4-aminophenol or 4-amino-2,6-dibromophenol or 4-amino-2,6-dichlorophenol, NaHCO_3_, MeCN, 90 °C, 24 h; (**b**) 2-chloro-*N*,*N*-diethylethan-1-amine × HCl, KOH or K_2_CO_3_, EtOH or MeCN, 100 °C or 90 °C, overnight; (**c**) 1-(2-chloroethyl)pyrrolidine × HCl, K_2_CO_3_, MeCN, 90 °C, 24 h; (**d**) 1-(2-chloroethyl)piperidine × HCl, K_2_CO_3_, MeCN, 90 °C, 24 h; (**e**) 1-(2-chloroethyl)azepane hydrochloride × HCl, K_2_CO_3_, MeCN, 90 °C, 24 h; (**f**) 2-chloro-*N*,*N*-dimethylethan-1-amine × HCl, MeCN, KOH or K_2_CO_3_, 100 °C or 90 °C, 16 h.

The syntheses of the new analogues are shown in Scheme 1, Scheme 2, Scheme 3, Scheme 4 and Scheme 5. The differently substituted diarylphenols **3a**–**b**, **7**, **8a**–**b**, and **12a**–**d** were prepared through four different procedures. The synthesis of 4-hydroxyphenoxy analogues **3a**–**b** was carried out with the addition of CuO as a catalyst and a base at high temperature (Scheme 1) [30]. For the preparation of benzamide **7**, 2-nitro-4-(trifluoromethyl)benzoic acid was first converted with oxalyl chloride to the corresponding benzoyl chloride and the product was then refluxed in the presence of base and *p*-aminophenol to give benzamide **7** (Scheme 2). For the synthesis of compounds **8a**–**b** without the electron-withdrawing nitro group, CuO and Cu were used as catalysts (Scheme 3) [30]. The carboxylic acids **8a**–**b** were converted to the methyl esters **9a**–**b** by addition of thionyl chloride in anhydrous methanol (Scheme 3). *o*-Nitro-*p*-trifluoromethyl-substituted diarylphenols **12a** and **12c**–**d** were synthesised in two separate steps (Scheme 4 and Scheme 5). First, the nitro group was introduced by nitration of 1-chloro-4-(trifluoromethyl)benzene with concentrated nitric and sulfuric acids to give **11**, followed by the addition of the nonhalogenated or dihalogenated *p*-aminophenols at high temperature in the presence of a base to give **12a** and **12c**–**d** [31]. The dinitro-substituted compound **12b** was prepared by coupling *p*-aminophenol and 1-fluoro-2,4-dinitrobenzene with the addition of a base at reflux (Scheme 4). Epoxides **4a**–**g** were prepared using (±)-epichlorohydrin and K_2_CO_3_ as base (Scheme 1, Scheme 2, Scheme 3 and Scheme 4). For the epoxide opening, morpholine was used to give a racemic mixture of compounds **5a**–**f**, dimethylamine to give a racemic mixture of compounds **6a**–**d**, methylamine to give a racemic mixture of compound **15**, and the corresponding amines with aromatic moieties to give a racemic mixture of analogues **16a**–**d**. The methyl esters **5d** and **6c** were hydrolysed with 1 M sodium hydroxide to form the corresponding benzoic acids **10a**–**b** (Scheme 3). Compounds **13**, **19**, **20**, and **21** were prepared from *o*-nitro-*p*-trifluoromethyl-substituted diarylamine phenol **12a** by addition of 4-(2-chloroethyl)morpholine for **13**, 1-(2-chloroethyl)pyrrolidine for **19**, 1-(2-chloroethyl)piperidine for **20**, or 1-(2-chloroethyl)azepane for **21** and base at reflux (Scheme 4 and Scheme 5). Compound **14** was synthesised from **12a** by addition of 4-benzyloxyaniline and base at high temperature (Scheme 4). Differently substituted diethylamino and dimethylamino analogues were formed at reflux from nonhalogenated or dihalogenated diarylamine phenols **12a** or **12c**–**d** by addition of base and 2-chloro-*N*,*N*-diethylethan-1-amine for **17a**–**c** or 2-chloro-*N*,*N*-dimethylethan-1-amine for **18a**–**c**.

### 3.3. Analogues of Compounds ***1*** and ***2*** and Structure–Activity Relationships

Five different types of new K_V_10.1 inhibitors were designed and synthesised to increase the potency on K_V_10.1. Based on the study of the structure–activity relationships (SAR) of the analogues of ZVS-08 (**1**), we identified the structural components in this structural type of K_V_10.1 inhibitor that are important for K_V_10.1 inhibition.

The new analogues **16a**–**d**, based on three aromatic scaffolds (Table 1, strategy I), were an unsuccessful attempt to increase the potency of previously published compounds **1** and **2**. Similarly, almost all compounds from strategy II (Table 2) were inactive at 10 μM unless an amine linker (R^1^), two nitro groups (R^2^ and R^3^), and morpholino (**5f**, R^4^) or dimethylamino (**6d**, R^4^) basic centres were present. Compound **14** from strategy III without a tertiary amine (R^4^) was inactive as a K_V_10.1 inhibitor (Table 3), but analogue **15** with a secondary aliphatic methylamine showed significant inhibition of the Kv10.1 current. Because the results for the new analogues of strategies II and III showed that the amine linker (R^1^), two electron-withdrawing groups (-NO_2_ as R^2^ and -CF_3_ as R^3^), and the aliphatic tertiary amine (R^4^) were very important structural elements for the inhibitory activity, these structural elements were included in the new compounds of strategies IV and V, but the alkoxy segments of **1** and **2** (strategies IV and V) and their second aromatic part (strategy V) were modified. The new compounds in strategy IV (Table 4), which included shorter ethoxy linkers and different aliphatic tertiary amines, such as diethylamine (**17a**), dimethylamine (**18a**), piperidin-1-yl (**20**) and azepan-1-yl (**21**) were the most potent K_V_10.1 inhibitors. Some of the new dihalogenated diarylamines in strategy V (Table 5) with basic diethylamine or dimethylamine centres caused a high-percentage inhibition of K_V_10.1 already at 1 μM.

It can be concluded that the most potent new diarylamine inhibitors contain a combination of a disubstituted phenyl ring with *para*-trifluoromethyl and *ortho*-nitro groups and a phenyl ring with two *meta*-halogen groups or an unsubstituted phenyl ring. By modifications of the aliphatic chain between the basic centre and the aromatic ring of ZVS-08 (**1**), we succeeded in increasing the potency and the most potent K_V_10.1 inhibitors contained a combination of a shorter ethyloxy linker and various aliphatic tertiary amines, of which diethylamine or methylamine were optimal.

**Figure 3:**
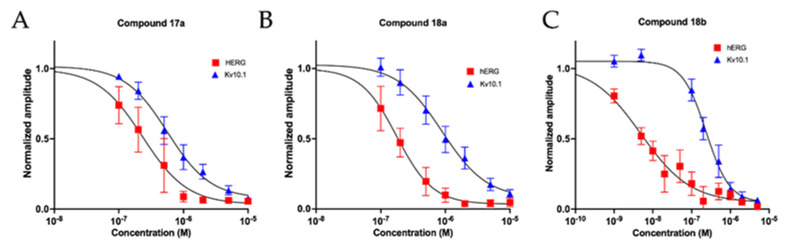
(**A**) Dose–response curves of compound **17a** for K_V_10.1 (blue) and hERG (red) inhibition. The IC_50_ values from the fit were 568 nM (95% CI 427 to 783 nM N = 4–7) for K_V_10.1 and 232 nM (CI: 186 to 296 nM N= 5–6) for hERG. (**B**) Dose–response curves of compound **18a** for K_V_10.1 (blue) and hERG (red) inhibition. The IC_50_ values from the fit were 892 nM (95% CI 640 nM to 1.40 µM, N = 5–6) for K_V_10.1 and 177 nM (CI: 163 to 191 nM, N = 2–3) for hERG. (**C**) Dose–response curves of compound **18b** for K_V_10.1 (blue) and hERG (red) inhibition. The IC_50_ values from the fit were 237 nM (95% CI 177 to 331 nM, N = 2–6) for K_V_10.1 and 4.9 nM (CI: 2.34 to 9.44 nM, N = 2–6) for hERG.

Diethylamine-containing compound **17a** and dimethylamine-containing compound **18a** from strategy IV and novel 3,5-dibromo-(dimethylamino)ethoxy) analogue **18b** from strategy V were further evaluated for K_V_10.1 and hERG inhibition (dose–response curves, Figure 3). Compound **17a** inhibited K_V_10.1 with an IC_50_ value of 568 nM and was more potent than the previously most active compound **2** (IC_50_ = 740 nM). Compound **17a** was also similarly potent against hERG (IC_50_ = 232 nM) than **2** (IC_50_ =207 nM), allowing us to slightly increase selectivity index against hERG with the new analogue. Compound **18b** was the most potent new K_V_10.1 inhibitor (IC_50_ = 216 nM), but also inhibited hERG very strongly with an IC_50_ value of 4.9 nM. Increasing the lipophilicity of the molecule by adding two *meta*-halogen groups to the unsubstituted phenyl ring, as in the case of **18b**, increased the potency for both channels but impaired the affinity for hERG considerably more. It appears that the diarylamine core with a disubstituted phenyl ring containing *ortho*-nitro and *para*-trifluoromethyl groups and an unsubstituted phenyl ring is essential for K_V_10.1 inhibition, but that with modifications of the alkoxy linker lengths and the basic dimethylamino centre, selectivity toward hERG can be increased.

The new analogues were also tested for their K_V_10.1 and hERG inhibition in another independent assay ex vivo in *Xenopus laevis* oocytes expressing K_V_10.1 or hERG (Tables S1–S5, Supplementary Materials). Some of the most active compounds (**17a**, **18a**, **18c**, **20**, and **21**) on HEK-293 cells also achieved the highest percentage of K_V_10.1 in *Xenopus laevis* oocytes. In addition, we determined their IC_50_ values for K_V_10.1 and hERG and the most potent compound was **17a**, which inhibited K_V_10.1 currents with an IC_50_ value of 3.6 µM and hERG currents with an IC_50_ value of 2.0 µM (Table S4).

### 3.4. Antiproliferative Activities of Novel K_V_10.1 Inhibitors ***17a*** and ***18b***

K_V_10.1 inhibitors are promising candidates that have antiproliferative activity against cancer cell lines expressing this channel. Our new, highly potent K_V_10.1 inhibitors **17a** and **18b** were tested for their antiproliferative activity against the MCF-7 breast cancer cell line, which has high K_V_10.1 and low hERG expression. Different concentrations of the compounds were added to the culture medium and the MTS assay was used to determine the number of viable cells after 72 h of incubation. The results obtained are shown in Table 6. Both compounds moderately inhibited the growth of MCF-7 cancer cells. Compound **18b**, which was the most potent K_V_10.1 inhibitor, inhibited the proliferation of MCF-7 cancer cells with an IC_50_ value of 11.9 μM. Compound **17a**, whose IC_50_ value for K_V_10.1 was approximately twice that of **18b**, inhibited proliferation of MCF-7 cancer cells with an IC_50_ value of 18.5 μM.

**Figure 4:**
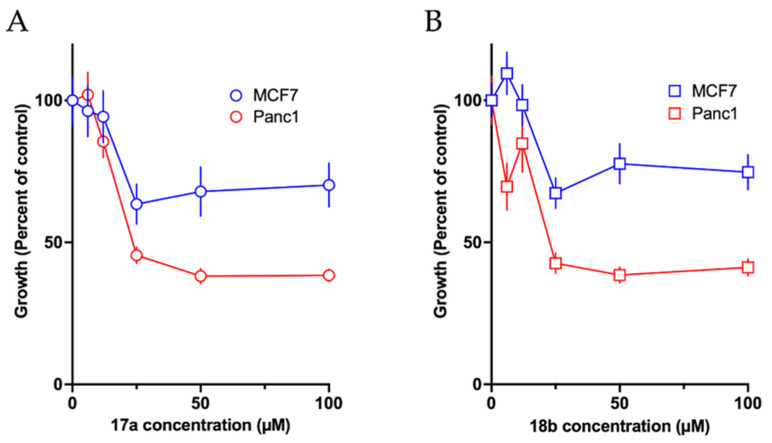
Effects of compounds **17a** and **18b** on the growth of Panc-1 and MCF-7 cells. (**A**) Compound **17a** inhibited the growth of Panc-1 (red) and MCF-7 cells (blue) by approximately 50% at concentrations higher than 25 µM. The inhibition of growth was stronger on Panc-1 cells. (**B**) Compound **18b** showed a similar behaviour on both cell lines. Compound **18b** inhibited the growth of Panc-1 cells more potently than MCF-7.

The effect of different concentrations of inhibitors **17a** and **18b** was tested on a cancer cell line with low hERG and high K_V_10.1 (MCF-7, breast) expression and another cancer cell line with high hERG and very low K_V_10.1 expression (Panc-1, from pancreas). Cell growth was then monitored over 48–72 h (Figure 4). Both compounds inhibited cancer cell growth to a very similar extent and also with similar potency, reducing proliferation of both cell lines by approximately half at 25 µM. Higher concentrations up to 100 µM only slightly increased growth inhibition. The MCF-7 cells were less sensitive to both compounds. The higher sensitivity of Panc-1 cells may be due to the high potency of both compounds in inhibiting hERG (Table 6), which is highly expressed in this cell line and is one of the major ion-channel-encoding genes involved in the initiation and maintenance of neoplastic growth [32,33]. In summary, **17a** and **18b** were able to reduce the growth of cancer cells expressing K_V_10.1 or hERG, suggesting that both channels contribute to cancer cell proliferation. Therefore, our new inhibitors have a potential anticancer effect.

## 4. Conclusions

Two new potent K_V_10.1 inhibitors were discovered by structural optimisation of previously published compounds based on the diarylamine scaffold. Important structural elements for the activity of these inhibitors were identified by SAR studies. We learned that the aliphatic chain between the basic centre and the diarylamine scaffold can be modified to increase potency at K_V_10.1 and improve selectivity toward hERG. The new inhibitor **17a**, with a shorter ethoxy linker and a basic diethylamine centre, showed improved potency (IC_50_ = 568 nM) and a better ratio between IC_50_ values for hEAG1 and hERG than previously published diarylamine inhibitors. The most potent analogue of the series, **18b**, inhibited K_V_10.1 with an IC_50_ value of 216 nM, but also caused significant hERG inhibition. Compounds **17a** and **18b** moderately inhibited the growth of MCF-7 breast cancer cells, which have low hERG and high K_V_10.1 expression. The new diarylamine K_V_10.1 inhibitors provided new structural information required for K_V_10.1 inhibition, but selectivity toward hERG remains a challenge.

## Figures and Tables

**Table 1:** Structures and potencies of compounds from Strategy I.

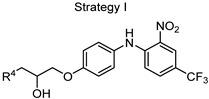		
Compound ID	R^4^	% of K_V_10.1 Inhibition at 50 μM (HEK-293 Cells)
**16a**	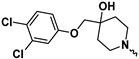	44.6 ± 8.6%
**16b**	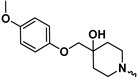	60.7 ± 9.6%
**16c**	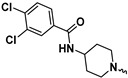	26.8 ± 10.1%
**16d**	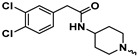	21.8 ± 4.4%

**Table 2:** Structures and potencies of compounds from Strategy II.

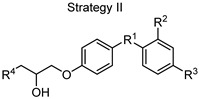					
Compound ID	R^1^	R^2^	R^3^	R^4^	% of Kv10.1 Inhibition at 10 μM (HEK-293 Cells)
**5a**	O	COOCH_3_	H	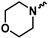	4.1 ± 2.6%
**5b**	O	NO_2_	CF_3_	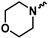	2.9 ± 5.3%
**5c**	NHCO	NO_2_	CF_3_	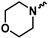	7.5 ± 3.5%
**5d**	NH	COOCH_3_	H	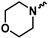	2.7 ± 1.2%
**5e**	NH	COOCH_3_	CF_3_	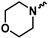	12.6 ± 6.4%
**5f**	NH	NO_2_	NO_2_	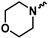	61.7± 9.1%
**6a**	O	NO_2_	CF_3_	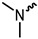	18.8 ± 4.0%
**6b**	NHCO	NO_2_	CF_3_	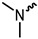	1.7 ± 6%
**6c**	NH	COOCH_3_	CF_3_	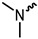	4.2 ± 5.5%
**6d**	NH	NO_2_	NO_2_	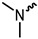	61.9 ± 9.0%
**10a**	NH	COOH	H	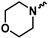	5.8 ± 5.3%
**10b**	NH	COOH	CF_3_	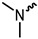	−2.1 ± 5.7%

**Table 3:** Structures and potencies of compounds from Strategy III.

Compound ID	Structure	% of K_V_10.1 Inhibition at 10 μM (HEK-293 Cells)
**14**	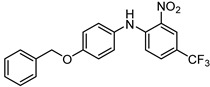	3.3 ± 1.7%
**15**	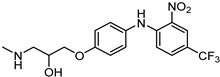	66.6 ± 4.3%

**Table 4:** Structures and potencies of compounds from Strategy IV.

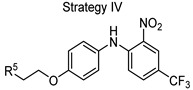		
Compound ID	R^5^	% of Kv10.1 Inhibition at 10 μM (HEK-293 Cells)
**13**	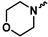	62.4 ± 3.9%
**17a**	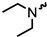	86.2 ± 6.8%
**18a**	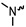	81.4 ± 5.6%
**19**	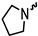	68.4 ± 14.8%
**20**	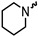	65 ± 6.0%
**21**	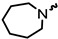	71.0 ± 4.12%

**Table 5:** Structures and potencies of compounds from Strategy V.

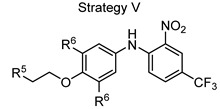			
Compound ID	R^5^	R^6^	% of Kv10.1 Inhibition at 1 μM (HEK-293 Cells)
**17b**	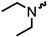	Br	38.2 ± 1.4%
**17c**	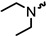	Cl	66.5 ± 5.5%
**18b**	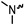	Br	73.5 ± 0.5%
**18c**	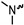	Cl	28.4 ± 12.1%

**Table 6:** IC_50_ values for K_V_10.1, hERG, and the antiproliferative activities in the MCF-7 breast cancer cell line of the synthesized compounds **17a** and **18b**.

Compound ID	IC_50_ [nM] on K_V_10.1	IC_50_ [nM] on hERG1	IC_50_ [μM] on MCF-7
**17a**	568 nM	232 nM	18.5 ± 1.5
**18b**	216 nM	4.9 nM	11.9 ± 1.0

## Data Availability

The data presented in this study are available on request from the corresponding author.

## Supplementary Materials

The following supporting information can be downloaded at: https://www.mdpi.com/article/10.3390/pharmaceutics14091963/s1, Table S1: K_V_1.3 inhibitory potencies on *Xenopus laevis* oocytes of newly designed and synthesised ZVS-08 (**1**) analogues (Strategy I), voltage clamped to determine the percentage of inhibition at 10 μM; Table S2: K_V_1.3 inhibitory potencies on *Xenopus laevis* oocytes of newly designed and synthesised ZVS-08 (**1**) analogues (Strategy II), voltage clamped to determine the percentage of inhibition at 10 μM on K_V_10.1 and hERG; Table S3: K_V_1.3 inhibitory potencies on *Xenopus laevis* oocytes of newly designed and synthesised ZVS-08 (**1**) analogues (Strategy II), voltage clamped to determine the percentage of inhibition at 10 μM on K_V_10.1 and hERG; Table S4: K_V_1.3 inhibitory potencies on *Xenopus laevis* oocytes of newly designed and synthesised ZVS-08 (**1**) analogues (Strategy IV), voltage clamped to determine the percentage of inhibition at 10 μM on K_V_10.1 and hERG; Table S5: K_V_1.3 inhibitory potencies on *Xenopus laevis* oocytes of newly designed and synthesised ZVS-08 (**1**) analogues (Strategy V), voltage clamped to determine the percentage of inhibition at 10 μM on K_V_10.1 and hERG.

## Appendix A

General procedure for synthesis of phenoxylphenol intermediates **3a**-**b** (**3a** is given as example)

Synthesis of methyl 2-(4-hydroxyphenoxy)benzoate (**3a**): To a stirring solution of methyl 2-bromobenzoate (2.14 g, 9.95 mmol) in pyridine (50 mL), hydroquinone (1.10 g, 9.95 mmol), K_2_CO_3_ (2.75 g, 19.90 mmol), and CuO (80 mg, 1.00 mmol) were added. The batch was stirred at 110 °C for 72 h and then cooled to room temperature and filtered through Celite. The solvent was removed under reduced pressure and the crude product was purified by flash column chromatography using ethyl acetate/hexane = 1:1 as a mobile phase.

Methyl 2-(4-hydroxyphenoxy)benzoate (**3a**)**:** Yield: 10%; white solid (250 mg); ^1^H NMR (400 MHz, CDCl_3_) δ 7.87 (dd, *J_1_* = 7.8 Hz, *J_2_* = 1.8 Hz, 1 H), 7.44–7.37 (m, 1 H), 7.11 (td, *J_1_* = 7.7 Hz, *J_2_* = 1.0 Hz, 1 H), 6.91–6.85 (m, 3 H), 6.83–6.78 (m, 2 H), 4.90 (brs, 1 H), and 3.86 (s, 3 H).

4-(2-Nitro-4-(trifluoromethyl)phenoxy)phenol (**3b**): Synthesised from 1-chloro-2-nitro-4-(trifluoromethyl)benzene (4.00 g, 17.73 mmol), hydroquinone (1.95 g, 17.73 mmol), K_2_CO_3_ (7.35 g, 53.20 mmol), and CuO (141 mg, 1.77 mmol) in pyridine (50 mL). Purified by flash column chromatography using ethyl acetate/hexane = 1:1 as mobile phase. Yield: 67%; yellow solid (3.565 g); ^1^H NMR (400 MHz, CDCl_3_) δ 8.21 (d, *J* = 2.0 Hz, 1 H), 7.73–7.62 (m, 1 H), 7.03–6.96 (m, 3 H), 6.95–6.87 (m, 2 H), and 6.22 (brs, 1 H).

General procedure for binding of epichlorohydrin to phenol intermediates to obtain **4a**–**4g** (**4a** is given as example)

To the argon-flushed solution of **3a** (250 mg, 1.02 mmol) in MeCN (40 mL) on ice bath K_2_CO_3_ (423 mg, 3.06 mmol) and (±)-epichlorohydrin (0.94 g, 10.20 mmol, 0.80 mL) were added. Reaction mixture was stirred at 80 °C for 16 h. K_2_CO_3_ was filtered off and the solvent was removed under reduced pressure. Crude product was purified with flash column chromatography using ethyl acetate/hexane = 1:1 as eluent to obtain **4a** (428 mg).

Methyl 2-(4-(oxiran-2-ylmethoxy)phenoxy)benzoate (**4a**): Yield: 91%; white solid (280 mg); ^1^H NMR (400 MHz, CDCl_3_) δ 7.88 (dd, *J_1_* = 7.8 Hz, *J_2_* = 1.8 Hz, 1 H), 7.41 (ddd, *J_1_* = 8.3 Hz, *J_2_* = 7.4 Hz, *J_3_* = 1.8 Hz, 1 H), 7.12 (td, *J_1_* = 7.7 Hz, *J_2_* = 1.1 Hz, 1 H), 6.98–6.85 (m, 5 H), 4.21 (dd, *J_1_* = 11.0 Hz, *J_2_* = 3.1 Hz, 1 H), 3.93 (dd, *J_1_* = 11.0 Hz, *J_2_* = 5.7 Hz, 1 H), 3.85 (s, 3 H), 3.35 (ddt, *J_1_* = 5.7 Hz, *J_2_* = 4.1 Hz, *J_3_* = 3.0 Hz, 1 H), 2.91 (dd, *J_1_* = 4.9 Hz, *J_2_* = 4.2 Hz, 1 H), and 2.76 (dd, *J_1_* = 4.9 Hz, *J_2_* = 2.7 Hz, 1 H).

2-((4-(2-Nitro-4-(trifluoromethyl)phenoxy)phenoxy)methyl)oxirane (**4b**): Synthesised from 4-(2-nitro-4-(trifluoromethyl)phenoxy)phenol (**3b**) (3.55 g, 11.87 mmol, 1.0 equiv) with 10 equivalents of (±)-epichlorohydrin and 3 equivalents of K_2_CO_3_. Purified with flash column chromatography using ethyl acetate/hexane = 1:2 as mobile phase. Yield: 61.1%; yellow oil (1.45 g); ^1^H NMR (400 MHz, DMSO) δ 8.44 (d, *J* = 2.0 Hz, 1 H), 8.02–7.92 (m, 1 H), 7.24–7.15 (m, 2 H), 7.13–7.03 (m, 3 H), 4.36 (dd, *J_1_* = 11.4 Hz, *J_2_* = 2.6 Hz, 1 H), 3.86 (dd, *J_1_* = 11.4 Hz, *J_2_* = 6.6 Hz, 1 H), 3.37–3.33 (m, 1 H), 2.86 (dd, *J_1_* = 5.0 Hz, *J_2_* = 4.3 Hz, 1 H), and 2.72 (dd, *J_1_* = 5.1 Hz, *J_2_* = 2.7 Hz, 1 H).

2-Nitro-*N*-(4-(oxiran-2-ylmethoxy)phenyl)-4-(trifluoromethyl)benzamide (**4c**): Synthesised from *N*-(4-hydroxyphenyl)-2-nitro-4-(trifluoromethyl)benzamide (**7**) (1.60 g, 4.90 mmol) and 10 equivalents of (±)-epichlorohydrin and purified with flash column chromatography using ethyl acetate/hexane = 1:1 as eluent and with dry sample loading. Yield: 33%; red crystals (618 mg); ^1^H NMR (400 MHz, DMSO) δ 10.66 (s, 1 H), 8.50 (s, 1 H), 8.28 (dd, *J_1_* = 8.0 Hz, *J_2_* = 1.1 Hz, 1 H), 8.03 (d, *J* = 7.9 Hz, 1 H), 7.65–7.51 (m, 2 H), 7.04–6.92 (m, 2 H), 4.32 (dd, *J_1_* = 11.4 Hz, *J_2_* = 2.7 Hz, 1 H), 3.82 (dd, *J_1_* = 11.4 Hz, *J_2_* = 6.5 Hz, 1 H), 3.36–3.33 (m, 1 H), 2.87–2.81 (m, 1 H), and 2.71 (dd, *J_1_* = 5.1 Hz, *J_2_* = 2.7 Hz, 1 H).

Methyl 2-((4-(oxiran-2-ylmethoxy)phenyl)amino)benzoate (**4d**): resynthesized according to described procedures [26].

Methyl 2-((4-(oxiran-2-ylmethoxy)phenyl)amino)-5-(trifluoromethyl)benzoate (**4e**): Synthesised from methyl 2-((4-hydroxyphenyl)amino)-5-(trifluoromethyl)benzoate (**9b**) (330 mg, 1.06 mmol, 1.0 equiv.) with 10 equivalents of (±)-epichlorohydrin and purified with flash column chromatography using ethyl acetate/hexane = 1:4 as eluent. Yield: 72%; white solid (281 mg); ^1^H NMR (400 MHz, DMSO) δ 9.49 (s, 1 H), 8.10 (d, *J* = 1.7 Hz, 1 H), 7.63 (dd, *J_1_* = 9.0 Hz, *J_2_* = 2.3 Hz, 1 H), 7.29–7.19 (m, 2 H), 7.08–7.00 (m, 2 H), 6.97 (d, *J* = 9.0 Hz, 1 H), 4.35 (dd, *J_1_* = 11.4 Hz, *J_2_* = 2.6 Hz, 1 H), 3.89 (s, 3 H), 3.88–3.80 (m, 1 H), 3.40–3.33 (m, 1 H), 2.86 (dd, *J_1_* = 5.0 Hz, *J_2_* = 4.3 Hz, 1 H), and 2.72 (dd, *J_1_* = 5.1 Hz, *J_2_* = 2.7 Hz, 1 H).

2-Nitro-*N*-(4-(oxiran-2-ylmethoxy)phenyl)-4-(trifluoromethyl)aniline (**4f**): resynthesized according to described procedures [26].

2,4-Dinitro-*N*-(4-(oxiran-2-ylmethoxy)phenyl)aniline (**4g**): Synthesised from 4-((2,4-dinitrophenyl)amino)phenol (**12b**) (1.93 g, 7.0 mmol, 1.0 equiv.), 10 equivalents of (±)-epichlorohydrin and K_2_CO_3_ (2.91 g, 21.1 mmol, 3.0 equiv.). Purified with flash column chromatography ethyl acetate/hexane = 1:1 as eluent. Yield: 78%; red solid (1.8 g); ^1^H NMR (400 MHz, DMSO) δ 10.10 (s, 1 H), 8.89 (d, *J* = 2.7 Hz, 1 H), 8.20 (dd, *J_1_* = 9.6 Hz, *J_2_* = 2.8 Hz, 1 H), 7.38–7.23 (m, 2 H), 7.16–7.04 (m, 2 H), 6.96 (d, *J* = 9.6 Hz, 1 H), 4.39 (dd, *J_1_* = 11.4 Hz, *J_2_* = 2.6 Hz, 1 H), 3.88 (dd, *J_1_* = 11.4 Hz, *J_2_* = 6.6 Hz, 1 H), 3.43–3.32 (m, 1 H), 2.87 (dd, *J_1_* = 5.0 Hz, *J_2_* = 4.3 Hz, 1 H), and 2.73 (dd, *J_1_* = 5.1, *J_2_* = 2.7 Hz, 1 H).

General procedure for opening of the epoxide with morpholine to obtain **5a**-**5f** (**5a** is given as example)

To a stirring solution of methyl 2-(4-(oxiran-2-ylmethoxy)phenoxy)benzoate (**4a**) (100 mg, 0.333 mmol) in acetonitrile (20 mL), morpholine (87 mg, 0.999 mmol, 0.086 mL, 3.0 equiv.) was added and stirred for 72 h at room temperature. After 72 h, solvent was removed under reduced pressure and product was purified with flash column chromatography using dichloromethane/methanol/ammonium = 20:1:0.1.

Methyl 2-(4-(2-hydroxy-3-morpholinopropoxy)phenoxy)benzoate (**5a**): Yield: 40%; white solid (50 mg); ^1^H NMR (400 MHz, MeOD) δ 7.82 (dd, *J_1_* = 7.8 Hz, *J_2_* = 1.7 Hz, 1 H), 7.45 (ddd, *J_1_* = 8.4 Hz, *J_2_* = 7.4 Hz, *J_3_* = 1.8 Hz, 1 H), 7.13 (td, *J_1_* = 7.7 Hz, *J_2_* = 1.0 Hz, 1 H), 6.99–6.88 (m, 4 H), 6.85 (dd, *J_1_* = 8.3 Hz, *J_2_* = 0.8 Hz, 1 H), 4.87 (brs, 1 H), 4.12 (ddd, *J_1_* = 9.2 Hz, *J_2_* = 7.4 Hz, *J_3_* = 5.3 Hz, 1 H), 3.99 (dd, *J_1_* = 9.7 Hz, *J_2_* = 4.1 Hz, 1 H), 3.91 (dd, *J_1_* = 9.8 Hz, *J_2_* = 5.9 Hz, 1 H), 3.80 (s, 3 H), 3.74–3.60 (m, 4 H), 2.62–2.47 (m, 6 H); ^13^C NMR (101 MHz, MeOD) δ 168.01, 158.65, 156.71, 151.89, 134.71, 132.55, 123.76, 123.47, 121.21, 120.07, 116.78, 72.42, 68.11, 67.78, 62.57, 55.35, and 52.62; HRMS (ESI^+^) for C_21_H_26_NO_6_ ([M+H]^+^) calculated 388.1755 found 388.1745; HPLC retention time: 2.793 min (96.90% at 254 nm).

1-Morpholino-3-(4-(2-nitro-4-(trifluoromethyl)phenoxy)phenoxy)propan-2-ol (**5b**): Synthesised from 2-((4-(2-nitro-4-(trifluoromethyl)phenoxy)phenoxy)methyl)oxirane (**4b**) (500 mg, 1.41 mmol) in acetonitrile (30 mL) and three equivalents of morpholine (368 mg, 4.22 mmol, 0.364 mL). Purified with flash column chromatography using dichloromethane/methanol: 20/1 as eluent. Yield: 21%; yellow oil (130 mg); ^1^H NMR (400 MHz, CDCl_3_) δ 8.21 (d, *J* = 1.9 Hz, 1 H), 7.71–7.64 (m, 1 H), 7.09–7.03 (m, 2 H), 6.99 (dt, *J_1_* = 5.7 Hz, *J_2_* = 3.3 Hz, 3 H), 4.18 (dq, *J_1_* = 9.2 Hz, *J_2_* = 4.7 Hz, 1 H), 4.05–3.95 (m, 2 H), 3.85–3.70 (m, 5 H), 2.81–2.71 (m, 2 H), 2.70–2.60 (m, 2 H), 2.60–2.52 (m, 2 H); ^13^C NMR (101 MHz, CDCl_3_) δ 156.71, 154.78, 147.72, 139.90, 130.86 (q, *J* = 3.4 Hz), 124.52 (q, *J* = 34.5 Hz), 123.64 (q, *J* = 3.9 Hz), 123.01 (q, *J* = 271.0 Hz), 121.80, 118.60, 116.27, 70.77, 66.86, 65.34, 61.18, and 53.87. HRMS (ESI^+^) for C_20_H_22_F_3_N_2_O_6_ ([M+H]^+^) calculated 443.1425 found 443.1415; HPLC retention time: 3.600 min (98.51% at 254 nm).

*N*-(4-(2-Hydroxy-3-morpholinopropoxy)phenyl)-2-nitro-4-(trifluoromethyl)benzamide (**5c**): Synthesised from 2-nitro-*N*-(4-(oxiran-2-ylmethoxy)phenyl)-4-(trifluoromethyl)benzamide (**4c**) (300 mg, 0.78 mmol, 1.0 equiv.) and three equivalents of morpholine in dichloromethane (40 mL). Reaction was stopped after 72 h. Mobile phase for flash column chromatography was dichloromethane/methanol: 20/1. Yield: 41%; yellow solid (150 mg); ^1^H NMR (400 MHz, DMSO) δ 10.63 (s, 1 H), 8.50 (d, *J* = 1.0 Hz, 1 H), 8.28 (dd, *J_1_* = 8.0 Hz, *J_2_* = 1.1 Hz, 1 H), 8.02 (d, *J* = 7.9 Hz, 1 H), 7.59–7.49 (m, 2 H), 7.00–6.91 (m, 2 H), 4.87 (d, *J* = 4.5 Hz, 1 H), 4.00–3.92 (m, 2 H), 3.86 (dd, *J_1_ =* 11.0 Hz, *J_2_* = 7.3 Hz, 1 H), 3.57 (t, *J* = 4.6 Hz, 4 H), 2.48–2.30 (m, 6 H); ^13^C NMR (101 MHz, MeOD) δ 165.61, 157.71, 147.99, 137.53, 133.66 (q, *J* = 34.2 Hz), 132.56, 131.82 (q, *J* = 3.4 Hz), 131.62, 124.20 (q, *J* = 271.0 Hz), 123.34, 122.88 (q, *J* = 3.8 Hz), 115.91, 72.19, 68.17, 67.84, 62.60, and 55.40. HRMS (ESI^+^) for C_21_H_23_F_3_N_3_O_6_ ([M+H]^+^) calculated 470.1534 found 470.1523; HPLC retention time: 2.930 min (99.76% at 254 nm).

Methyl 2-((4-(2-hydroxy-3-morpholinopropoxy)phenyl)amino)benzoate (**5d**): Synthesised from methyl 2-((4-(oxiran-2-ylmethoxy)phenyl)amino)benzoate (**4d**) (590 mg, 1.97 mmol, 1.0 equiv.) and three equivalents of morpholine (515 mg, 5.91 mmol, 0.512 mL) for 72 h at room temperature. Purified with flash column chromatography using ethyl acetate/hexane = 2:1 and one more column with dichloromethane/methanol/ammonium = 20:1:0.1 as eluent and dry sample loading. Yield: 32%; yellow oil (240 mg); ^1^H NMR (400 MHz, DMSO) δ 9.15 (s, 1 H), 7.86 (dd, *J_1_* = 8.0 Hz, *J_2_* = 1.6 Hz, 1 H), 7.34 (ddd, *J_1_* = 8.6 Hz, *J_2_* = 7.2 Hz, *J_3_* = 1.6 Hz, 1 H), 7.21–7.13 (m, 2 H), 7.00–6.95 (m, 2 H), 6.93 (dd, *J_1_* = 8.6 Hz, *J_2_* = 0.7 Hz, 1 H), 6.71 (ddd, *J_1_* = 8.1 Hz, *J_2_* = 7.1 Hz, *J_3_* = 1.1 Hz, 1 H), 4.87 (d, *J* = 4.7 Hz, 1 H), 4.01–3.92 (m, 2 H), 3.87 (dd, *J_1_* = 10.0 Hz, *J_2_* = 6.4 Hz, 1 H), 3.85 (s, 3 H), 3.56 (t, *J* = 4.6 Hz, 4 H), 2.48–2.31 (m, 6 H). ^13^C NMR (101 MHz, MeOD) δ 170.24, 157.48, 150.64, 135.28, 134.88, 132.56, 126.63, 117.38, 116.53, 114.31, 112.04, 72.22, 68.14, 67.81, 62.61, 55.38, and 52.16; HRMS (ESI^+^) for C_21_H_27_N_2_O_5_ ([M+H]^+^) calculated 387.1915 found 387.1905; HPLC retention time: 3.940 min (98.44% at 254 nm).

Methyl 2-((4-(2-hydroxy-3-morpholinopropoxy)phenyl)amino)benzoate (**5e**): Synthesised from methyl 2-((4-(oxiran-2-ylmethoxy)phenyl)amino)-5-(trifluoromethyl)benzoate (**4e**) (590 mg, 1.97 mmol) with three equivalents of morpholine (100 mg, 1.14 mmol, 0.099 mL) at room temperature for 72 h. Purified with flash column chromatography using ethyl acetate/hexane = 1:4. Yield: 11%; yellow oil (100 mg); ^1^H NMR (400 MHz, CDCl_3_) δ 9.57 (s, 1 H), 8.21 (d, *J* = 1.4 Hz, 1 H), 7.43 (dd, *J_1_* = 9.0 Hz, *J_2_* = 2.2 Hz, 1 H), 7.21–7.12 (m, 2 H), 7.02–6.89 (m, 3 H), 4.13 (tt, *J_1_* = 9.5 Hz, *J_2_* = 4.9 Hz, 1 H), 4.05–3.97 (m, 2 H), 3.93 (s, 3 H), 3.81–3.66 (m, 4 H), 2.76–2.64 (m, 2 H), 2.64–2.53 (m, 2 H), 2.54–2.43 (m, 2 H); ^13^C NMR (101 MHz, CDCl_3_) δ 168.38, 156.75, 151.92, 132.51, 130.71 (q, *J* = 3.3 Hz), 129.46 (q, *J* = 3.9 Hz), 124.50 (q, *J* = 270.6 Hz), 126.74, 117.98 (q, *J* = 33.2 Hz), 115.69, 113.42, 109.99, 70.64, 67.15, 65.52, 61.14, 53.90, and 52.16; HRMS (ESI^+^) for C_22_H_26_F_3_N_2_O_5_ ([M+H]^+^) calculated 455.1788 found 455.1777; HPLC retention time: 3.953 min (96.49% at 254 nm).

1-(4-((2,4-Dinitrophenyl)amino)phenoxy)-3-morpholinopropan-2-ol (**5f**): Synthesised from 2,4-dinitro-*N*-(4-(oxiran-2-ylmethoxy)phenyl)aniline (**4g**) (442 mg, 1.34 mmol, 1.0 equiv.) with morpholine (465 mg, 5.34 mmol, 0.470 mL, 4.0 equiv.) in acetonitrile (50 mL) at room temperature for 72 h. Purified with flash column chromatography using dichloromethane/methanol/NH_3_ = 20:1:0.01 as eluent and Biotage Isolera One System reversed-phase chromatography (Biotage SNAP Cartridge KP-C18-HS 12 g column), MF: gradient 0,1% trifluoroacetic acid in H_2_O/acetonitrile). Yield: 18%; red amorph solid (100 mg); ^1^H NMR (400 MHz, CDCl_3_) δ 9.87 (s, 1 H), 9.17 (d, *J* = 2.6 Hz, 1 H), 8.21–8.07 (m, 1 H), 7.25–7.19 (m, 2 H), 7.09–6.94 (m, 3 H), 4.16 (td, *J_1_* = 9.4 Hz, *J_2_* = 4.5 Hz, 1 H), 4.09–3.98 (m, 2 H), 3.85–3.68 (m, 4 H), 2.79–2.44 (m, 6 H); ^13^C NMR (101 MHz, CDCl_3_) δ 158.35, 148.03, 137.24, 130.88, 130.01, 129.64, 127.59, 124.25, 116.19, 116.09, 70.69, 67.10, 65.39, 61.03, and 53.86; HRMS (ESI^+^) for C_19_H_23_N_4_O_7_ ([M+H]^+^) calculated 419.1561 found 419.1550; HPLC retention time: 3.430 min (99.08% at 254 nm).

General procedure for opening of the epoxide with dimethylamine to obtain **6a**–**6d** (**6a** is given as example)

To a stirring solution of methyl 2-(4-(oxiran-2-ylmethoxy)phenoxy)benzoate (**4b**) (500 mg, 1.4 mmol, 1.0 equiv) in acetonitrile (30 mL), Me_2_NH × HCl (460 mg, 5.63 mmol, 4.0 equiv) and K_2_CO_3_ (580 mg, 4.2 mmol, 3.0 equiv) were added and the batch was stirred at 70 °C for 24 h. The solvent was removed under reduced pressure and the crude product was purified with Biotage Isolera One System reversed-phase chromatography (Biotage SNAP Cartridge KP-C18-HS 12 g column), MF: gradient 0,1% trifluoroacetic acid in H_2_O/acetonitrile). Fractions containing 1-(dimethylamino)-3-(4-(2-nitro-4-(trifluoromethyl)phenoxy)phenoxy)propan-2-ol (**6a**) were combined, acetonitrile was removed under reduced pressure and pH of remaining water phase was set to 8, and product was extracted into ethyl acetate (2 × 25 mL). Organic phase was washed with brine (2 × 25 mL) dried over Na_2_SO_4_ filtered and the solvent removed under reduced pressure to obtain **6a** (100 mg).

1-(Dimethylamino)-3-(4-(2-nitro-4-(trifluoromethyl)phenoxy)phenoxy)propan-2-ol (**6a**): Yield: 18%; yellow oil (100 mg); ^1^H NMR (400 MHz, CDCl_3_) δ 8.19 (d, *J* = 1.8 Hz, 1 H), 7.68 (dd, *J_1_* = 8.9 Hz, *J_2_* = 2.0 Hz, 1 H), 7.09–6.93 (m, 5 H), 4.36 (brs, 1 H), 4.22–4.13 (m, 1 H), 4.01 (d, *J* = 4.9 Hz, 2 H), 2.77–2.67 (m, 1 H), 2.55 (dd, *J_1_* = 12.4 Hz, *J_2_* = 2.7 Hz, 1 H), 2.45 (s, 6 H); ^13^C NMR (101 MHz, CDCl_3_) δ 156.43, 154.67, 147.80, 139.85, 130.95 (q, *J* = 3.7 Hz), 124.55 (q, *J* = 34.5 Hz), 123.60 (q, *J* = 3.8 Hz), 123.02 (q, *J* = 271.0 Hz), 121.75, 118.73, 116.30, 70.54, 65.41, 61.62, and 45.07; HRMS (ESI^+^) for C_18_H_20_F_3_N_2_O_5_ ([M+H]^+^) calculated 401.1319 found 401.1310; HPLC retention time: 3.593 min (95.01% at 254 nm).

*N*-(4-(3-(Dimethylamino)-2-hydroxypropoxy)phenyl)-2-nitro-4-(trifluoromethyl)benzamide (**6b**): Synthesised from 2-nitro-*N*-(4-(oxiran-2-ylmethoxy)phenyl)-4-(trifluoromethyl)benzamide (**4c**) (300 mg, 0.78 mmol) and six equivalents of Me_2_NH × HCl in acetonitrile (40 mL). Purified with Biotage Isolera One System reversed-phase chromatography (Biotage SNAP Cartridge KP-C18-HS 12 g column), MF: gradient 0,1% trifluoroacetic acid in H_2_O/acetonitrile). Yield: 27%; yellow solid (90 mg); ^1^H NMR (400 MHz, DMSO) δ 10.77 (s, 1 H), 9.82 (s, 1 H), 8.50 (d, *J* = 0.9 Hz, 1 H), 8.28 (dd, *J_1_* = 8.0 Hz, *J_2_* = 1.1 Hz, 1 H), 8.03 (d, *J* = 7.9 Hz, 1 H), 7.64–7.53 (m, 2 H), 7.04–6.91 (m, 2 H), 6.00 (d, *J* = 3.9 Hz, 1 H), 4.29 (d, *J* = 2.5 Hz, 1 H), 4.02–3.88 (m, 2 H), 3.40–3.13 (m, 2 H), 2.84 (d, *J* = 11.5 Hz, 6 H); ^13^C NMR (101 MHz, MeOD) δ 165.70, 157.18, 148.00, 137.49, 133.73 (q, *J* = 34.2 Hz), 133.05, 131.86 (q, *J* = 3.5 Hz), 130.79, 123.76 (q, *J* = 271.0 Hz), 123.40, 122.90 (q, *J* = 3.3 Hz), 115.94, 71.30, 65.37, and 61.01. HRMS (ESI^+^) for C_19_H_21_F_3_N_3_O_5_ ([M+H]^+^) calculated 428.1428 found 428.1420; HPLC retention time: 2.893 min (97.37% at 254 nm).

Methyl 2-((4-(3-(dimethylamino)-2-hydroxypropoxy)phenyl)amino)-5-(trifluoromethyl)benzoate (**6c**): Synthesised from methyl 2-((4-(oxiran-2-ylmethoxy)phenyl)amino)-5-(trifluoromethyl)benzoate (**4e**) (140 mg, 0.381 mmol), K_2_CO_3_ (157 mg, 1.14 mmol, 3.0 equiv.) and Me_2_NH × HCl (186 mg, 2.28 mmol, 6.0 equiv) in acetonitrile (35 mL) at 70 °C overnight. Yield: 64%; yellow oil (100 mg); ^1^H NMR (400 MHz, CDCl_3_) δ 9.56 (s, 1 H), 8.21 (d, *J* = 1.4 Hz, 1 H), 7.42 (dd, *J_1_* = 9.0 Hz, *J_2_* = 2.3 Hz, 1 H), 7.21–7.10 (m, 2 H), 7.02–6.90 (m, 3 H), 4.12–4.04 (m, 1 H), 4.03–3.96 (m, 2 H), 3.93 (s, 3 H), 2.63–2.50 (m, 1 H), 2.40 (dd, *J_1_* = 12.2 Hz, *J_2_* = 3.8 Hz, 1 H), 2.34 (s, 6 H). ^13^C NMR (101 MHz, CDCl_3_) δ 168.32, 156.81, 151.90, 132.36, 130.66 (q, *J* = 3.2 Hz), 129.40 (q, *J* = 3.9 Hz), 126.68, 124.48 (q, *J* = 270.6 Hz), 117.87 (q, *J* = 33.2 Hz), 115.66, 113.40, 109.92, 70.84, 66.31, 61.92, 52.11, and 45.67; HRMS (ESI^+^) C_20_H_24_F_3_N_2_O_4_ ([M+H]^+^) calculated 413.1683 found 413.1672; HPLC retention time: 3.950 min (98.29% at 254 nm).

1-(Dimethylamino)-3-(4-((2,4-dinitrophenyl)amino)phenoxy)propan-2-ol (**6d**): Synthesised from 2,4-dinitro-*N*-(4-(oxiran-2-ylmethoxy)phenyl)aniline (**4g**) (170 mg, 0.706 mmol, 1.0 equiv.), Me_2_NH × HCl (344 mg, 4.220 mmol, 6.0 equiv.) and K_2_CO_3_ (291 mg, 2.110 mmol, 3.0 equiv.) in acetonitrile (30 mL) at 70 °C for 24 h. Purified with flash column chromatography using dichloromethane/methanol/NH_3_ = 20:1:0.01 as eluent and Biotage Isolera One System reversed-phase chromatography (Biotage SNAP Cartridge KP-C18-HS 12 g column), MF: gradient 0,1% trifluoroacetic acid in H_2_O/acetonitrile). Yield: 30%; red solid (80 mg); ^1^H NMR (400 MHz, DMSO) δ 10.09 (s, 1 H), 8.89 (d, *J* = 2.7 Hz, 1 H), 8.20 (dd, *J_1_* = 9.6 Hz, *J_2_* = 2.8 Hz, 1 H), 7.33–7.22 (m, 2 H), 7.12–7.04 (m, 2 H), 6.97 (d, *J* = 9.6 Hz, 1 H), 4.88 (d, *J* = 3.8 Hz, 1 H), 4.02 (dd, *J_1_* = 9.0 Hz, *J_2_* = 2.9 Hz, 1 H), 3.97–3.86 (m, 2 H), 2.46–2.27 (m, 2 H), and 2.21 (s, 6 H); ^13^C NMR (101 MHz, CDCl_3_) δ 158.39, 148.03, 137.16, 130.80, 129.97, 129.51, 127.53, 124.19, 116.16, 116.11, 70.86, 66.15, 61.78, and 45.65. HRMS (ESI^+^) for C_17_H_21_N_4_O_6_ ([M+H]^+^) calculated 377.1456 found 377.1447; HPLC retention time: 3.460 min (99.44% at 254 nm).

Synthesis of amide intermediate **7**

To a solution of 2-nitro-4-(trifluoromethyl)benzoic acid (3.00 g, 12.76 mmol, 1.0 equiv.) in dichloromethane (100 mL), two drops of DMF and oxalyl chloride (4.86 g, 38.28 mmol, 3.28 mL, 3.0 equiv.) were added while ice cooling and the batch was stirred at 40 °C for 24 h. Solvent was removed under reduced pressure and crude product was resolved in the acetonitrile (20 mL) on the ice bath. To the ice cooled solution, 4-aminophenol (1.67 g, 15.31 mmol, 1.2 equiv.) and triethylamine (3.87 g, 51.04 mmol, 5.33 mL, 3.0 equiv.) were added dropwise and then reaction mixture was heated at reflux overnight. The solvent was removed under reduced pressure. Product was further purified using flash column chromatography with ethyl acetate/hexane = 1:2 as mobile phase and with dry sample loading.

*N*-(4-Hydroxyphenyl)-2-nitro-4-(trifluoromethyl)benzamide (**7**): Yield: 38%; red solid (1.6 g); ^1^H NMR (400 MHz, DMSO) δ 10.52 (s, 1 H), 9.34 (s, 1 H), 8.49 (d, *J* = 0.8 Hz, 1 H), 8.26 (dd, *J_1_* = 8.0 Hz, *J_2_* = 1.1 Hz, 1 H), 8.00 (d, *J* = 7.9 Hz, 1 H), 7.49–7.36 (m, 2 H), and 6.83–6.68 (m, 2 H).

General procedure for synthesis of benzoic acid derivatives **8a**–**b** (**8a** is given as example)

To an argon-flushed solution of 2-bromobenzoic acid (4.00 g, 19.90 mmol, 1.0 equiv.) in 2-ethoxyethanol (20 mL), CuO (63 mg, 0.80 mmol, 0.04 equiv.), Cu (114 mg, 1.79 mmol, 0.09 equiv.), K_2_CO_3_ (5.50 g, 39.80 mmol), and 4-aminophenol (2.28 g, 20.89 mmol, 1.05 equiv.) were added and the batch was stirred at 130 °C for 16 h. Solution was cooled to room temperature and then water (100 mL) was added. Mixture was filtered through Celite and then pH was set to 5 to precipitate black solid that was filtered off. Black precipitate was dissolved in the 5% solution of Na_2_CO_3_ (250 mL) and solution was filtered through Celite. pH in solution was then set to 5 to precipitate black solid that was filtered off and dried overnight.

2-((4-Hydroxyphenyl)amino)benzoic acid (**8a**): Yield: 50%; black solid (2.28 g); ^1^H NMR (400 MHz, DMSO) δ 12.90 (s, 1 H), 9.42 (brs, 1 H), 9.34 (brs, 1 H), 7.84 (dd, *J_1_* = 8.0 Hz, *J_2_* = 1.6 Hz, 1 H), 7.30 (ddd, *J_1_* = 8.7 Hz, *J_2_* = 7.1 Hz, *J_3_* = 1.7 Hz, 1 H), 7.08–7.02 (m, 2 H), 6.86 (dd, *J_1_* = 8.5 Hz, *J_2_* = 0.7 Hz, 1 H), 6.83–6.77 (m, 2 H), and 6.69–6.62 (m, 1 H).

2-((4-Hydroxyphenyl)amino)-5-(trifluoromethyl)benzoic acid (**8b**): Synthesised from 2-bromo-5-(trifluoromethyl)benzoic acid (3.00 g, 11.15 mmol, 1.0 equiv.), 4-aminophenol (1.46 g, 13.38 mmol, 1.2 equiv.), K_2_CO_3_ (4.62 g, 33.46 mmol, 3.0 equiv.), CuO (35 mg, 0.45 mmol, 0.04 equiv.), and Cu (64 mg, 1.00 mmol, 0.09 equiv.) in 2-ethoxyethanol (20 mL). Yield: 60%; black solid (2 g); ^1^H NMR (400 MHz, DMSO) δ 9.69 (s, 1 H), 9.54 (brs, 1 H), 8.08 (d, *J* = 1.7 Hz, 1 H), 7.58 (dd, *J_1_* = 9.0 Hz, *J_2_* = 2.2 Hz, 1 H), 7.14–7.06 (m, 2 H), 6.91 (d, *J* = 8.9 Hz, 1 H), and 6.86–6.79 (m, 2 H).

General procedure for synthesis of methyl ester derivatives **9a**–**b** (**9a** is given as an example)

2-((4-Hydroxyphenyl)amino)benzoic acid (**8a**) (2.28 g, 9.96 mmol, 1.0 equiv.) was dissolved in anhydrous methanol (100 mL) and then thionyl chloride (11.85 g, 99.59 mmol, 7.22 mL, 10.0 equiv) was slowly added while ice cooling. The batch was stirred overnight at 70 °C. The solvent was removed under reduced pressure, crude product was purified with flash column chromatography using ethyl acetate/hexane = 1:4 as mobile phase and with dry sample loading.

Methyl 2-((4-hydroxyphenyl)amino)benzoate (**9a**): Yield: 55%; white solid (1.33 g); ^1^H NMR (400 MHz, DMSO) δ 9.38 (s, 1 H), 9.08 (s, 1 H), 7.85 (dd, *J_1_ =* 8.0 Hz, *J_2_* = 1.6 Hz, 1 H), 7.32 (ddd, *J_1_ =* 8.6 Hz, *J_2_* = 7.2 Hz, *J_3_* = 1.6 Hz, 1 H), 7.09–7.02 (m, 2 H), 6.86 (dd, *J_1_* = 8.6 Hz, *J_2_* = 0.8 Hz, 1 H), 6.83–6.76 (m, 2 H), 6.67 (ddd, *J* = 8.1 Hz, *J_2_* = 7.1 Hz, *J_3_* = 1.1 Hz, 1 H), and 3.84 (s, 3 H).

Methyl 2-((4-hydroxyphenyl)amino)-5-(trifluoromethyl)benzoate (**9b**): Synthesised from 2-((4-hydroxyphenyl)amino)-5-(trifluoromethyl)benzoic acid (**7b**) (2.00 g, 6.73 mmol, 1.0 equiv.) with thionyl chloride (8.00 g, 67.29 mmol, 4.88 mL, 10.0 equiv.) in anhydrous methanol (75 mL). Purified with flash column chromatography using ethyl acetate/hexane = 1:4 as mobile phase. Yield: 16%; white solid (330 mg); ^1^H NMR (400 MHz, DMSO) δ 9.54 (s, 1 H), 9.41 (s, 1 H), 8.09 (d, *J* = 1.7 Hz, 1 H), 7.61 (dd, *J_1_* = 9.1 Hz, *J_2_* = 2.2 Hz, 1 H), 7.10 (d, *J* = 8.7 Hz, 2 H), 6.91 (d, *J* = 9.0 Hz, 1 H), 6.86–6.73 (m, 2 H), and 3.88 (s, 3 H).

General procedure for hydrolysis of methyl ester to obtain **10a**–**b** (**10a** is given as an example)

Methyl 2-((4-(2-hydroxy-3-morpholinopropoxy)phenyl)amino)benzoate (**5d**) (123 mg, 0.318 mmol, 1.0 equiv.) was dissolved in ethanol (20 mL) and then solution of NaOH (19 mg, 0.477 mmol, 1.5 equiv.) in 1 mL of water was added and stirred at room temperature for 72 h. Solvent was removed under reduced pressure to obtain product **10a**.

2-((4-(2-Hydroxy-3-morpholinopropoxy)phenyl)amino)benzoic acid (**10a**): Yield: 100%; white solid (118 mg); ^1^H NMR (400 MHz, DMSO) δ 11.50 (s, 1 H), 7.90 (dd, *J_1_* = 7.7 Hz, *J_2_* = 1.6 Hz, 1 H), 7.11–7.02 (m, 3 H), 6.98 (d, *J* = 7.6 Hz, 1 H), 6.88 (d, *J* = 8.9 Hz, 2 H), 6.62–6.49 (m, 1 H), 4.95 (brs, 1 H), 4.01–3.89 (m, 2 H), 3.88–3.75 (m, 1 H), 3.56 (t, *J* = 4.6 Hz, 4 H), 2.48–2.28 (m, 6 H); ^13^C NMR (101 MHz, DMSO) δ 171.88, 153.58, 146.66, 135.84, 132.10, 129.92, 122.22, 121.71, 115.62, 115.33, 111.85, 71.34, 66.45, 66.29, 61.60, and 54.09; HRMS (ESI^+^) for C_20_H_25_N_2_O_5_ ([M+H]^+^) calculated 373.1758 found 373.1751; HPLC retention time: 3.033 min (99.37% at 254 nm).

2-((4-(3-(Dimethylamino)-2-hydroxypropoxy)phenyl)amino)-5-(trifluoromethyl)benzoic acid (**10b**): Synthesised from methyl 2-((4-(3-(dimethylamino)-2-hydroxypropoxy)phenyl)amino)-5-(trifluoromethyl)benzoate (**6c**) (28 mg, 0.07 mmol, 1.0 equiv.) with solution of NaOH (4.2 mg, 0.11 mmol, 1.5 equiv.) in 1 mL of water and in ethanol (5 mL) at room temperature for 72 h. Yield: 100%; white solid (28 mg); ^1^H NMR (400 MHz, DMSO) δ 12.10 (s, 1 H), 8.16 (d, *J* = 2.2 Hz, 1 H), 7.33 (dd, *J_1_* = 8.7 Hz, *J_2_* = 2.3 Hz, 1 H), 7.10 (d, *J* = 8.8 Hz, 2 H), 6.98 (d, *J* = 8.6 Hz, 1 H), 6.93 (d, *J* = 8.9 Hz, 2 H), 4.87 (brs, 1 H), 4.00–3.77 (m, 3 H), 2.44–2.24 (m, 2 H), 2.18 (s, 6 H). ^13^C NMR (101 MHz, MeOD) δ 174.97, 157.11, 151.89, 135.41, 130.67 (q, *J* = 3.7 Hz), 128.77 (q, *J* = 3.5 Hz), 126.55 (q, *J* = 269.4 Hz), 125.95, 120.53, 118.11 (q, *J* = 32.4 Hz), 116.51, 112.99, 72.34, 68.80, 63.31, and 46.21; HRMS (ESI^+^) for C_19_H_22_F_3_N_2_O_4_ ([M+H]^+^) calculated 399.1526, found 399.1519; HPLC retention time: 3.830 min (95.01% at 254 nm).

1-Chloro-2-nitro-4-(trifluoromethyl)benzene (**11**): resynthesized according to described procedure [26].

General procedure for synthesis of phenol intermediates **12a**–**d** (**12b** is given as an example)

To a stirred solution of 1-fluoro-2,4-dinitrobenzene (3.00 g, 14.80 mmol, 1.0 equiv.) in acetonitrile (150 mL), NaHCO_3_ (3.73 g, 44.40 mmol, 3.0 equiv.) and 4-aminophenol (1.94 g, 17.77 mmol, 1.2 equiv.) were added and the mixture was stirred at 90 °C for 24 h. After reaction mixture was cooled to room temperature and NaHCO_3_ was filtered off, the solvent was removed under reduced pressure. The crude product was purified with flash column chromatography using dichloromethane/methanol = 20:1) as eluent and dry sampling loading to obtain **12b**.

4-((2-Nitro-4-(trifluoromethyl)phenyl)amino)phenol (**12a**): resynthesized according to described procedure [26].

4-((2,4-Dinitrophenyl)amino)phenol (**12b**): Yield: 47%; red solid (1.93 g); ^1^H NMR (400 MHz, DMSO) δ 10.04 (s, 1 H), 9.73 (s, 1 H), 8.86 (d, *J* = 2.7 Hz, 1 H), 8.17 (dd, *J_1_* = 9.6 Hz, *J_2_* = 2.8 Hz, 1 H), 7.21–7.08 (m, 2 H), 6.94 (d, *J* = 9.6 Hz, 1 H), and 6.91–6.79 (m, 2 H).

2,6-Dibromo-4-((2-nitro-4-(trifluoromethyl)phenyl)amino)phenol (**12c**)

Synthesised from 1-chloro-2-nitro-4-(trifluoromethyl)benzene (**11**) (2.5 g, 11.1 mmol, 1.0 equiv), 4-amino-2,6-dibromophenol (3.55 g, 13.3 mmol, 1.2 equiv), and NaHCO_3_ (2 g, 22.2 mmol, 2.0 equiv) in acetonitrile (125 mL) at 90 °C. Purified with flash column chromatography using ethyl acetate/hexane = 1:3 as eluent. Yield: 31%; orange solid (1.59 g); ^1^H NMR (400 MHz, CDCl_3_) δ 9.51 (s, 1 H), 8.56–8.45 (m, 1 H), 7.59 (dd, *J*_*1*_ = 9.0, *J*_*2*_ = 2.1 Hz, 1 H), 7.49–7.41 (m, 2 H), 7.08 (d, *J* = 9.0 Hz, 1 H), and 5.98 (s, 1 H).

2,6-Dichloro-4-((2-nitro-4-(trifluoromethyl)phenyl)amino)phenol (**12d**)

Synthesised from 1-chloro-2-nitro-4-(trifluoromethyl)benzene (**11**) (2.5 g, 11.1 mmol, 1.0 equiv), 4-amino-2,6-dichlorophenol (2.37 g, 13.3 mmol, 1.2 equiv), and NaHCO_3_ (2 g, 22.2 mmol, 2.0 equiv) in acetonitrile (125 mL) at 90 °C. Purified with flash column chromatography using ethyl acetate/hexane = 1:3 as eluent. Yield: 25%; orange solid (1.00 g); ^1^H NMR (400 MHz, CDCl_3_) δ 9.50 (s, 1 H), 8.61–8.35 (m, 1 H), 7.59 (dd, *J*_1_ = 9.0 Hz, *J*_2_ = 2.1 Hz, 1 H), 7.26 (s, 2 H), 7.09 (d, J = 9.0 Hz, 1 H), and 5.93 (s, 1 H).

*N*-(4-(2-Morpholinoethoxy)phenyl)-2-nitro-4-(trifluoromethyl)aniline (**13**). To a stirring solution of 4-((2-nitro-4-(trifluoromethyl)phenyl)amino)phenol (**12a**) (300 mg, 1.01 mmol, 1.0 equiv.) in ethanol (30 mL), 4-(2-chloroethyl)morpholine hydrochloride (223 mg, 1.21 mmol, 1.2 equiv.) and KOH (414 mg, 3.03 mmol, 3.0 equiv.) were added to reaction mixture while stirring. The batch was stirred at 100 °C overnight. The next day, solvent was removed under reduced pressure and crude product was purified with flash column chromatography using dichloromethane/methanol/NH_3_ = 20:1:0.01 as eluent. Yield: 29%; red solid (120 mg); R_f_ (dichloromethane/methanol = 20:1) = 0.55; ^1^H NMR (400 MHz, CDCl_3_) δ 9.61 (s, 1 H), 8.50 (d, *J* = 1.1 Hz, 1 H), 7.49 (dd, *J_1_* = 9.1 Hz, *J_2_* = 2.1 Hz, 1 H), 7.22–7.15 (m, 2 H), 7.05 (d, *J* = 9.1 Hz, 1 H), 7.02–6.96 (m, 2 H), 4.15 (t, *J* = 5.7 Hz, 2 H), 3.84–3.68 (m, 4 H), 2.84 (t, *J* = 5.7 Hz, 2 H), 2.66–2.53 (m, 4 H); ^13^C NMR (101 MHz, CDCl_3_) δ 157.90, 146.45, 131.84 (q, *J* = 3.0 Hz), 131.47, 130.32, 127.56, 124.88 (q, *J* = 4.5 Hz), 123.60 (q, *J* = 271.2 Hz), 118.96 (q, *J* = 34.4 Hz), 116.62, 116.03, 67.08, 66.34, 57.73, and 54.27; HRMS (ESI^+^) for C_19_H_21_F_3_N_3_O_4_ ([M+H]^+^) calculated 412.1479 found 412.1473; HPLC retention time: 4.247 min (99.01% at 254 nm).

*N*-(4-(Benzyloxy)phenyl)-2-nitro-4-(trifluoromethyl)aniline (**14**): To a stirring solution of 4-((2-nitro-4-(trifluoromethyl)phenyl)amino)phenol (**12a**) (200 mg, 0.8 mmol, 1.0 equiv.) in toluene (50 mL), NaHCO_3_ (134 mg, 1.6 mmol, 2.0 equiv) and 4-benzyloxy aniline were added (150 mg, 1.6 mmol, 2.0 equiv.). The batch was stirred at 100 °C for 24 h. Solvent was removed under reduced pressure and crude product was purified with Biotage Isolera One System reversed-phase chromatography (Biotage SNAP Cartridge KP-C18-HS 12 g column), MF: gradient water in H_2_O/acetonitrile. Yield: 31%; red solid (95 mg); ^1^H NMR (400 MHz, CDCl_3_) δ 9.61 (s, 1 H), 8.50 (s, 1 H), 7.53–7.32 (m, 6 H), 7.19 (d, *J* = 8.8 Hz, 2 H), 7.06 (d, *J* = 8.9 Hz, 3 H), 5.11 (s, 2 H); ^13^C NMR (101 MHz, CDCl_3_) δ ^13^C NMR (101 MHz, CDCl_3_) δ 157.92, 146.43, 136.65, 131.83 (q, *J* = 3.0 Hz), 131.47, 130.38, 128.84, 128.34, 127.63, 127.56, 124.87 (q, *J* = 4.3 Hz), 123.65 (q, *J* = 268.0 Hz), 118.95 (q, *J* = 34.4 Hz), 116.65, 116.33, and 70.50; HRMS (ESI^-^) for C_20_H_14_F_3_N_2_O_3_ ([M]^-^) calculated 387.0962 found 387.0964; HPLC retention time: 7.033 min (99.77% at 254 nm).

1-(Methylamino)-3-(4-((2-nitro-4-(trifluoromethyl)phenyl)amino)phenoxy)propan-2-ol (**15**): To a stirring solution of 2-nitro-*N*-(4-(oxiran-2-ylmethoxy)phenyl)-4-(trifluoromethyl)aniline (**4f**) (500 mg, 1.41 mmol, 1.0 equiv.) in acetonitrile (50 mL), MeNH_2_ (263 mg, 8.47 mmol, 6.0 equiv.) and K_2_CO_3_ (581 mg. 4.23 mmol, 3.0 equiv.) were added. The batch was stirred at 70 °C for 24 h. Solvent was removed under reduced pressure and crude product was purified with flash column chromatography using dichloromethane/methanol/NH_3_ = 20:1:0. as mobile phase. Yield: 22%; red solid (120 mg); ^1^H NMR (400 MHz, DMSO) δ 9.73 (s, 1 H), 8.35 (d, *J* = 1.3 Hz, 1 H), 7.72 (dd, *J_1_* = 9.2 Hz, *J_2_* = 2.2 Hz, 1 H), 7.35–7.20 (m, 2 H), 7.10–6.95 (m, 3 H), 3.99 (q, *J* = 7.0 Hz, 1 H), 3.91 (t, *J* = 5.9 Hz, 2 H), 2.69–2.51 (m, 2 H), 2.31 (s, 3 H). ^13^C NMR (101 MHz, CDCl_3_) δ 157.84, 146.41, 131.85 (q, *J* = 2.9 Hz), 131.46, 130.46, 127.56, 124.86 (q, *J* = 4.9 Hz), 123.54 (q, *J* = 270.0 Hz), 118.95 (q, *J* = 34.4 Hz), 116.61, 116.01, 73.35, 70.99, 68.10, 63.07, 53.98, 47.99, 36.48, and 31.09; HRMS (ESI^+^) for C_17_H_19_F_3_N_3_O_4_ ([M+H]^+^) calculated 386.1322 found 386.1318; HPLC retention time: 4.063 min (95.47% at 254 nm).

General procedure for opening of the epoxide with various aromatic amines to obtain **16a**–**d** (**16a** is given as example)

To a stirring solution of 2-nitro-*N*-(4-(oxiran-2-ylmethoxy)phenyl)-4-(trifluoromethyl)aniline (**4f**) (100 mg, 0.28 mmol, 1.0 equiv.) in acetonitrile (20 mL), 4-((3,4-dichlorophenoxy)methyl)piperidin-4-ol (78 mg, 0.28 mmol, 1.0 equiv) was added. The batch was stirred for 24 h at 70 °C. The next day, solvent was removed under reduced pressure and crude product was purified with flash column chromatography using dichloromethane/methanol = 100:1.

4-((3,4-Dichlorophenoxy)methyl)-1-(2-hydroxy-3-(4-((2-nitro-4-(trifluoromethyl)phenyl)amino)phenoxy)propyl)piperidin-4-ol (**16a**): Yield: 17%; red solid (30 mg); ^1^H NMR (400 MHz, CDCl_3_) δ 9.61 (s, 1 H), 8.49 (d, *J* = 1.0 Hz, 1 H), 7.49 (dd, *J_1_* = 9.1 Hz, *J_2_* = 2.0 Hz, 1 H), 7.34 (d, *J* = 8.9 Hz, 1 H), 7.19 (t, *J* = 5.9 Hz, 2 H), 7.11–6.95 (m, 4 H), 6.79 (dd, *J_1_* = 8.9 Hz, *J_2_* = 2.9 Hz, 1 H), 4.16 (td, *J_1_* = 9.3 Hz, *J_2_* = 4.8 Hz, 1 H), 4.09–3.97 (m, 2 H), 3.81 (s, 2 H), 2.87 (d, *J* = 11.3 Hz, 1 H), 2.83–2.57 (m, 4 H), 2.51 (td, *J_1_* = 11.3 Hz, *J_2_* = 3.0 Hz, 1 H), 1.92–1.66 (m, 4 H); ^13^C NMR (101 MHz, CDCl_3_) δ 157.90, 157.71, 146.39, 133.09, 131.82 (q, *J* = 3.0 Hz), 131.42, 130.90, 130.41, 127.51, 124.82 (q, *J* = 4.2 Hz), 124.69, 123.58 (q, *J* = 271.0 Hz), 118.90 (q, *J* = 34.4 Hz), 116.62, 116.60, 116.00, 114.67, 76.24, 70.79, 68.80, 65.64, 60.50, 50.41, 47.90, 34.21, and 34.00; HRMS (ESI^+^) for C_28_H_29_Cl_2_F_3_N_3_O_6_ ([M+H]^+^) calculated 630.1380 found 630.1365; HPLC retention time: 5.577 min (98.11% at 254 nm).

1-(2-Hydroxy-3-(4-((2-nitro-4-(trifluoromethyl)phenyl)amino)phenoxy)propyl)-4-((4-methoxyphenoxy)methyl)piperidin-4-ol (**16b**): Synthesised from 2-nitro-*N*-(4-(oxiran-2-ylmethoxy)phenyl)-4-(trifluoromethyl)aniline (4f) (100 mg, 0.282 mmol, 1.0 equiv.) and 4-((4-methoxyphenoxy)methyl)piperidin-4-ol (67 mg, 0.282 mmol, 1.0 equiv) in acetonitrile (20 mL). Purified with flash column chromatography using dichloromethane/methanol = 100:1. Yield: 14%; red solid (23 mg); ^1^H NMR (400 MHz, CDCl_3_) δ 9.60 (s, 1 H), 8.49 (d, *J* = 1.1 Hz, 1 H), 7.49 (dd, *J_1_* = 9.1 Hz, *J_2_* = 2.1 Hz, 1 H), 7.18 (t, *J* = 6.0 Hz, 2 H), 7.08–6.96 (m, 3 H), 6.92–6.77 (m, 4 H), 4.23–4.12 (m, 1 H), 4.10–3.97 (m, 2 H), 3.78 (s, 2 H), 3.77 (s, 3 H), 2.91–2.75 (m, 2 H), 2.74–2.57 (m, 3 H), 2.52 (td, *J_1_* = 11.3 Hz, *J_2_* = 2.9 Hz, 1 H), 1.88–1.67 (m, 4 H); ^13^C NMR (101 MHz, CDCl_3_) δ 157.92, 154.30, 152.87, 146.40, 131.80 (q, *J* = 2.9 Hz), 131.41, 130.37, 127.50, 124.81 (q, *J* = 4.2 Hz), 123.58 (q, *J* = 270.9 Hz), 118.87 (q, *J* = 34.3 Hz), 116.60, 116.00, 115.68, 114.81, 76.43, 70.82, 68.85, 65.60, 60.52, 55.83, 50.55, 48.06, 34.29, and 34.08; HRMS (ESI^+^) for C_29_H_33_F_3_N_3_O_7_ ([M+H]^+^) calculated 592.2265 found 592.2251; HPLC retention time: 5.073 min (97.04% at 254 nm).

3,4-Dichloro-*N*-(1-(2-hydroxy-3-(4-((2-nitro-4-(trifluoromethyl)phenyl)amino)phenoxy)propyl)piperidin-4-yl)benzamide (**16c**): Synthesised from 2-nitro-*N*-(4-(oxiran-2-ylmethoxy)phenyl)-4-(trifluoromethyl)aniline (4f) (100 mg, 0.282 mmol, 1.0 equiv.) and 3,4-dichloro-*N*-(piperidin-4-yl)benzamide (77 mg, 0.282 mmol, 1.0 equiv) in acetonitrile (20 mL). Purified with flash column chromatography using dichloromethane/methanol = 100:1. Yield: 17%; red solid (30 mg); ^1^H NMR (400 MHz, CDCl_3_) δ 9.60 (s, 1 H), 8.50 (d, *J* = 1.1 Hz, 1 H), 7.85 (d, *J* = 2.0 Hz, 1 H), 7.59 (dd, *J_1_* = 8.3 Hz, *J_2_* = 2.0 Hz, 1 H), 7.54–7.46 (m, 2 H), 7.24–7.16 (m, 2 H), 7.08–6.96 (m, 3 H), 6.07 (d, *J* = 7.7 Hz, 1 H), 4.13 (td, *J_1_* = 9.3 Hz, *J_2_* = 4.4 Hz, 1 H), 4.09–3.96 (m, 3 H), 3.07 (d, *J* = 11.6 Hz, 1 H), 2.90 (d, *J* = 11.7 Hz, 1 H), 2.70–2.48 (m, 3 H), 2.29–2.19 (m, 1 H), 2.09 (d, *J* = 11.2 Hz, 2 H), 1.72–1.50 (m, 2 H); ^13^C NMR (101 MHz, CDCl_3_) δ 164.84, 157.85, 146.40, 136.06, 134.53, 133.21, 131.85 (q, *J* = 2.9 Hz), 131.47, 130.77, 130.47, 129.25, 127.54, 126.21, 124.87 (q, *J* = 4.6 Hz), 123.59 (q, *J* = 271.1 Hz), 118.97 (q, *J* = 34.4 Hz), 116.60, 115.99, 70.67, 65.74, 60.36, 54.15, 51.34, 47.40, 32.63, and 32.33; HRMS (ESI^+^) for C_28_H_28_Cl_2_F_3_N_4_O_5_ ([M+H]^+^) calculated 627.1383 found 627.1369; HPLC retention time: 5.313 min (99.57% at 254 nm).

2-(3,4-Dichlorophenyl)-*N*-(1-(2-hydroxy-3-(4-((2-nitro-4-(trifluoromethyl)phenyl)amino)phenoxy)propyl)piperidin-4-yl)acetamide (**16d**): Synthesised from 2-nitro-*N*-(4-(oxiran-2-ylmethoxy)phenyl)-4-(trifluoromethyl)aniline (4f) (100 mg, 0.282 mmol, 1.0 equiv.) and 2-(3,4-dichlorophenyl)-*N*-(piperidin-4-yl)acetamide (81 mg, 0.282 mmol, 1.0 equiv) in acetonitrile (20 mL). Purified with flash column chromatography using dichloromethane/methanol = 100:1. Yield: 22%; red solid (40 mg); ^1^H NMR (400 MHz, DMSO) δ 9.74 (s, 1 H), 8.35 (d, *J* = 1.3 Hz, 1 H), 8.04 (d, *J* = 7.6 Hz, 1 H), 7.72 (dd, *J_1_* = 9.2 Hz, *J_2_* = 2.2 Hz, 1 H), 7.55 (d, *J* = 8.2 Hz, 1 H), 7.50 (d, *J* = 2.0 Hz, 1 H), 7.31–7.25 (m, 2 H), 7.22 (dd, *J_1_* = 8.3 Hz, *J_2_* = 2.0 Hz, 1 H), 7.13–6.98 (m, 3 H), 4.87 (d, *J* = 4.2 Hz, 1 H), 4.01 (dd, *J* = 9.2, 3.1 Hz, 1 H), 3.98–3.85 (m, 1 H), 3.56–3.44 (m, 1 H), 3.41 (s, 1 H), 2.91–2.77 (m, 1 H), 2.40 (ddd, *J* = 33.4, 12.7, 5.9 Hz, 1 H), 2.08 (dd, *J* = 19.9, 9.4 Hz, 1 H), 1.76–1.63 (m, 1 H), 1.39 (dd, *J* = 21.8, 10.8 Hz, 1 H); ^13^C NMR (101 MHz, CDCl_3_) δ 169.14, 157.87, 146.40, 135.13, 132.98, 131.84 (q, *J* = 2.9 Hz), 131.68, 131.45, 131.31, 130.91, 130.44, 128.74, 127.54, 124.86 (q, *J* = 4.7 Hz), 123.55 (q, *J* = 277.7 Hz), 118.96 (q, *J* = 34.5 Hz), 116.60, 116.00, 70.69, 65.69, 60.32, 54.02, 51.25, 46.90, 42.85, 32.50, and 32.21; HRMS (ESI^+^) for C_29_H_29_Cl_2_F_3_N_4_O_5_ ([M+H]^+^) calculated 641.1540 found 641.1524; HPLC retention time: 5.280 min (97.83% at 254 nm).

General procedure for synthesis of (diethylamino)ethoxy compounds **17a**–**c** (**17a** is given as example)

To a stirring solution of 4-((2-nitro-4-(trifluoromethyl)phenyl)amino)phenol (**12a**) (300 mg, 1.00 mmol, 1.0 equiv.) in ethanol (30 mL), 2-chloro-*N*,*N*-diethylethan-1-amine hydrochloride (206 mg, 1.2 mmol, 1.2 equiv) and KOH (414 mg, 7.38 mmol, 7.3 equiv) were added. Batch was stirred overnight at 100 °C. KOH was filtered off and solvent was removed under reduced pressure. Crude product was purified with flash column chromatography using dichloromethane/methanol/NH_3_ = 10:1:0.01 and dry sample loading.

*N*-(4-(2-(Diethylamino)ethoxy)phenyl)-2-nitro-4-(trifluoromethyl)aniline (**17a**): Yield: 38%; red solid (150 mg); ^1^H NMR (400 MHz, CDCl_3_) δ 9.60 (s, 1 H), 8.49 (d, *J* =, 1.3 Hz, 1 H), 7.48 (dd, *J*_1_ = 9.1 Hz, *J*_2_ = 2.1 Hz, 1 H), 7.20–7.14 (m, 2 H), 7.04 (d, *J* = 9.1 Hz, 1 H), 7.01–6.95 (m, 2 H), 4.07 (t, *J* = 6.3 Hz, 2 H), 2.90 (t, *J* = 6.3 Hz, 2 H), 2.66 (q, *J* = 7.1 Hz, 4 H), 1.09 (t, *J* = 7.1 Hz, 6 H); ^13^C NMR (101 MHz, CDCl_3_) δ 158.11, 146.52, 131.82 (q, *J* = 3.0 Hz), 131.41, 130.06, 127.54, 124.86 (q, J = 4.3 Hz), 123.62 (q, *J* = 270.8 Hz), 118.87 (q, *J* = 34.4 Hz), 116.65, 115.96, 67.20, 51.87, 48.05, and 12.01; HRMS (ESI^+^) for C_19_H_22_F_3_N_3_O_3_ ([M+H]^+^) calculated 398.1686 found 398.1682; HPLC retention time: 4.323 min (98.90% at 254 nm).

3,5-Dibromo-4-(2-(diethylamino)ethoxy)-*N*-(2-nitro-4-(trifluoromethyl)phenyl)aniline (**17b**): Synthesised from 2,6-dibromo-4-((2-nitro-4-(trifluoromethyl)phenyl)amino)phenol (**12c**) (300 mg, 0.66 mmol, 1.0 equiv), 2-chloro-*N*,*N*-diethylethan-1-amine hydrochloride (227 mg, 1.32 mmol, 2.0 equiv), and K_2_CO_3_ (273 mg, 2.0 mmol, 3.0 equiv) at 90 °C in acetonitrile (40 mL) overnight. Purified with flash column chromatography using ethyl acetate/hexane = 1:1 as eluent and dry sample loading. Yield: 15%; orange solid (55 mg); ^1^H NMR (400 MHz, CDCl_3_) δ 9.53 (s, 1 H), 8.49 (d, *J* = 1.4 Hz, 1 H), 7.63 (dd, *J*_1_ = 9.0 Hz, *J*_2_ = 2.0 Hz, 1 H), 7.48 (s, 2 H), 7.21 (d, *J* = 9.0 Hz, 1 H), 4.37 (t, *J* = 5.0 Hz, 2 H), 3.54 (t, *J* = 9.2 Hz, 2 H), 3.29 (q, J = 6.8 Hz, 4 H), 1.36 (t, *J* = 7.2 Hz, 6 H); ^13^C NMR (101 MHz, CDCl_3_) δ 151.00, 144.11, 136.23, 132.75, 132.30 (q, *J* = 3.1 Hz), 129.08, 124.88 (q, *J* = 4.1 Hz), 123.25 (q, *J* = 271.8 Hz), 120.85 (q, *J* = 34.6 Hz), 119.20, 116.58, 68.46, 50.73, 47.52, and 9.53; HRMS (ESI^+^) for C_19_H_20_Br_2_F_3_N_3_O_3_ ([M+H]^+^) calculated 553.9896 found 553.9894; HPLC retention time: 4.717 min (95.89% at 254 nm).

3,5-Dichloro-4-(2-(diethylamino)ethoxy)-*N*-(2-nitro-4-(trifluoromethyl)phenyl)aniline (**17c**): Synthesised from 2,6-dichloro-4-((2-nitro-4-(trifluoromethyl)phenyl)amino)phenol (**12d**) (240 mg, 0.65 mmol, 1.0 equiv), 2-chloro-*N*,*N*-diethylethan-1-amine hydrochloride (190 mg,1.3 mmol, 2.0 equiv), and K_2_CO_3_ (271 mg, 2.0 mmol, 3.0 equiv.) at 90 °C in acetonitrile (40 mL) overnight. Purified with flash column chromatography using ethyl acetate/hexane = 1:1 as eluent and dry sample loading. Yield: 15%; orange solid (40 mg); ^1^H NMR (400 MHz, CDCl_3_) δ 9.53 (s, 1 H), 8.51 (d, *J* = 1.3 Hz, 1 H), 7.62 (dd, *J*_1_ = 9.0 Hz, *J*_2_ = 2.1 Hz, 1 H), 7.26 (s, 2 H), 7.21 (d, *J* = 9.0 Hz, 1 H), 4.14 (t, *J* = 6.5 Hz, 2 H), 3.00 (t, *J* = 6.5 Hz, 2 H), 2.69 (q, *J* = 7.1 Hz, 4 H), 1.09 (t, *J* = 7.1 Hz, 6 H); ^13^C NMR (101 MHz, CDCl_3_) δ 150.59, 144.51, 134.30, 132.56, 132.27 (q, *J* = 3.0 Hz), 130.74, 125.63, 124.95 (q, *J* = 4.1 Hz), 123.35 (q, *J* = 271.5 Hz), 120.58 (q, *J* = 34.6 Hz), 116.63, 72.00, 52.37, 47.64, and 11.87; HRMS (ESI^+^) for C_19_H_20_Cl_2_F_3_N_3_O_3_ ([M+H]^+^) calculated 466.0903 found 466.0907; HPLC retention time: 4.603 min (95.04% at 254 nm).

General procedure for synthesis of (dimethylamino)ethoxy compounds **18a**–**c** (1**8a** is given as an example)

To a stirring solution of 4-((2-nitro-4-(trifluoromethyl)phenyl)amino)phenol (**12a**) (300 mg, 1.01 mmol, 1.0 equiv.) in ethanol (30 mL), 2-chloro-*N*,*N*-dimethylethan-1-amine hydrochloride (174 mg, 1.21 mmol) and KOH (414 mg, 7.38 mmol, 7.3 equiv.) were added. Batch was stirred overnight at 100 °C. Solvent was removed under reduced pressure and product was purified with flash column chromatography using dichloromethane/methanol/NH_3_ = 10:1:0.01 and dry sample loading.

*N*-(4-(2-(Dimethylamino)ethoxy)phenyl)-2-nitro-4-(trifluoromethyl)aniline (**18a**): Yield: 27%; red solid (100 mg); R_f_ (dichloromethane/methanol = 20:1) = 0.05; ^1^H NMR (400 MHz, CDCl_3_) δ 9.61 (s, 1 H), 8.50 (d, *J* = 1.1 Hz, 1 H), 7.49 (dd, *J_1_* = 9.1 Hz, *J_2_* = 2.1 Hz, 1 H), 7.22–7.14 (m, 2 H), 7.08–6.96 (m, 3 H), 4.11 (t, *J* = 5.7 Hz, 2 H), 2.78 (t, *J* = 5.7 Hz, 2 H), 2.38 (s, 6 H); ^13^C NMR (101 MHz, CDCl_3_) δ 158.01, 146.48, 131.83 (q, *J* = 3.1 Hz), 131.42, 130.19, 127.52, 124.85 (q, *J* = 4.3 Hz), 123.61 (q, *J* = 271.0 Hz), 118.89 (q, *J* = 34.4 Hz), 116.65, 116.00, 66.46, 58.37, and 46.05; HRMS (ESI^+^) for C_17_H_19_F_3_N_3_O_3_ ([M+H]^+^) calculated 370.1373 found 370.1368; HPLC retention time: 4.200 min (97.85% at 254 nm).

3,5-Dibromo-4-(2-(dimethylamino)ethoxy)-N-(2-nitro-4-(trifluoromethyl)phenyl)aniline (**18b**): Synthesised from 2,6-dibromo-4-((2-nitro-4-(trifluoromethyl)phenyl)amino)phenol (**12c**) (300 mg, 0.66 mmol, 1.0 equiv), 2-chloro-*N*,*N*-dimethylethan-1-amine hydrochloride (112 mg, 0.8 mmol, 1.2 equiv) and K_2_CO_3_ (273 mg, 2.0 mmol, 3.0 equiv) in acetonitrile (40 mL) at 90 °C overnight. Purified with flash column chromatography using flash column chromatography using ethyl acetate/hexane = 1:1 as eluent and dry sample loading. Yield: 17%; orange solid (60 mg); ^1^H NMR (400 MHz, CDCl_3_) δ 9.54 (s, 1 H), 8.51 (d, *J* = 1.3 Hz, 1 H), 7.62 (dd, *J_1_* = 9.0 Hz, *J_2_* = 2.0 Hz, 1 H), 7.47 (s, 2 H), 7.20 (d, *J* = 9.0 Hz, 1 H), 4.14 (t, *J* = 5.9 Hz, 2 H), 2.85 (t, *J* = 5.9 Hz, 2 H), 2.39 (s, 6 H); ^13^C NMR (101 MHz, CDCl_3_) δ 152.41, 144.49, 135.27, 132.54, 132.27 (q, *J* = 3.1 Hz), 129.30, 124.93 (q, *J* = 4.1 Hz), 123.34 (d, *J* = 271.2 Hz), 120.59 (q, *J* = 34.6 Hz), 119.31, 116.61, 71.35, 58.87, and 46.00; HRMS (ESI^+^) for C_17_H_16_Br_2_F_3_N_3_O_3_ ([M+H]^+^) calculated 525.9583 found 525.9580; HPLC retention time: 4.417 min (99.88% at 254 nm).

3,5-Dichloro-4-(2-(dimethylamino)ethoxy)-N-(2-nitro-4-(trifluoromethyl)phenyl)aniline (**18c**): Synthesized from 2,6-dichloro-4-((2-nitro-4-(trifluoromethyl)phenyl)amino)phenol (**12d**) (300 mg, 0.82 mmol, 1.0 equiv), 2-chloro-*N*,*N*-dimethylethan-1-amine hydrochloride (235 mg, 1.6 mmol, 2.0 equiv), and K_2_CO_3_ (338 mg, 2.45 mmol, 3.0 equiv) in acetonitrile (40 mL) at 90 °C overnight. Purified with flash column chromatography using ethyl acetate/hexane = 1:1 as eluent and dry sample loading. Yield: 16%; orange solid (57 mg); ^1^H NMR (400 MHz, CDCl_3_) δ 9.53 (s, 1 H), 8.51 (d, *J* = 1.4 Hz, 1 H), 7.62 (dd, *J_1_* = 9.0 Hz, *J_2_* = 2.1 Hz, 1 H), 7.26 (s, 2 H), 7.21 (d, *J* = 9.0 Hz, 1 H), 4.15 (t, *J* = 5.8 Hz, 2 H), 2.82 (t, *J* = 5.8 Hz, 2 H), 2.38 (s, 6 H); ^13^C NMR (101 MHz, CDCl_3_) δ 150.46, 144.45, 134.38, 132.55, 132.24 (q, *J* = 3.1 Hz), 130.76, 125.57, 124.92 (q, *J* = 4.2 Hz), 123.33 (q, *J* = 271.3 Hz), 120.56 (q, *J* = 34.6 Hz), 116.63, 71.51, 58.89, and 45.93; HRMS (ESI^+^) for C_17_H_16_Cl_2_F_3_N_3_O_3_ ([M+H]^+^) calculated 438.0594 found 438.0591; HPLC retention time: 4.307 min (98.68% at 254 nm).

2-Nitro-*N*-(4-(2-(pyrrolidin-1-yl)ethoxy)phenyl)-4-(trifluoromethyl)aniline (**19**): To a stirring solution of 4-((2-nitro-4-(trifluoromethyl)phenyl)amino)phenol (**12a**) (300 mg, 1.00 mmol, 1.0 equiv.) in acetonitrile (40 mL), 1-(2-chloroethyl)pyrrolidine × HCl (204 mg, 1.2 mmol, 1.2 equiv) and K_2_CO_3_ (414 mg, 3.0 mmol, 3.0 equiv) were added. Batch was stirred overnight at 90 °C. K_2_CO_3_ was filtered off and solvent was removed under reduced pressure. Crude product was purified with flash column chromatography using dichloromethane/methanol = 40:1 and dry sample loading. Yield: 30%; red solid (120 mg); ^1^H NMR (400 MHz, CDCl_3_) δ 9.60 (s, 1 H), 8.49 (d, *J* = 1.6 Hz, 1 H), 7.48 (dd, *J*_1_ = 9.1 Hz, *J*_2_ = 2.1 Hz, 1 H), 7.21–7.13 (m, 2 H), 7.04 (d, *J* = 9.1 Hz, 1 H), 7.02–6.93 (m, 2 H), 4.15 (t, *J* = 5.9 Hz, 2 H), 2.95 (t, *J* = 5.9 Hz, 2 H), 2.67 (t, *J* = 6.6 Hz, 4 H), 1.84 (h, *J* = 3.2 Hz, 4 H); ^13^C NMR (101 MHz, CDCl_3_) δ 158.00, 146.47, 131.81 (q, *J* = 3.1 Hz), 131.40, 130.14, 127.52, 124.93 (d, *J* = 271.6 Hz), 124.83 (q, *J* = 4.3 Hz), 118.86 (q, *J* = 34.4 Hz), 116.64, 116.00, 67.52, 55.14, 54.90, and 23.63; HRMS (ESI^+^) for C_19_H_20_F_3_N_3_O_3_ ([M+H]^+^) calculated 396.15295 found 396.15260; HPLC retention time: 4.190 min (98.91% at 254 nm).

2-Nitro-*N*-(4-(2-(piperidin-1-yl)ethoxy)phenyl)-4-(trifluoromethyl)aniline (**20**): To a stirring solution of 4-((2-nitro-4-(trifluoromethyl)phenyl)amino)phenol (**12a**) (300 mg, 1.00 mmol, 1.0 equiv.) in acetonitrile (40 mL), 1-(2-chloroethyl)piperidine × HCl (370 mg, 2.0 mmol, 2.0 equiv) and K_2_CO_3_ (414 mg, 3.0 mmol, 3.0 equiv) were added. Batch was stirred overnight at 90 °C. K_2_CO_3_ was filtered off and solvent was removed under reduced pressure. Crude product was purified with flash column chromatography using ethyl acetate/hexane = 1:1 as eluent and dry sample loading. Yield: 27%; red solid (109 mg); ^1^H NMR (400 MHz, CDCl_3_) δ 9.60 (s, 1 H), 8.49 (d, *J* = *1*.3 Hz, 1 H), 7.48 (dd, *J_1_* = 9.1 Hz, *J_2_* =2.2 Hz, 1 H), 7.22–7.13 (m, 2 H), 7.04 (d, *J* = 9.1 Hz, 1 H), 7.01–6.94 (m, 2 H), 4.13 (t, *J* = 6.0 Hz, 2 H), 2.79 (t, *J* = 6.0 Hz, 2 H), 2.52 (s, 4 H), 1.62 (p, *J* = 5.6 Hz, 4 H), 1.46 (q, *J* = 5.9 Hz, 2 H); ^13^C NMR (101 MHz, CDCl_3_) δ 158.04, 146.49, 131.81 (q, *J* = 3.1 Hz), 131.41, 130.11, 127.52, 124.85 (q, *J* = 4.3 Hz), 123.61 (q, *J* = 271.0 Hz), 118.88 (q, *J* = 34.4 Hz), 116.65, 116.02, 66.53, 58.03, 55.26, 26.09, and 24.32; HRMS (ESI^+^) for C_20_H_22_F_3_N_3_O_3_ ([M+H]^+^) calculated 410.1686 found 410.1682; HPLC retention time: 4.283 min (99.95% at 254 nm).

*N*-(4-(2-(Azepan-1-yl)ethoxy)phenyl)-2-nitro-4-(trifluoromethyl)aniline (**21**): To a stirring solution of 4-((2-nitro-4-(trifluoromethyl)phenyl)amino)phenol (**12a**) (300 mg, 1.00 mmol, 1.0 equiv.) in acetonitrile (40 mL), 1-(2-chloroethyl)azepane hydrochloride × HCl (400 mg, 2.0 mmol, 2.0 equiv) and K_2_CO_3_ (414 mg, 3.0 mmol, 3.0 equiv) were added. Batch was stirred overnight at 90 °C. K_2_CO_3_ was filtered off and solvent was removed under reduced pressure. Crude product was purified with flash column chromatography using ethyl acetate/hexane = 1:1 as eluent and dry sample loading. Yield: 26%; red solid (112 mg); ^1^H NMR (400 MHz, CDCl_3_) δ 9.60 (s, 1 H), 8.49 (d, *J* = 1.4 Hz, 1 H), 7.48 (dd, *J_1_* = 9.1 Hz, *J_2_* = 2.1 Hz, 1 H), 7.21–7.15 (m, 2 H), 7.04 (d, *J* = 9.1 Hz, 1 H), 7.01–6.96 (m, 2 H), 4.09 (t, *J* = 6.2 Hz, 2 H), 2.98 (t, *J* = 6.2 Hz, 2 H), 2.84–2.74 (m, 4 H), 1.75–1.57 (m, 8 H); ^13^C NMR (101 MHz, CDCl_3_) δ 158.14, 146.51, 131.81 (q, *J* = 3.0 Hz), 131.41, 130.05, 127.52, 124.85 (q, *J* = 4.3 Hz), 123.61 (q, *J* = 271.0 Hz), 118.87 (q, *J* = 34.4 Hz), 116.65, 116.02, 67.09, 56.41, 56.05, 28.10, and 27.21; HRMS (ESI^+^) for C_21_H_24_F_3_N_3_O_3_ ([M+H]^+^) calculated 424.1843 found 424.1837; HPLC retention time: 4.450 min (99.58% at 254 nm).
