# Supplementary material for: New Diarylamine KV10.1 Inhibitors and Their Anticancer Potential
Source: Pharmaceutics. 2022 Sep 17;14(9):1963. doi: 10.3390/pharmaceutics14091963 (PMC9501377; doi:10.3390/pharmaceutics14091963)

Supplementary Materials: New Diarylamine Kv10.1 Inhibitors and Their Anticancer Potential

Špela Gubič, Žan Toplak , Xiaoyi Shi, Jaka Dernovšek, Louise Antonia Hendrickx, Ernesto Lopes Pinheiro-Junior, Steve Peigneur, Jan Tytgat, Luis A. Pardo, Lucija Peterlin Mašič and Tihomir Tomašič

1. Supplementary tables

Table S1. Kv1.3 inhibitory potencies on *Xenopus laevis* oocytes of newly designed and synthesised ZVS-08 (**1**) analogues (Strategy I), voltage-clamped to determine the percentage of inhibition at 10 μM.

**Compound ID**	Strategy I<br>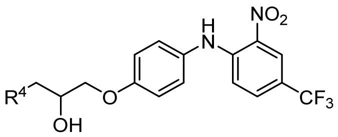<br>**R[4]**	**% of Kv10.1 inhibition at 10 μM (*Xenopus laevis* oocytes)**
**16d**	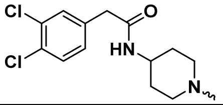	0%

Table S2. Kv1.3 inhibitory potencies on *Xenopus laevis* oocytes of newly designed and synthesised ZVS-08 (**1**) analogues (Strategy II), voltage-clamped to determine the percentage of inhibition at 10 μM on Kv10.1 and hERG.

**Compound ID**	**R^1**	**R^2**	**R^3**	**R^4**	**% of Kv10.1 inhibition at 10 μM (*Xenopus laevis* oocytes)t**	**% of hERG inhibition at 10 μM (*Xenopus laevis* oocytes)t**
**5a**	O	$COOCH_3$	H	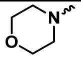	-4.54 % ± 2.92	14.99% ± 0.36
**5b**	O	NO_2	CF_3	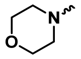	24.89% ± 5.54	11.46% ± 2.33
**5c**	NHCO	NO_2	CF_3	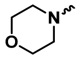	-0.66% ± 0.78	21.47% ± 6.02
**5d**	NH	$COOCH_3$	H	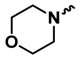	-2.11% ± 1.50	20.43% ± 3.19
**5e**	NH	$COOCH_3$	CF_3	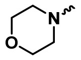	-2.17% ± 3.06	37.32% ± 13.11
**5f**	NH	NO_2	NO_2	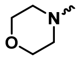	41.31% ± 2.54	65.85% ± 1.01
**6a**	O	NO_2	CF_3	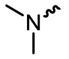	23.66% ± 1.76	69.14% ± 1.87
**6b**	NHCO	NO_2	CF_3	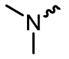	-0.41% ± 6.31	9.83% ± 5.36
**6c**	NH	$COOCH_3$	CF_3	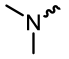	9.91% ± 1.77	63.66% ± 1.72
**6d**	NH	NO_2	NO_2	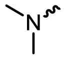	44.15% ± 5.59	74.33% ± 5.28
**10a**	NH	COOH	H	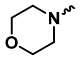	12.97% ± 1.20	3.42% ± 5.17
**10b**	NH	COOH	CF_3	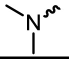	15.66% ± 6.79	14.71% ± 4.82

Table S3. Kv1.3 inhibitory potencies on *Xenopus laevis* oocytes of newly designed and synthesised ZVS-08 (**1**) analogues (Strategy II), voltage-clamped to determine the percentage of inhibition at 10 μM on Kv10.1 and hERG.

Compound ID	Structure	% of Kv10.1 inhibition at 10 μM (*Xenopus laevis* oocytes)t	% of hERG inhibition at 10 μM (*Xenopus laevis* oocytes)t
**14**	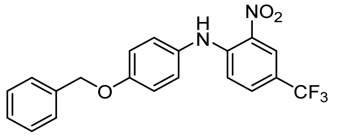	-1.18% ± 0.71	4.23% ± 4.72
**15**	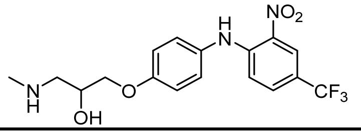	39.92% ± 0.98	58.58% ± 6.58

Table S4. Kv1.3 inhibitory potencies on *Xenopus laevis* oocytes of newly designed and synthesised ZVS-08 (**1**) analogues (Strategy IV), voltage-clamped to determine the percentage of inhibition at 10 μM on Kv10.1 and hERG.

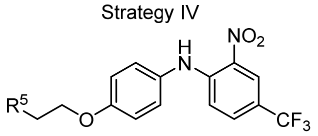

Compound ID	R_5	% of Kv10.1 inhibition at 10 μM (*Xenopus laevis* oocytes)t	% of hERG inhibition at 10 μM (*Xenopus laevis* oocytes)t
**13**	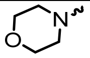	22.58% ± 4.57	34.69% ± 7.67
**17a**	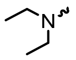	63.50% ± 0.33<br>IC_{50} = **3.58 ± 1.50** μM	64.49% ± 6.43<br>IC_{50} = **2.01 ± 0.65** μM
**18a**	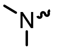	41.68% ± 2.26<br>IC_{50} = **11.63 ± 0.35** μM	65.16% ± 8.48<br>IC_{50} = **7.56 ± 0.41** μM
**19**	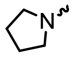	55.66% ± 1.95	65.91% ± 7.63
**20**	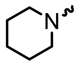	36.28%± 9.00<br>IC_{50} = **12.93 ± 0.04** μM	75.61% ± 1.77<br>IC_{50} = **0.59 ± 0.01** μM
**21**	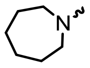	43.35% ± 1.33<br>IC_{50} = **13.34 ± 1.89** μM	77.45 ± 5.71<br>IC_{50} = **1.34 ± 0.32** μM

Table S5. Kv1.3 inhibitory potencies on *Xenopus laevis* oocytes of newly designed and synthesised ZVS-08 (**1**) analogues (Strategy V), voltage-clamped to determine the percentage of inhibition at 10 μM on Kv10.1 and hERG.

Strategy V

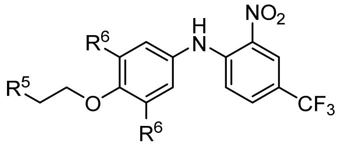

Compound ID	R_5	R_6	% of Kv10.1 inhibition at 10 μM (*Xenopus laevis* oocytes)t	% of hERG inhibition at 10 μM (*Xenopus laevis* oocytes)t
**17b**	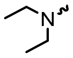	Br	19.52% ± 4.90	60.13% ± 4.79
**17c**	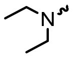	Cl	9.21% ± 2.42	54.69% ± 7.36
**18b**	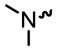	Br	25.99% ± 4.04	66.82% ± 11.56
**18c**	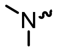	Cl	58.24% ± 2.23<br>IC_{50} = **19.05 ± 2.85** μM	76.76% ± 4.16<br>IC_{50} = **0.66 ± 0.27** μM

2. Representative ^{1}H NMR, ^{13}C NMR spectra and HRMS data

Methyl 2-(4-hydroxyphenoxy)benzoate (3a)

^{1}H NMR (400 MHz, $CDCl_3$) δ 7.87 (dd, J_1 = 7.8 Hz, J_2 = 1.8 Hz, 1H), 7.44 – 7.37 (m, 1H), 7.11 (td, J_1 = 7.7 Hz, J_2 = 1.0 Hz, 1H), 6.91 – 6.85 (m, 3H), 6.83 – 6.78 (m, 2H), 4.90 (brs, 1H), 3.86 (s, 3H).

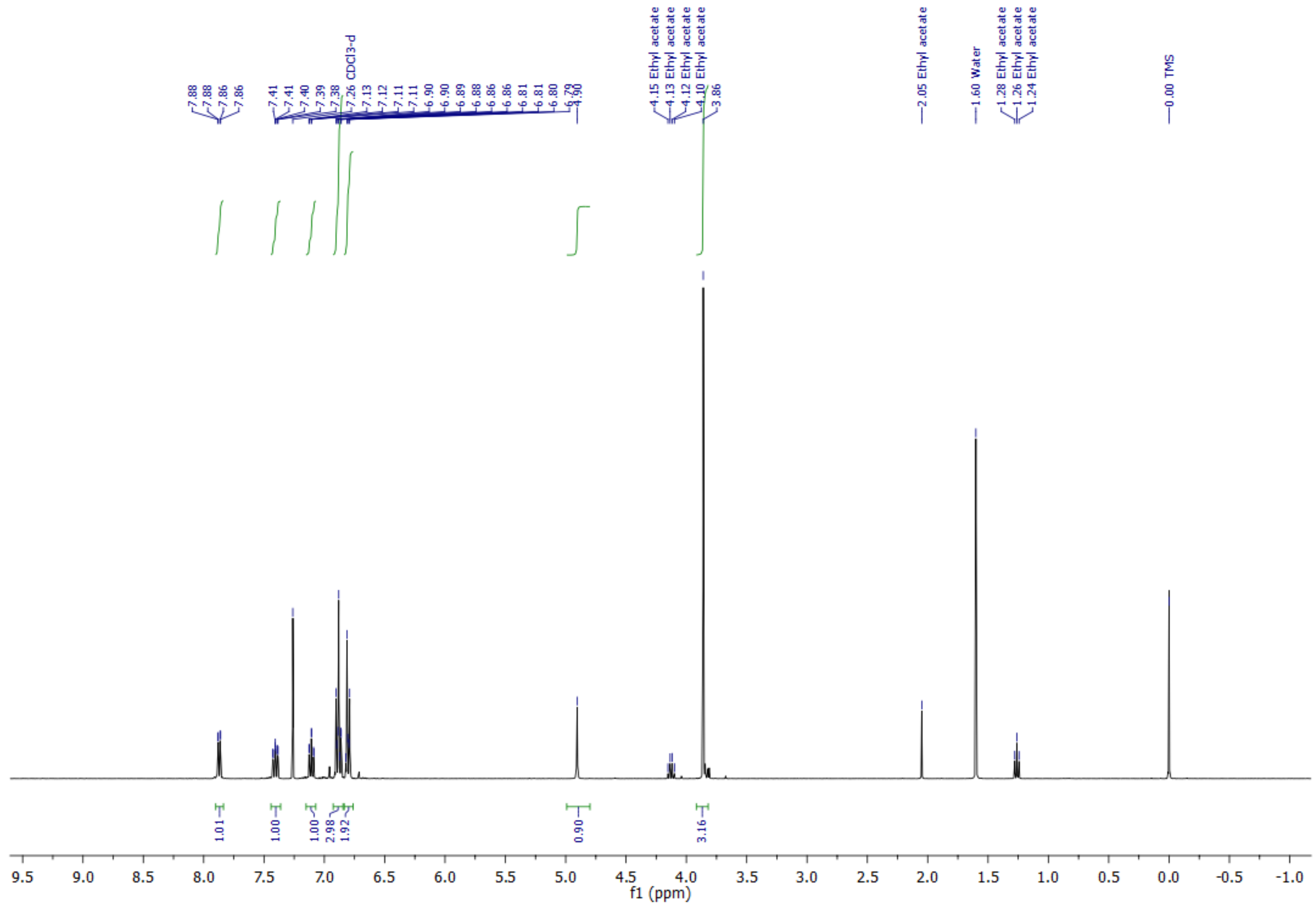

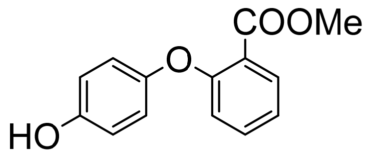

4-(2-Nitro-4-(trifluoromethyl)phenoxy)phenol (3b)

^{1}H NMR (400 MHz, $CDCl_3$) δ 8.21 (d, *J* = 2.0 Hz, 1H), 7.73 – 7.62 (m, 1H), 7.03 – 6.96 (m, 3H), 6.95 – 6.87 (m, 2H), 6.22 (brs, 1H)

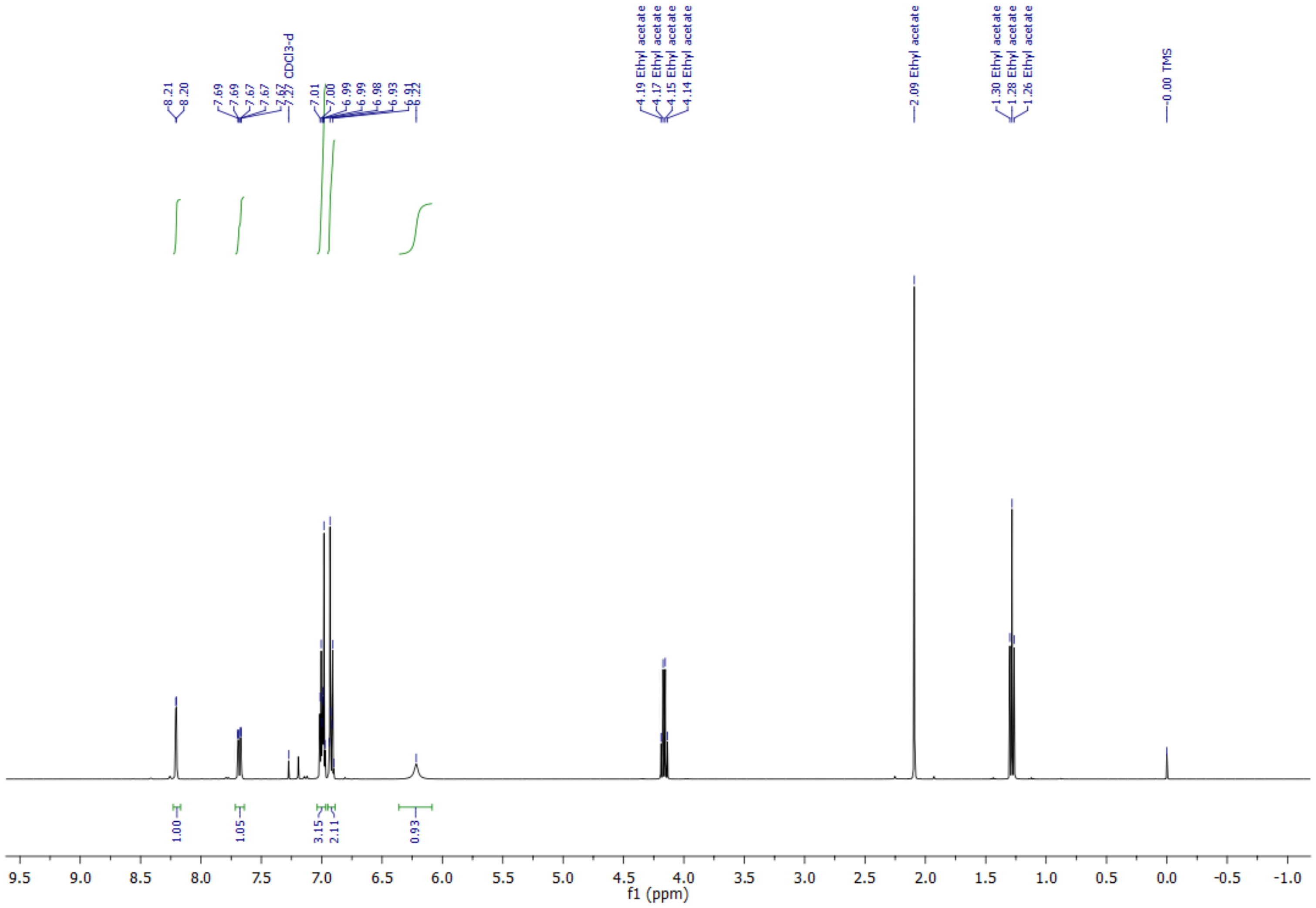

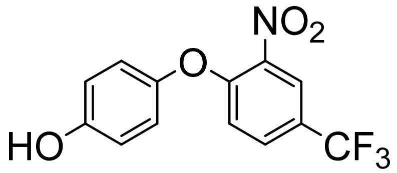

Methyl 2-(4-(oxiran-2-ylmethoxy)phenoxy)benzoate (4a)

^{1}H NMR (400 MHz, $CDCl_3$) δ 7.88 (dd, J_1 = 7.8 Hz, J_2 = 1.8 Hz, 1H), 7.41 (ddd, J_1 = 8.3 Hz, J_2 = 7.4 Hz, J_3 = 1.8 Hz, 1H), 7.12 (td, J_1 = 7.7 Hz, J_2 = 1.1 Hz, 1H), 6.98 – 6.85 (m, 5H), 4.21 (dd, J_1 = 11.0 Hz, J_2 = 3.1 Hz, 1H), 3.93 (dd, J_1 = 11.0 Hz, J_2 = 5.7 Hz, 1H), 3.85 (s, 3H), 3.35 (ddt, J_1 = 5.7 Hz, J_2 = 4.1 Hz, J_3 = 3.0 Hz, 1H), 2.91 (dd, J_1 = 4.9 Hz, J_2 = 4.2 Hz, 1H), 2.76 (dd, J_1 = 4.9 Hz, J_2 = 2.7 Hz, 1H).

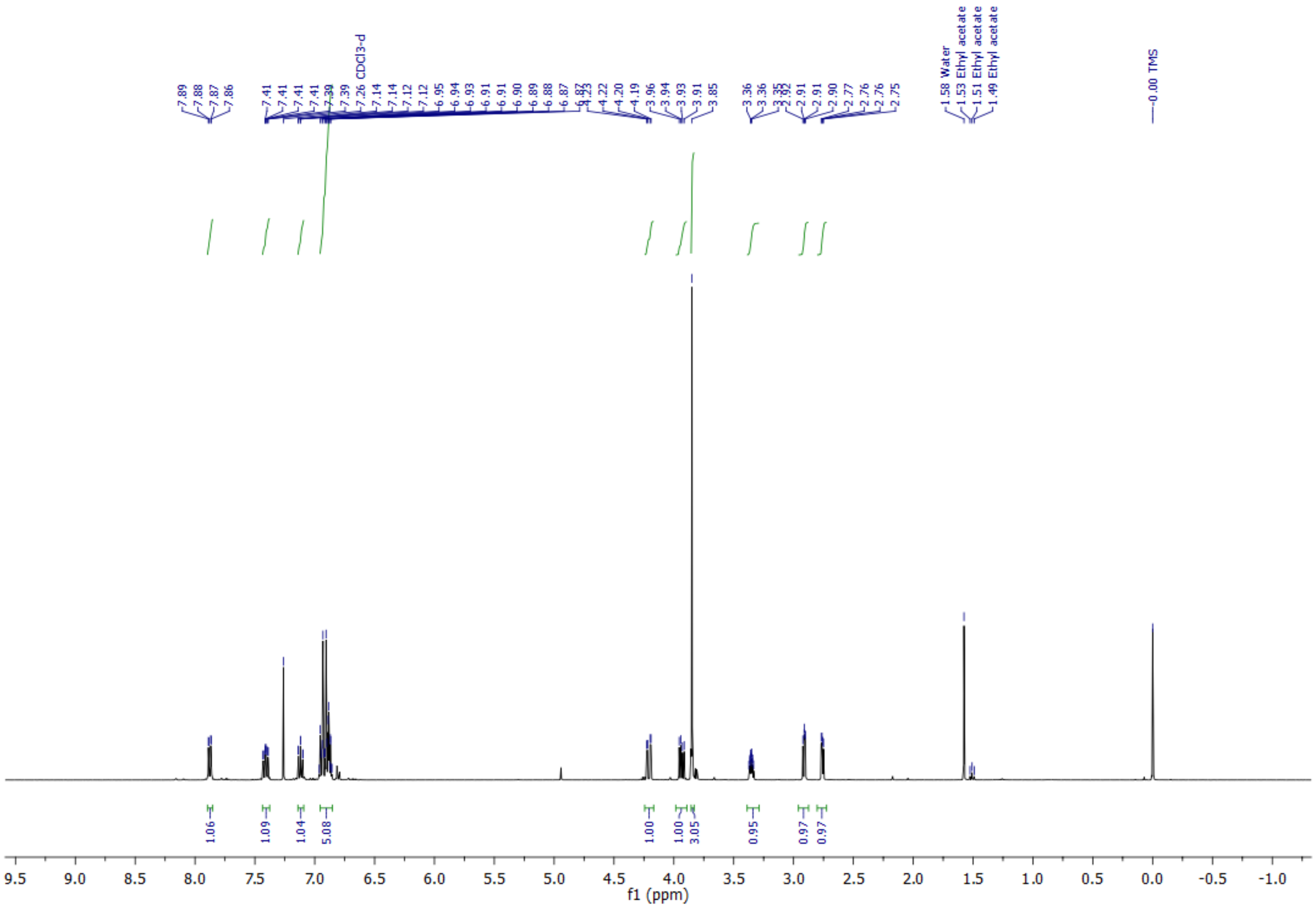

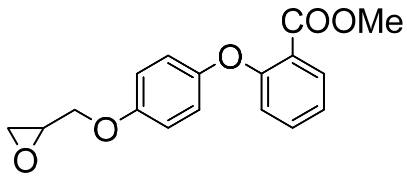

2-((4-(2-Nitro-4-(trifluoromethyl)phenoxy)phenoxy)methyl)oxirane (4b)

^{1}H NMR (400 MHz, DMSO) δ 8.44 (d, *J* = 2.0 Hz, 1H), 8.02 – 7.92 (m, 1H), 7.24 – 7.15 (m, 2H), 7.13 – 7.03 (m, 3H), 4.36 (dd, J_1 = 11.4 Hz, J_2 = 2.6 Hz, 1H), 3.86 (dd, J_1 = 11.4 Hz, J_2 = 6.6 Hz, 1H), 3.37 – 3.33 (m, 1H), 2.86 (dd, J_1 = 5.0 Hz, J_2 = 4.3 Hz, 1H), 2.72 (dd, J_1 = 5.1 Hz, J_2 = 2.7 Hz, 1H).

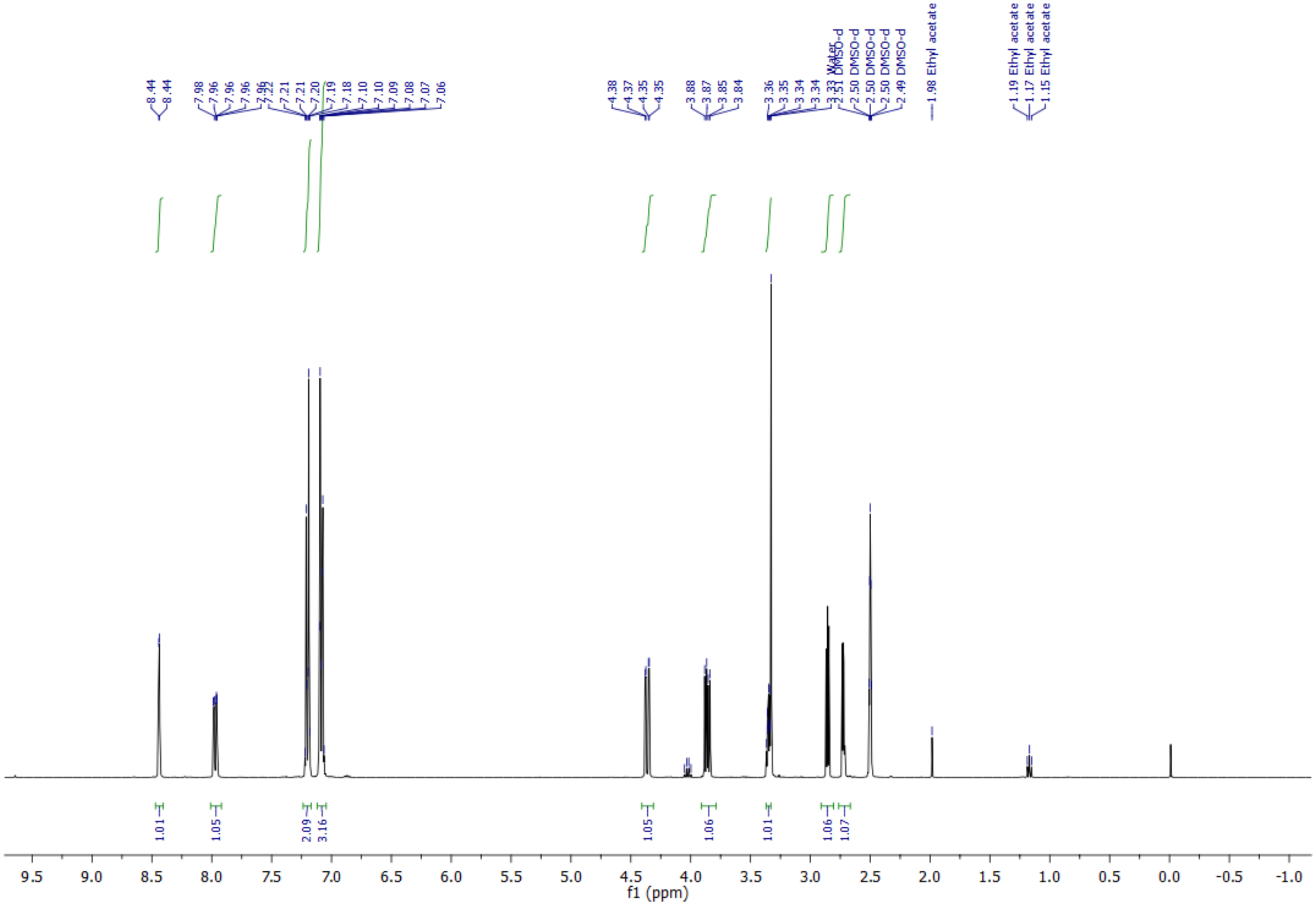

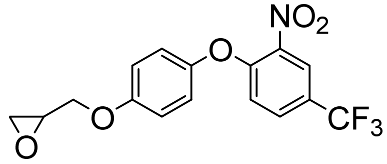

2-Nitro-*N*-(4-(oxiran-2-ylmethoxy)phenyl)-4-(trifluoromethyl)benzamide (4c)

^{1}H NMR (400 MHz, DMSO) δ 10.66 (s, 1H), 8.50 (s, 1H), 8.28 (dd, J_1 = 8.0 Hz, J_2 = 1.1 Hz, 1H), 8.03 (d, J = 7.9 Hz, 1H), 7.65 – 7.51 (m, 2H), 7.04 – 6.92 (m, 2H), 4.32 (dd, J_1 = 11.4 Hz, J_2 = 2.7 Hz, 1H), 3.82 (dd, J_1 = 11.4 Hz, J_2 = 6.5 Hz, 1H), 3.36 – 3.33 (m, 1H), 2.87 – 2.81 (m, 1H), 2.71 (dd, J_1 = 5.1 Hz, J_2 = 2.7 Hz, 1H).

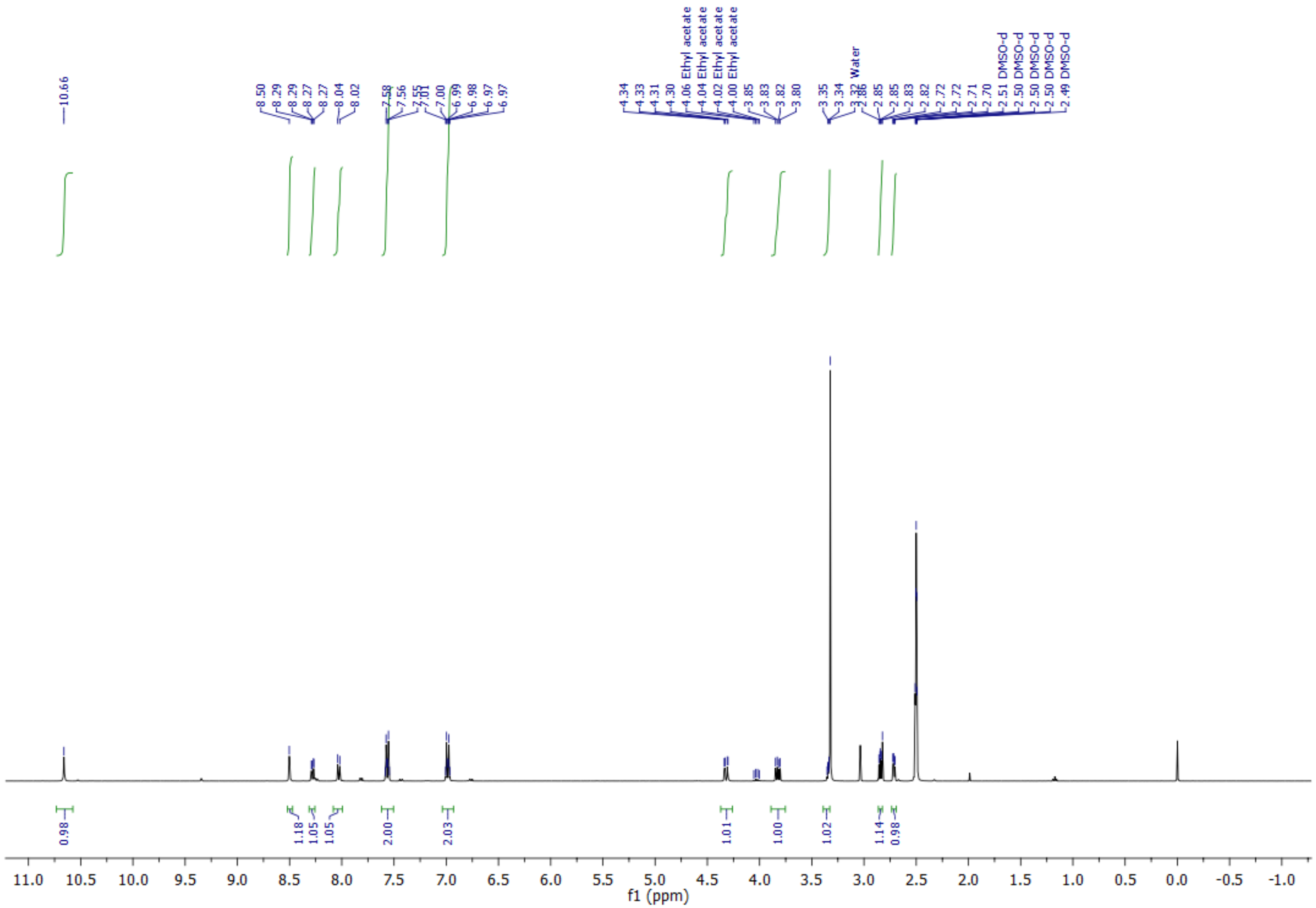

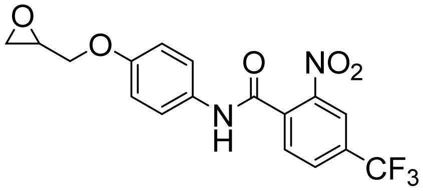

Methyl 2-((4-(oxiran-2-ylmethoxy)phenyl)amino)benzoate (4d)

^{1}H NMR (400 MHz, DMSO) δ 9.17 (s, 1H), 7.86 (dd, J_1 = 8.0 Hz, J_2 = 1.5 Hz, 1H), 7.34 (ddd, J_1 = 8.6 Hz, J_2 = 7.2 Hz, J_3 = 1.6 Hz, 1H), 7.22 – 7.15 (m, 2H), 7.02 – 6.96 (m, 2H), 6.95 (dd, J_1 = 8.5 Hz, J_2 = 0.7 Hz, 1H), 6.71 (ddd, J_1 = 8.1 Hz, J_2 = 7.1 Hz, J_3 = 1.1 Hz, 1H), 4.32 (dd, J_1 = 11.4 Hz, J_2 = 2.7 Hz, 1H), 3.84 (s, 3H), 3.88 – 3.82 (m, 1H), 3.39 – 3.28 (m, 1H), 2.85 (dd, J_1 = 5.0 Hz, J_2 = 4.3 Hz, 1H), 2.71 (dd, J_1 = 5.1 Hz, J_2 = 2.7 Hz, 1H).

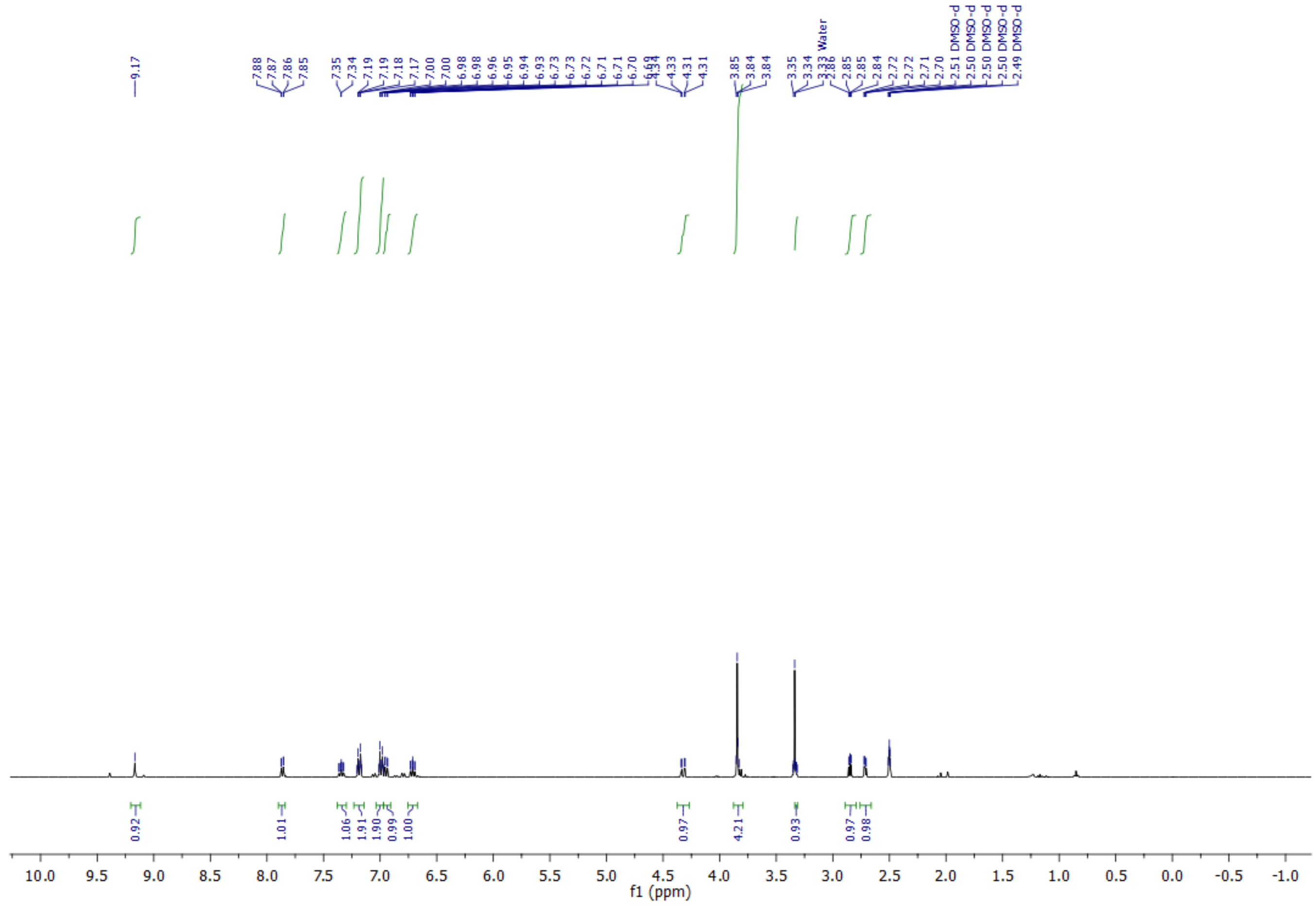

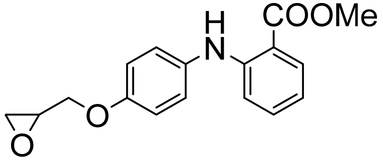

Methyl 2-((4-(oxiran-2-ylmethoxy)phenyl)amino)-5-(trifluoromethyl)benzoate (4e)

^{1}H NMR (400 MHz, DMSO) δ 9.49 (s, 1H), 8.10 (d, J = 1.7 Hz, 1H), 7.63 (dd, J_1 = 9.0 Hz, J_2 = 2.3 Hz, 1H), 7.29 – 7.19 (m, 2H), 7.08 – 7.00 (m, 2H), 6.97 (d, J = 9.0 Hz, 1H), 4.35 (dd, J_1 = 11.4 Hz, J_2 = 2.6 Hz, 1H), 3.89 (s, 3H), 3.88 – 3.80 (m, 1H), 3.40 – 3.33 (m, 1H), 2.86 (dd, J_1 = 5.0 Hz, J_2 = 4.3 Hz, 1H), 2.72 (dd, J_1 = 5.1 Hz, J_2 = 2.7 Hz, 1H).

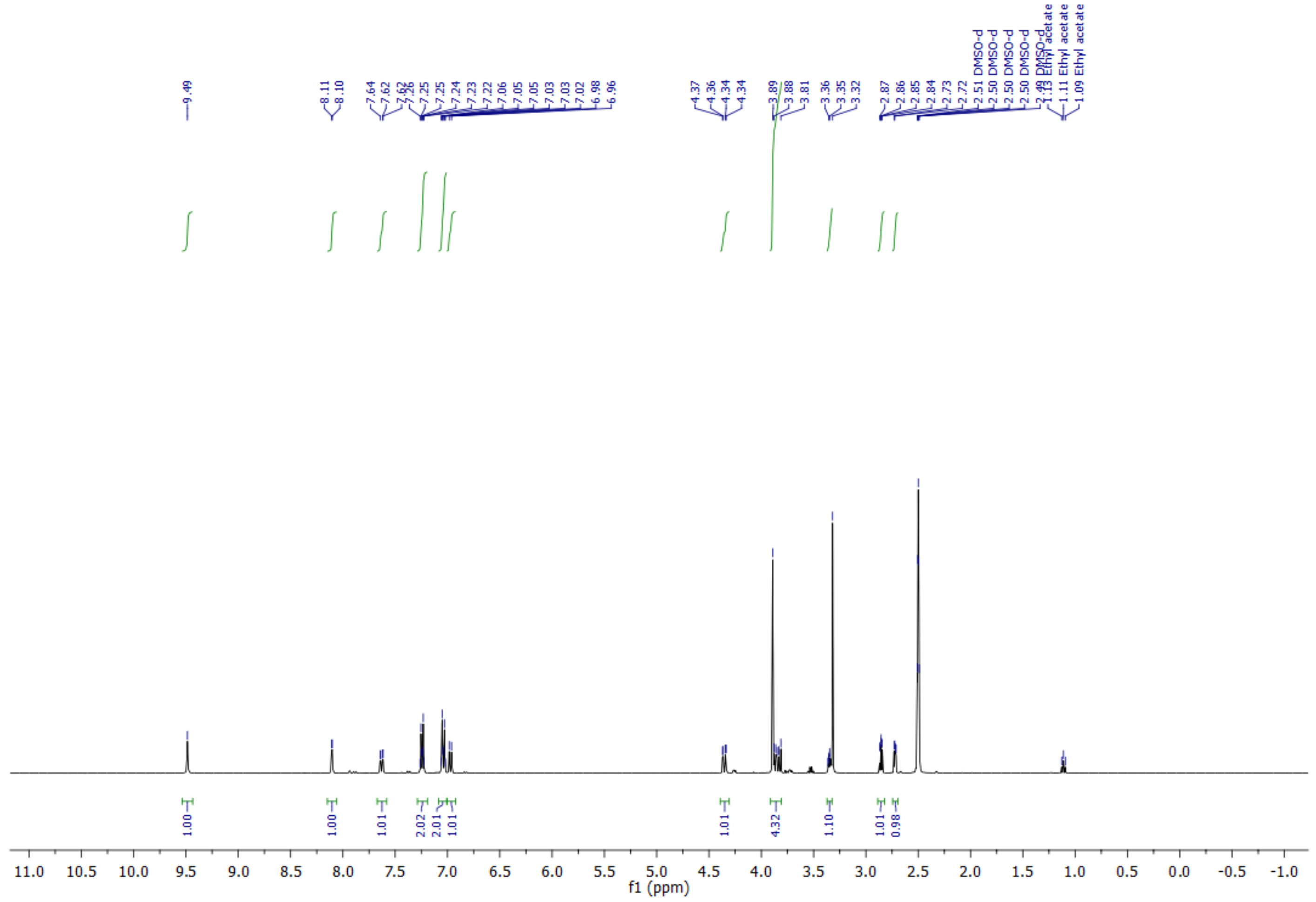

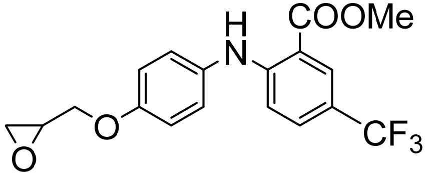

2-Nitro-*N*-(4-(oxiran-2-ylmethoxy)phenyl)-4-(trifluoromethyl)aniline (4f)

^{1}H NMR (400 MHz, $CDCl_3$) δ 9.61 (s, 1H), 8.50 (d, *J* = 1.1 Hz, 1H), 7.50 (dd, J_1 = 9.1 Hz, J_2 = 2.1 Hz, 1H), 7.24 – 7.15 (m, 2H), 7.10 – 6.95 (m, 3H), 4.31 (dd, J_1 = 11.1 Hz, J_2 = 2.9 Hz, 1H), 3.98 (dd, J_1 = 11.1 Hz, J_2 = 5.8 Hz, 1H), 3.46 – 3.32 (m, 1H), 3.00 – 2.91 (m, 1H), 2.79 (dd, J_1 = 4.9 Hz, J_2 = 2.7 Hz, 1H).

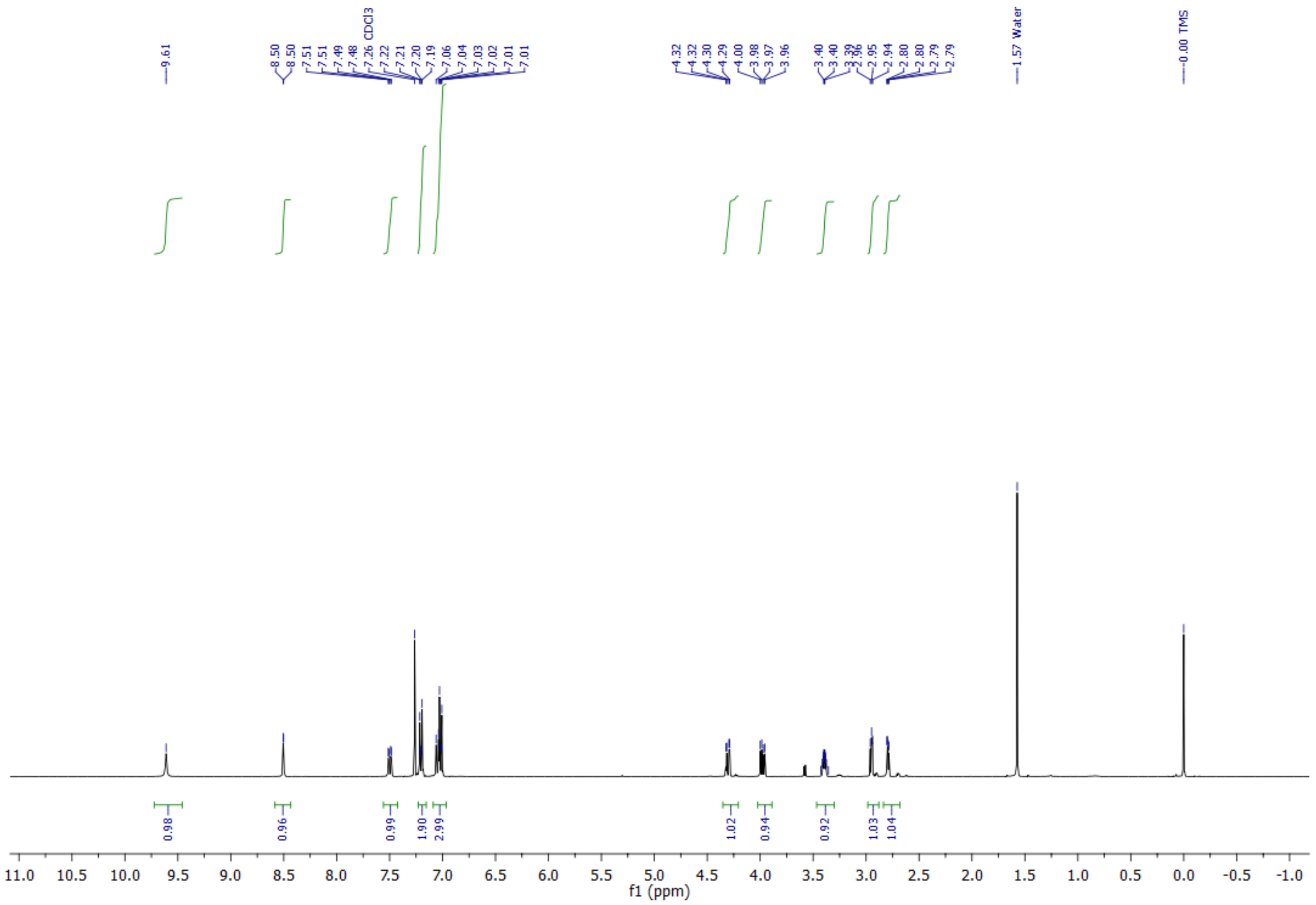

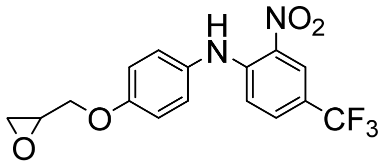

2,4-Dinitro-*N*-(4-(oxiran-2-ylmethoxy)phenyl)aniline (4g)

^{1}H NMR (400 MHz, DMSO) δ 10.10 (s, 1H), 8.89 (d, J = 2.7 Hz, 1H), 8.20 (dd, J_1 = 9.6 Hz, J_2 = 2.8 Hz, 1H), 7.38 – 7.23 (m, 2H), 7.16 – 7.04 (m, 2H), 6.96 (d, J = 9.6 Hz, 1H), 4.39 (dd, J_1 = 11.4 Hz, J_2 = 2.6 Hz, 1H), 3.88 (dd, J_1 = 11.4 Hz, J_2 = 6.6 Hz, 1H), 3.43 – 3.32 (m, 1H), 2.87 (dd, J_1 = 5.0 Hz, J_2 = 4.3 Hz, 1H), 2.73 (dd, J_1 = 5.1, J_2 = 2.7 Hz, 1H).

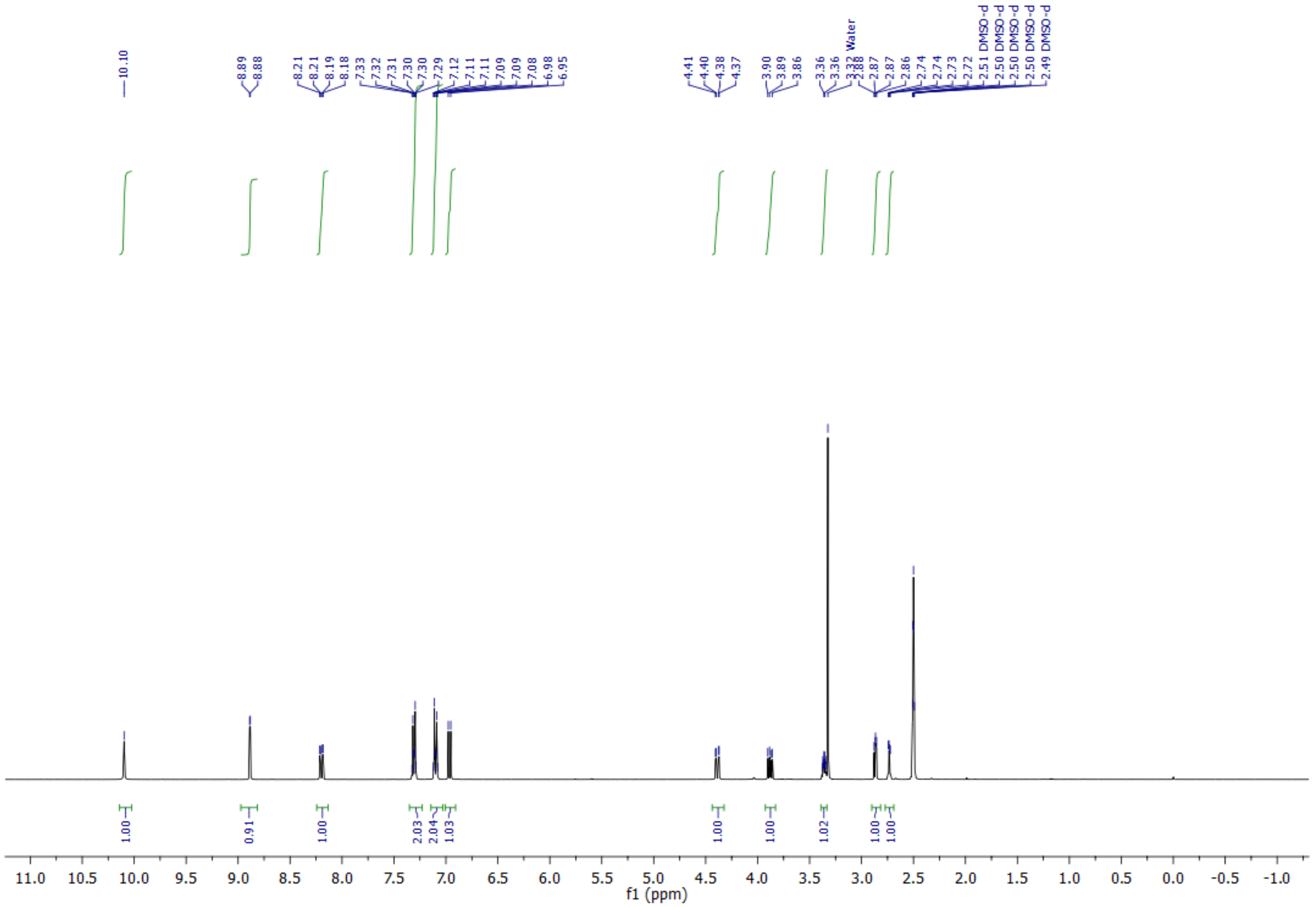

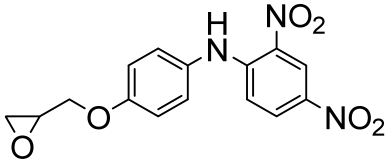

Methyl 2-(4-(2-hydroxy-3-morpholinopropoxy)phenoxy)benzoate (5a)

^{1}H NMR (400 MHz, MeOD) δ 7.82 (dd, J_1 = 7.8 Hz, J_2 = 1.7 Hz, 1H), 7.45 (ddd, J_1 = 8.4 Hz, J_2 = 7.4 Hz, J_3 = 1.8 Hz, 1H), 7.13 (td, J_1 = 7.7 Hz, J_2 = 1.0 Hz, 1H), 6.99 – 6.88 (m, 4H), 6.85 (dd, J_1 = 8.3 Hz, J_2 = 0.8 Hz, 1H), 4.87 (brs, 1H), 4.12 (ddd, J_1 = 9.2 Hz, J_2 = 7.4 Hz, J_3 = 5.3 Hz, 1H), 3.99 (dd, J_1 = 9.7 Hz, J_2 = 4.1 Hz, 1H), 3.91 (dd, J_1 = 9.8 Hz, J_2 = 5.9 Hz, 1H), 3.80 (s, 3H), 3.74 – 3.60 (m, 4H), 2.62 – 2.47 (m, 6H)

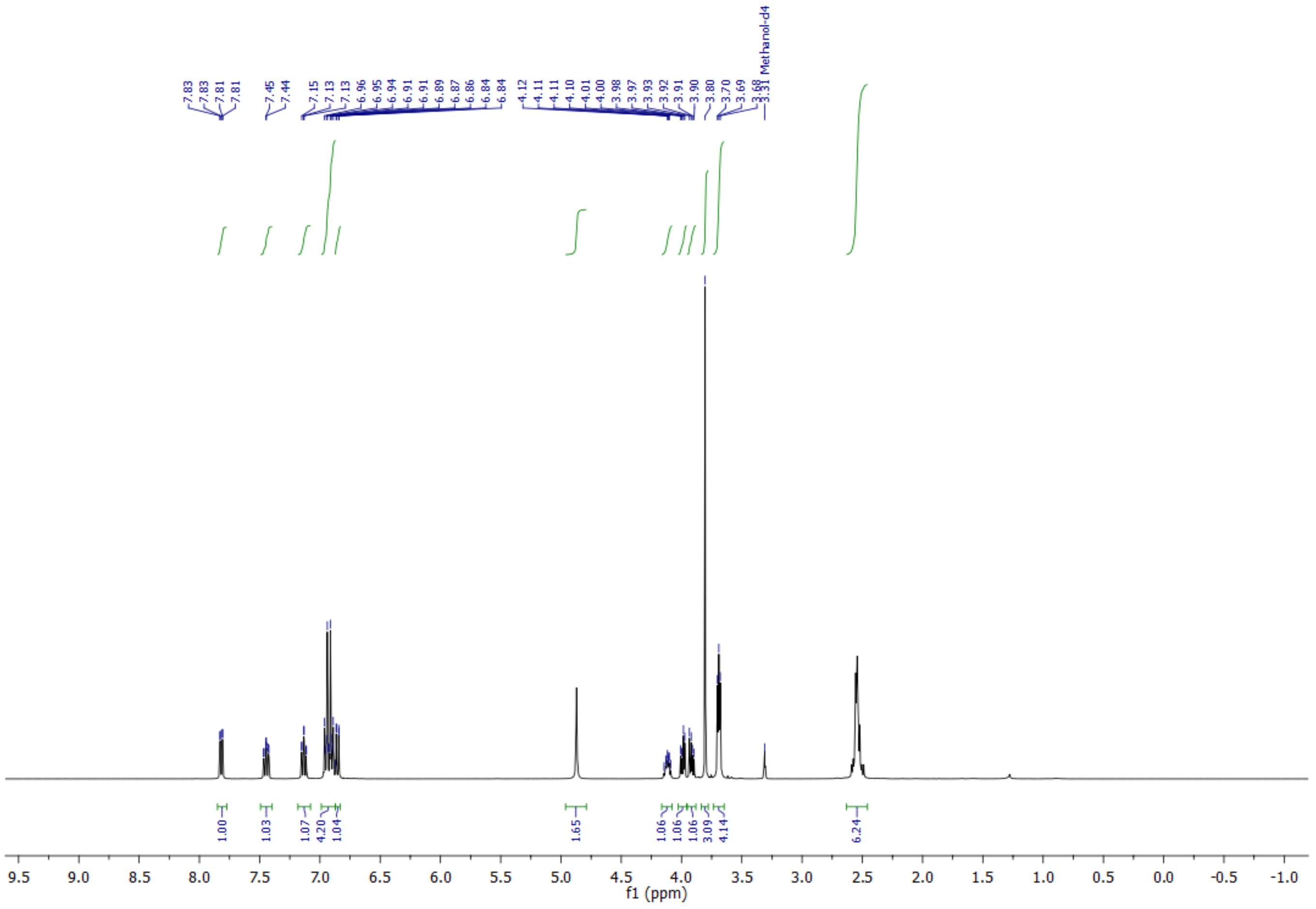

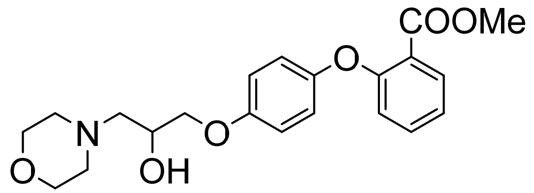

^{13}C NMR (101 MHz, MeOD) δ 168.01, 158.65, 156.71, 151.89, 134.71, 132.55, 123.76, 123.47, 121.21, 120.07, 116.78, 72.42, 68.11, 67.78, 62.57, 55.35, 52.62; HRMS (ESI$^+$) for $C_{21}H_{26}NO_6$ ([M+H]$^+$) calculated 388.1755 found 388.1745.

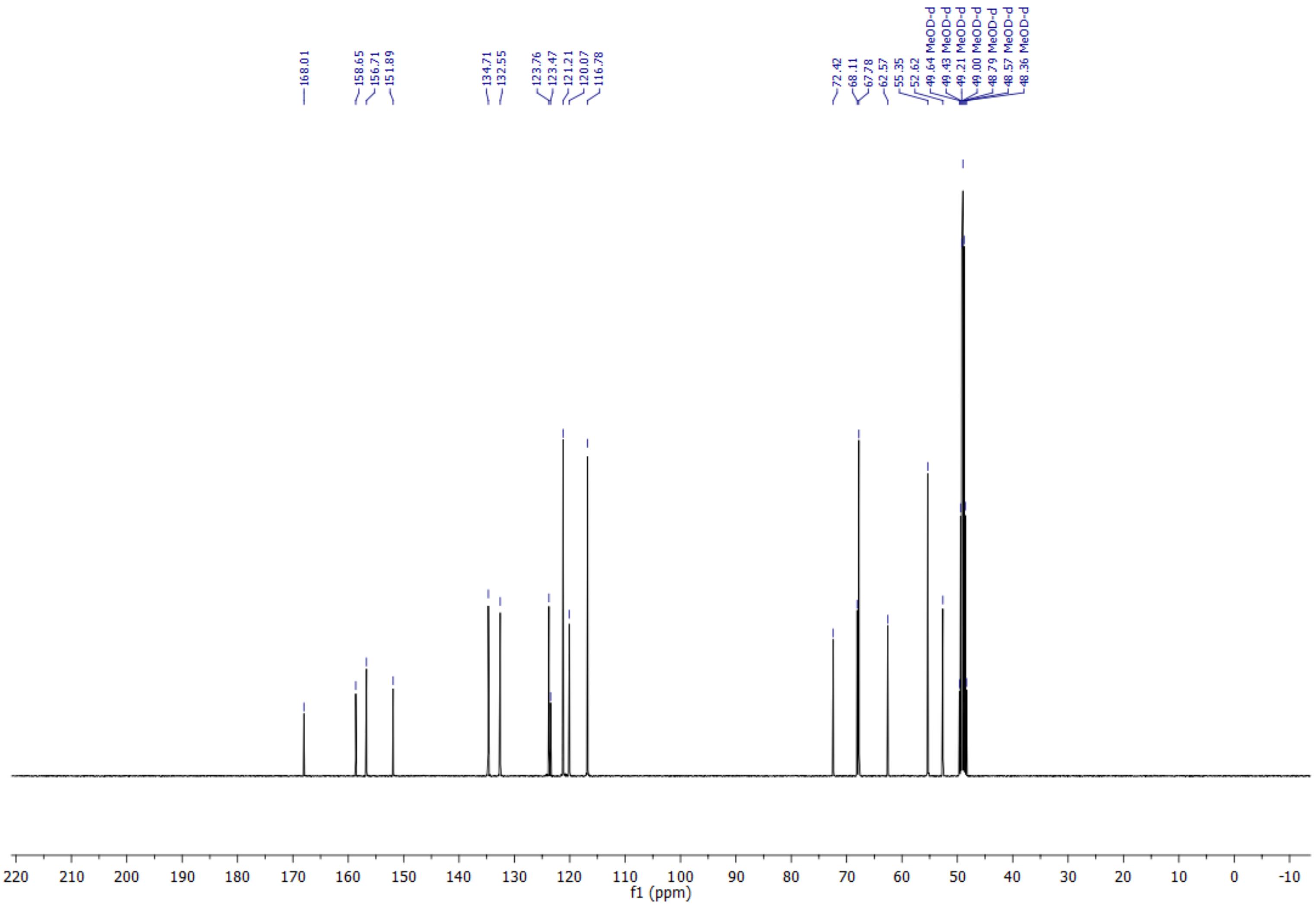

1-Morpholino-3-(4-(2-nitro-4-(trifluoromethyl)phenoxy)phenoxy)propan-2-ol (5b)

^{1}H NMR (400 MHz, $CDCl_3$) δ 8.21 (d, *J* = 1.9 Hz, 1H), 7.71 – 7.64 (m, 1H), 7.09 – 7.03 (m, 2H), 6.99 (dt, J_1 = 5.7 Hz, J_2 = 3.3 Hz, 3H), 4.18 (dq, J_1 = 9.2 Hz, J_2 = 4.7 Hz, 1H), 4.05 – 3.95 (m, 2H), 3.85 – 3.70 (m, 5H), 2.81 – 2.71 (m, 2H), 2.70 – 2.60 (m, 2H), 2.60 – 2.52 (m, 2H).

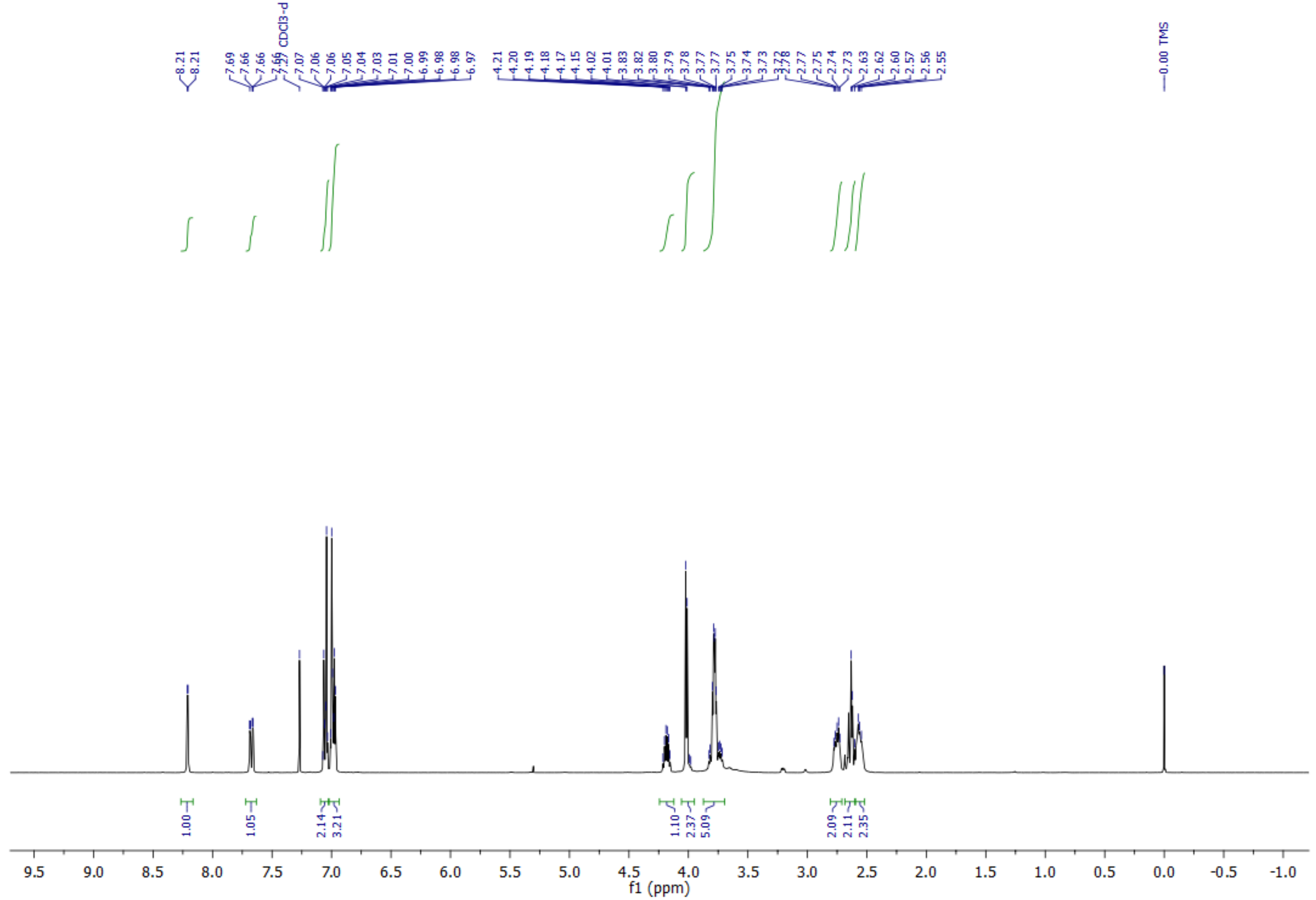

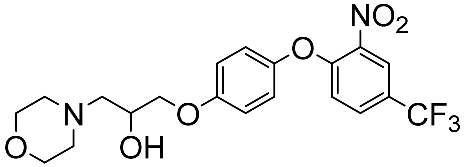

^{13}C NMR (101 MHz, $CDCl_3$) δ 156.71, 154.78, 147.72, 139.90, 130.86 (q, *J* = 3.4 Hz), 124.52 (q, *J* = 34.5 Hz), 123.64 (q, *J* = 3.9 Hz), 123.01 (q, *J* = 271.0 Hz), 121.80, 118.60, 116.27, 70.77, 66.86, 65.34, 61.18, 53.87. HRMS (ESI$^+$) for $C_{20}H_{22}F_3N_2O_6$ ([M+H]$^+$) calculated 443.1425 found 443.1415.

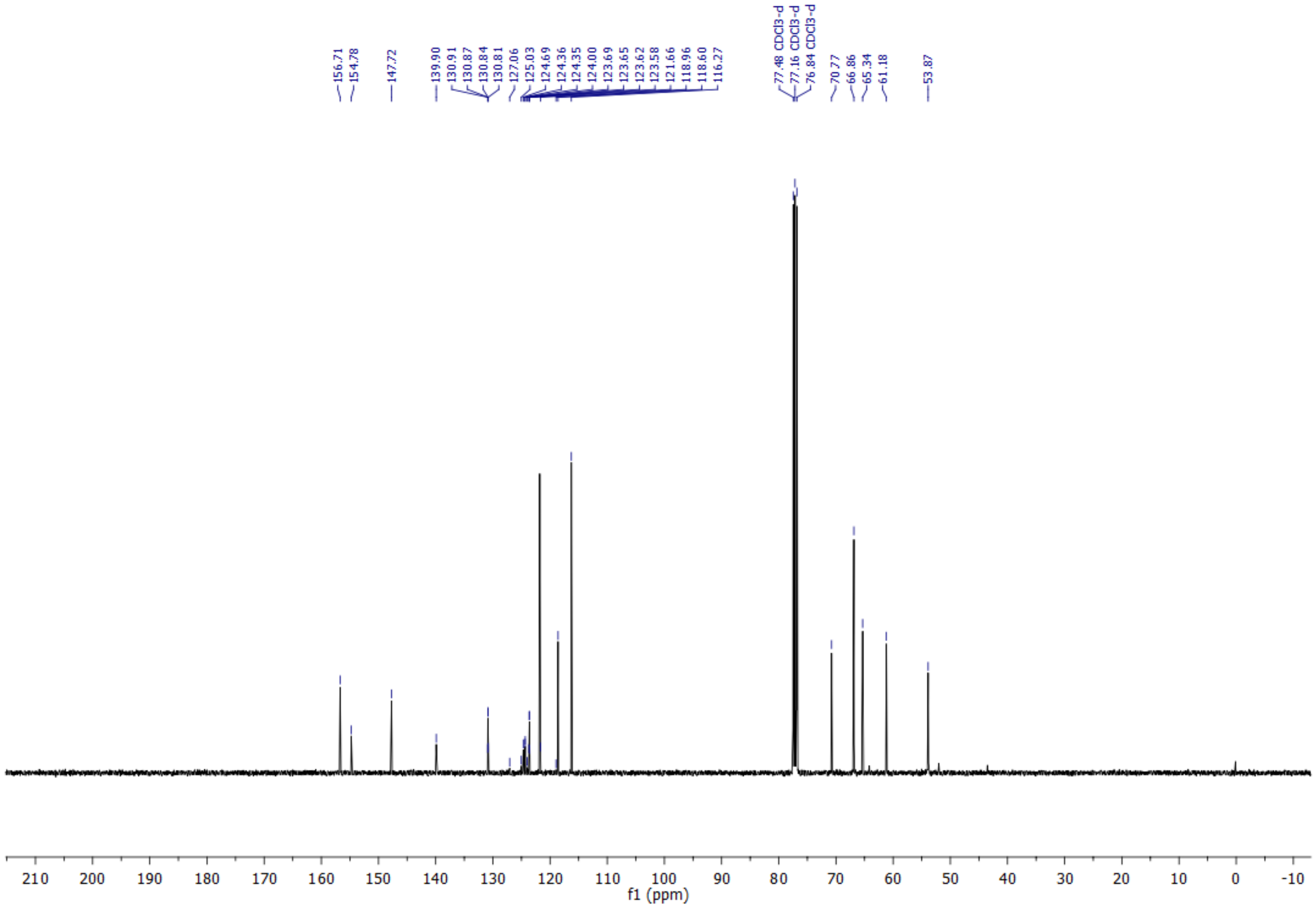

***N*-(4-(2-hydroxy-3-morpholinopropoxy)phenyl)-2-nitro-4-(trifluoromethyl)benzamide (5c)**

^{1}H NMR (400 MHz, DMSO) δ 10.63 (s, 1H), 8.50 (d, J = 1.0 Hz, 1H), 8.28 (dd, J_1 = 8.0 Hz, J_2 = 1.1 Hz, 1H), 8.02 (d, J = 7.9 Hz, 1H), 7.59 – 7.49 (m, 2H), 7.00 – 6.91 (m, 2H), 4.87 (d, J = 4.5 Hz, 1H), 4.00 – 3.92 (m, 2H), 3.86 (dd, J_1 = 11.0 Hz, J_2 = 7.3 Hz, 1H), 3.57 (t, J = 4.6 Hz, 4H), 2.48 – 2.30 (m, 6H)

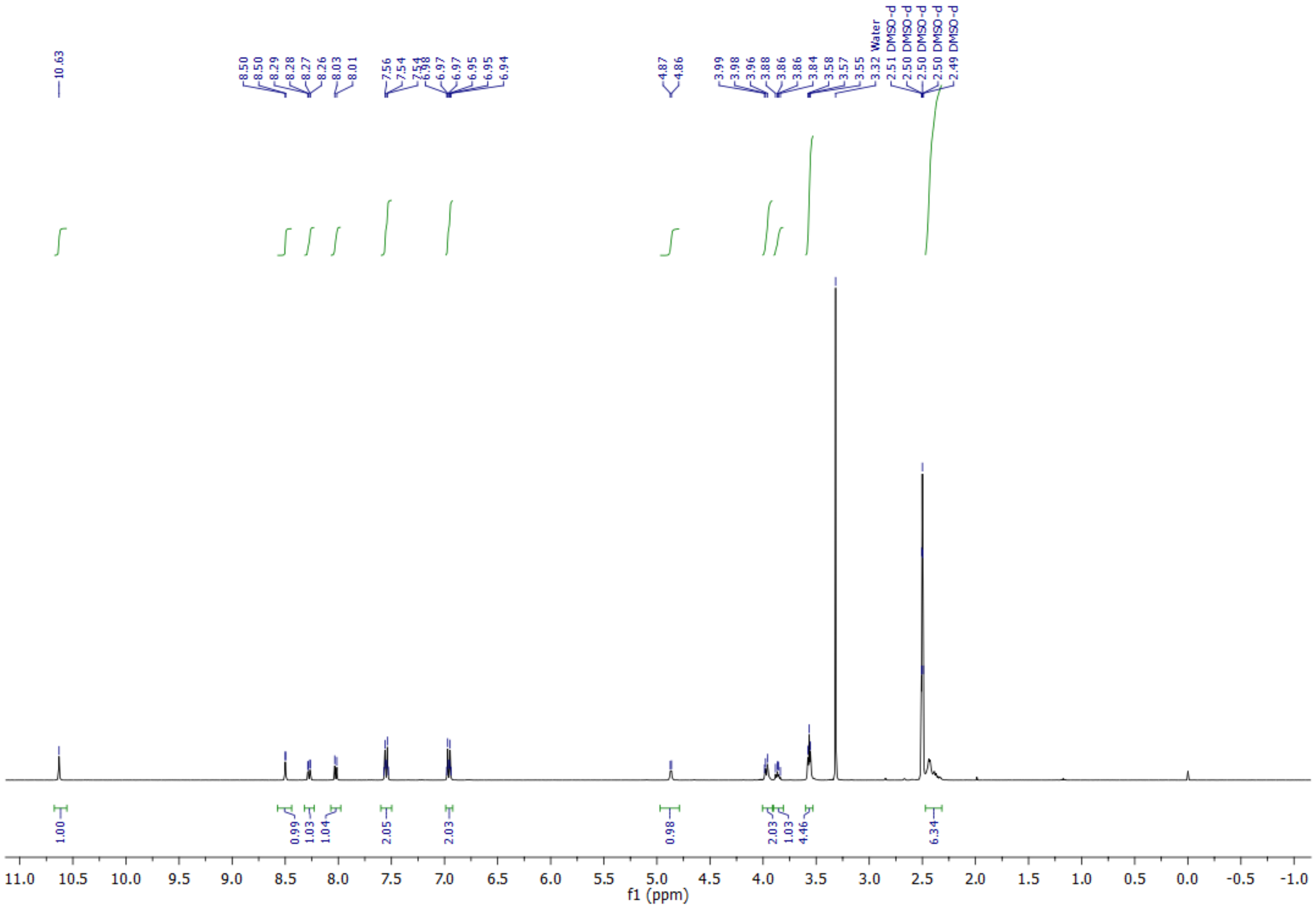

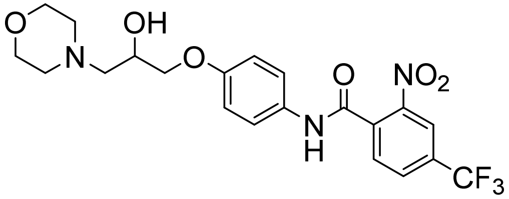

^{13}C NMR (101 MHz, MeOD) δ 165.61, 157.71, 147.99, 137.53, 133.66 (q, *J* = 34.2 Hz), 132.56, 131.82 (q, *J* = 3.4 Hz), 131.62, 124.20 (q, *J* = 271.0 Hz), 123.34, 122.88 (q, *J* = 3.8 Hz), 115.91, 72.19, 68.17, 67.84, 62.60, 55.40. HRMS (ESI$^+$) for $C_{21}H_{23}F_3N_3O_6$ ([M+H]$^+$) calculated 470.1534 found 470.1523

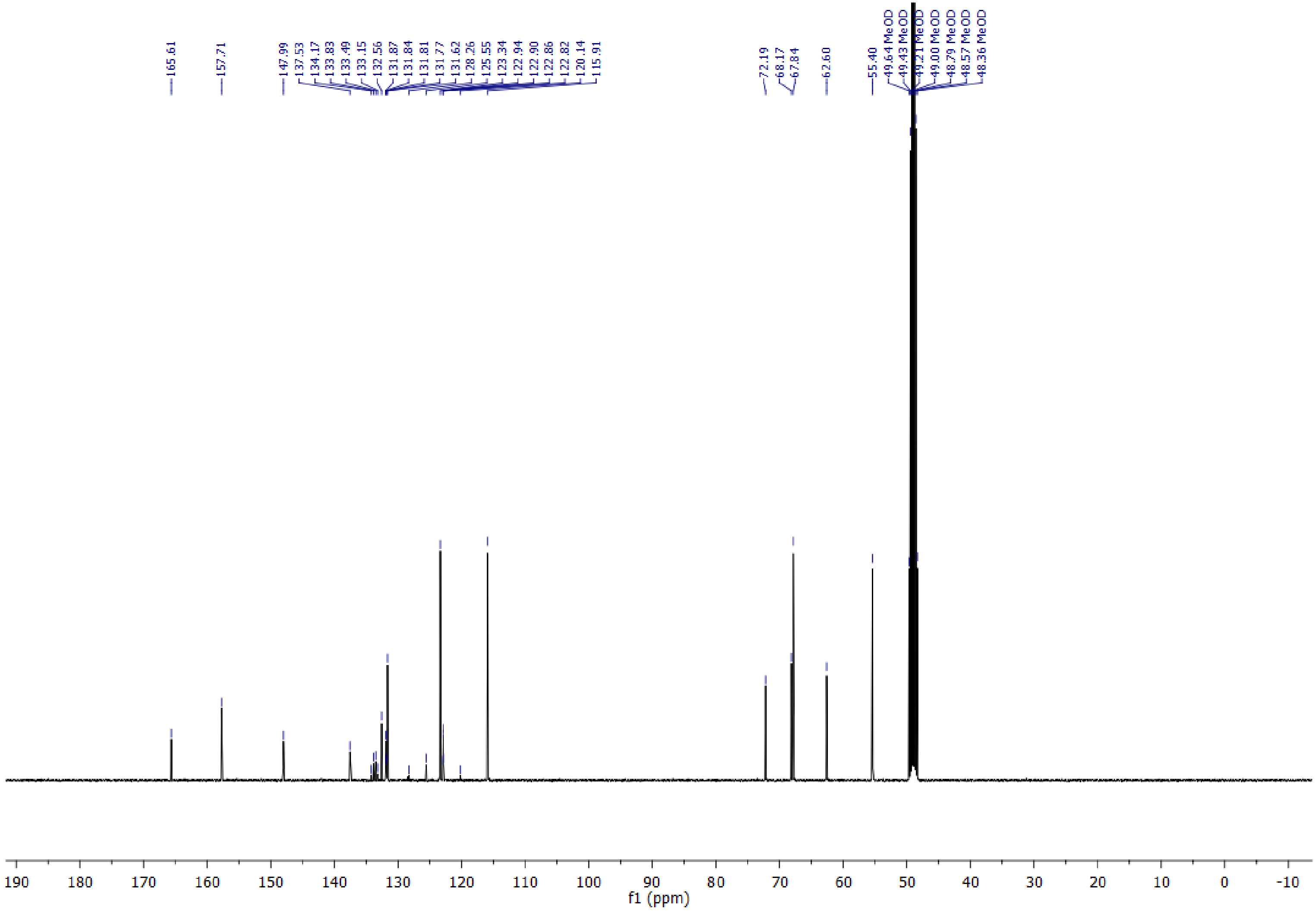

Methyl 2-((4-(2-hydroxy-3-morpholinopropoxy)phenyl)amino)benzoate (5d)

^{1}H NMR (400 MHz, DMSO) δ 9.15 (s, 1H), 7.86 (dd, J_1 = 8.0 Hz, J_2 = 1.6 Hz, 1H), 7.34 (ddd, J_1 = 8.6 Hz, J_2 = 7.2 Hz, J_3 = 1.6 Hz, 1H), 7.21 – 7.13 (m, 2H), 7.00 – 6.95 (m, 2H), 6.93 (dd, J_1 = 8.6 Hz, J_2 = 0.7 Hz, 1H), 6.71 (ddd, J_1 = 8.1 Hz, J_2 = 7.1 Hz, J_3 = 1.1 Hz, 1H), 4.87 (d, J = 4.7 Hz, 1H), 4.01 – 3.92 (m, 2H), 3.87 (dd, J_1 = 10.0 Hz, J_2 = 6.4 Hz, 1H), 3.85 (s, 3H), 3.56 (t, J = 4.6 Hz, 4H), 2.48 – 2.31 (m, 6H)

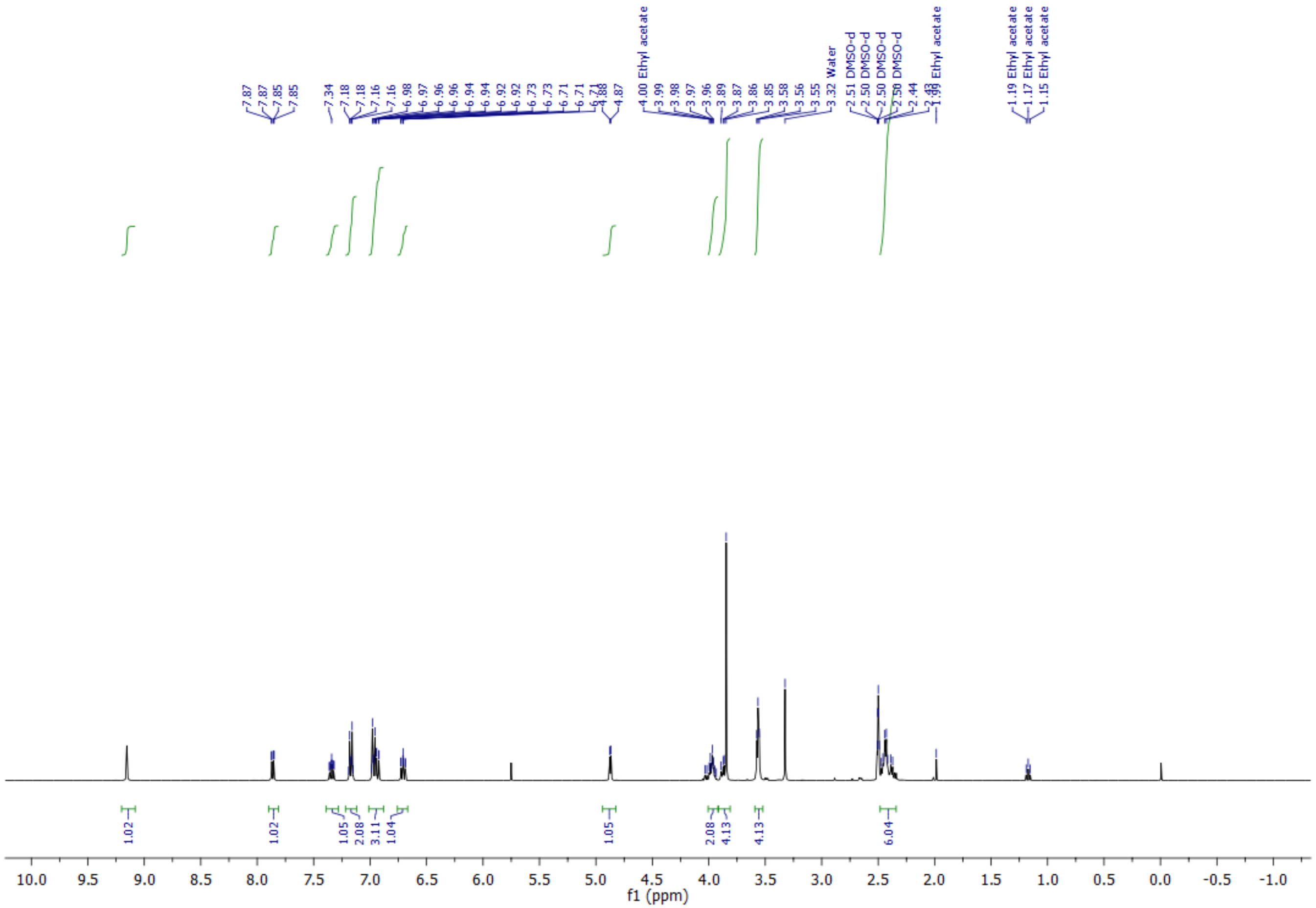

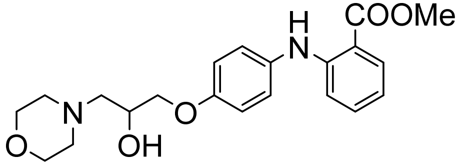

^{13}C NMR (101 MHz, MeOD) δ 170.24, 157.48, 150.64, 135.28, 134.88, 132.56, 126.63, 117.38, 116.53, 114.31, 112.04, 72.22, 68.14, 67.81, 62.61, 55.38, 52.16; HRMS (ESI$^+$) for $C_{21}H_{27}N_2O_5$ ([M+H]$^+$) calculated 387.1915 found 387.1905

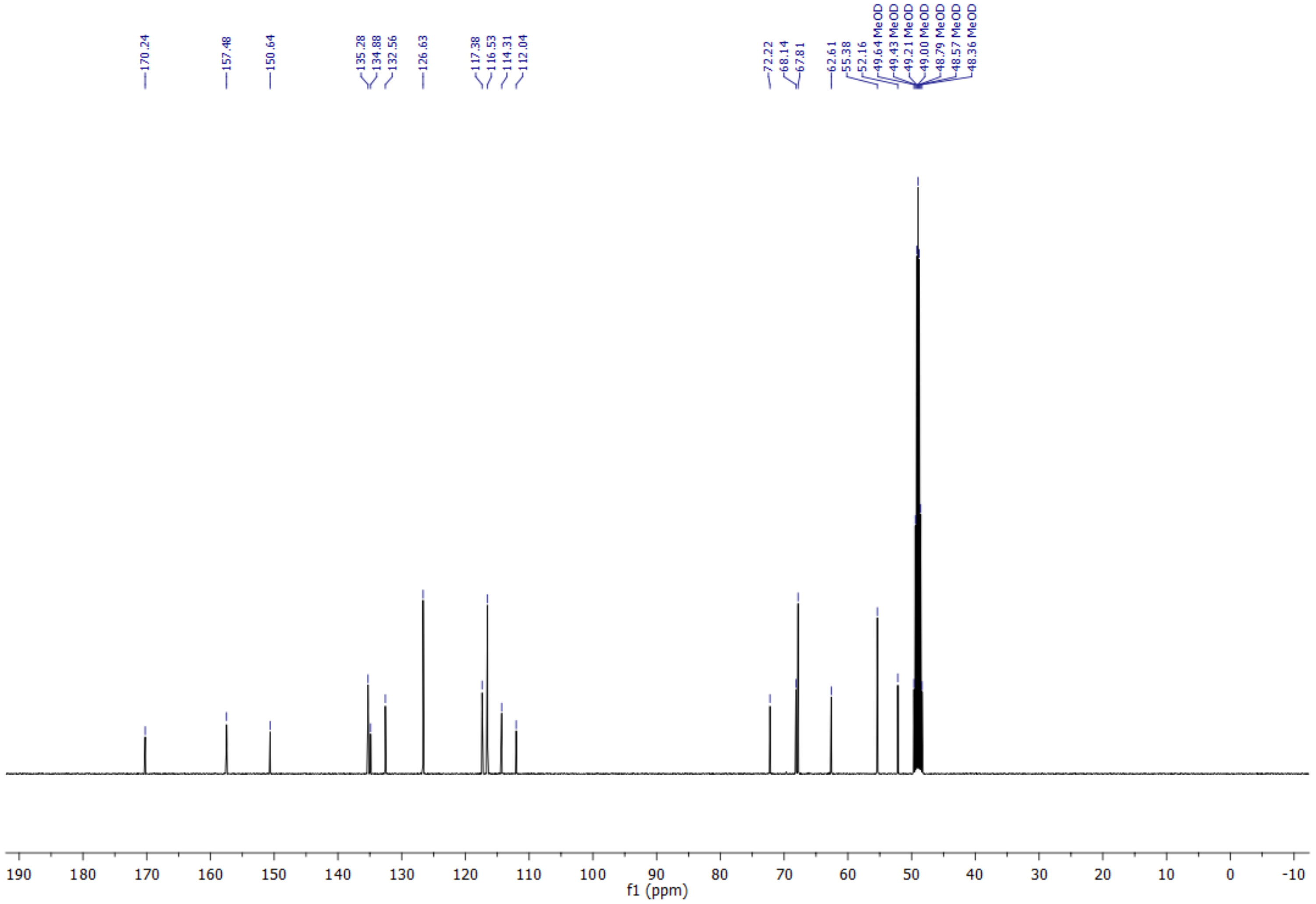

Methyl 2-((4-(2-hydroxy-3-morpholinopropoxy)phenyl)amino)benzoate (5e)

^{1}H NMR (400 MHz, $CDCl_3$) δ 9.57 (s, 1H), 8.21 (d, *J* = 1.4 Hz, 1H), 7.43 (dd, J_1 = 9.0 Hz, J_2 = 2.2 Hz, 1H), 7.21 – 7.12 (m, 2H), 7.02 – 6.89 (m, 3H), 4.13 (tt, J_1 = 9.5 Hz, J_2 = 4.9 Hz, 1H), 4.05 – 3.97 (m, 2H), 3.93 (s, 3H), 3.81 – 3.66 (m, 4H), 2.76 – 2.64 (m, 2H), 2.64 – 2.53 (m, 2H), 2.54 – 2.43 (m, 2H)

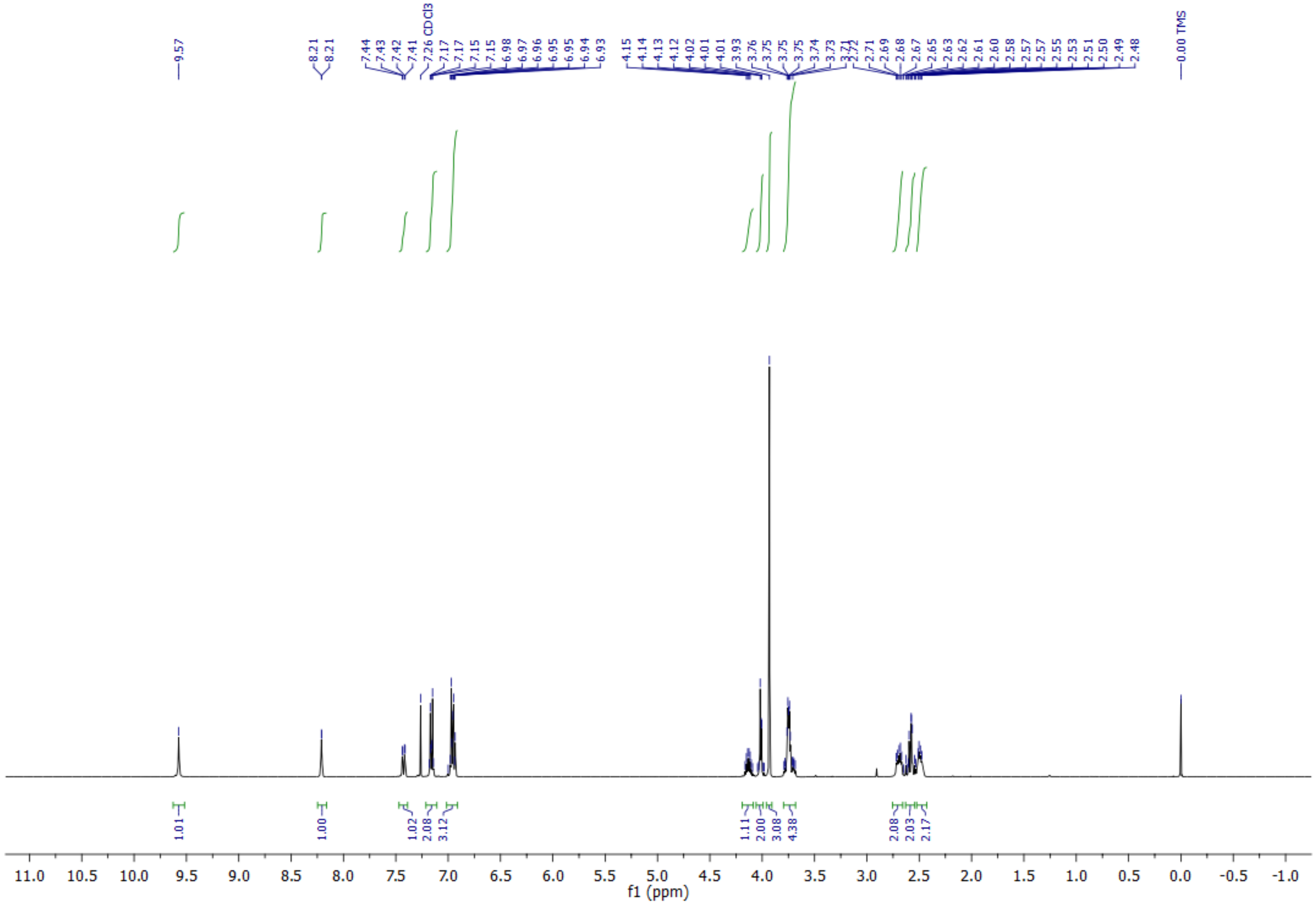

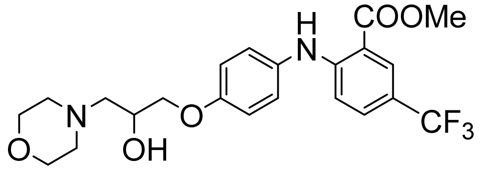

^{13}C NMR (101 MHz, $CDCl_3$) δ 168.38, 156.75, 151.92, 132.51, 130.71 (q, *J* = 3.3 Hz), 129.46 (q, *J* = 3.9 Hz), 124.50 (q, *J* = 270.6 Hz), 126.74, 117.98 (q, *J* = 33.2 Hz), 115.69, 113.42, 109.99, 70.64, 67.15, 65.52, 61.14, 53.90, 52.16; HRMS (ESI$^+$) for $C_{22}H_{26}F_3N_2O_5$ ([M+H]$^+$) calculated 455.1788 found 455.1777

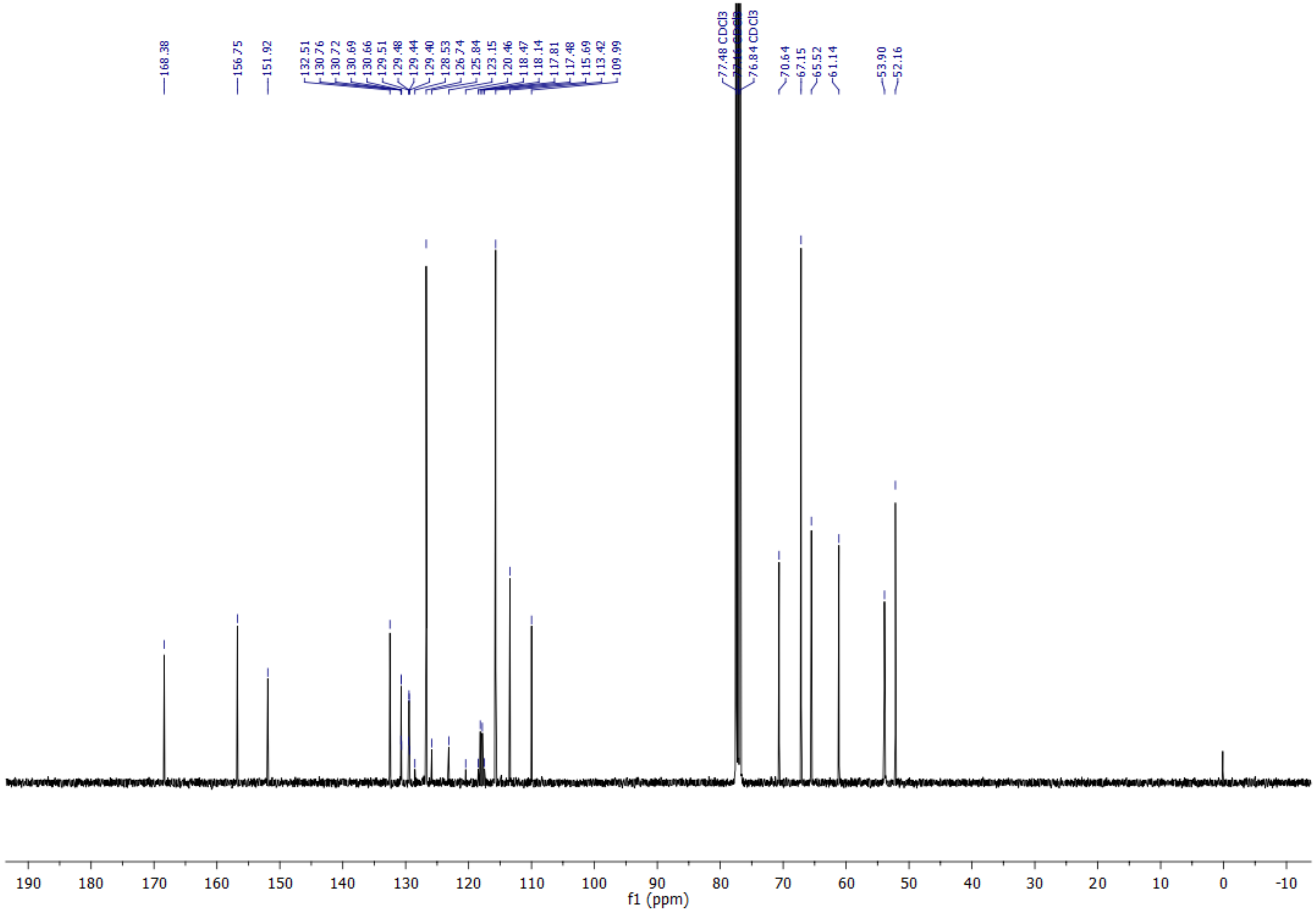

1-(4-((2,4-Dinitrophenyl)amino)phenoxy)-3-morpholinopropan-2-ol (5f)

^{1}H NMR (400 MHz, $CDCl_3$) δ 9.87 (s, 1H), 9.17 (d, J = 2.6 Hz, 1H), 8.21 – 8.07 (m, 1H), 7.25 – 7.19 (m, 2H), 7.09 – 6.94 (m, 3H), 4.16 (td, J_1 = 9.4 Hz, J_2 = 4.5 Hz, 1H), 4.09 – 3.98 (m, 2H), 3.85 – 3.68 (m, 4H), 2.79 – 2.44 (m, 6H)

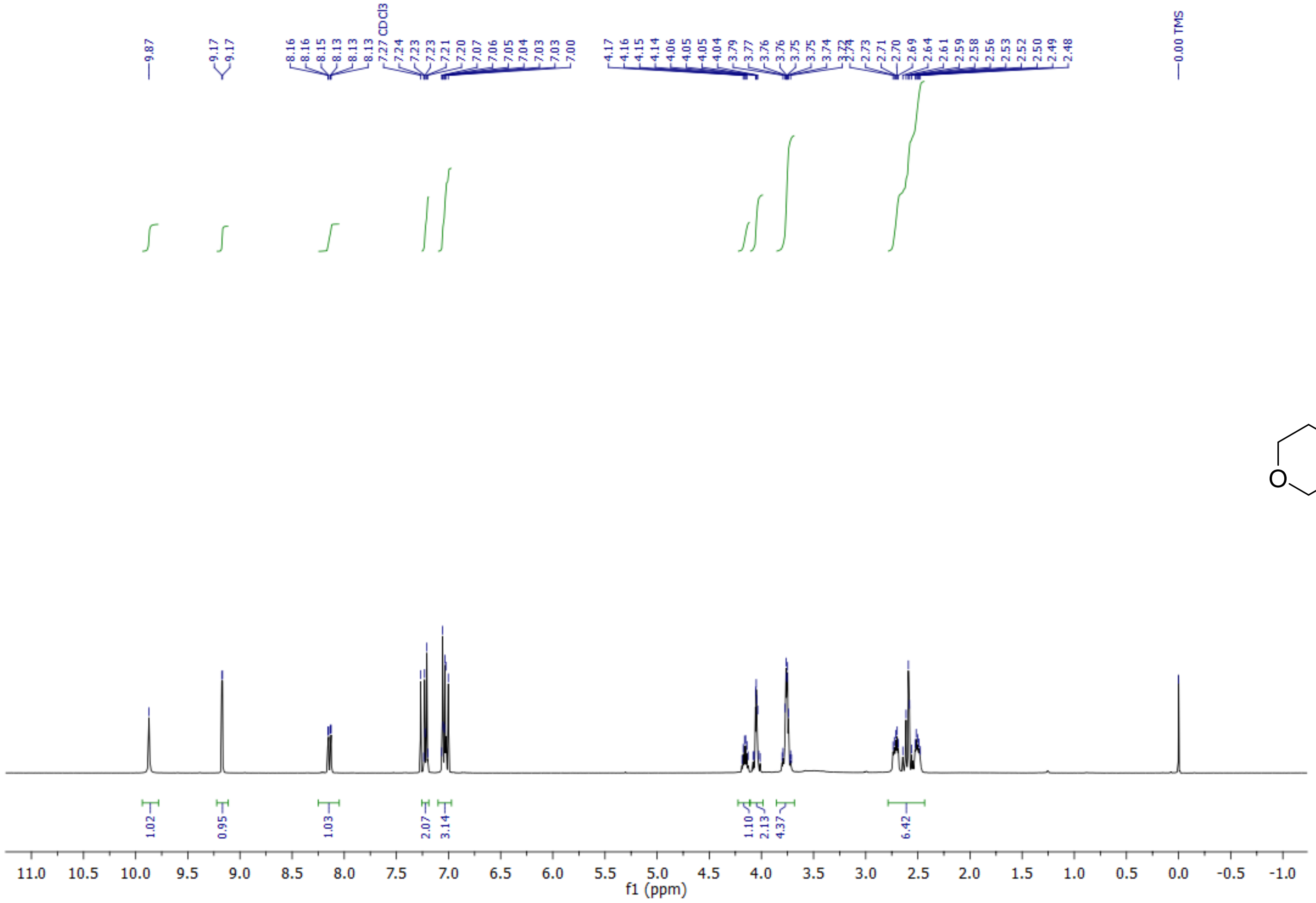

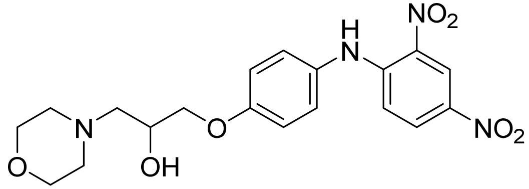

^{13}C NMR (101 MHz, $CDCl_3$) δ 158.35, 148.03, 137.24, 130.88, 130.01, 129.64, 127.59, 124.25, 116.19, 116.09, 70.69, 67.10, 65.39, 61.03, 53.86; HRMS (ESI+) for $C_{19}H_{23}N_4O_7$ ([M+H]+) calculated 419.1561 found 419.1550

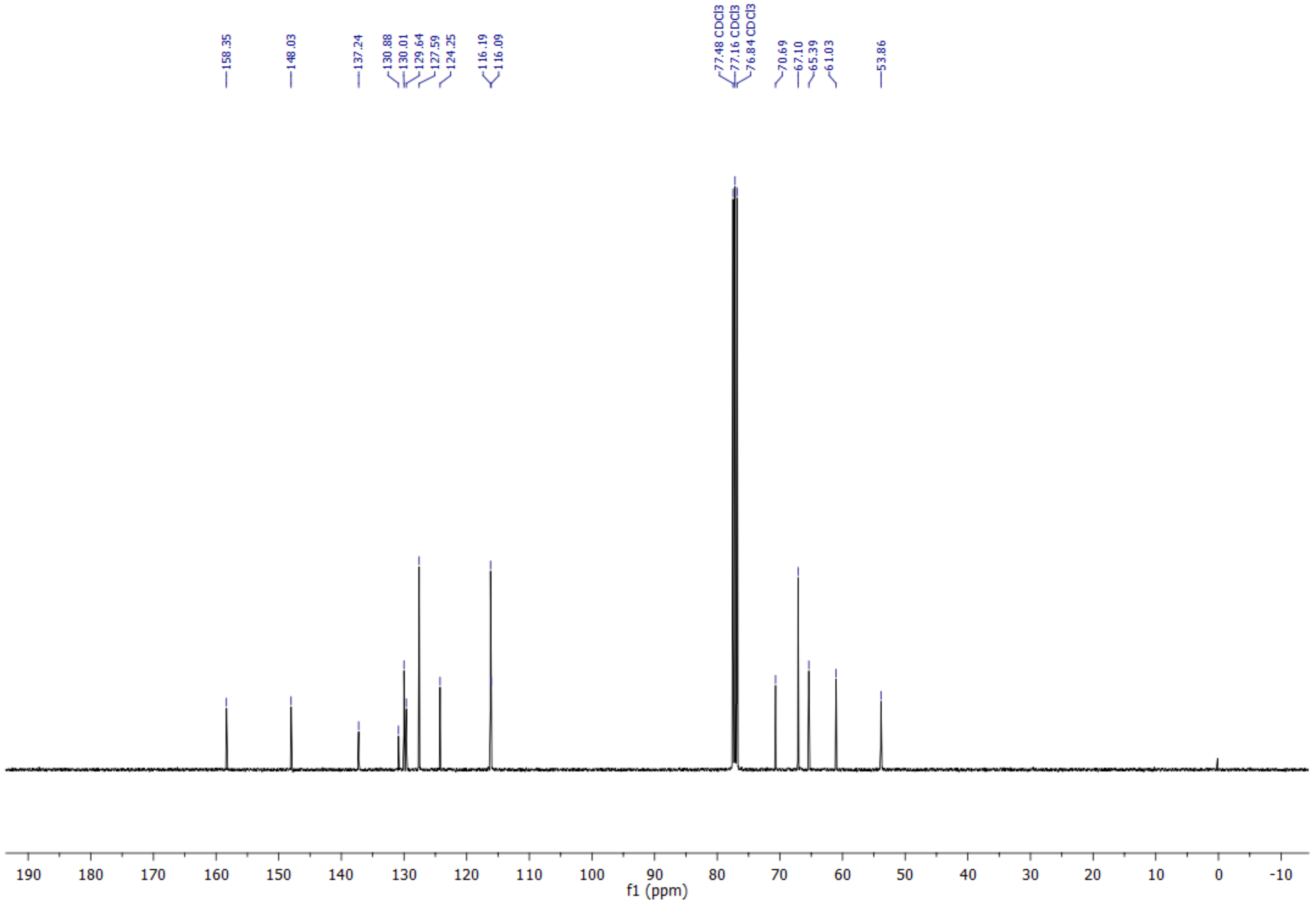

2-((4-(2-Nitro-4-(trifluoromethyl)phenoxy)phenoxy)methyl)oxirane (6a)

^{1}H NMR (400 MHz, $CDCl_3$) δ 8.19 (d, *J* = 1.8 Hz, 1H), 7.68 (dd, J_1 = 8.9 Hz, J_2 = 2.0 Hz, 1H), 7.09 – 6.93 (m, 5H), 4.36 (brs, 1H), 4.22 – 4.13 (m, 1H), 4.01 (d, *J* = 4.9 Hz, 2H), 2.77 – 2.67 (m, 1H), 2.55 (dd, J_1 = 12.4 Hz, J_2 = 2.7 Hz, 1H), 2.45 (s, 6H)

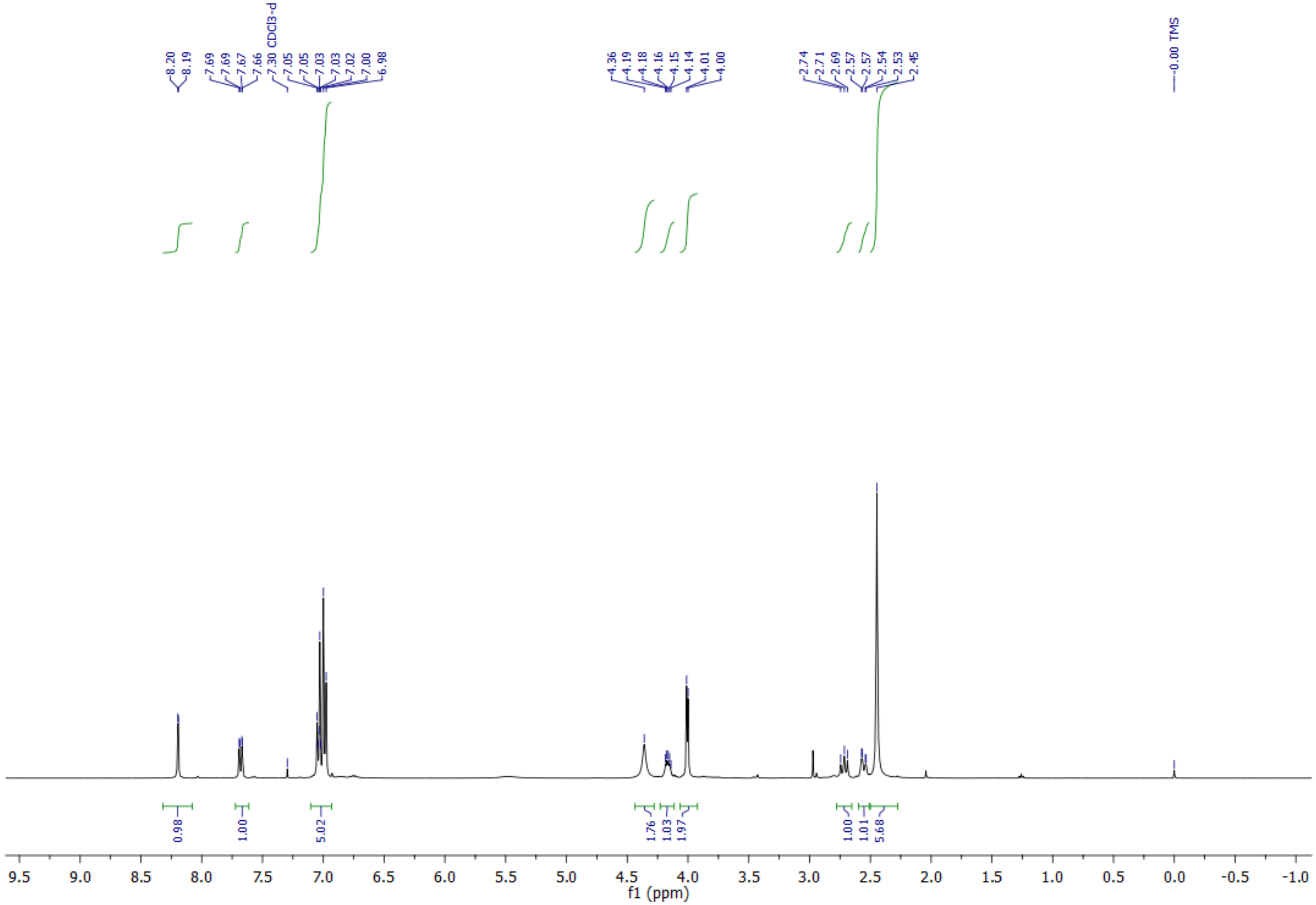

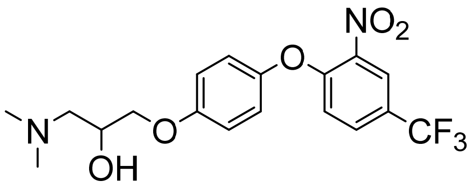

^{13}C NMR (101 MHz, $CDCl_3$) δ 156.43, 154.67, 147.80, 139.85, 130.95 (q, *J* = 3.7 Hz), 124.55 (q, *J* = 34.5 Hz), 123.60 (q, *J* = 3.8 Hz), 123.02 (q, *J* = 271.0 Hz), 121.75, 118.73, 116.30, 70.54, 65.41, 61.62, 45.07; HRMS (ESI$^+$) for $C_{18}H_{20}F_3N_2O_5$ ([M+H]$^+$) calculated 401.1319 found 401.1310

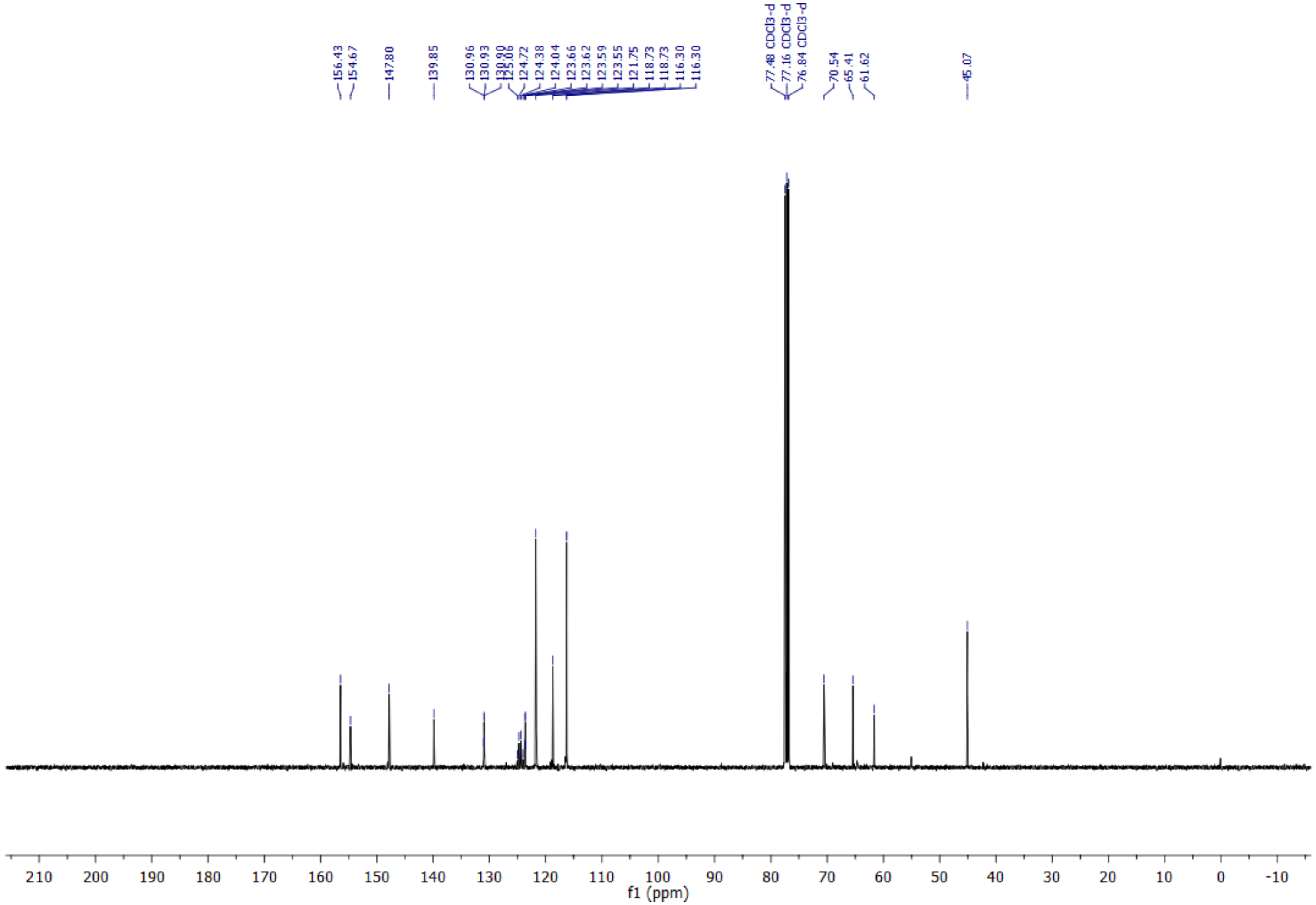

N-(4-(3-(dimethylamino)-2-hydroxypropoxy)phenyl)-2-nitro-4-(trifluoromethyl)benzamide (6b)

^{1}H NMR (400 MHz, DMSO) δ 10.77 (s, 1H), 9.82 (s, 1H), 8.50 (d, J = 0.9 Hz, 1H), 8.28 (dd, J_1 = 8.0 Hz, J_2 = 1.1 Hz, 1H), 8.03 (d, J = 7.9 Hz, 1H), 7.64 – 7.53 (m, 2H), 7.04 – 6.91 (m, 2H), 6.00 (d, J = 3.9 Hz, 1H), 4.29 (d, J = 2.5 Hz, 1H), 4.02 – 3.88 (m, 2H), 3.40 – 3.13 (m, 2H), 2.84 (d, J = 11.5 Hz, 6H)

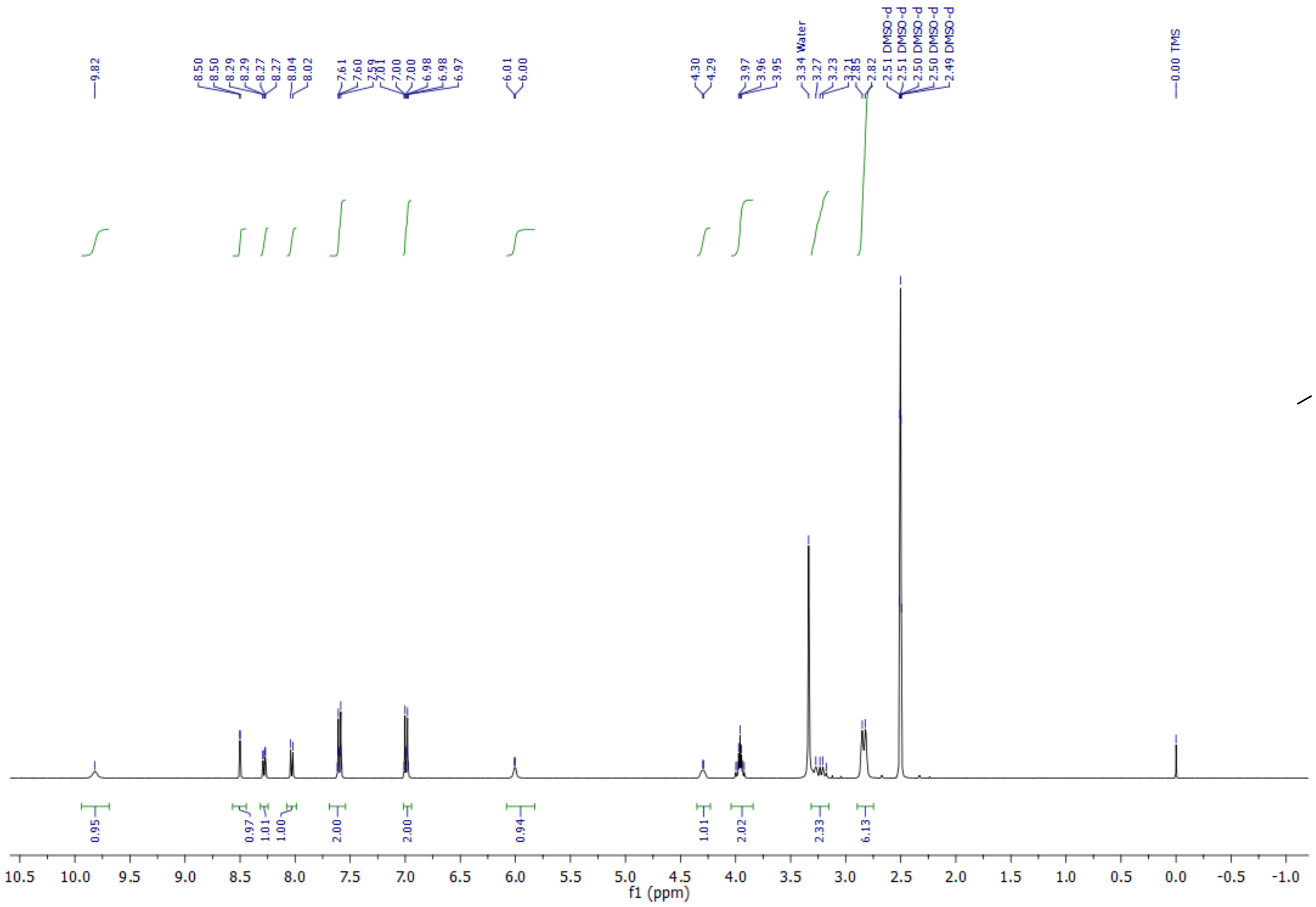

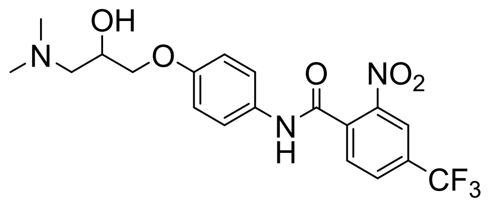

^{13}C NMR (101 MHz, MeOD) δ 165.70, 157.18, 148.00, 137.49, 133.73 (q, *J* = 34.2 Hz), 133.05, 131.86 (q, *J* = 3.5 Hz), 130.79, 123.76 (q, *J* = 271.0 Hz), 123.40, 122.90 (q, *J* = 3.3 Hz), 115.94, 71.30, 65.37, 61.01. HRMS (ESI$^+$) for $C_{19}H_{21}F_3N_3O_5$ ([M+H]$^+$) calculated 428.1428 found 428.1420

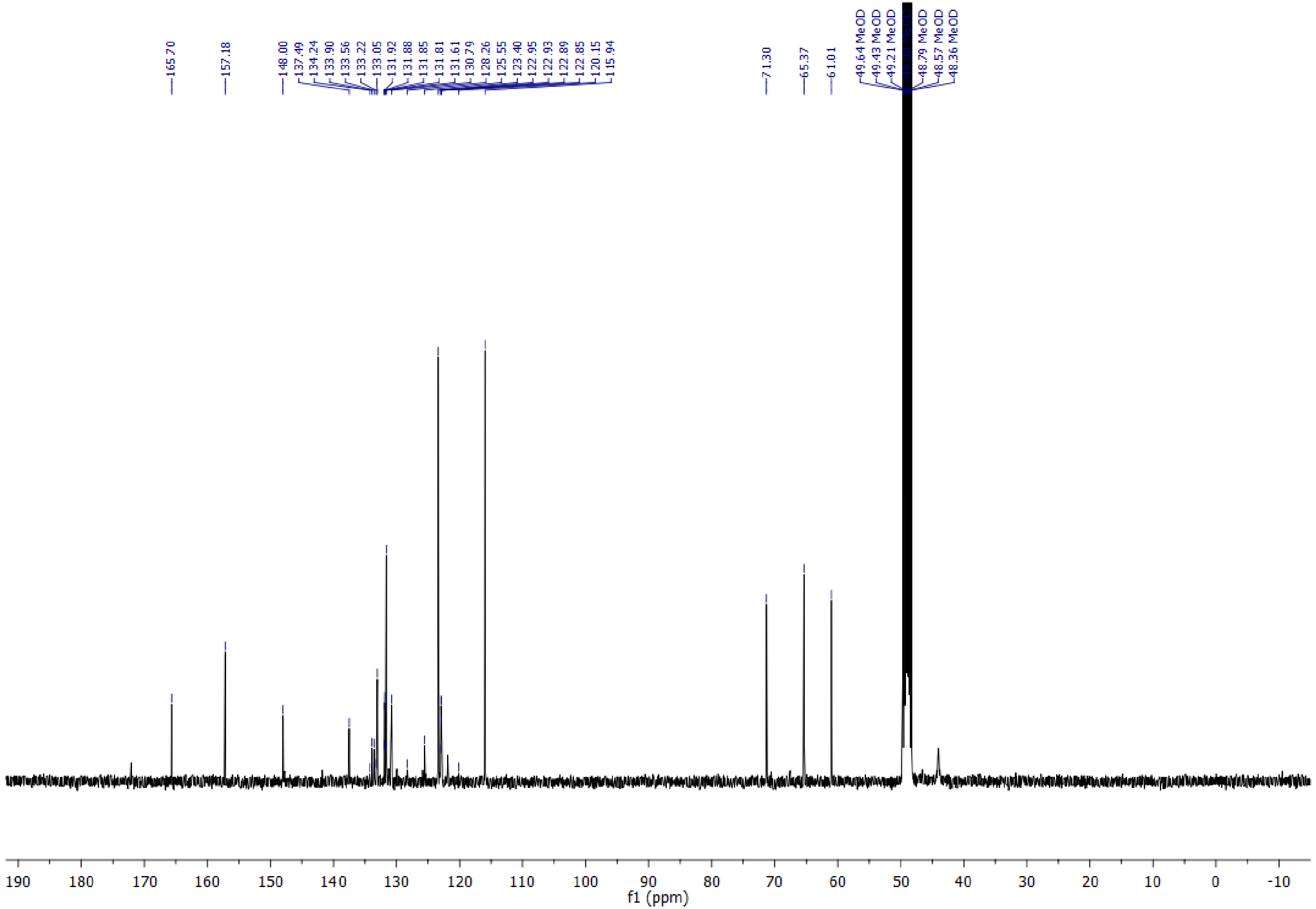

Methyl 2-((4-(3-(dimethylamino)-2-hydroxypropoxy)phenyl)amino)-5-(trifluoromethyl)benzoate (6c)

^{1}H NMR (400 MHz, $CDCl_3$) δ 9.56 (s, 1H), 8.21 (d, *J* = 1.4 Hz, 1H), 7.42 (dd, J_1 = 9.0 Hz, J_2 = 2.3 Hz, 1H), 7.21 – 7.10 (m, 2H), 7.02 – 6.90 (m, 3H), 4.12 – 4.04 (m, 1H), 4.03 – 3.96 (m, 2H), 3.93 (s, 3H), 2.63 – 2.50 (m, 1H), 2.40 (dd, J_1 = 12.2 Hz, J_2 = 3.8 Hz, 1H), 2.34 (s, 6H)

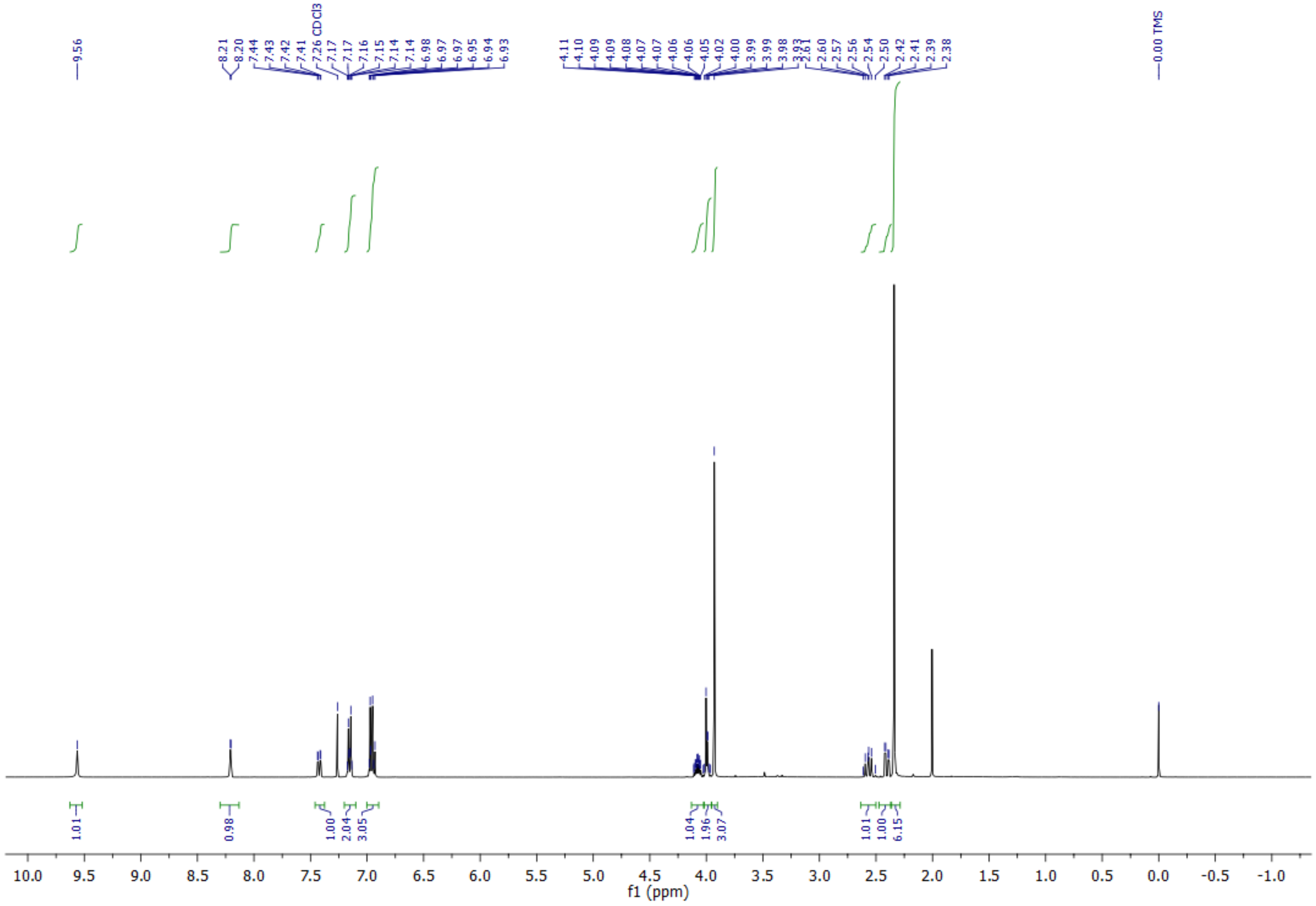

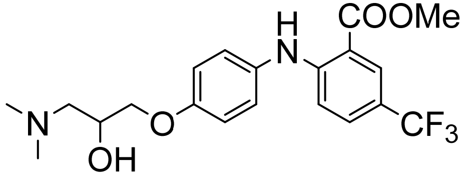

^{13}C NMR (101 MHz, $CDCl_3$) δ 168.32, 156.81, 151.90, 132.36, 130.66 (q, *J* = 3.2 Hz), 129.40 (q, *J* = 3.9 Hz), 126.68, 124.48 (q, *J* = 270.6 Hz), 117.87 (q, *J* = 33.2 Hz), 115.66, 113.40, 109.92, 70.84, 66.31, 61.92, 52.11, 45.67; HRMS (ESI$^+$) $C_{20}H_{24}F_3N_2O_4$ ([M+H]$^+$) calculated 413.1683 found 413.1672

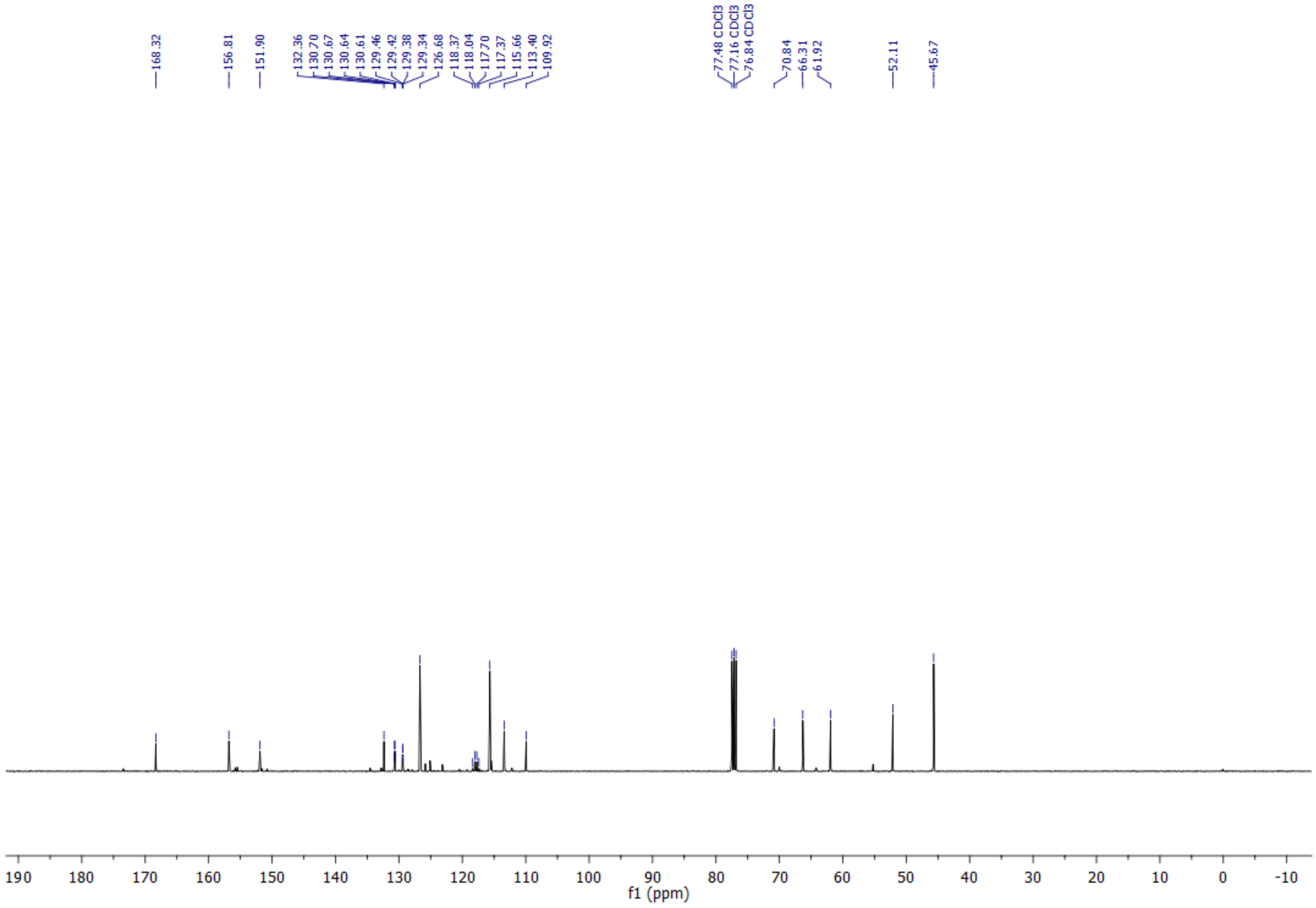

1-(Dimethylamino)-3-(4-((2,4-dinitrophenyl)amino)phenoxy)propan-2-ol (6d)

^{1}H NMR (400 MHz, DMSO) δ 10.09 (s, 1H), 8.89 (d, *J* = 2.7 Hz, 1H), 8.20 (dd, J_1 = 9.6 Hz, J_2 = 2.8 Hz, 1H), 7.33 – 7.22 (m, 2H), 7.12 – 7.04 (m, 2H), 6.97 (d, *J* = 9.6 Hz, 1H), 4.88 (d, *J* = 3.8 Hz, 1H), 4.02 (dd, J_1 = 9.0 Hz, J_2 = 2.9 Hz, 1H), 3.97 – 3.86 (m, 2H), 2.46 – 2.27 (m, 2H), 2.21 (s, 6H)

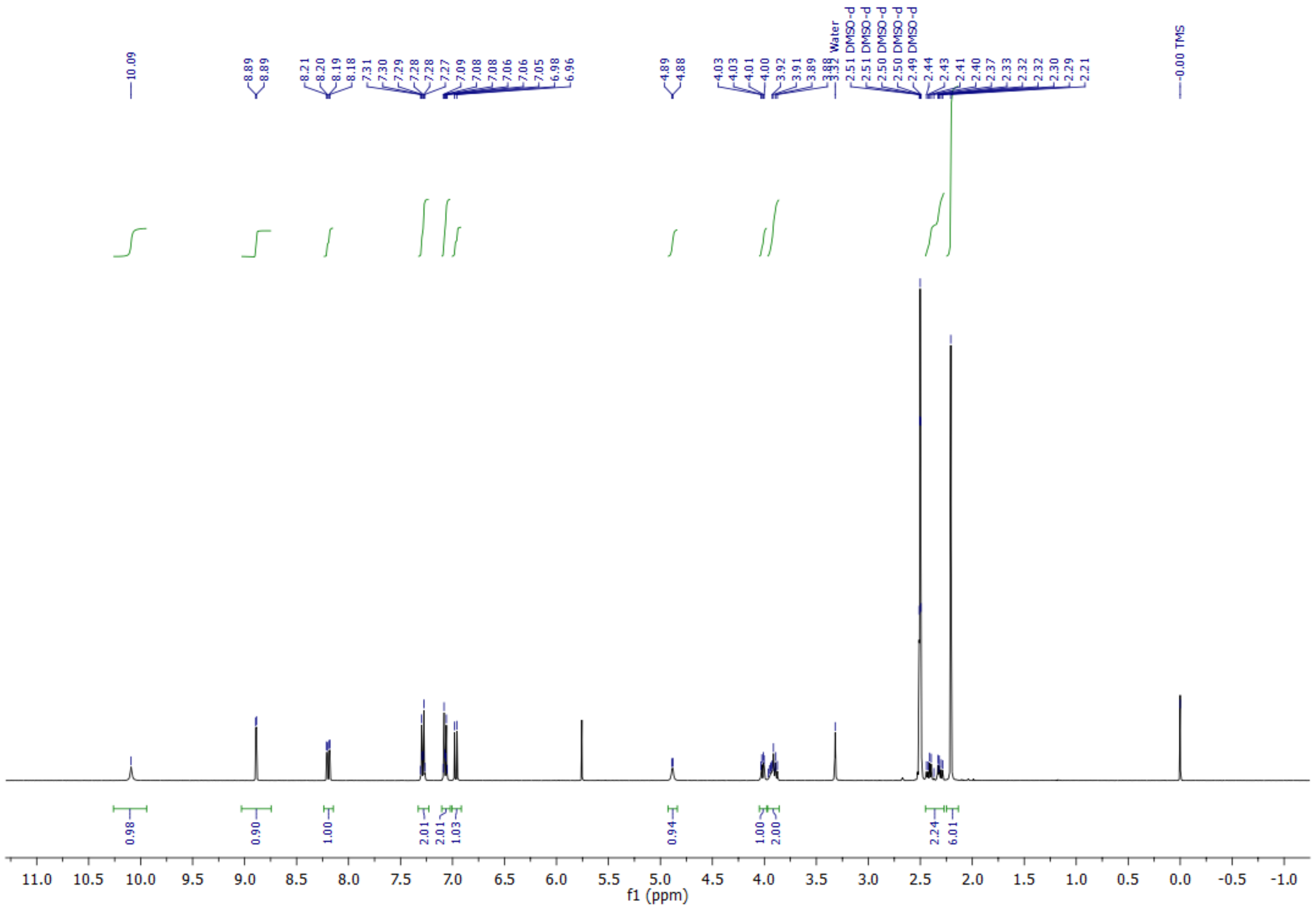

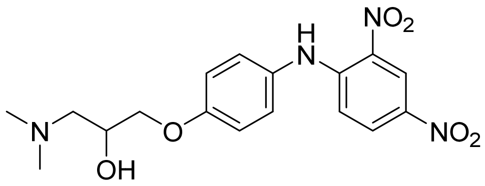

^{13}C NMR (101 MHz, $CDCl_3$) δ 158.39, 148.03, 137.16, 130.80, 129.97, 129.51, 127.53, 124.19, 116.16, 116.11, 70.86, 66.15, 61.78, 45.65. HRMS (ESI$^+$) for $C_{17}H_{21}N_4O_6$ ([M+H]$^+$) calculated 377.1456 found 377.1447

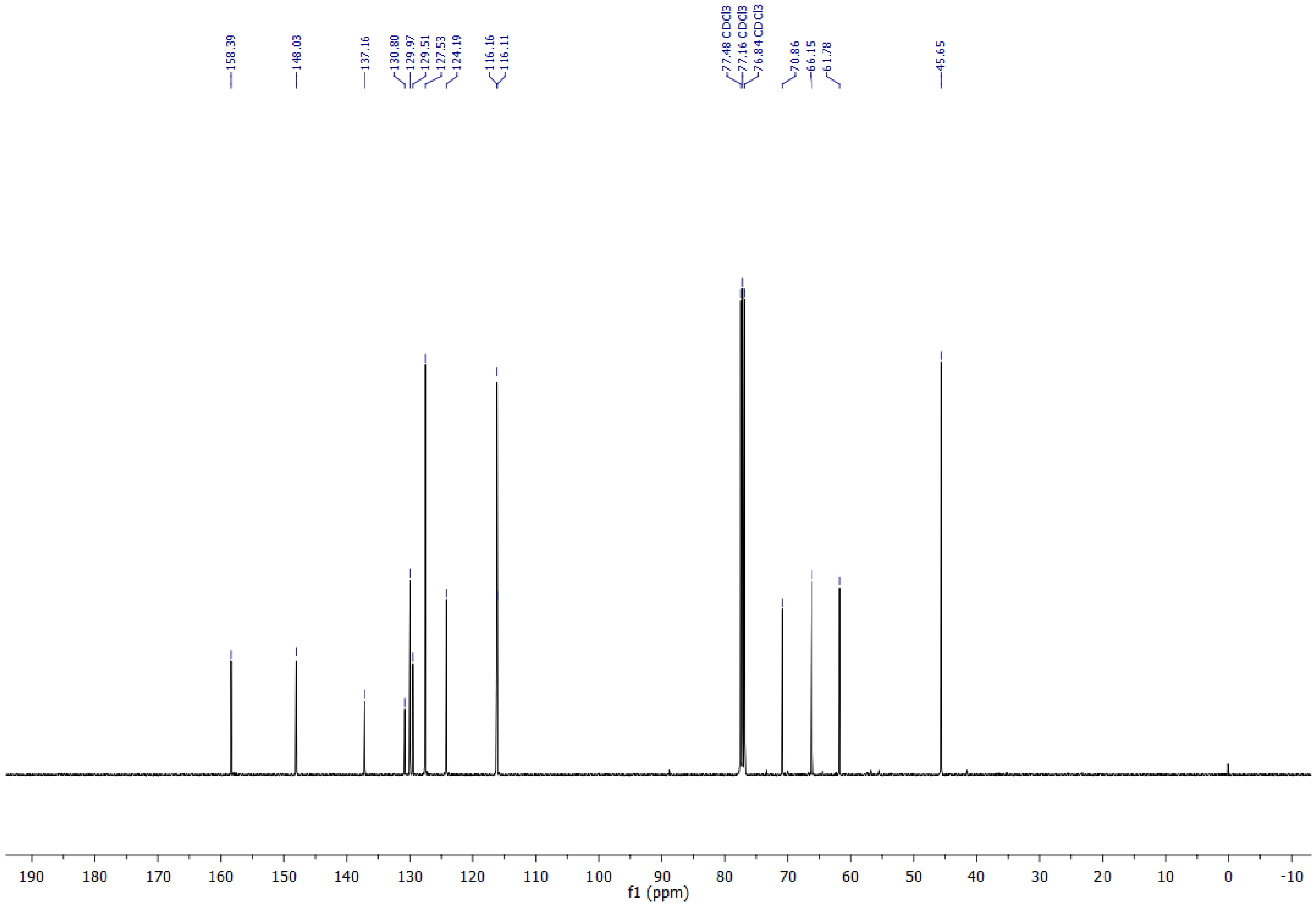

N-(4-hydroxyphenyl)-2-nitro-4-(trifluoromethyl)benzamide (7)

^{1}H NMR (400 MHz, DMSO) δ 10.52 (s, 1H), 9.34 (s, 1H), 8.49 (d, *J* = 0.8 Hz, 1H), 8.26 (dd, J_1 = 8.0 Hz, J_2 = 1.1 Hz, 1H), 8.00 (d, *J* = 7.9 Hz, 1H), 7.49 – 7.36 (m, 2H), 6.83 – 6.68 (m, 2H)

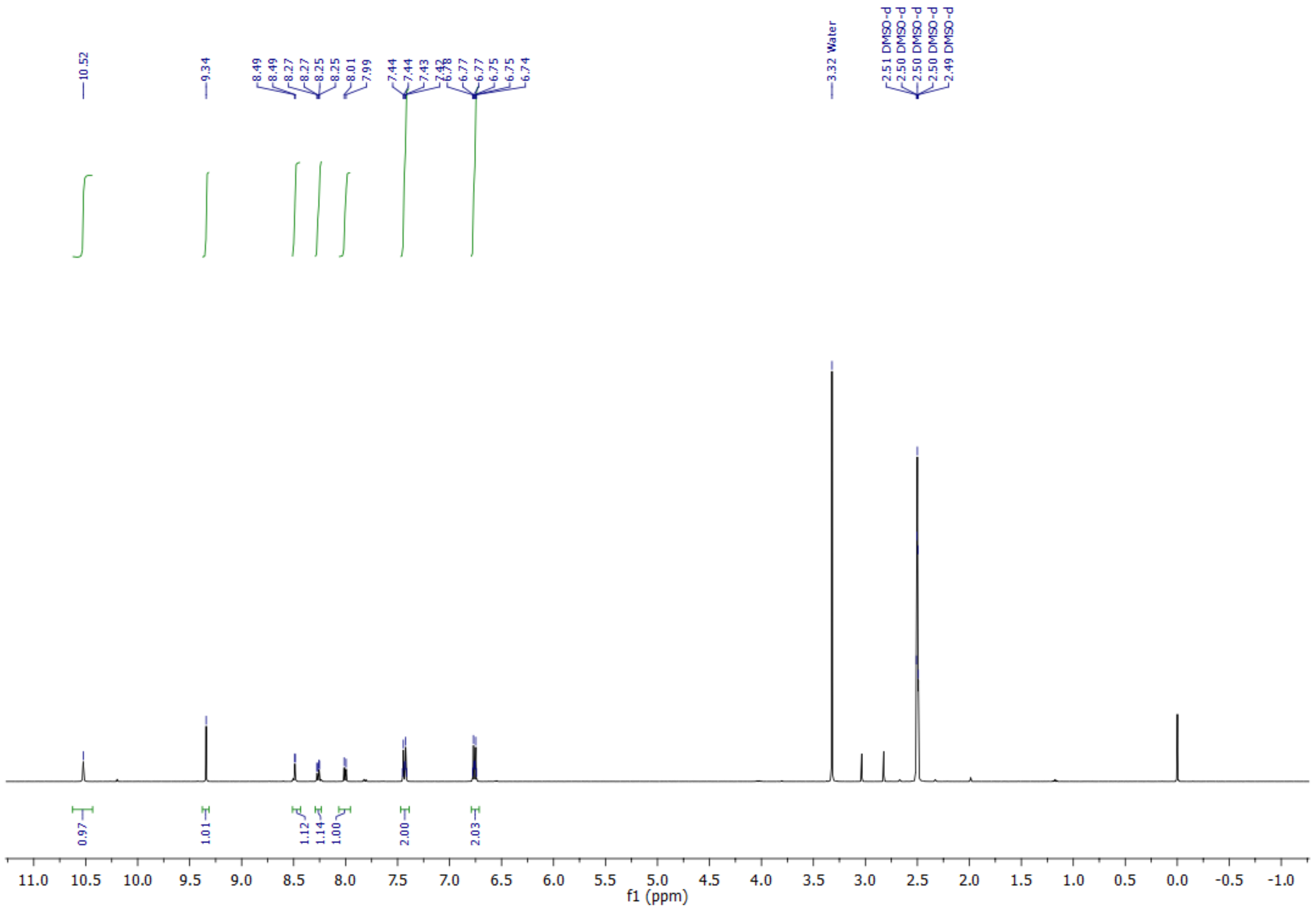

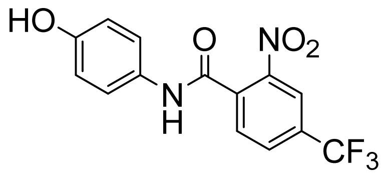

2-((4-Hydroxyphenyl)amino)benzoic acid (8a)

^{1}H NMR (400 MHz, DMSO) δ 12.90 (s, 1H), 9.42 (brs, 1H), 9.34 (brs, 1H), 7.84 (dd, J_1 = 8.0 Hz, J_2 = 1.6 Hz, 1H), 7.30 (ddd, J_1 = 8.7 Hz, J_2 = 7.1 Hz, J_3 = 1.7 Hz, 1H), 7.08 – 7.02 (m, 2H), 6.86 (dd, J_1 = 8.5 Hz, J_2 = 0.7 Hz, 1H), 6.83 – 6.77 (m, 2H), 6.69 – 6.62 (m, 1H)

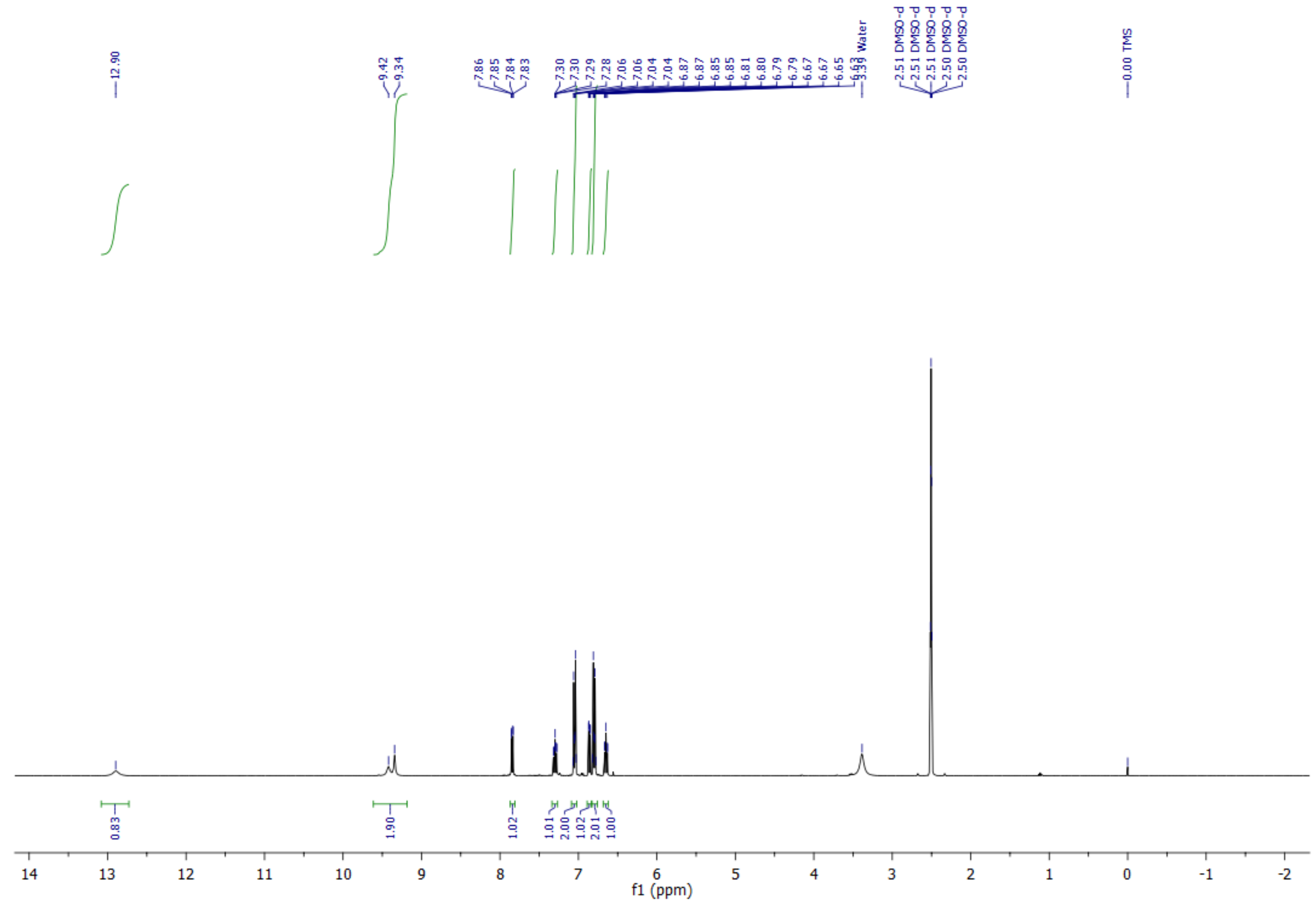

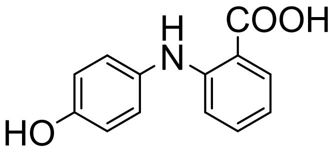

2-((4-Hydroxyphenyl)amino)-5-(trifluoromethyl)benzoic acid (8b)

^{1}H NMR (400 MHz, DMSO) δ 9.69 (s, 1H), 9.54 (brs, 1H), 8.08 (d, *J* = 1.7 Hz, 1H), 7.58 (dd, J_1 = 9.0 Hz, J_2 = 2.2 Hz, 1H), 7.14 – 7.06 (m, 2H), 6.91 (d, *J* = 8.9 Hz, 1H), 6.86 – 6.79 (m, 2H)

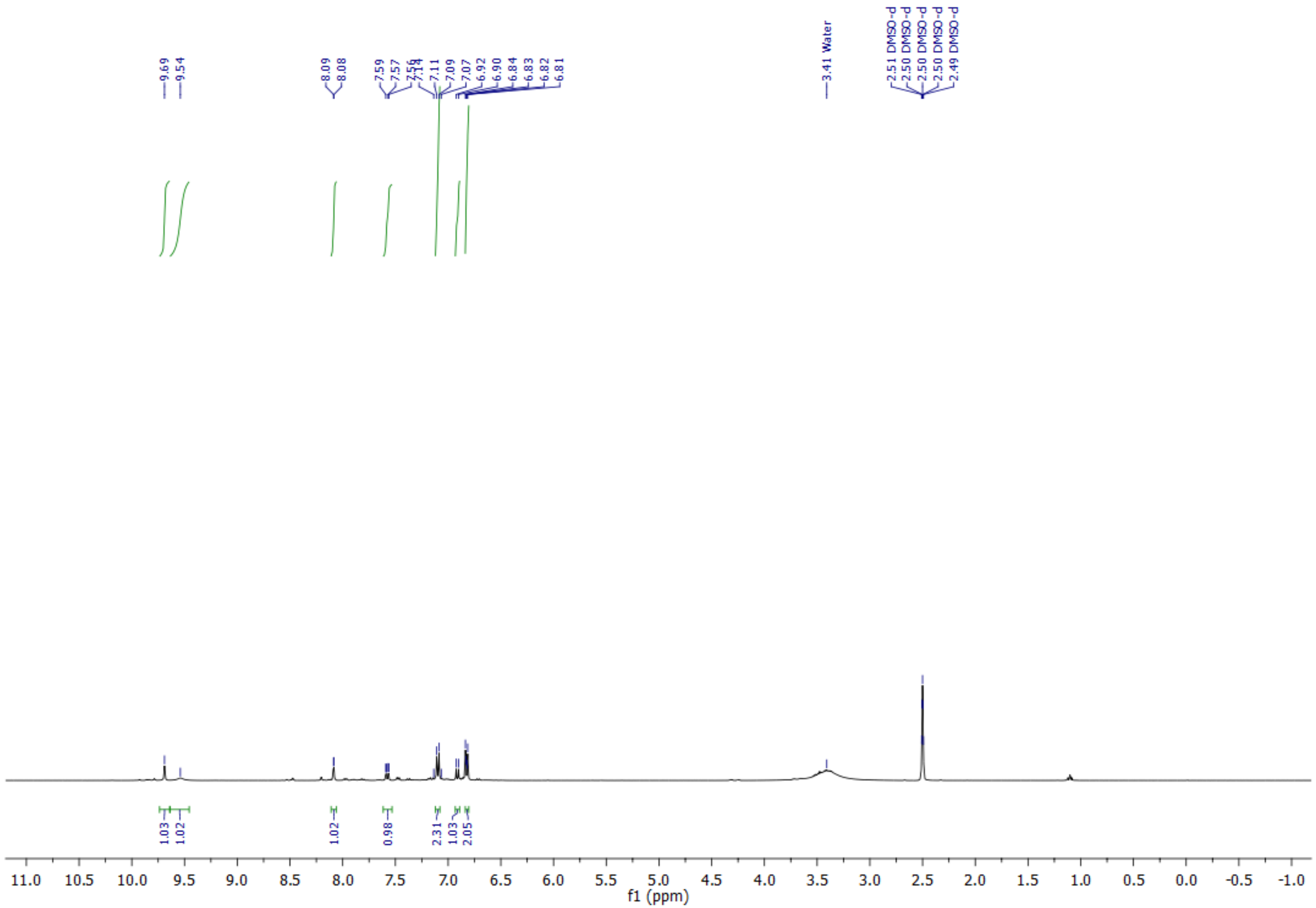

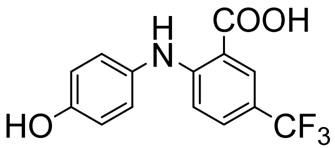

Methyl 2-((4-hydroxyphenyl)amino)benzoate (9a)

^1H NMR (400 MHz, DMSO) δ 9.38 (s, 1H), 9.08 (s, 1H), 7.85 (dd, J_1 = 8.0 Hz, J_2 = 1.6 Hz, 1H), 7.32 (ddd, J_1 = 8.6 Hz, J_2 = 7.2 Hz, J_3 = 1.6 Hz, 1H), 7.09 – 7.02 (m, 2H), 6.86 (dd, J_1 = 8.6 Hz, J_2 = 0.8 Hz, 1H), 6.83 – 6.76 (m, 2H), 6.67 (ddd, J = 8.1 Hz, J_2 = 7.1 Hz, J_3 = 1.1 Hz, 1H), 3.84 (s, 3H)

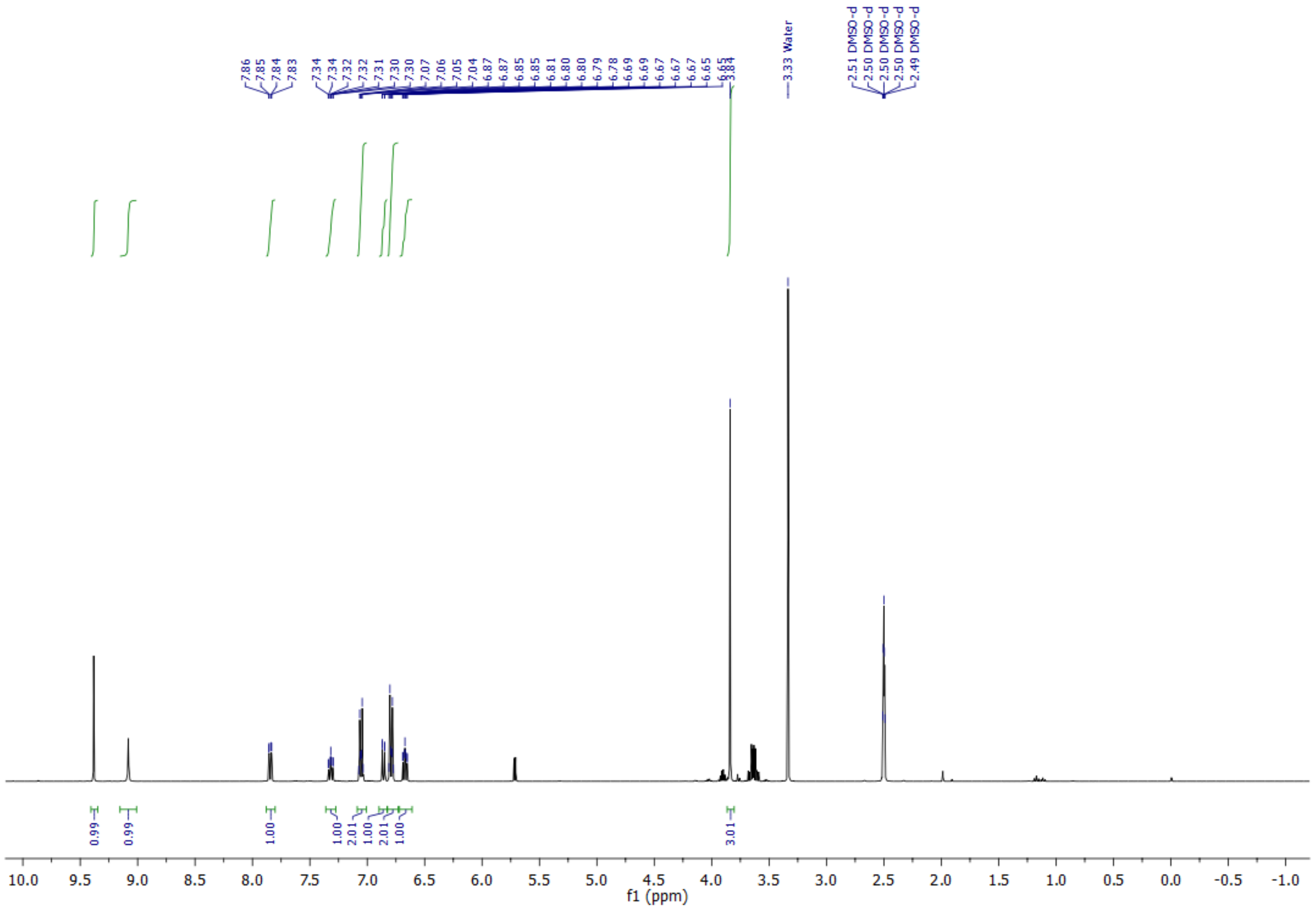

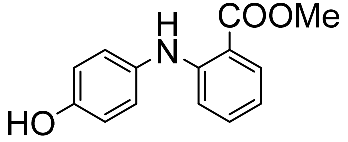

Methyl 2-((4-hydroxyphenyl)amino)-5-(trifluoromethyl)benzoate (9b)

^{1}H NMR (400 MHz, DMSO) δ 9.54 (s, 1H), 9.41 (s, 1H), 8.09 (d, *J* = 1.7 Hz, 1H), 7.61 (dd, J_1 = 9.1 Hz, J_2 = 2.2 Hz, 1H), 7.10 (d, *J* = 8.7 Hz, 2H), 6.91 (d, *J* = 9.0 Hz, 1H), 6.86 – 6.73 (m, 2H), 3.88 (s, 3H)

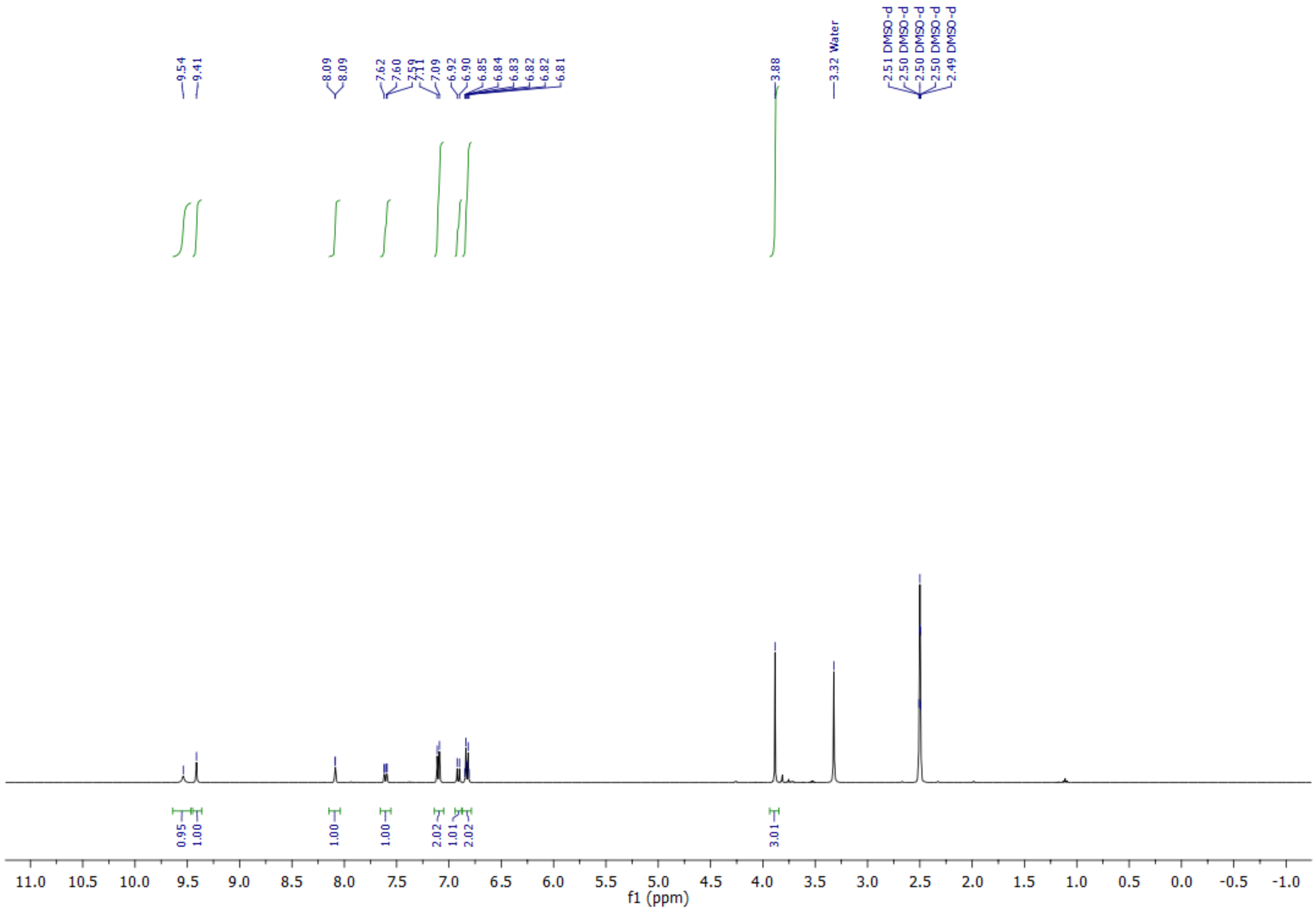

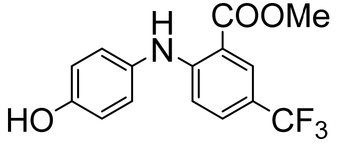

2-((4-(2-Hydroxy-3-morpholinopropoxy)phenyl)amino)benzoic acid (10a)

^{1}H NMR (400 MHz, DMSO) δ 11.50 (s, 1H), 7.90 (dd, J_1 = 7.7 Hz, J_2 = 1.6 Hz, 1H), 7.11 – 7.02 (m, 3H), 6.98 (d, J = 7.6 Hz, 1H), 6.88 (d, J = 8.9 Hz, 2H), 6.62 – 6.49 (m, 1H), 4.95 (brs, 1H), 4.01 – 3.89 (m, 2H), 3.88 – 3.75 (m, 1H), 3.56 (t, J = 4.6 Hz, 4H), 2.48 – 2.28 (m, 6H)

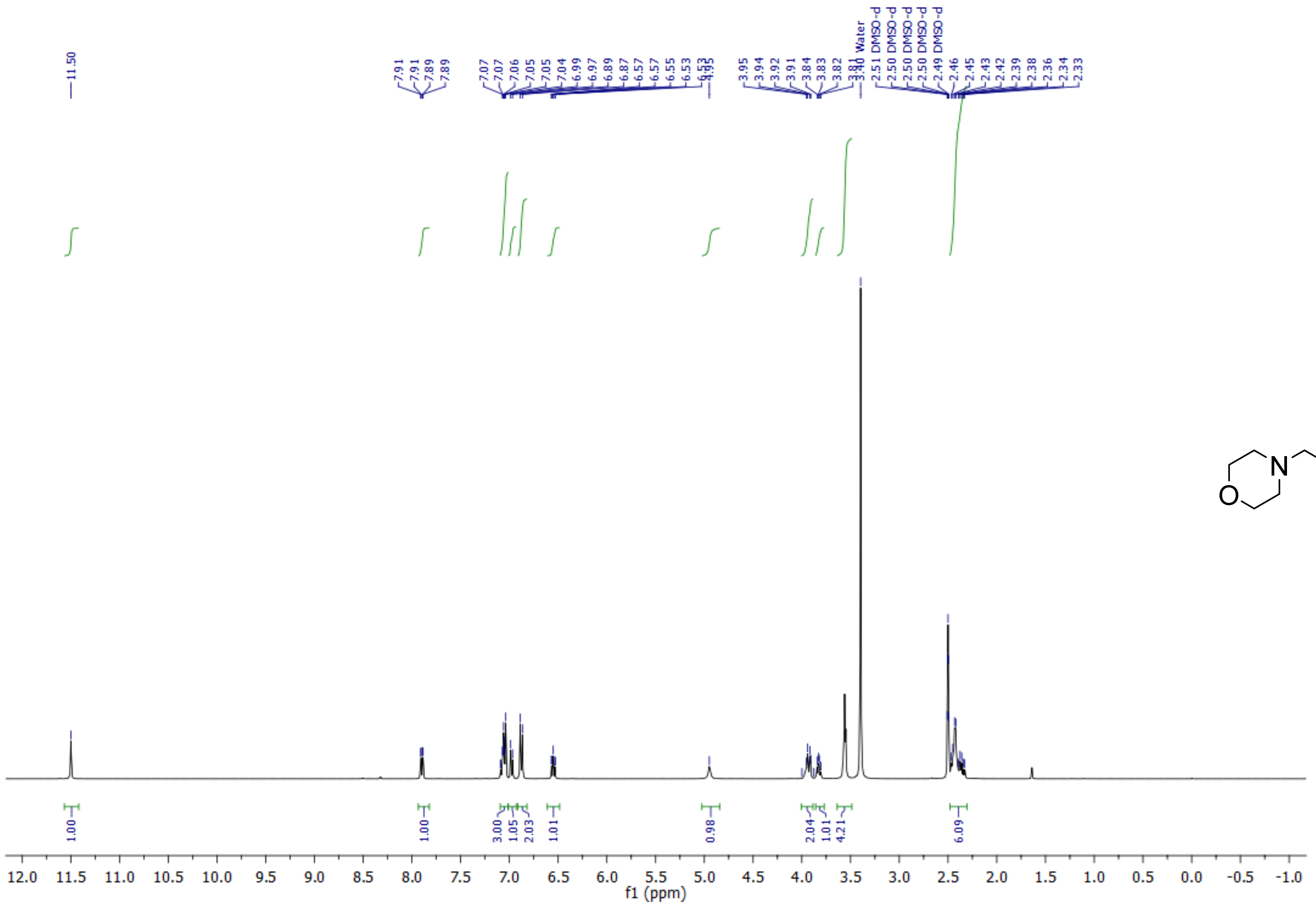

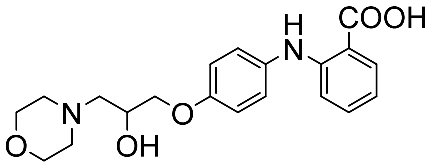

^{13}C NMR (101 MHz, DMSO) δ 171.88, 153.58, 146.66, 135.84, 132.10, 129.92, 122.22, 121.71, 115.62, 115.33, 111.85, 71.34, 66.45, 66.29, 61.60, 54.09; HRMS (ESI$^+$) for $C_{20}H_{25}N_2O_5$ ([M+H]$^+$) calculated 373.1758 found 373.1751

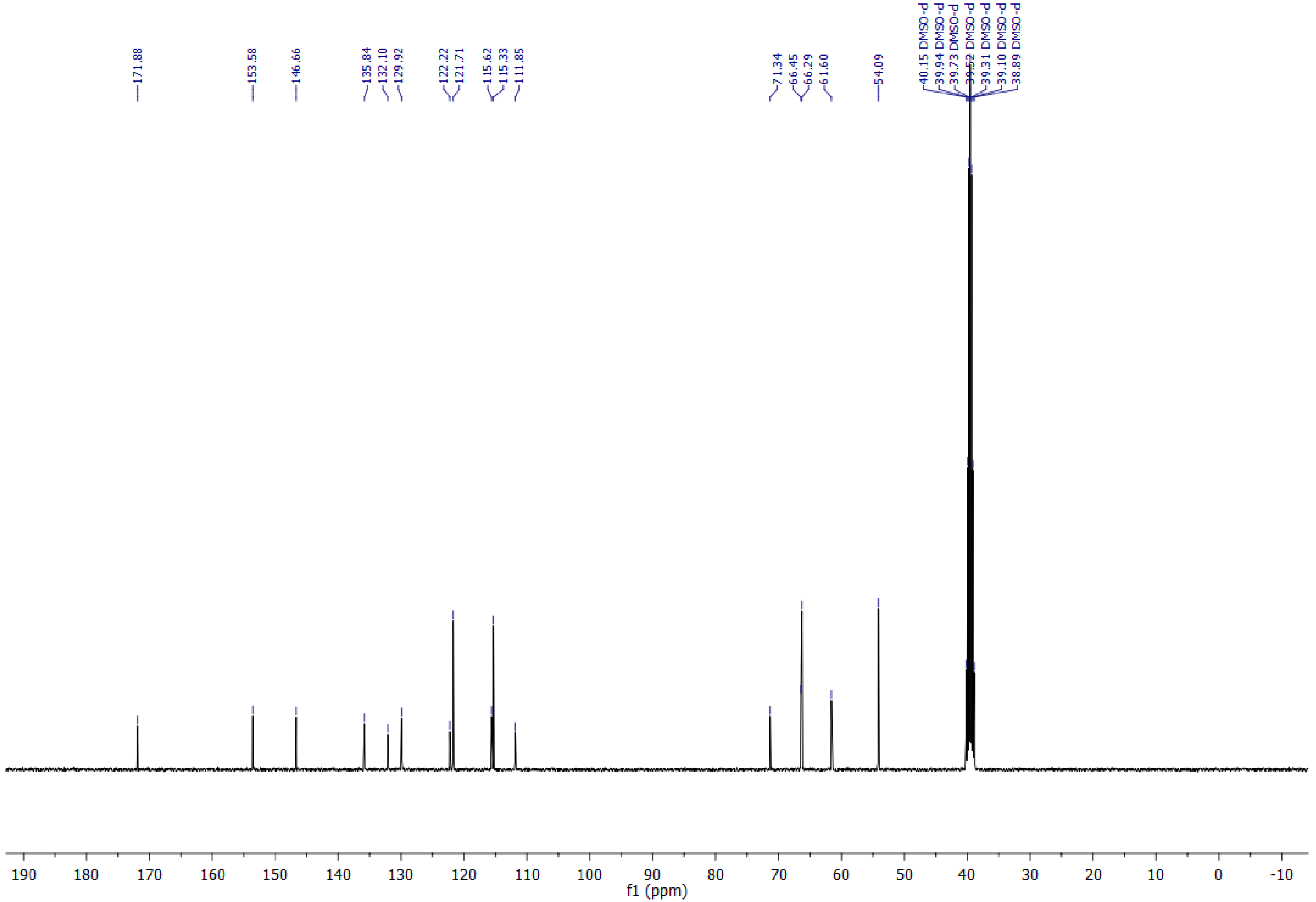

2-((4-(3-(Dimethylamino)-2-hydroxypropoxy)phenyl)amino)-5-(trifluoromethyl)benzoic acid (10b)

^{1}H NMR (400 MHz, DMSO) δ 12.10 (s, 1H), 8.16 (d, *J* = 2.2 Hz, 1H), 7.33 (dd, J_1 = 8.7 Hz, J_2 = 2.3 Hz, 1H), 7.10 (d, *J* = 8.8 Hz, 2H), 6.98 (d, *J* = 8.6 Hz, 1H), 6.93 (d, *J* = 8.9 Hz, 2H), 4.87 (brs, 1H), 4.00 – 3.77 (m, 3H), 2.44 – 2.24 (m, 2H), 2.18 (s, 6H)

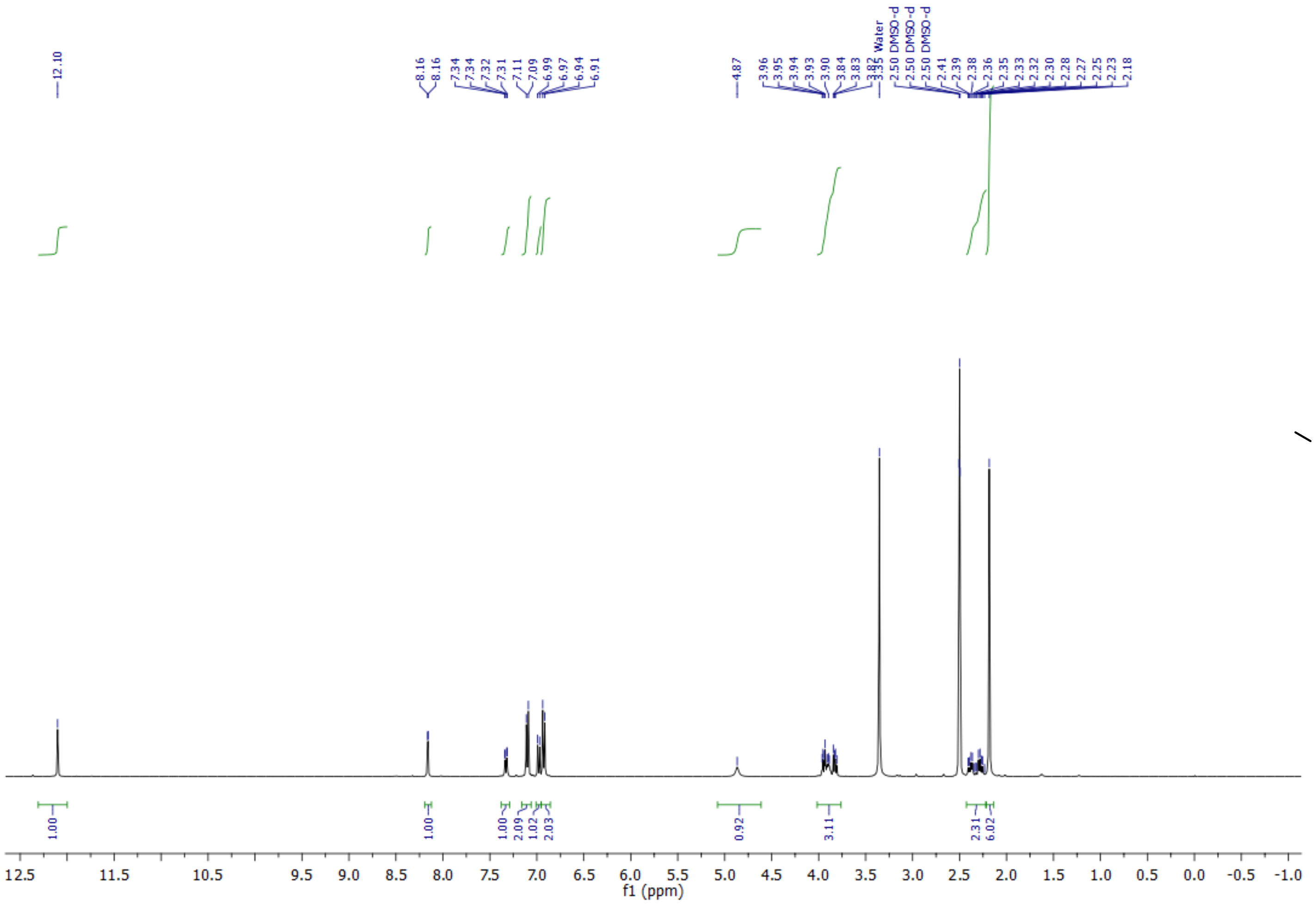

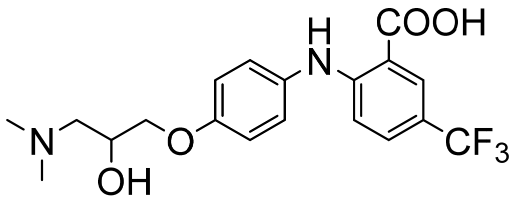

^{13}C NMR (101 MHz, MeOD) δ 174.97, 157.11, 151.89, 135.41, 130.67 (q, *J* = 3.7 Hz), 128.77 (q, *J* = 3.5 Hz), 126.55 (q, *J* = 269.4 Hz), 125.95, 120.53, 118.11 (q, *J* = 32.4 Hz), 116.51, 112.99, 72.34, 68.80, 63.31, 46.21; HRMS (ESI$^+$) for $C_{19}H_{22}F_3N_2O_4$ ([M+H]$^+$) calculated 399.1526, found 399.1519

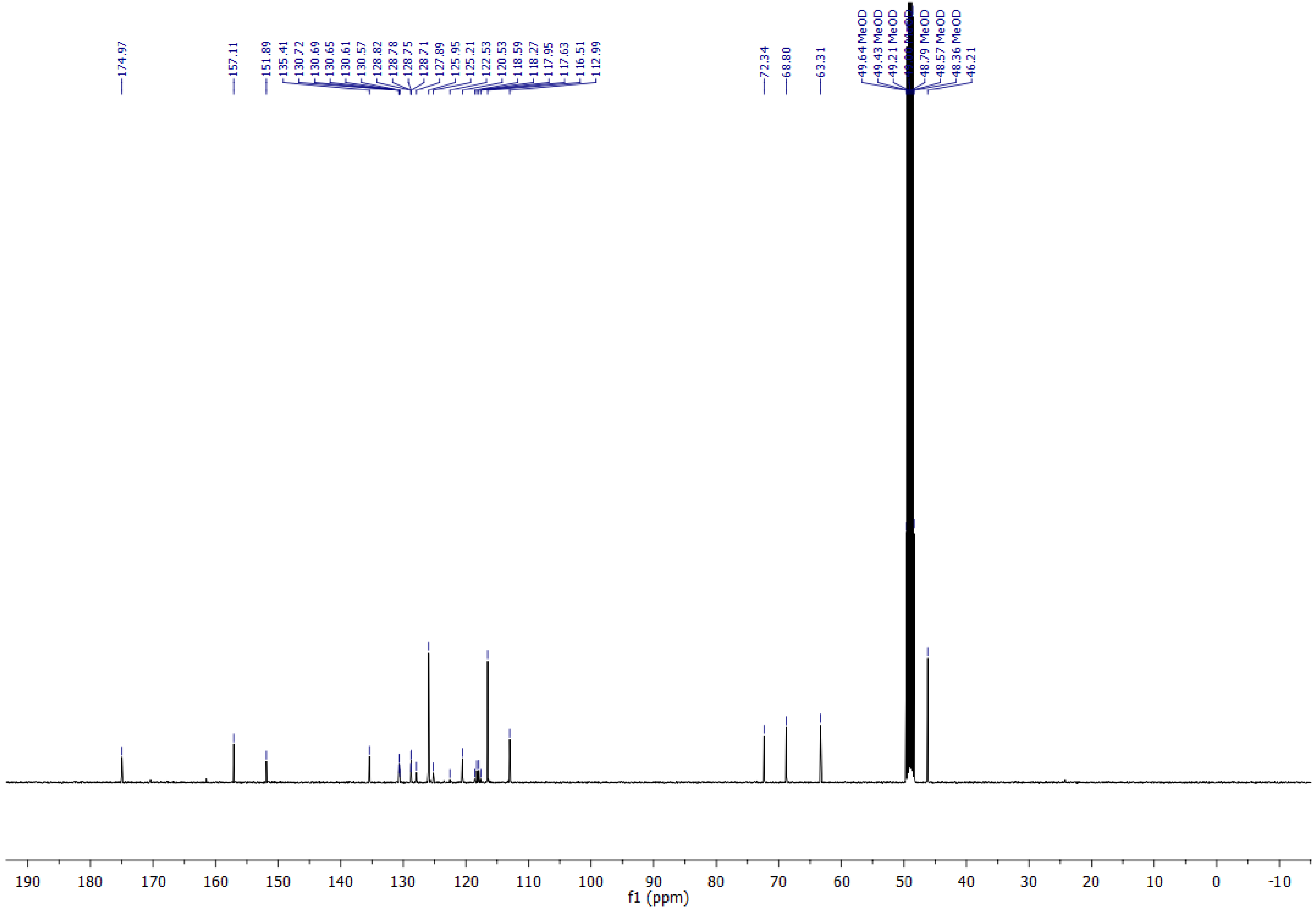

1-Chloro-2-nitro-4-(trifluoromethyl)benzene (11)

^{1}H NMR (400 MHz, $CDCl_3$) δ 8.16 (d, *J* = 1.8 Hz, 1H), 7.82 – 7.78 (m, 1H), 7.74 (d, *J* = 8.5 Hz, 1H)

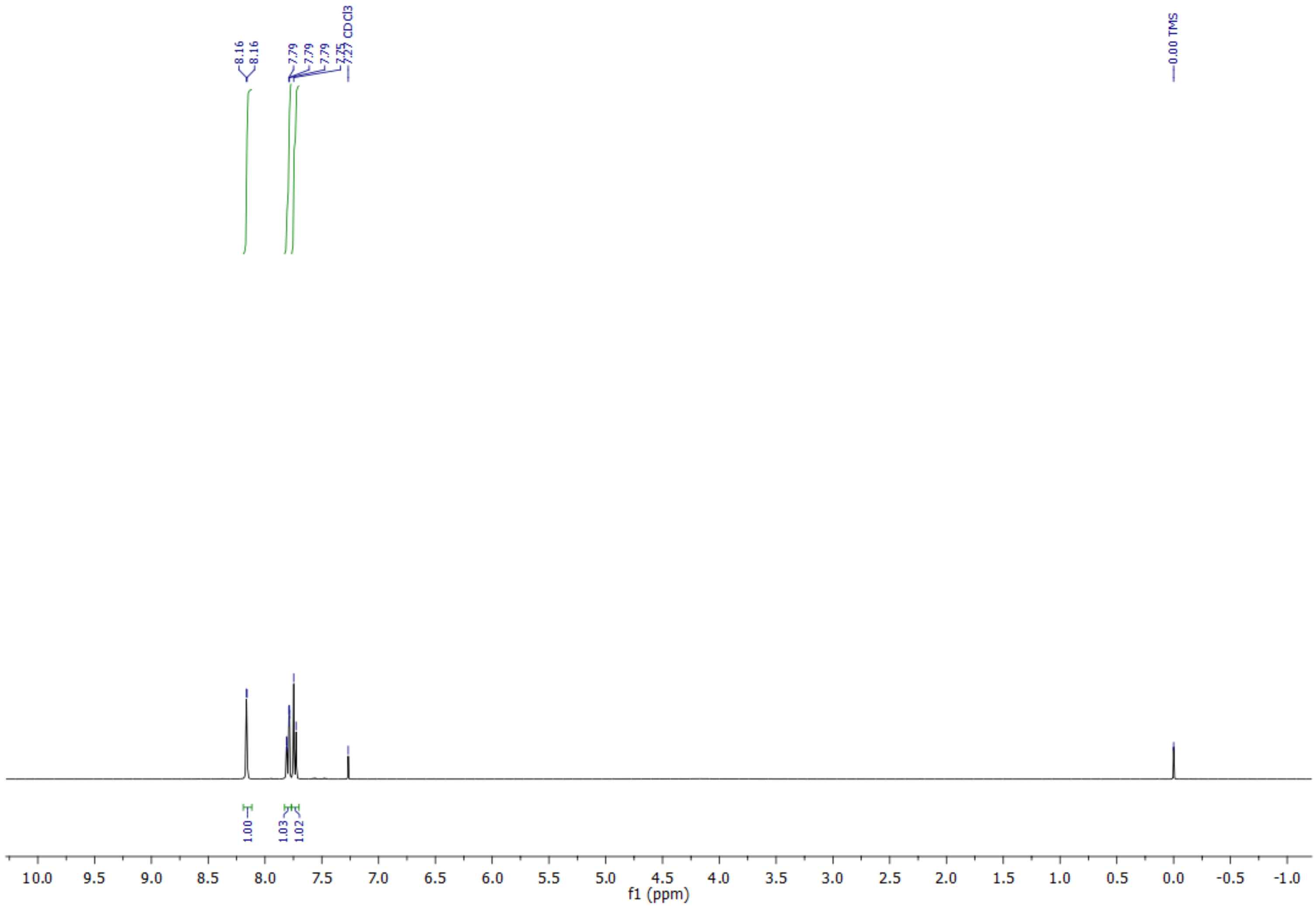

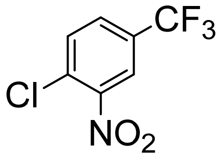

4-((2-Nitro-4-(trifluoromethyl)phenyl)amino)phenol (12a)

^{1}H NMR (400 MHz, $CDCl_3$) δ 9.58 (s, 1H), 8.50 (d, *J* = 1.0 Hz, 1H), 7.50 (dd, J_1 = 9.1 Hz, J_2 = 2.2 Hz, 1H), 7.20 – 7.10 (m, 2H), 7.04 (d, *J* = 9.1 Hz, 1H), 6.97 – 6.87 (m, 2H), 4.99 (s, 1H)

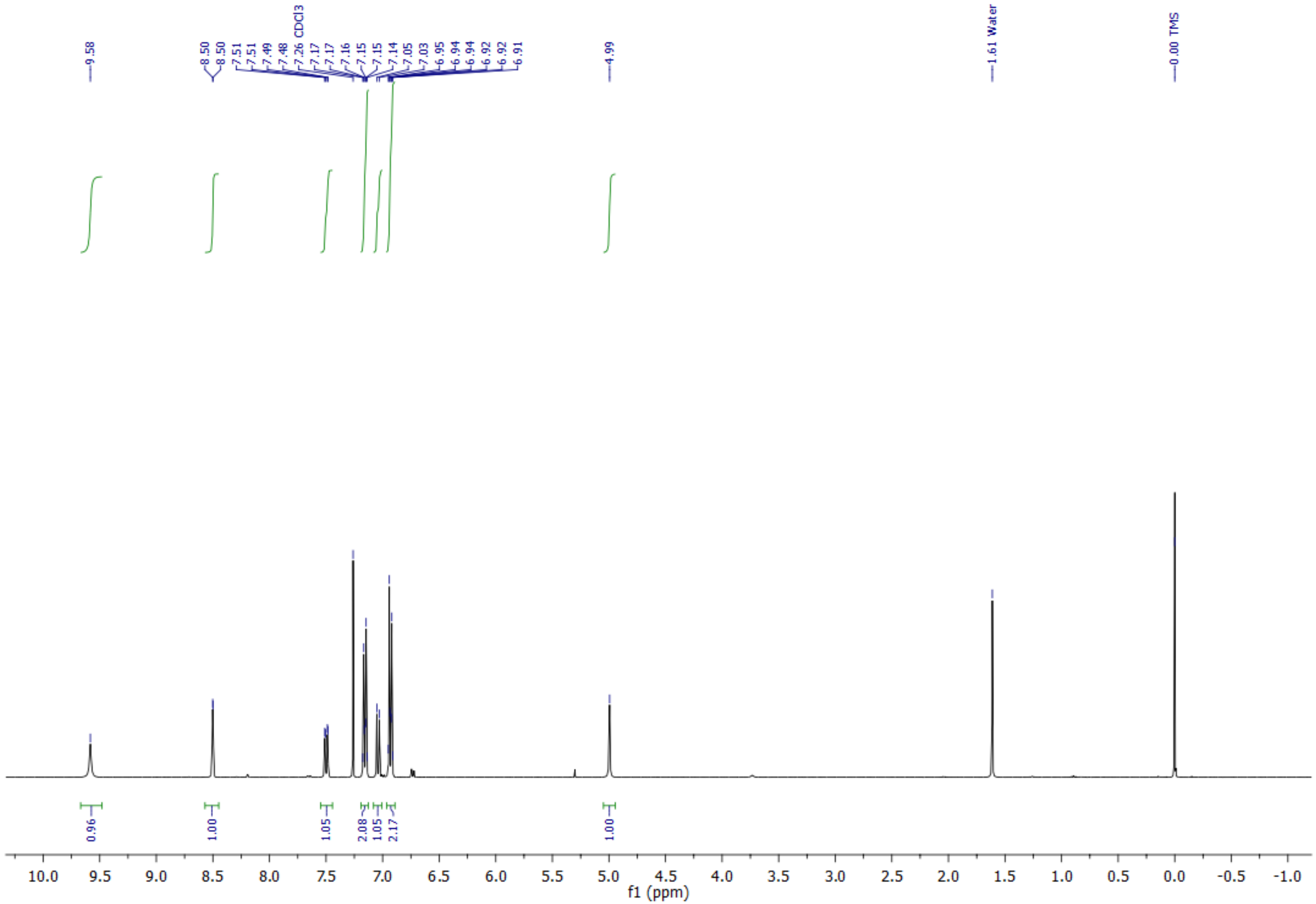

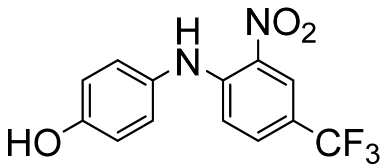

4-((2,4-Dinitrophenyl)amino)phenol (12b)

^{1}H NMR (400 MHz, DMSO) δ 10.04 (s, 1H), 9.73 (s, 1H), 8.86 (d, *J* = 2.7 Hz, 1H), 8.17 (dd, J_1 = 9.6 Hz, J_2 = 2.8 Hz, 1H), 7.21 – 7.08 (m, 2H), 6.94 (d, *J* = 9.6 Hz, 1H), 6.91 – 6.79 (m, 2H)

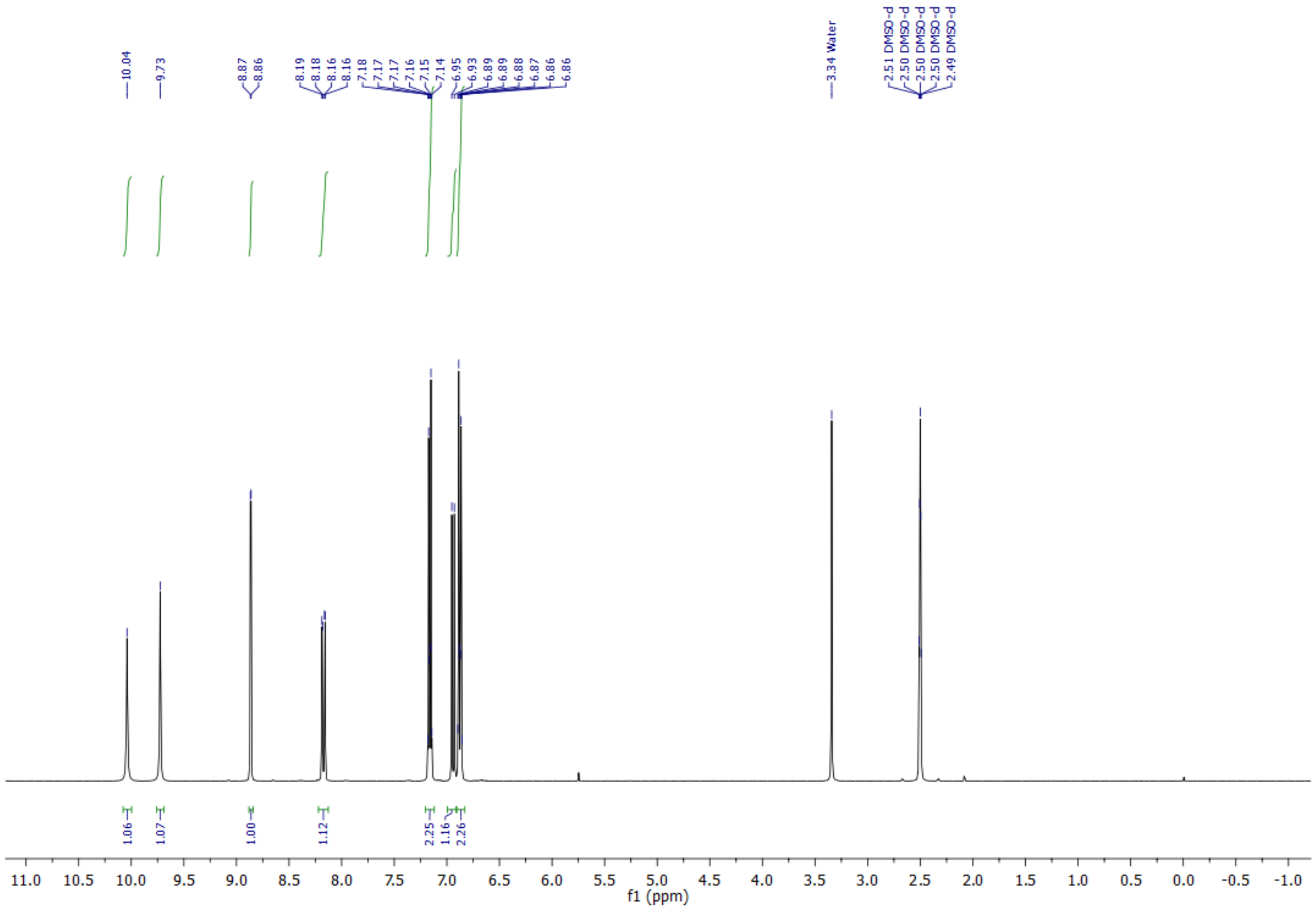

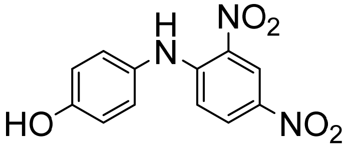

2,6-Dibromo-4-((2-nitro-4-(trifluoromethyl)phenyl)amino)phenol (12c)

^{1}H NMR (400 MHz, $CDCl_3$) δ 9.51 (s, 1H), 8.56 – 8.45 (m, 1H), 7.59 (dd, J_1 = 9.0, J_2 = 2.1 Hz, 1H), 7.49 – 7.41 (m, 2H), 7.08 (d, J = 9.0 Hz, 1H), 5.98 (s, 1H)

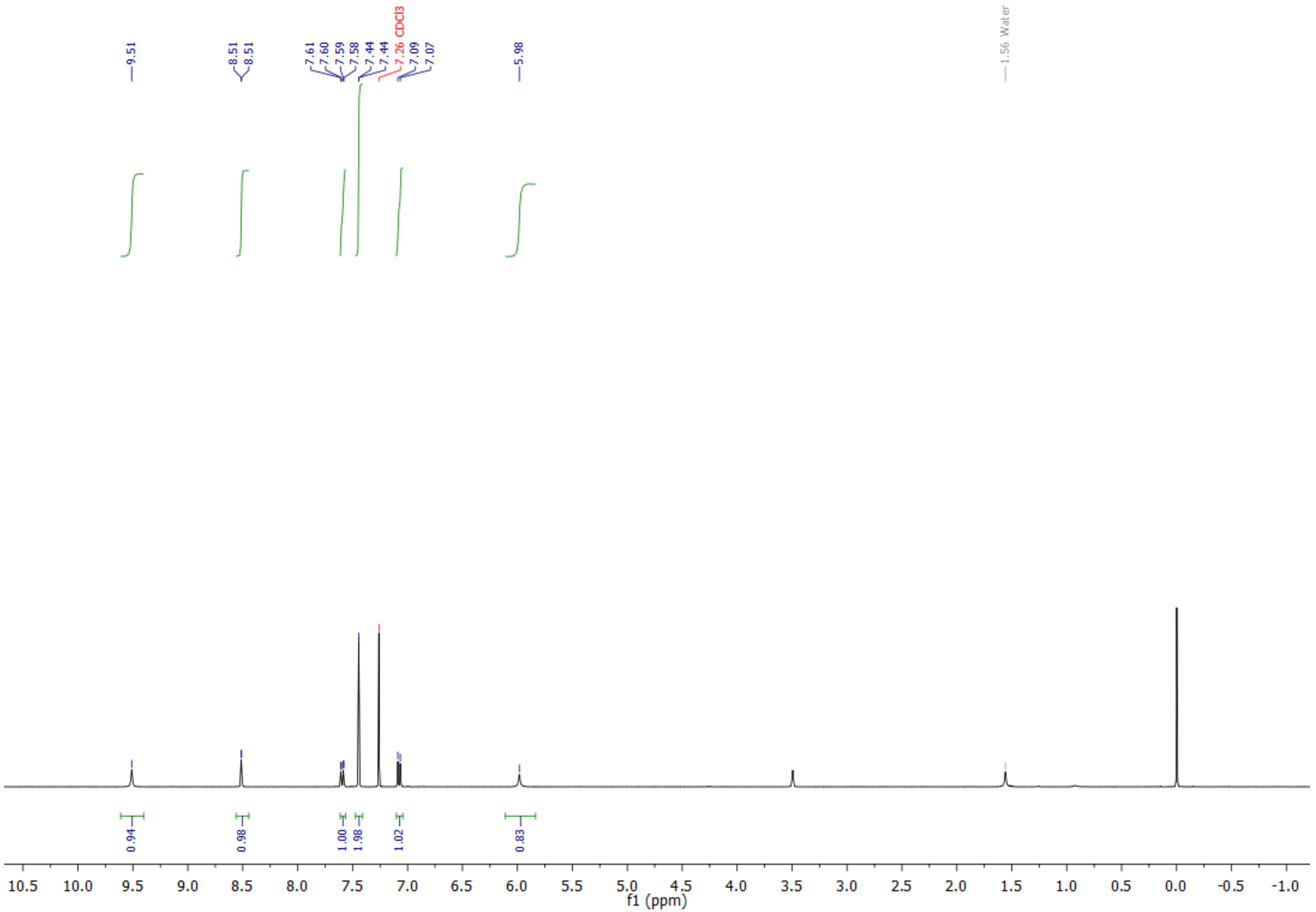

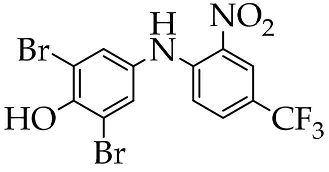

2,6-Dichloro-4-((2-nitro-4-(trifluoromethyl)phenyl)amino)phenol (12d)

^{1}H NMR (400 MHz, $CDCl_3$) δ 9.50 (s, 1H), 8.61 – 8.35 (m, 1H), 7.59 (dd, J_1 = 9.0 Hz, J_2 = 2.1 Hz, 1H), 7.26 (s, 2H), 7.09 (d, J = 9.0 Hz, 1H), 5.93 (s, 1H)

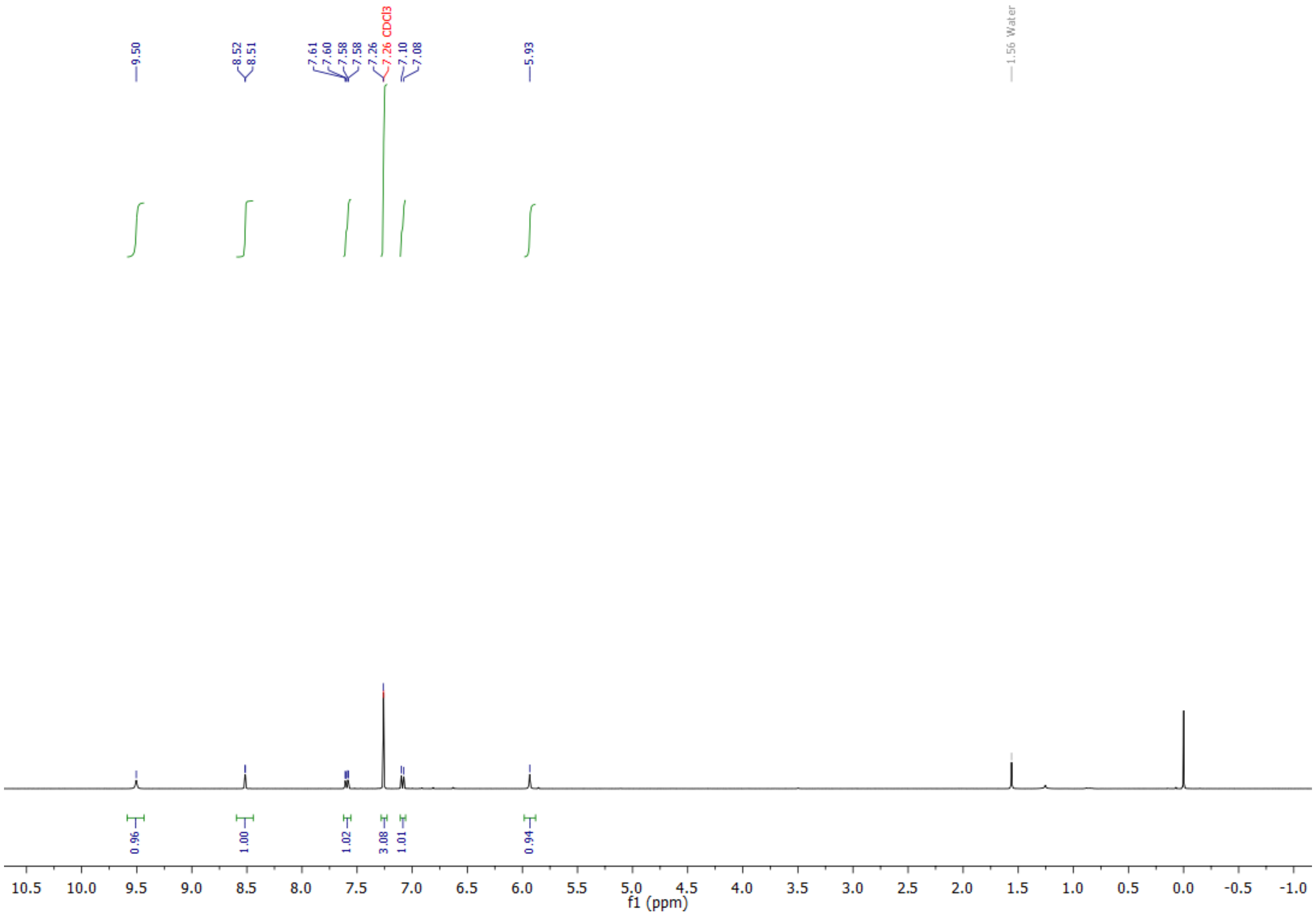

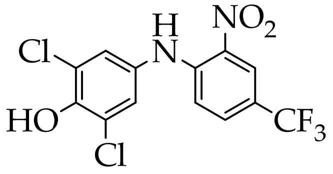

N-(4-(2-morpholinoethoxy)phenyl)-2-nitro-4-(trifluoromethyl)aniline (13)

^{1}H NMR (400 MHz, $CDCl_3$) δ 9.61 (s, 1H), 8.50 (d, J = 1.1 Hz, 1H), 7.49 (dd, J_1 = 9.1 Hz, J_2 = 2.1 Hz, 1H), 7.22 – 7.15 (m, 2H), 7.05 (d, J = 9.1 Hz, 1H), 7.02 – 6.96 (m, 2H), 4.15 (t, J = 5.7 Hz, 2H), 3.84 – 3.68 (m, 4H), 2.84 (t, J = 5.7 Hz, 2H), 2.66 – 2.53 (m, 4H)

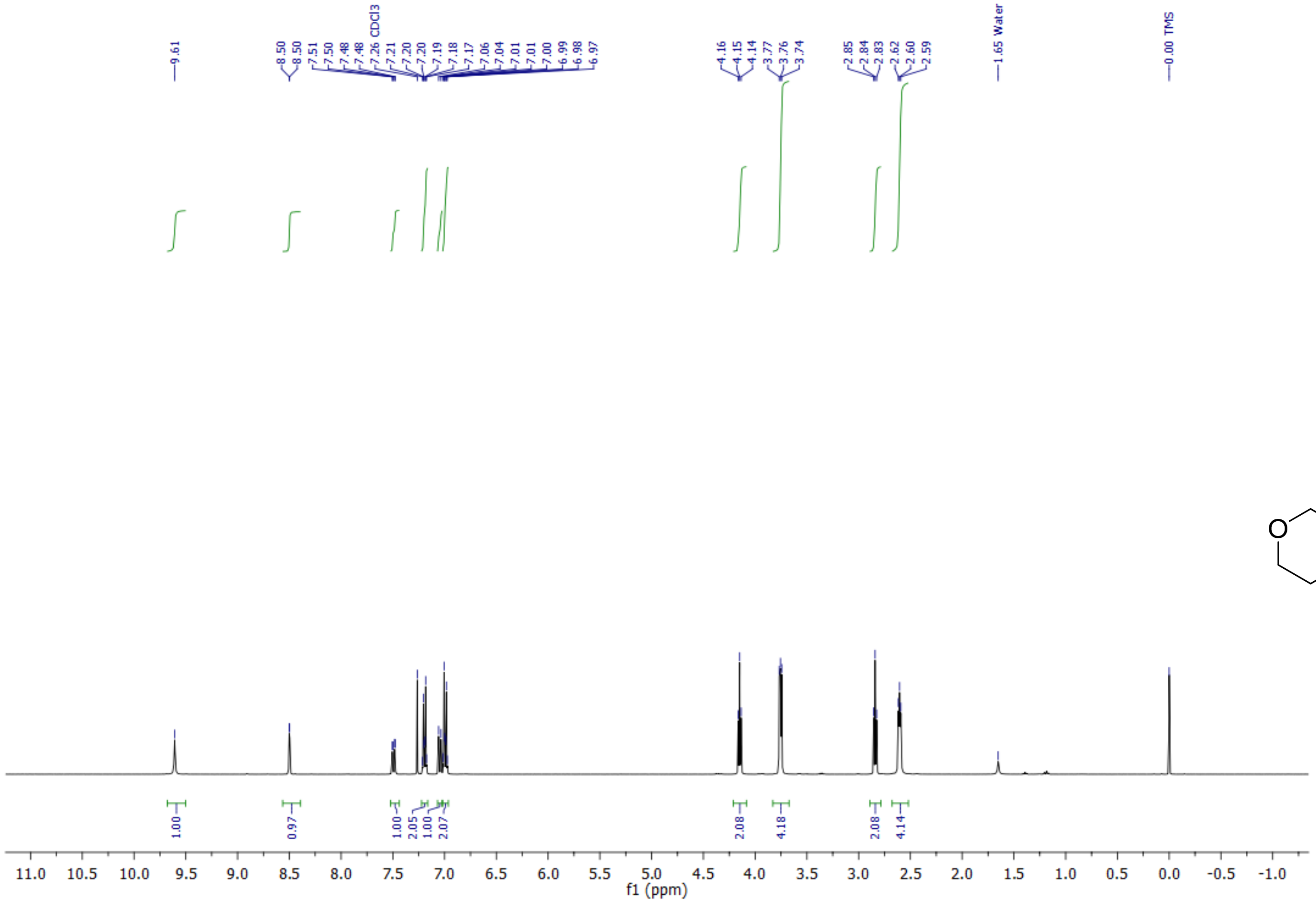

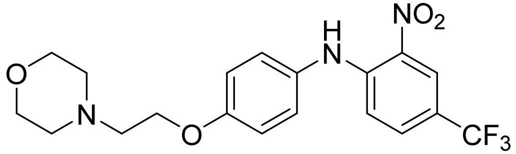

^{13}C NMR (101 MHz, $CDCl_3$) δ 157.90, 146.45, 131.84 (q, *J* = 3.0 Hz), 131.47, 130.32, 127.56, 124.88 (q, *J* = 4.5 Hz), 123.60 (q, *J* = 271.2 Hz), 118.96 (q, *J* = 34.4 Hz), 116.62, 116.03, 67.08, 66.34, 57.73, 54.27; HRMS (ESI$^+$) for $C_{19}H_{21}F_3N_3O_4$ ([M+H]$^+$) calculated 412.1479 found 412.1473

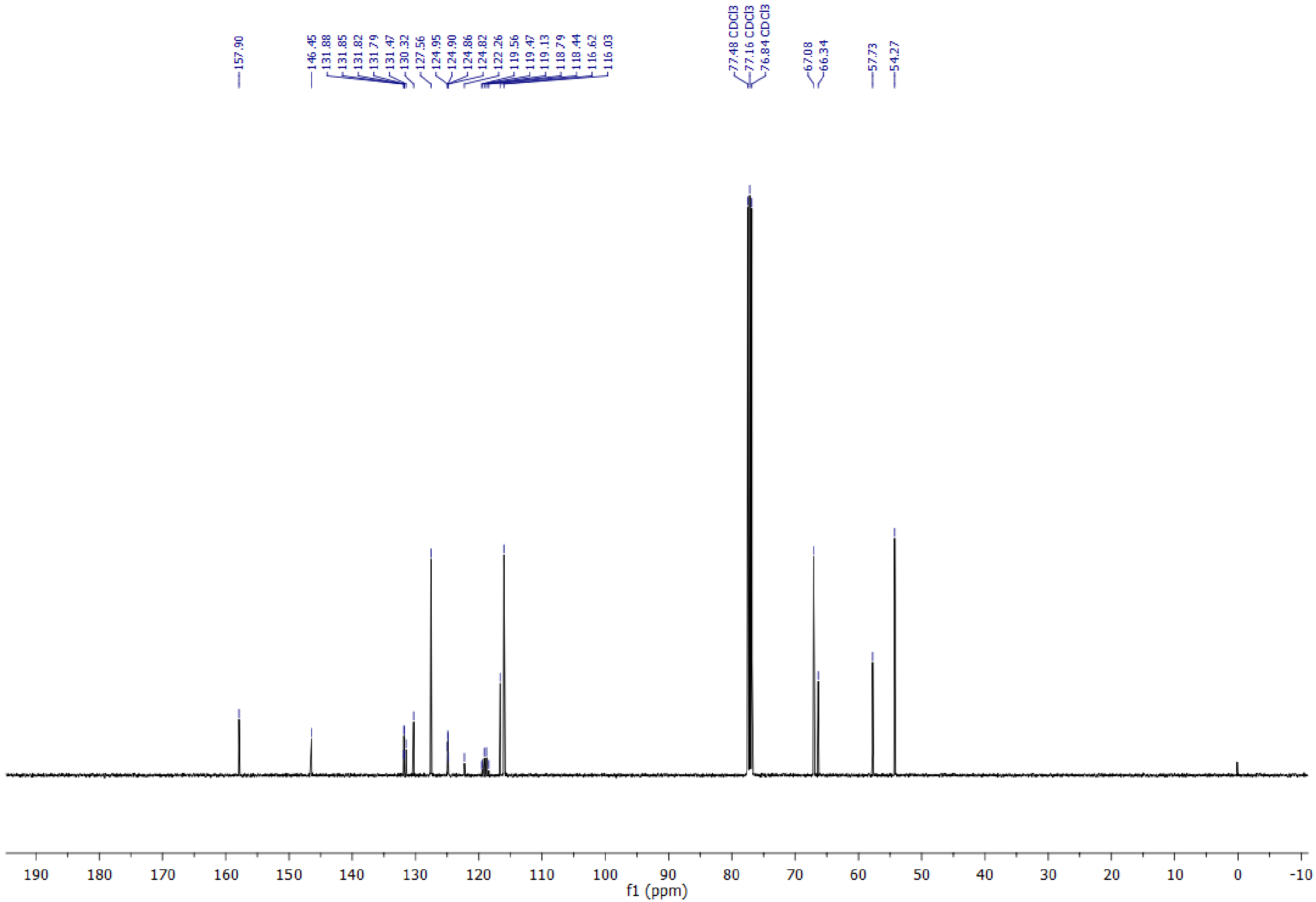

N-(4-(benzyloxy)phenyl)-2-nitro-4-(trifluoromethyl)aniline (14)

^{1}H NMR (400 MHz, $CDCl_3$) δ 9.61 (s, 1H), 8.50 (s, 1H), 7.53 – 7.32 (m, 6H), 7.19 (d, *J* = 8.8 Hz, 2H), 7.06 (d, *J* = 8.9 Hz, 3H), 5.11 (s, 2H)

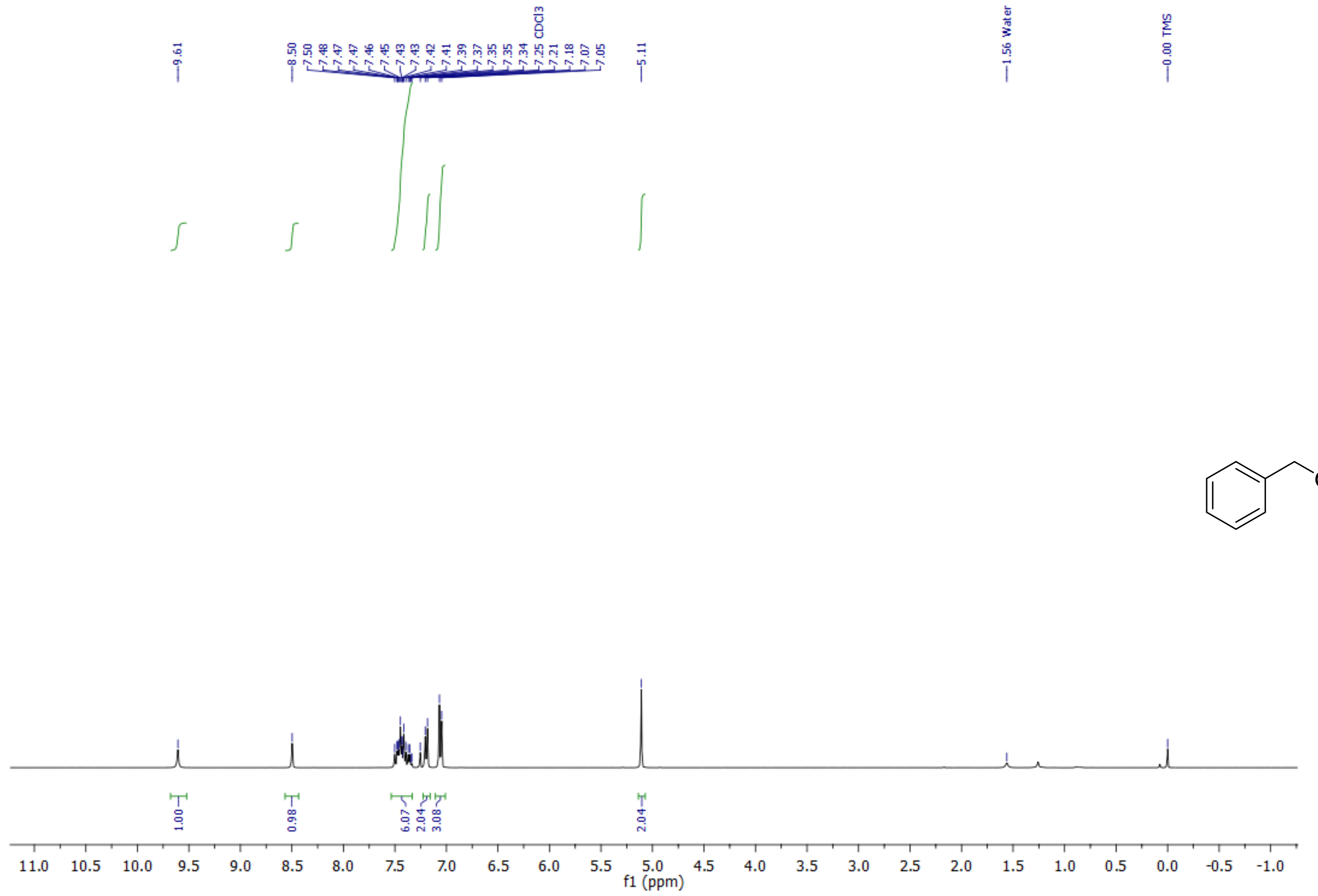

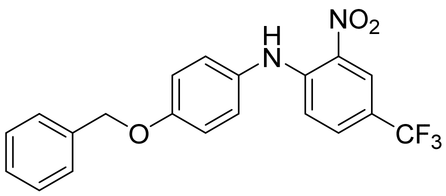

^{13}C NMR (101 MHz, $CDCl_3$) δ ^{13}C NMR (101 MHz, $CDCl_3$) δ 157.92, 146.43, 136.65, 131.83 (q, J = 3.0 Hz), 131.47, 130.38, 128.84, 128.34, 127.63, 127.56, 124.87 (q, J = 4.3 Hz), 123.65 (q, J = 268.0 Hz), 118.95 (q, J = 34.4 Hz), 116.65, 116.33, 70.50; HRMS (ESI$^-$) for $C_{20}H_{14}F_3N_2O_3$ ($[M]^-$) calculated 387.0962 found 387.0964

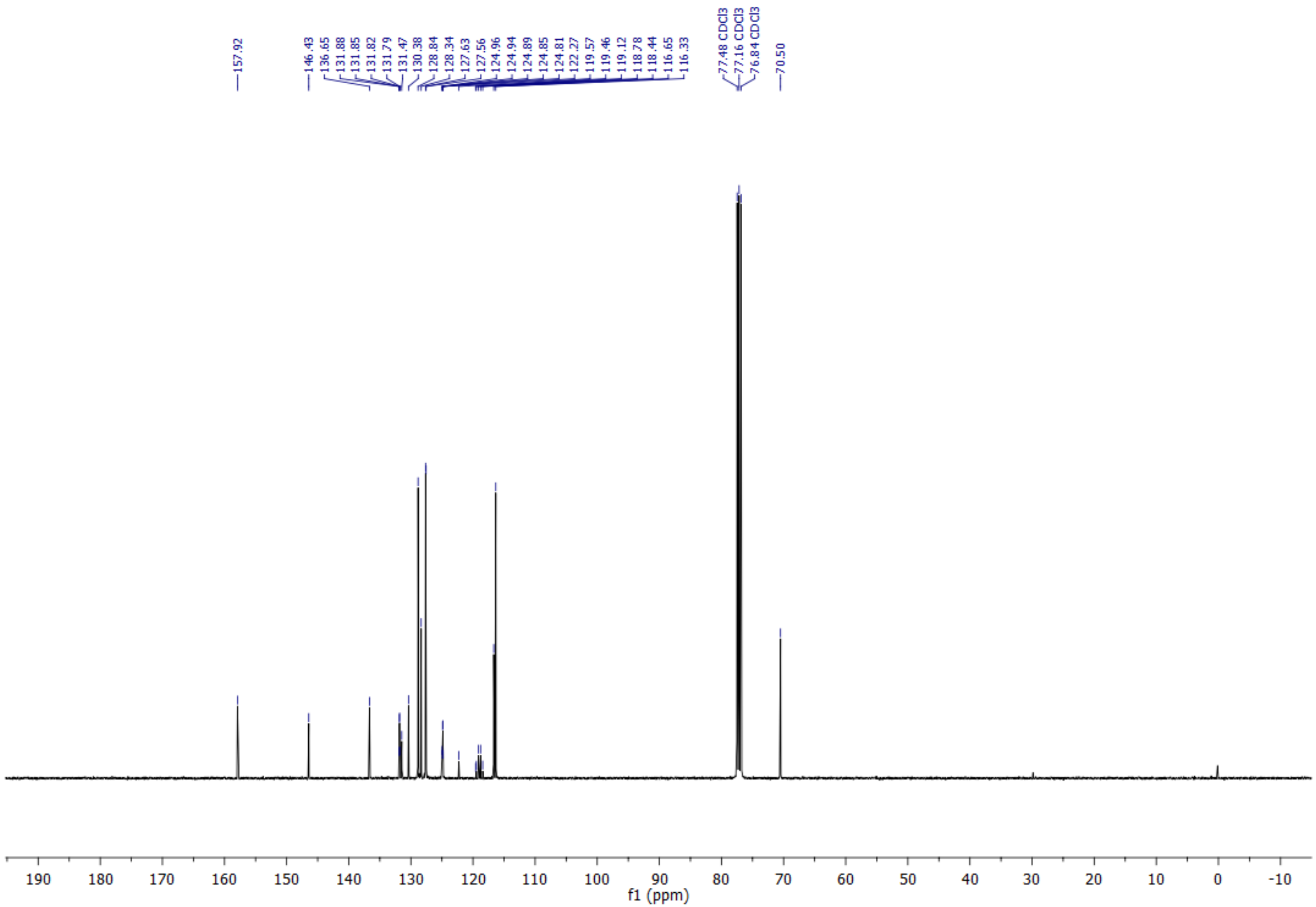

1-(Methylamino)-3-(4-((2-nitro-4-(trifluoromethyl)phenyl)amino)phenoxy)propan-2-ol (15)

^{1}H NMR (400 MHz, DMSO) δ 9.73 (s, 1H), 8.35 (d, *J* = 1.3 Hz, 1H), 7.72 (dd, J_1 = 9.2 Hz, J_2 = 2.2 Hz, 1H), 7.35 – 7.20 (m, 2H), 7.10 – 6.95 (m, 3H), 3.99 (q, *J* = 7.0 Hz, 1H), 3.91 (t, *J* = 5.9 Hz, 2H), 2.69 – 2.51 (m, 2H), 2.31 (s, 3H)

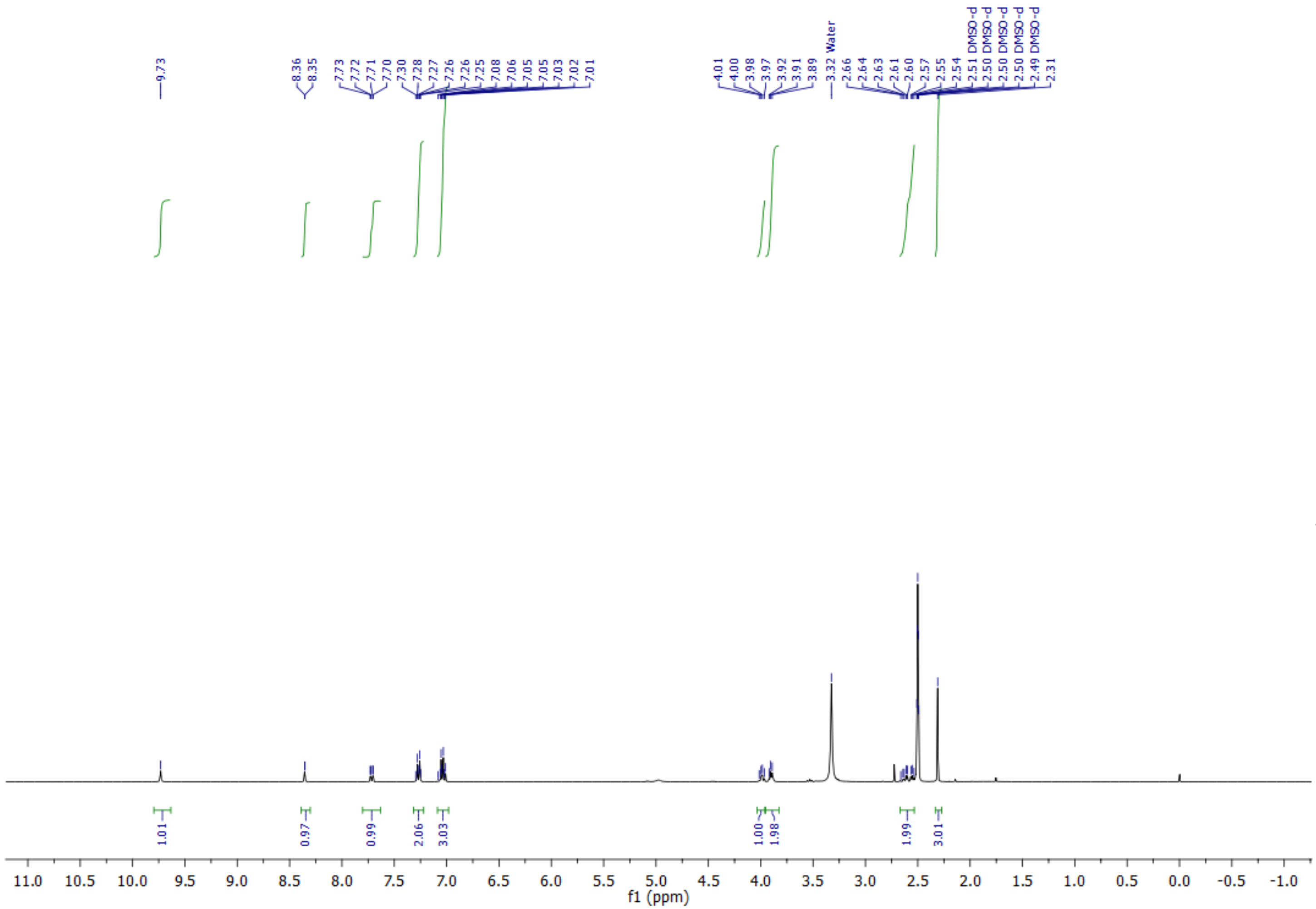

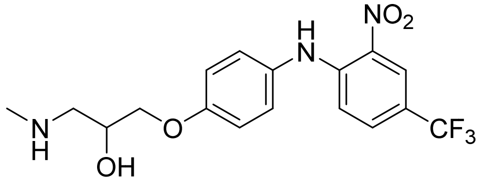

^{13}C NMR (101 MHz, $CDCl_3$) δ 157.84, 146.41, 131.85 (q, *J* = 2.9 Hz), 131.46, 130.46, 127.56, 124.86 (q, *J* = 4.9 Hz), 123.54 (q, *J* = 270.0 Hz), 118.95 (q, *J* = 34.4 Hz), 116.61, 116.01, 73.35, 70.99, 68.10, 63.07, 53.98, 47.99, 36.48, 31.09; HRMS (ESI$^+$) for $C_{17}H_{19}F_3N_3O_4$ ([M+H]$^+$) calculated 386.1322 found 386.1318

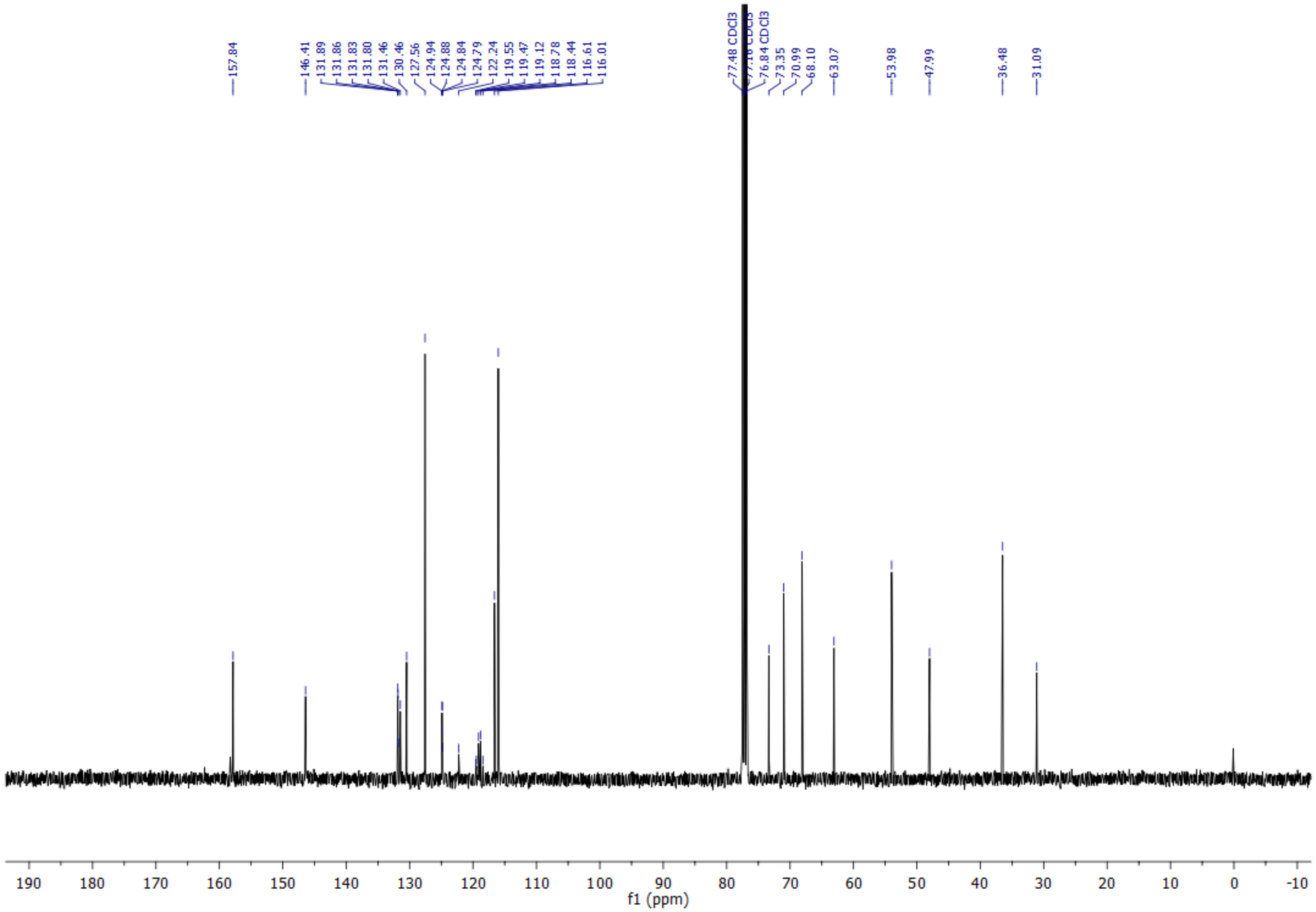

4-((3,4-Dichlorophenoxy)methyl)-1-(2-hydroxy-3-(4-((2-nitro-4-(trifluoromethyl)phenyl)amino)phenoxy)propyl)piperidin-4-ol (16a)

^{1}H NMR (400 MHz, $CDCl_3$) δ 9.61 (s, 1H), 8.49 (d, *J* = 1.0 Hz, 1H), 7.49 (dd, J_1 = 9.1 Hz, J_2 = 2.0 Hz, 1H), 7.34 (d, *J* = 8.9 Hz, 1H), 7.19 (t, *J* = 5.9 Hz, 2H), 7.11 – 6.95 (m, 4H), 6.79 (dd, J_1 = 8.9 Hz, J_2 = 2.9 Hz, 1H), 4.16 (td, J_1 = 9.3 Hz, J_2 = 4.8 Hz, 1H), 4.09 – 3.97 (m, 2H), 3.81 (s, 2H), 2.87 (d, *J* = 11.3 Hz, 1H), 2.83 – 2.57 (m, 4H), 2.51 (td, J_1 = 11.3 Hz, J_2 = 3.0 Hz, 1H), 1.92 – 1.66 (m, 4H)

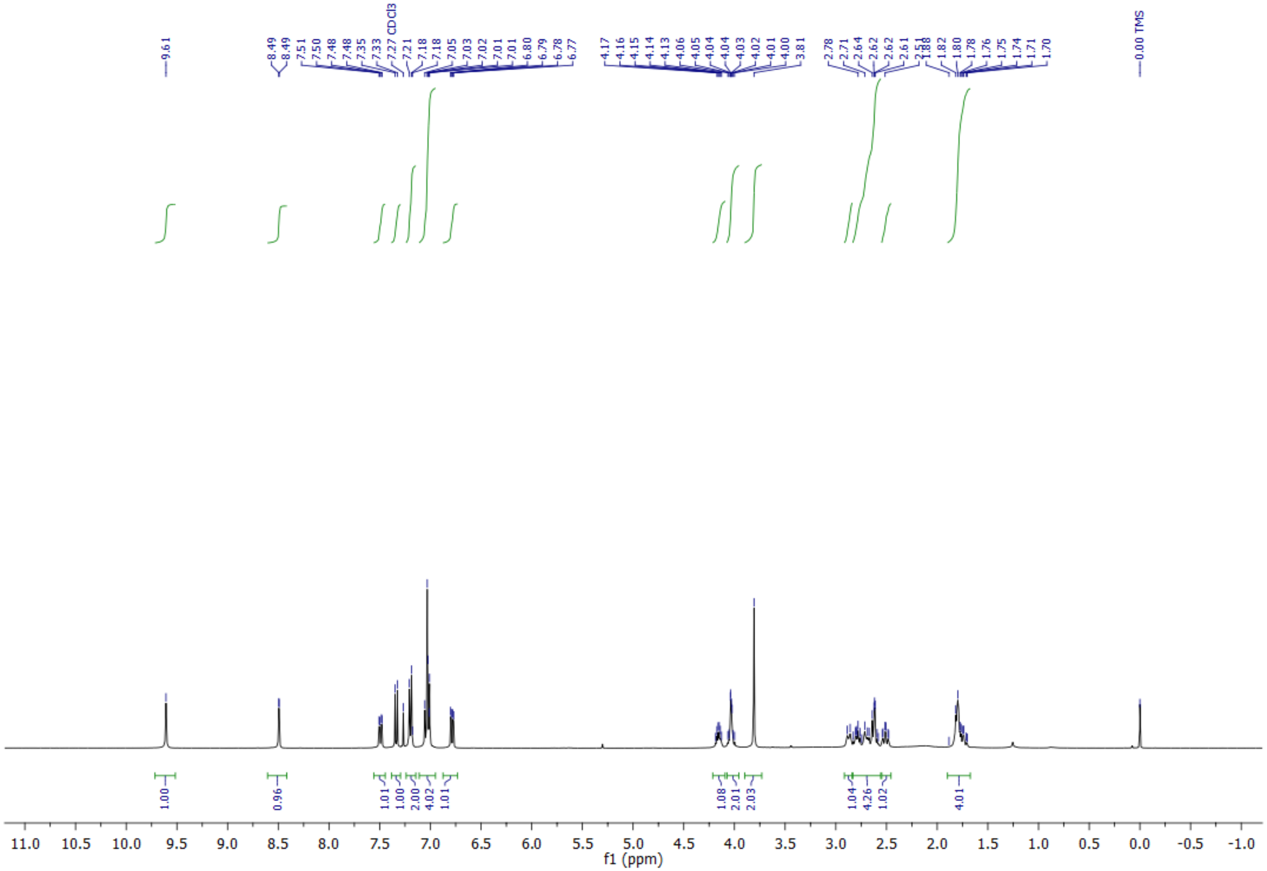

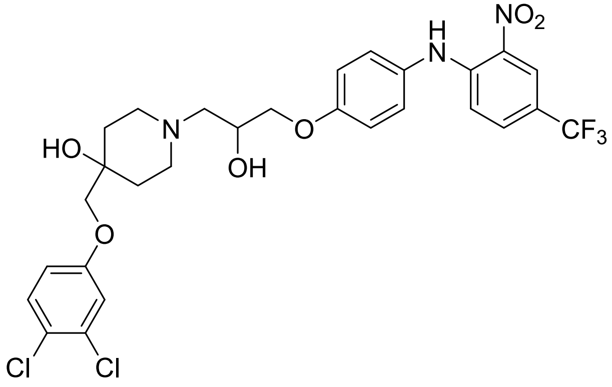

^{13}C NMR (101 MHz, $CDCl_3$) δ 157.90, 157.71, 146.39, 133.09, 131.82 (q, *J* = 3.0 Hz), 131.42, 130.90, 130.41, 127.51, 124.82 (q, *J* = 4.2 Hz), 124.69, 123.58 (q, *J* = 271.0 Hz), 118.90 (q, *J* = 34.4 Hz), 116.62, 116.60, 116.00, 114.67, 76.24, 70.79, 68.80, 65.64, 60.50, 50.41, 47.90, 34.21, 34.00; HRMS (ESI+) for $C_{28}H_{29}Cl_2F_3N_3O_6$ ([M+H]+) calculated 630.1380 found 630.1365

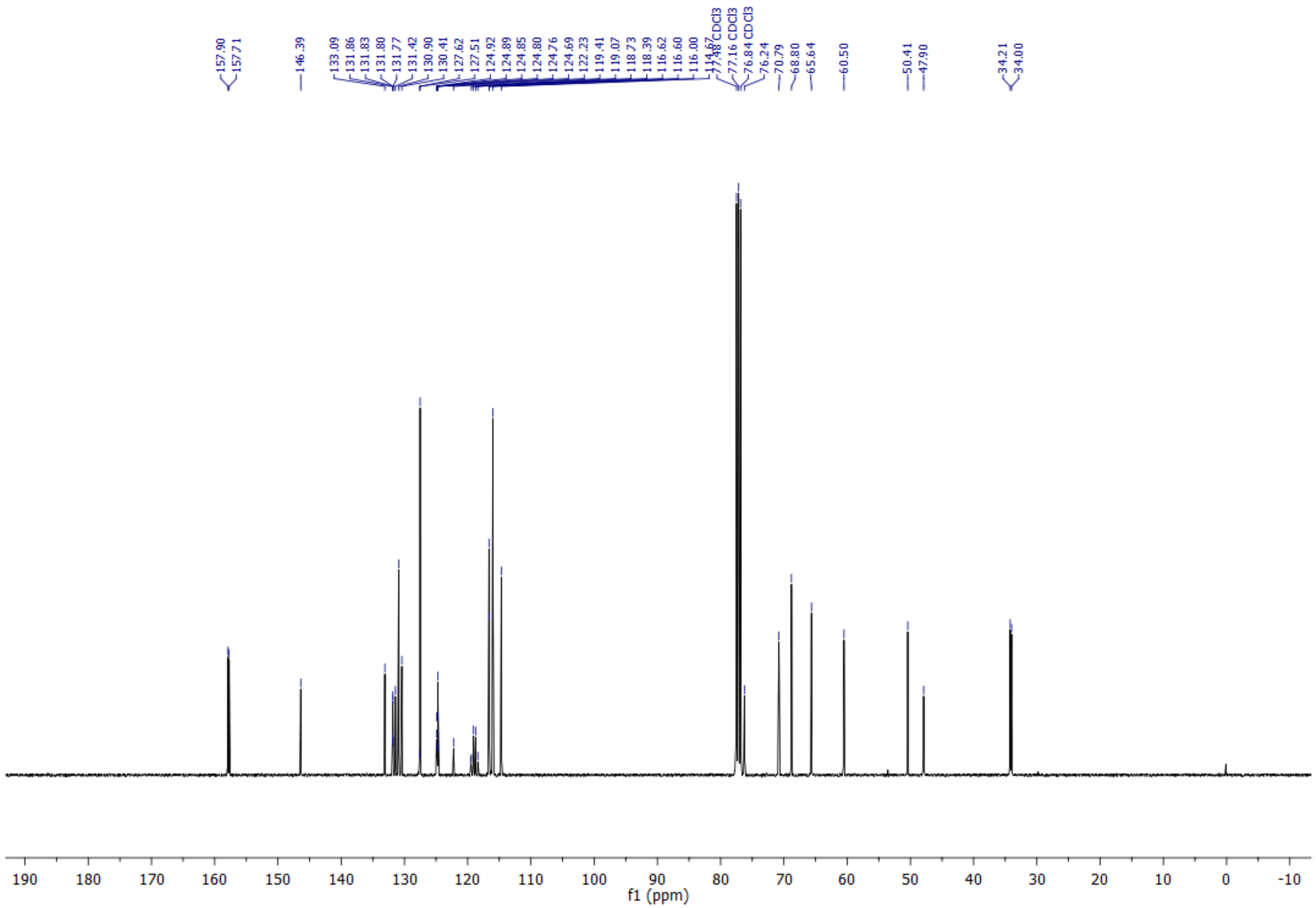

1-(2-Hydroxy-3-(4-((2-nitro-4-(trifluoromethyl)phenyl)amino)phenoxy)propyl)-4-((4-methoxyphenoxy)methyl)piperidin-4-ol (16b)

^{1}H NMR (400 MHz, $CDCl_3$) δ 9.60 (s, 1H), 8.49 (d, J = 1.1 Hz, 1H), 7.49 (dd, J_1 = 9.1 Hz, J_2 = 2.1 Hz, 1H), 7.18 (t, J = 6.0 Hz, 2H), 7.08 – 6.96 (m, 3H), 6.92 – 6.77 (m, 4H), 4.23 – 4.12 (m, 1H), 4.10 – 3.97 (m, 2H), 3.78 (s, 2H), 3.77 (s, 3H), 2.91 – 2.75 (m, 2H), 2.74 – 2.57 (m, 3H), 2.52 (td, J_1 = 11.3 Hz, J_2 = 2.9 Hz, 1H), 1.88 – 1.67 (m, 4H)

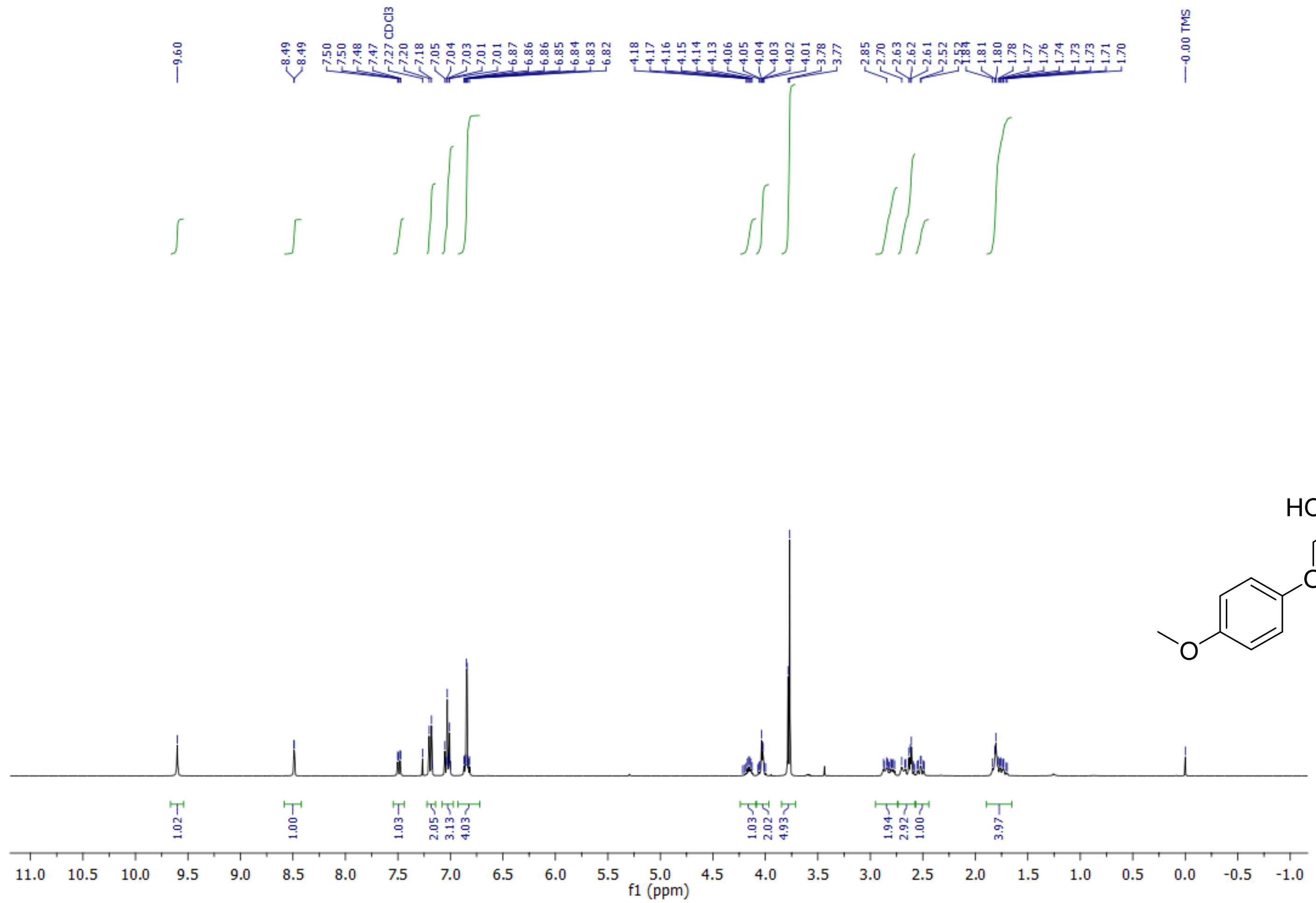

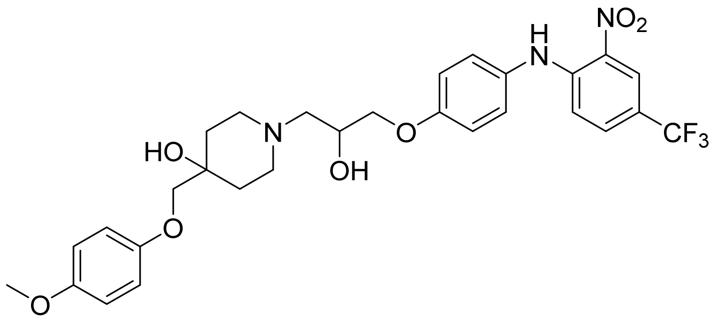

^{13}C NMR (101 MHz, $CDCl_3$) δ 157.92, 154.30, 152.87, 146.40, 131.80 (q, *J* = 2.9 Hz), 131.41, 130.37, 127.50, 124.81 (q, *J* = 4.2 Hz), 123.58 (q, *J* = 270.9 Hz), 118.87 (q, *J* = 34.3 Hz), 116.60, 116.00, 115.68, 114.81, 76.43, 70.82, 68.85, 65.60, 60.52, 55.83, 50.55, 48.06, 34.29, 34.08; HRMS (ESI$^+$) for $C_{29}H_{33}F_3N_3O_7$ ([M+H]$^+$) calculated 592.2265 found 592.2251

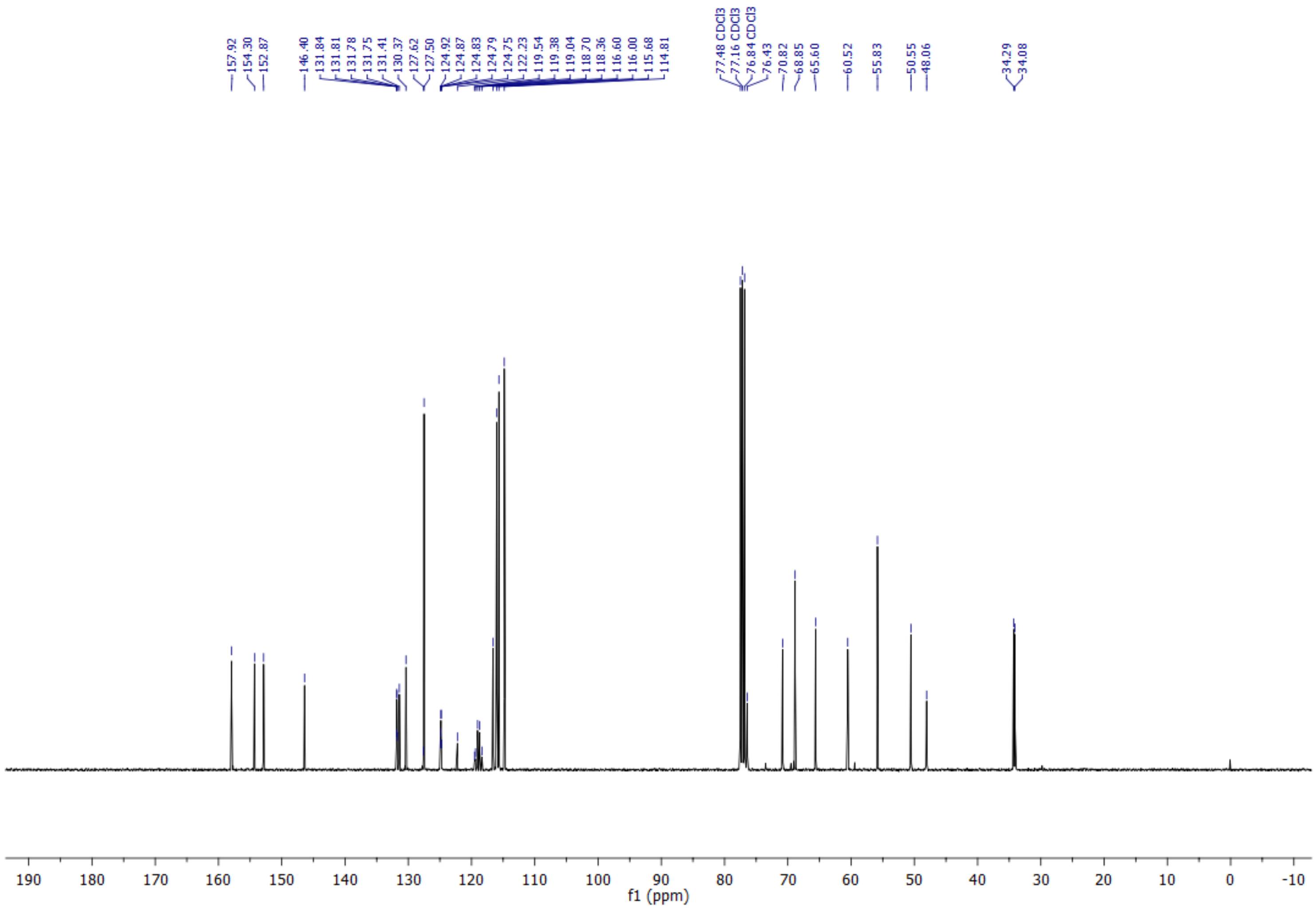

3,4-Dichloro-N-(1-(2-hydroxy-3-(4-((2-nitro-4-(trifluoromethyl)phenyl)amino)phenoxy)propyl)piperidin-4-yl)benzamide (16c)

^{1}H NMR (400 MHz, $CDCl_3$) δ 9.60 (s, 1H), 8.50 (d, J = 1.1 Hz, 1H), 7.85 (d, J = 2.0 Hz, 1H), 7.59 (dd, J_1 = 8.3 Hz, J_2 = 2.0 Hz, 1H), 7.54 – 7.46 (m, 2H), 7.24 – 7.16 (m, 2H), 7.08 – 6.96 (m, 3H), 6.07 (d, J = 7.7 Hz, 1H), 4.13 (td, J_1 = 9.3 Hz, J_2 = 4.4 Hz, 1H), 4.09 – 3.96 (m, 3H), 3.07 (d, J = 11.6 Hz, 1H), 2.90 (d, J = 11.7 Hz, 1H), 2.70 – 2.48 (m, 3H), 2.29 – 2.19 (m, 1H), 2.09 (d, J = 11.2 Hz, 2H), 1.72 – 1.50 (m, 2H)

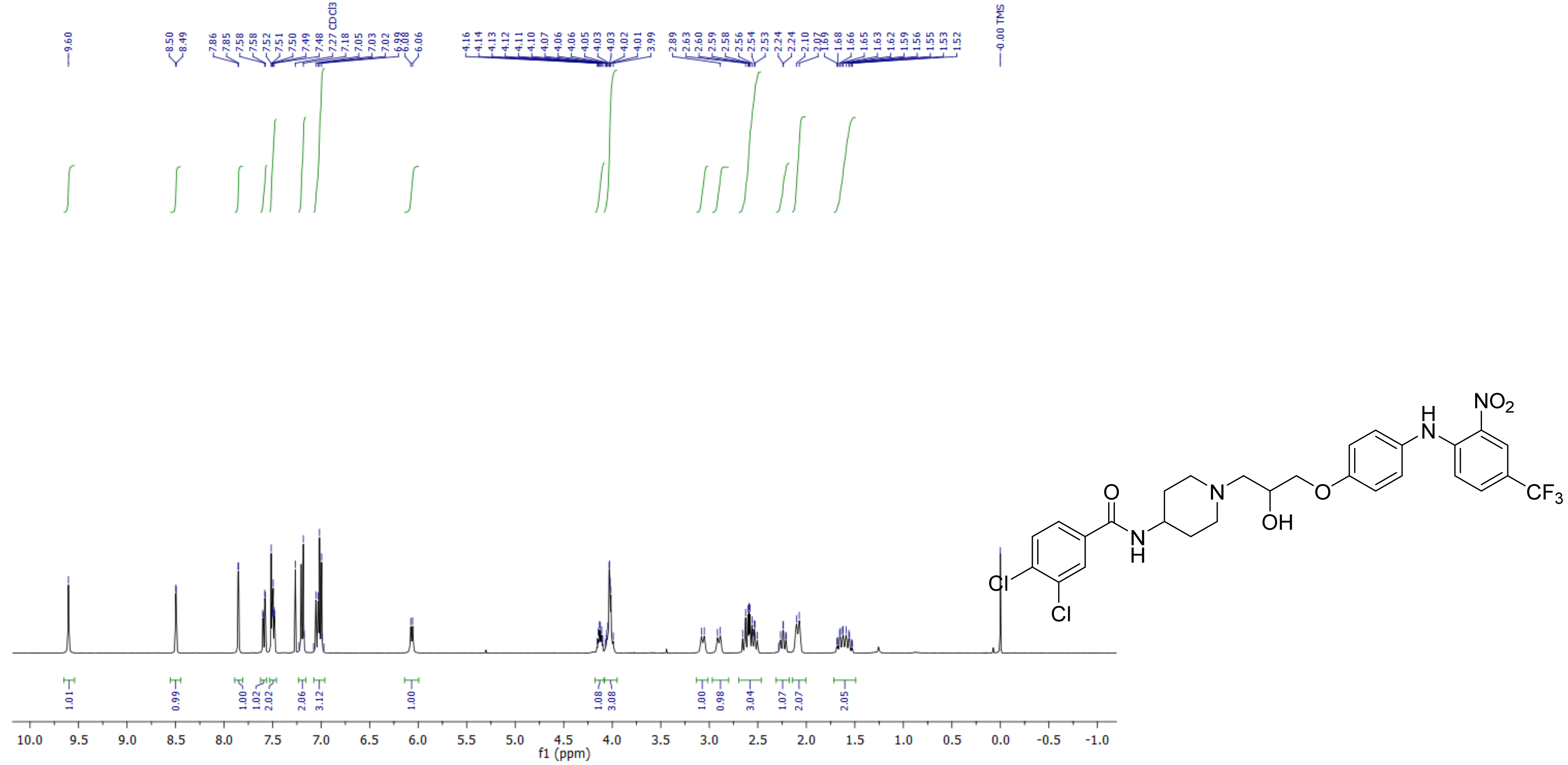

^{13}C NMR (101 MHz, $CDCl_3$) δ 164.84, 157.85, 146.40, 136.06, 134.53, 133.21, 131.85 (q, *J* = 2.9 Hz), 131.47, 130.77, 130.47, 129.25, 127.54, 126.21, 124.87 (q, *J* = 4.6 Hz), 123.59 (q, *J* = 271.1 Hz), 118.97 (q, *J* = 34.4 Hz), 116.60, 115.99, 70.67, 65.74, 60.36, 54.15, 51.34, 47.40, 32.63, 32.33; HRMS (ESI$^+$) for $C_{28}H_{28}Cl_2F_3N_4O_5$ ([M+H]$^+$) calculated 627.1383 found 627.1369

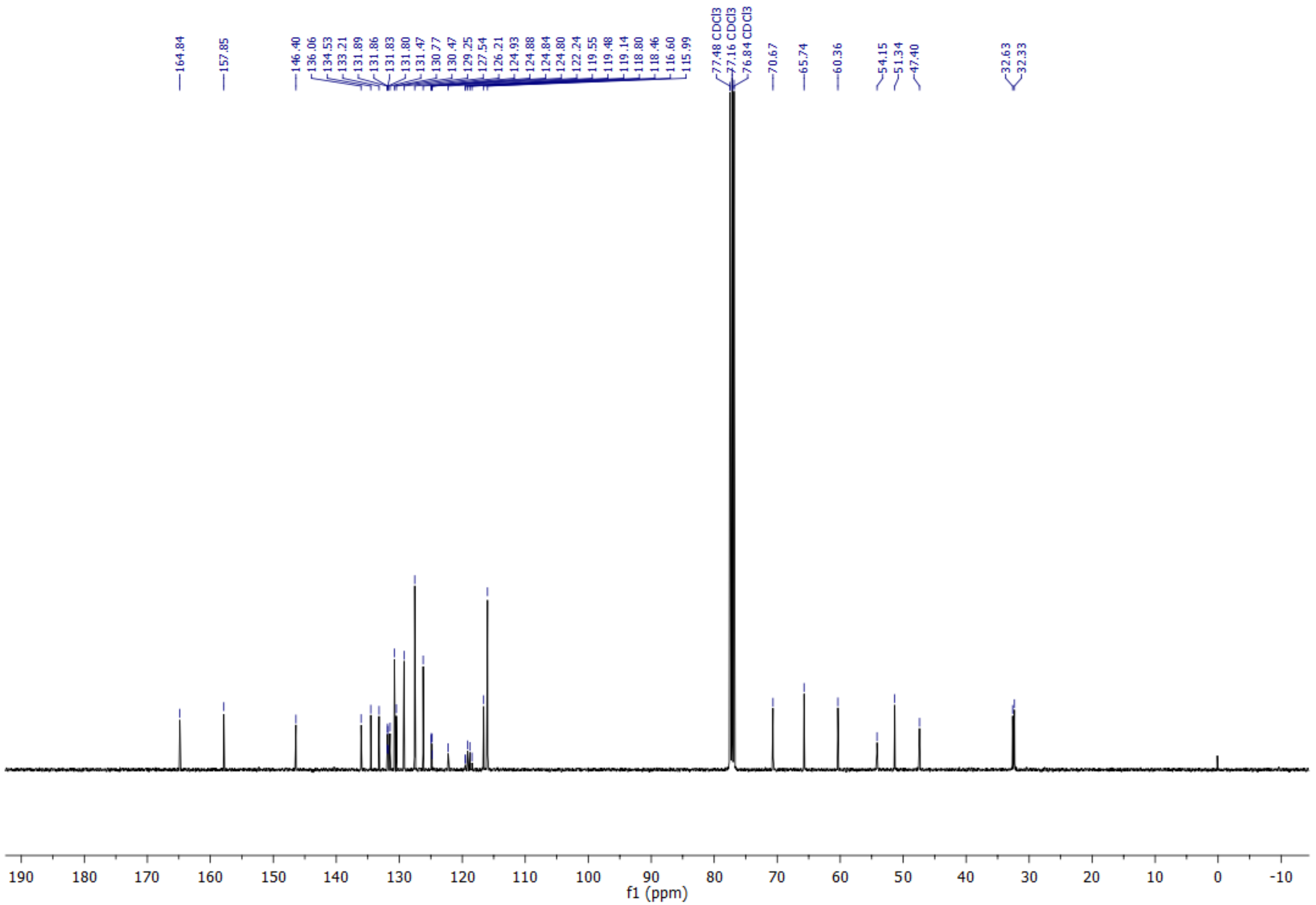

2-(3,4-Dichlorophenyl)-N-(1-(2-hydroxy-3-(4-((2-nitro-4-(trifluoromethyl)phenyl)amino)phenoxy)propyl)piperidin-4-yl)acetamide (16d)

^{1}H NMR (400 MHz, DMSO) δ 9.74 (s, 1H), 8.35 (d, *J* = 1.3 Hz, 1H), 8.04 (d, *J* = 7.6 Hz, 1H), 7.72 (dd, J_1 = 9.2 Hz, J_2 = 2.2 Hz, 1H), 7.55 (d, *J* = 8.2 Hz, 1H), 7.50 (d, *J* = 2.0 Hz, 1H), 7.31 – 7.25 (m, 2H), 7.22 (dd, J_1 = 8.3 Hz, J_2 = 2.0 Hz, 1H), 7.13 – 6.98 (m, 3H), 4.87 (d, *J* = 4.2 Hz, 1H), 4.01 (dd, *J* = 9.2, 3.1 Hz, 1H), 3.98 – 3.85 (m, 1H), 3.56 – 3.44 (m, 1H), 3.41 (s, 1H), 2.91 – 2.77 (m, 1H), 2.40 (ddd, *J* = 33.4, 12.7, 5.9 Hz, 1H), 2.08 (dd, *J* = 19.9, 9.4 Hz, 1H), 1.76 – 1.63 (m, 1H), 1.39 (dd, *J* = 21.8, 10.8 Hz, 1H)

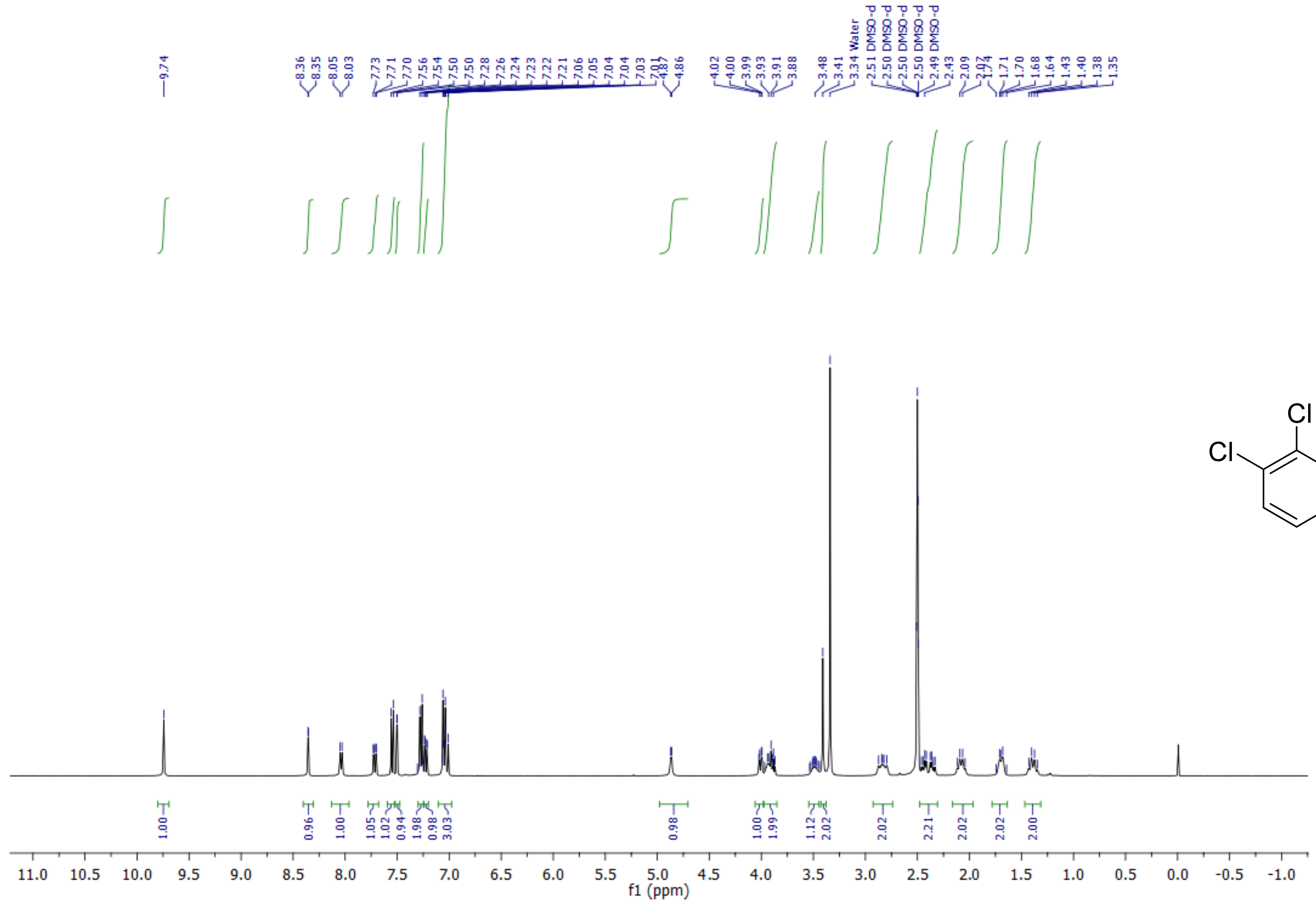

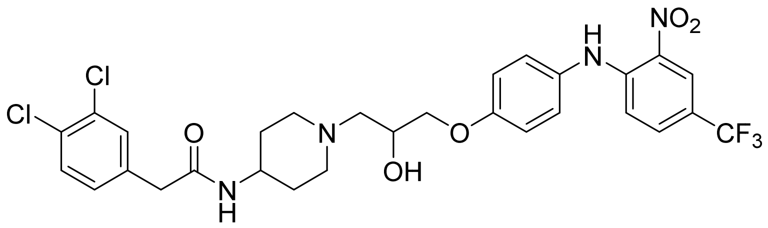

^{13}C NMR (101 MHz, $CDCl_3$) δ 169.14, 157.87, 146.40, 135.13, 132.98, 131.84 (q, *J* = 2.9 Hz), 131.68, 131.45, 131.31, 130.91, 130.44, 128.74, 127.54, 124.86 (q, *J* = 4.7 Hz), 123.55 (q, *J* = 277.7 Hz), 118.96 (q, *J* = 34.5 Hz), 116.60, 116.00, 70.69, 65.69, 60.32, 54.02, 51.25, 46.90, 42.85, 32.50, 32.21; HRMS (ESI$^+$) for $C_{29}H_{29}Cl_2F_3N_4O_5$ ($[M+H]^+$) calculated 641.1540 found 641.1524

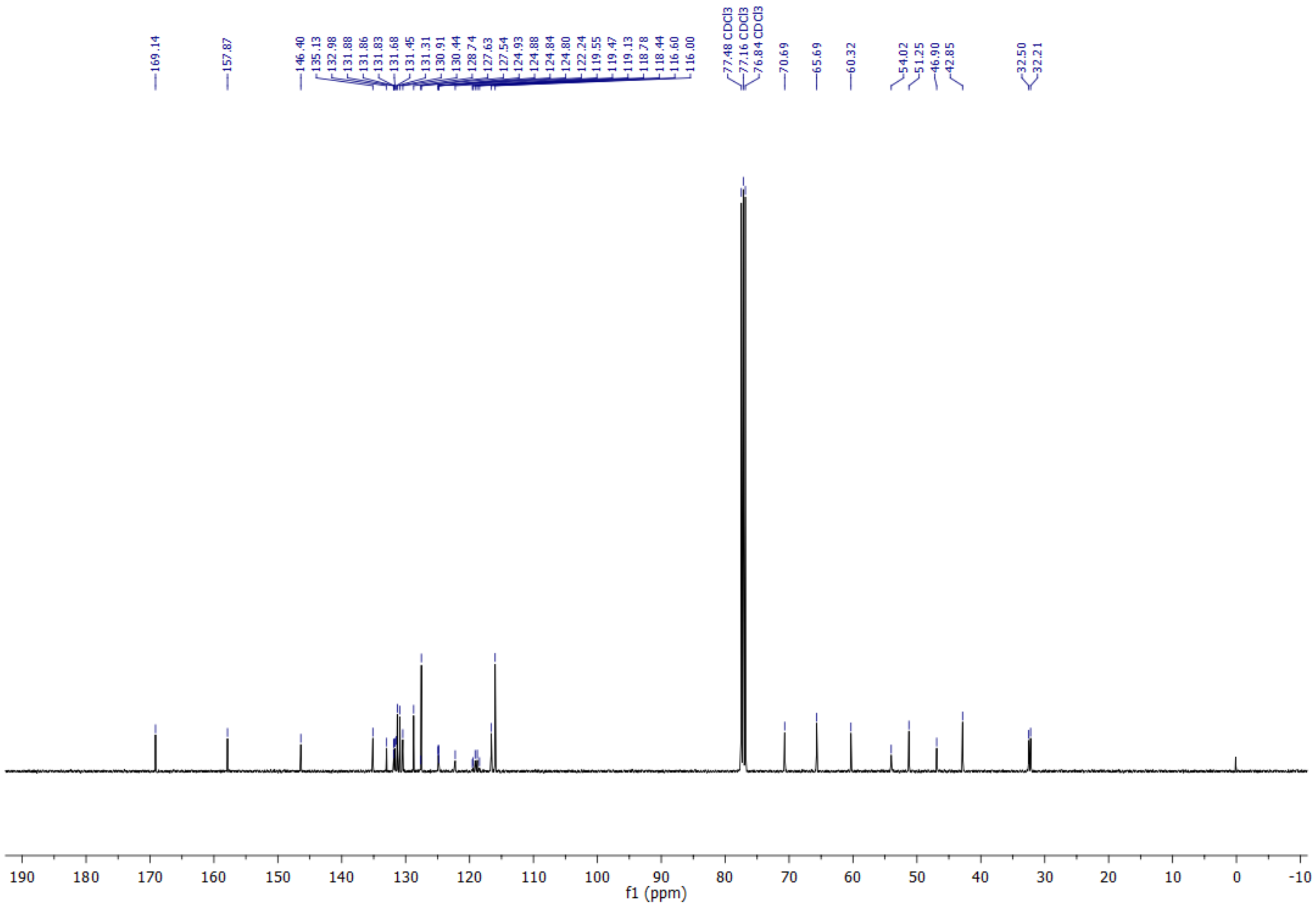

_N_-(4-(2-(Diethylamino)ethoxy)phenyl)-2-nitro-4-(trifluoromethyl)aniline (17a)

^{1}H NMR (400 MHz, $CDCl_3$) δ 9.60 (s, 1H), 8.49 (d, *J* =, 1.3 Hz, 1H), 7.48 (dd, J_1 = 9.1 Hz, J_2 = 2.1 Hz, 1H), 7.20 – 7.14 (m, 2H), 7.04 (d, *J* = 9.1 Hz, 1H), 7.01 – 6.95 (m, 2H), 4.07 (t, *J* = 6.3 Hz, 2H), 2.90 (t, *J* = 6.3 Hz, 2H), 2.66 (q, *J* = 7.1 Hz, 4H), 1.09 (t, *J* = 7.1 Hz, 6H)

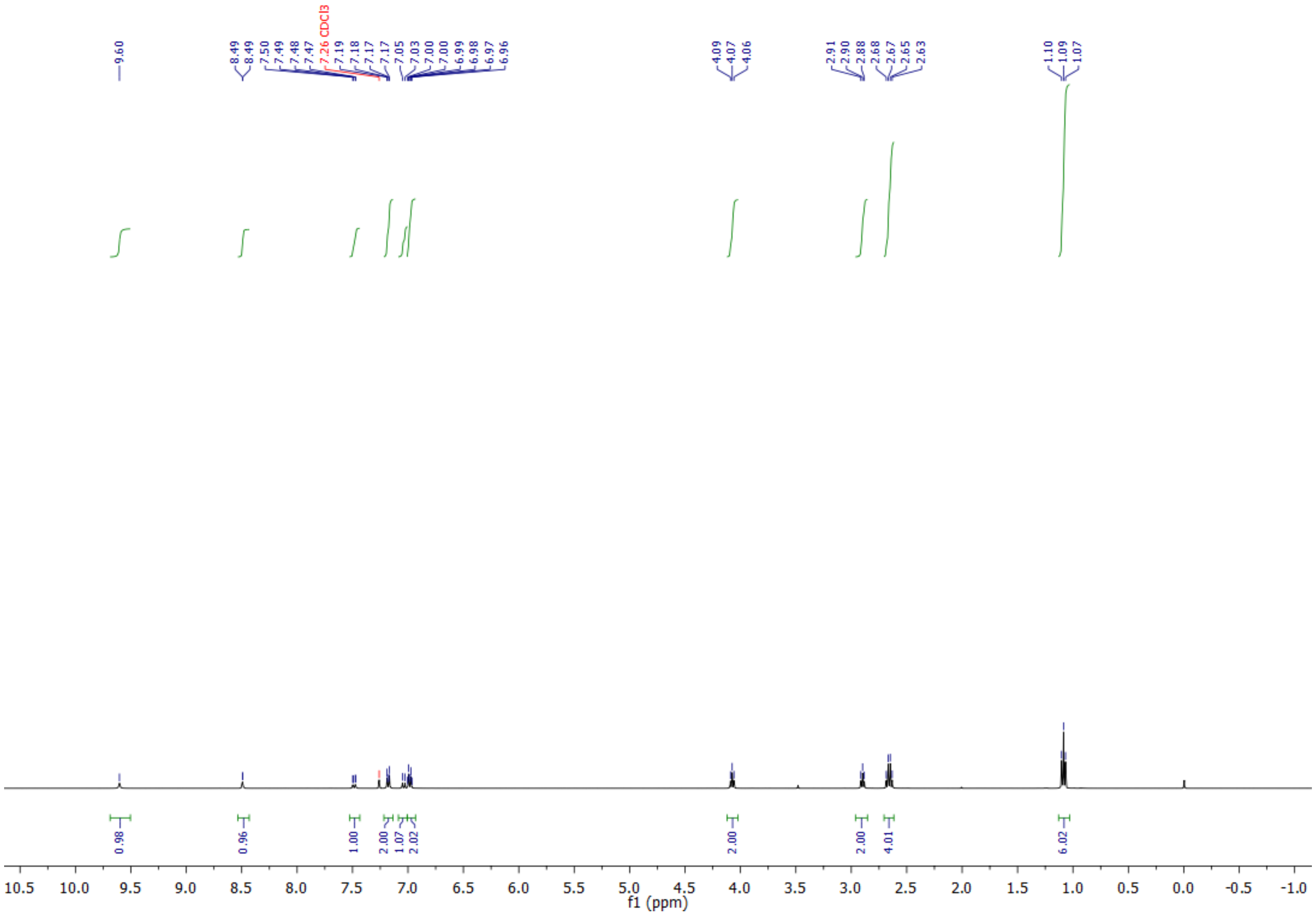

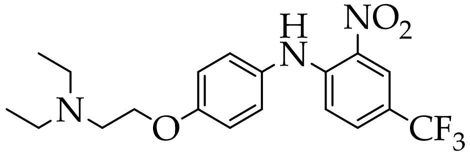

^{13}C NMR (101 MHz, $CDCl_3$) δ 158.11, 146.52, 131.82 (q, *J* = 3.0 Hz), 131.41, 130.06, 127.54, 124.86 (q, J = 4.3 Hz), 123.62 (q, *J* = 270.8 Hz), 118.87 (q, *J* = 34.4 Hz), 116.65, 115.96, 67.20, 51.87, 48.05, 12.01; HRMS (ESI$^+$) for $C_{19}H_{22}F_3N_3O_3$ ([M+H]$^+$) calculated 398.1686 found 398.1682

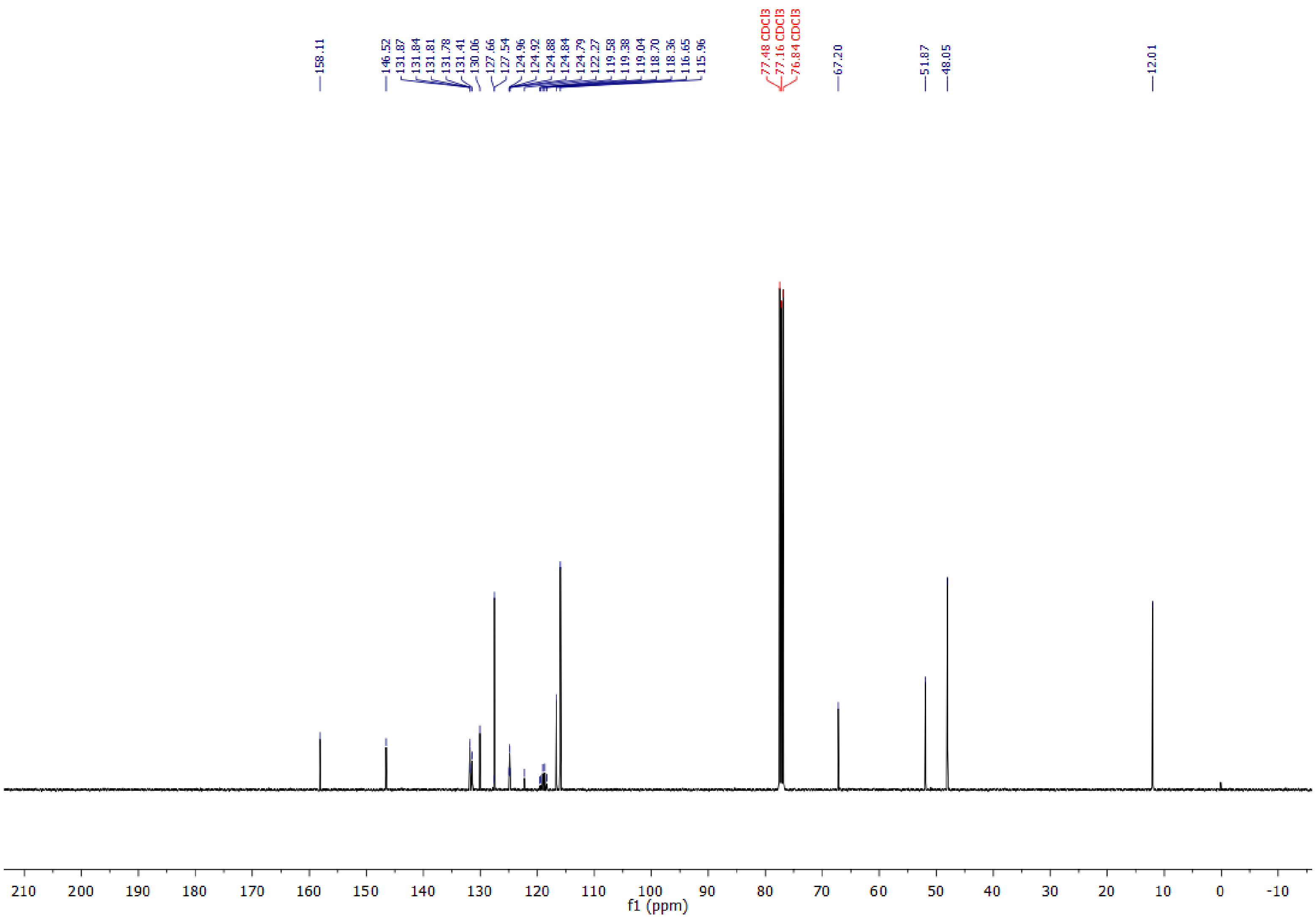

3,5-Dibromo-4-(2-(diethylamino)ethoxy)-*N*-(2-nitro-4-(trifluoromethyl)phenyl)aniline (17b)

^{1}H NMR (400 MHz, $CDCl_3$) δ 9.53 (s, 1H), 8.49 (d, *J* = 1.4 Hz, 1H), 7.63 (dd, J_1 = 9.0 Hz, J_2 = 2.0 Hz, 1H), 7.48 (s, 2H), 7.21 (d, *J* = 9.0 Hz, 1H), 4.37 (t, *J* = 5.0 Hz, 2H), 3.54 (t, *J* = 9.2 Hz, 2H), 3.29 (q, J = 6.8 Hz, 4H), 1.36 (t, *J* = 7.2 Hz, 6H)

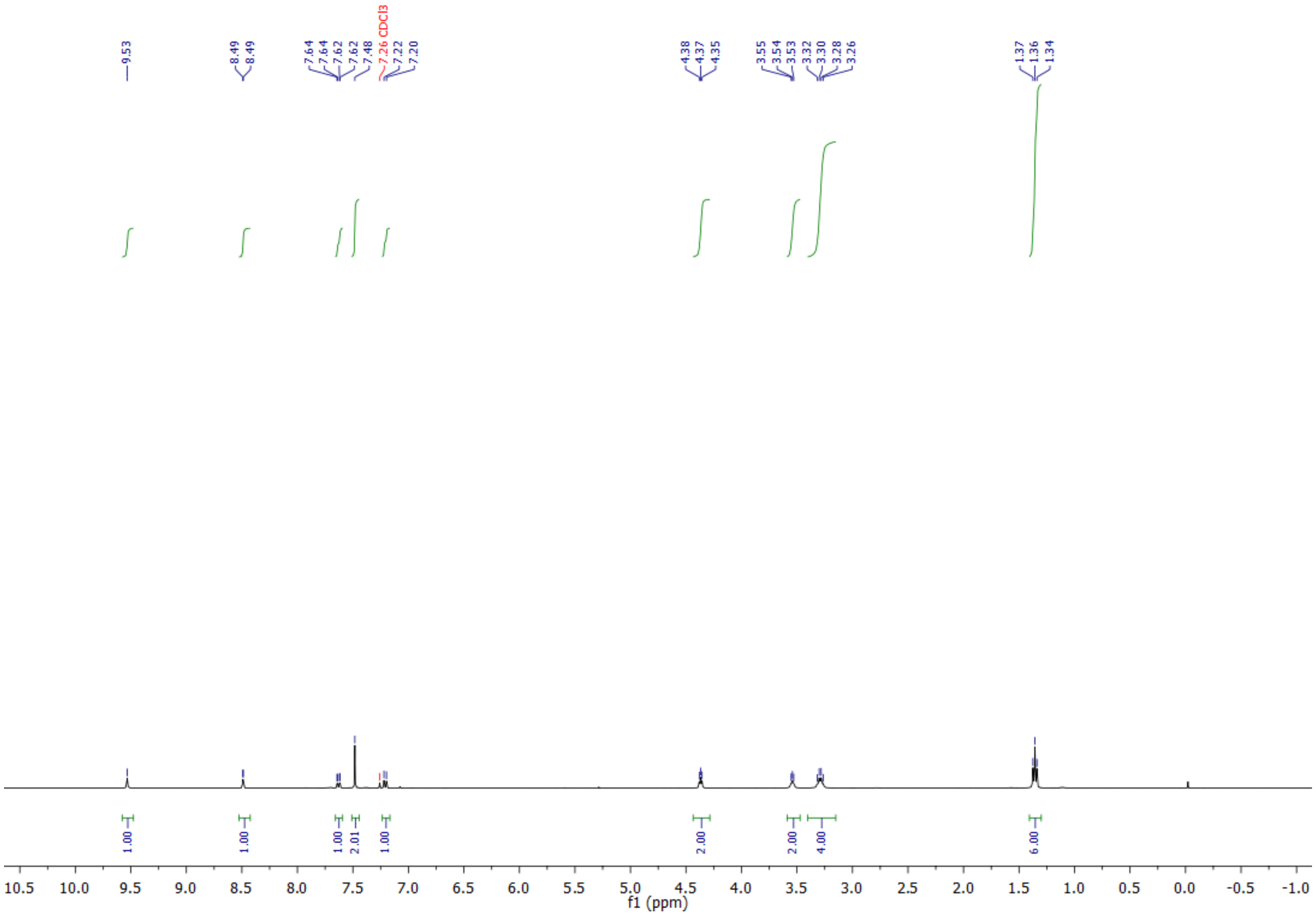

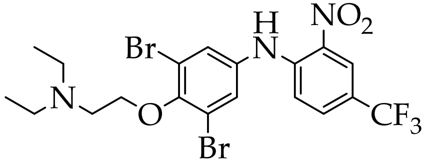

^{13}C NMR (101 MHz, $CDCl_3$) δ 151.00, 144.11, 136.23, 132.75, 132.30 (q, *J* = 3.1 Hz), 129.08, 124.88 (q, *J* = 4.1 Hz), 123.25 (q, *J* = 271.8 Hz), 120.85 (q, *J* = 34.6 Hz), 119.20, 116.58, 68.46, 50.73, 47.52, 9.53; HRMS (ESI$^+$) for $C_{19}H_{20}Br_2F_3N_3O_3$ ([M+H]$^+$) calculated 553.9896 found 553.9894

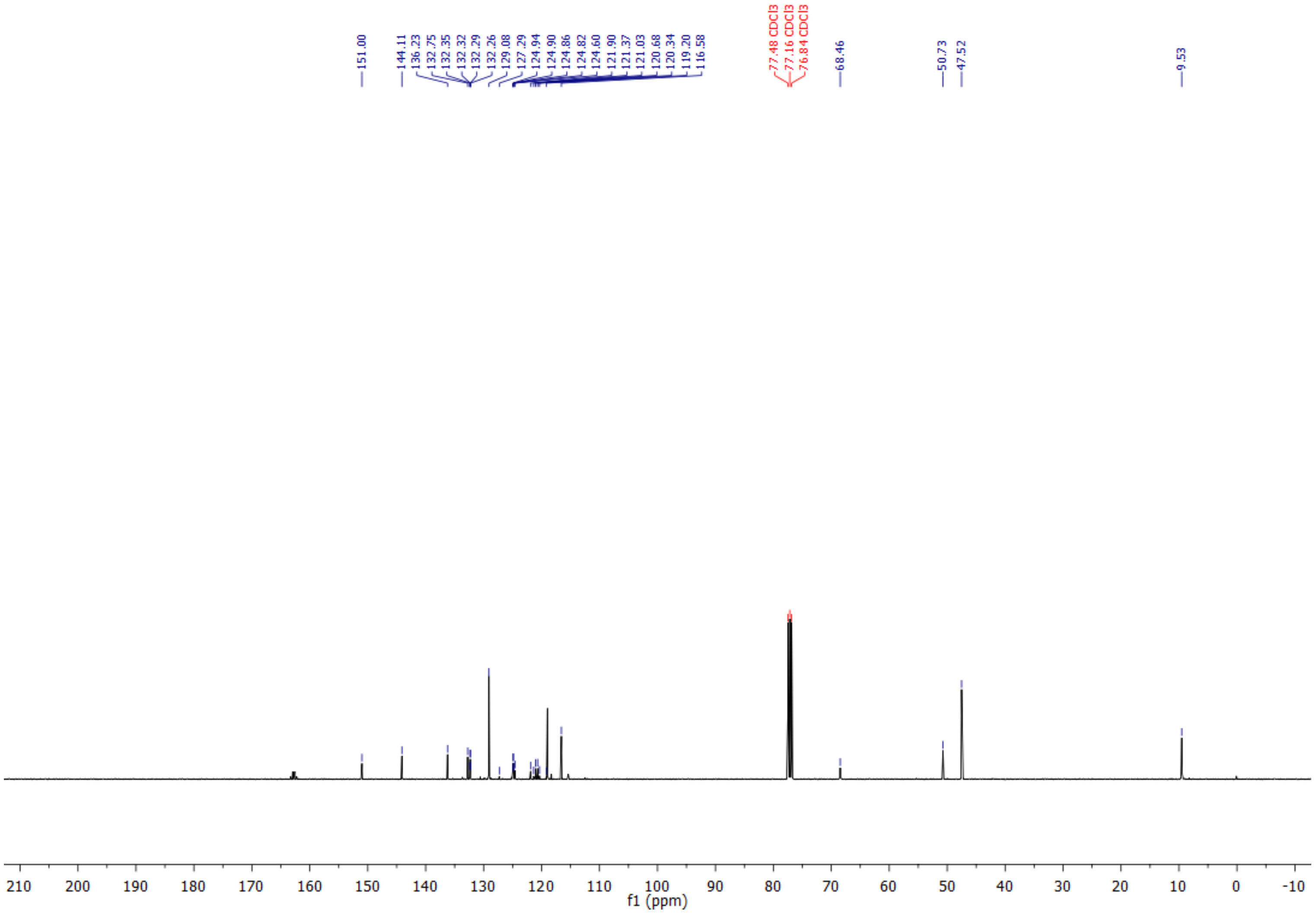

3,5-Dichloro-4-(2-(diethylamino)ethoxy)-*N*-(2-nitro-4-(trifluoromethyl)phenyl)aniline (17c)

^{1}H NMR (400 MHz, $CDCl_3$) δ 9.53 (s, 1H), 8.51 (d, J = 1.3 Hz, 1H), 7.62 (dd, J_1 = 9.0 Hz, J_2 = 2.1 Hz, 1H), 7.26 (s, 2H), 7.21 (d, J = 9.0 Hz, 1H), 4.14 (t, J = 6.5 Hz, 2H), 3.00 (t, J = 6.5 Hz, 2H), 2.69 (q, J = 7.1 Hz, 4H), 1.09 (t, J = 7.1 Hz, 6H)

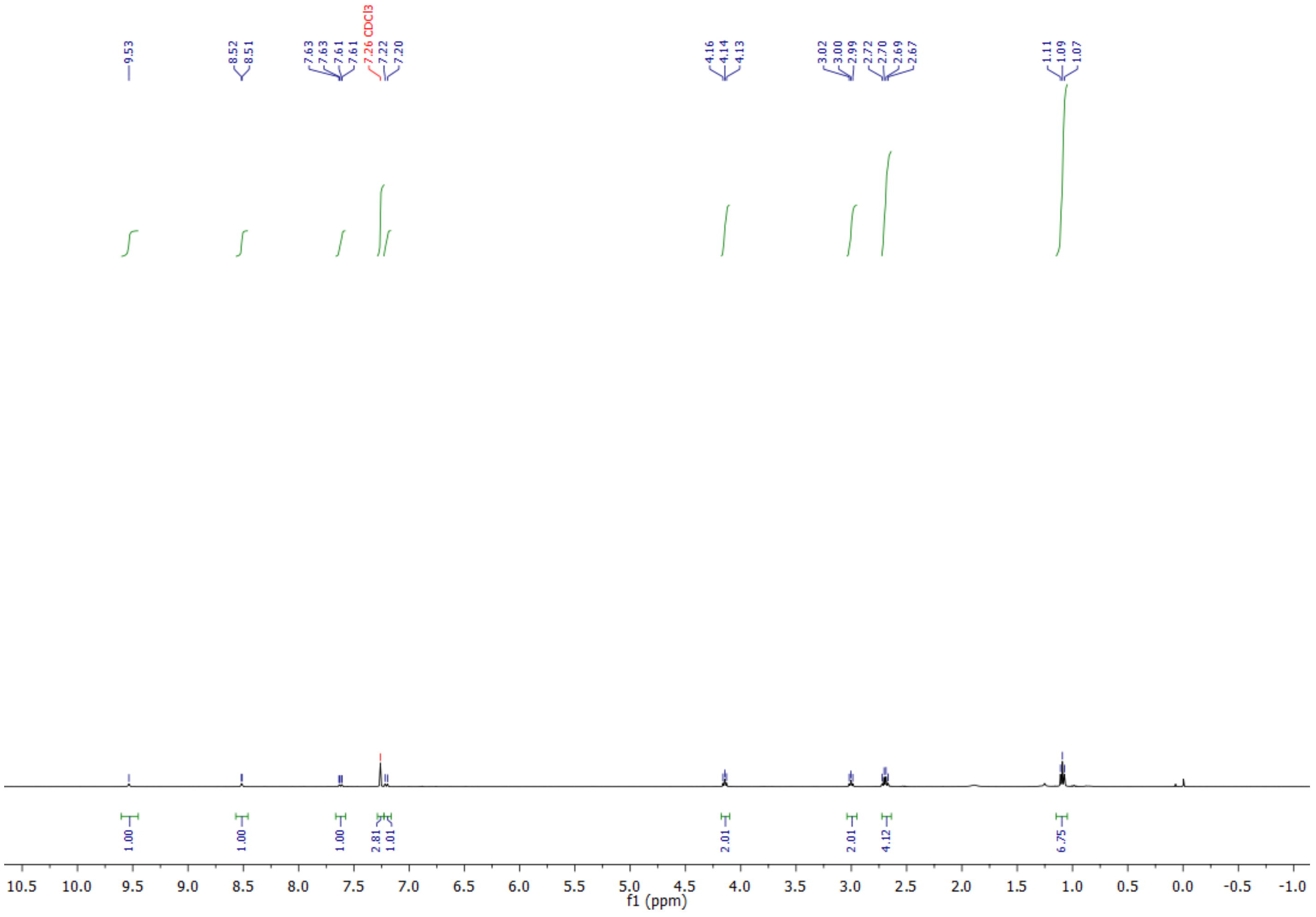

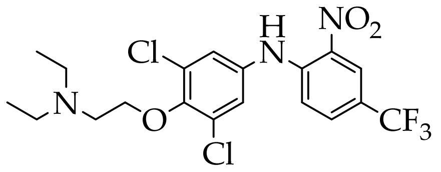

^{13}C NMR (101 MHz, $CDCl_3$) δ 150.59, 144.51, 134.30, 132.56, 132.27 (q, *J* = 3.0 Hz), 130.74, 125.63, 124.95 (q, *J* = 4.1 Hz), 123.35 (q, *J* = 271.5 Hz), 120.58 (q, *J* = 34.6 Hz), 116.63, 72.00, 52.37, 47.64, 11.87; HRMS (ESI$^+$) for $C_{19}H_{20}Cl_2F_3N_3O_3$ ($[M+H]^+$) calculated 466.0903 found 466.0907

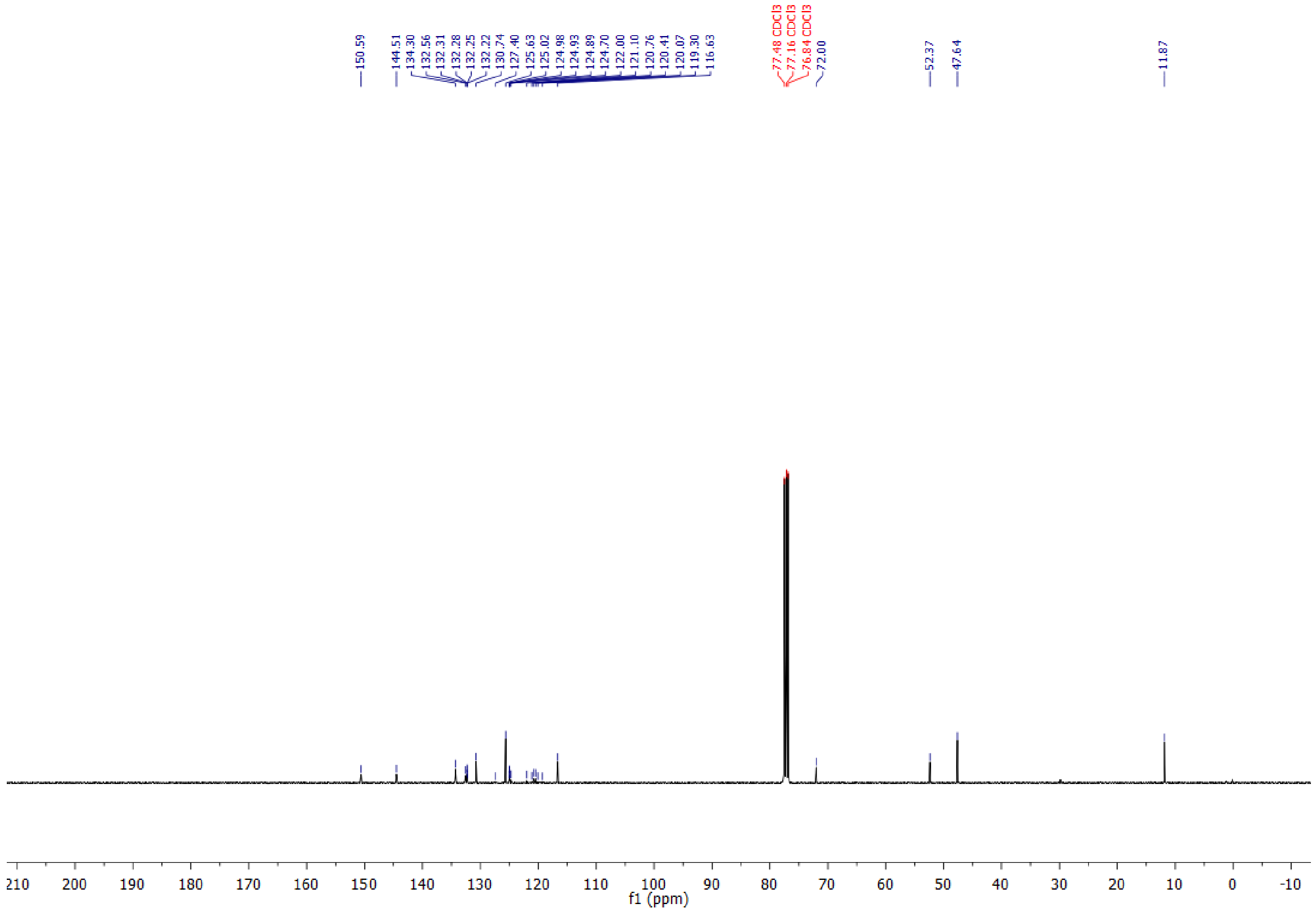

N-(4-(2-(Dimethylamino)ethoxy)phenyl)-2-nitro-4-(trifluoromethyl)aniline (18a)

^{1}H NMR (400 MHz, $CDCl_3$) δ 9.61 (s, 1H), 8.50 (d, J = 1.1 Hz, 1H), 7.49 (dd, J_1 = 9.1 Hz, J_2 = 2.1 Hz, 1H), 7.22 – 7.14 (m, 2H), 7.08 – 6.96 (m, 3H), 4.11 (t, J = 5.7 Hz, 2H), 2.78 (t, J = 5.7 Hz, 2H), 2.38 (s, 6H)

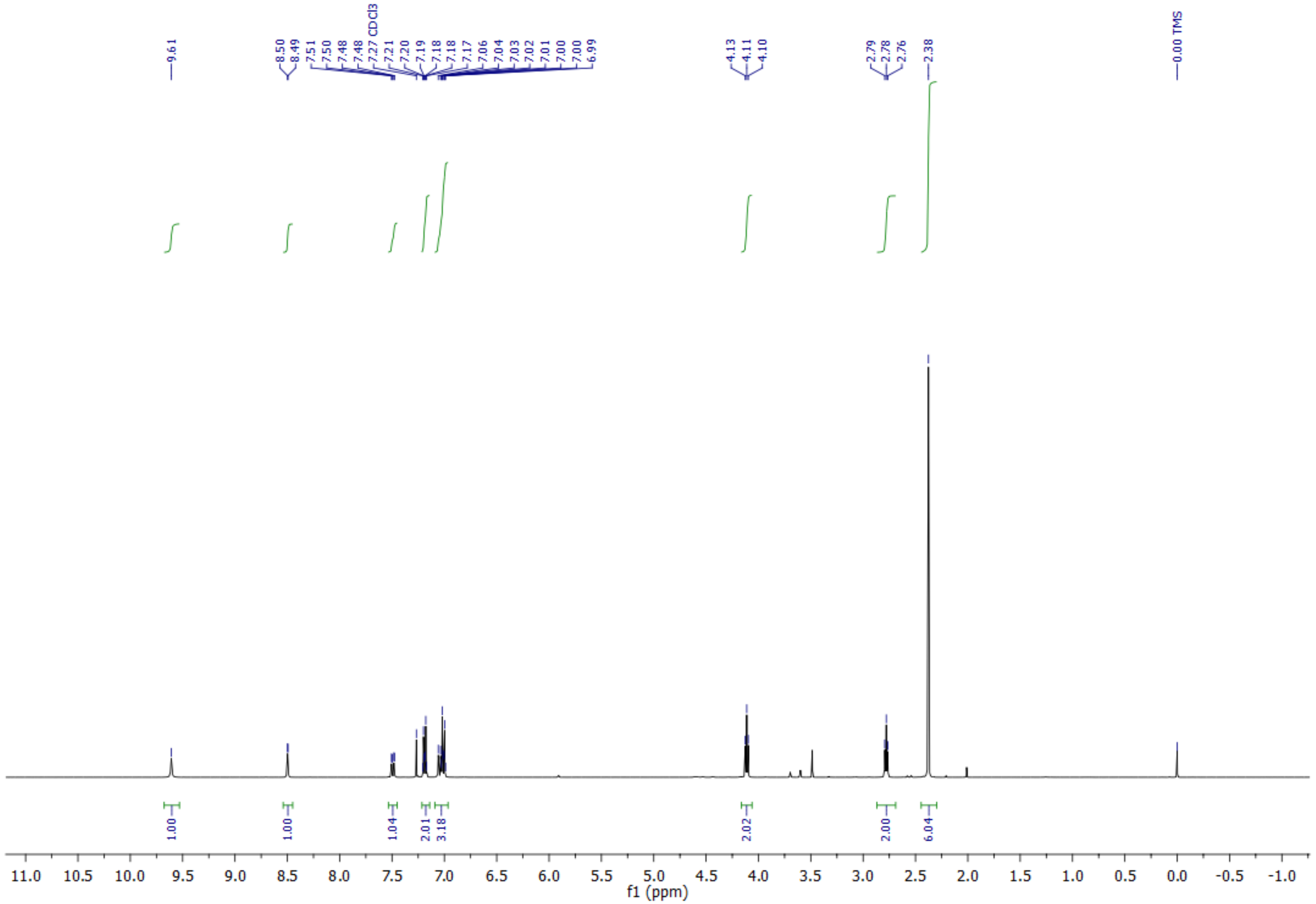

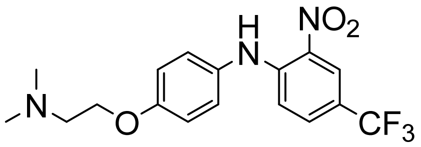

^{13}C NMR (101 MHz, $CDCl_3$) δ 158.01, 146.48, 131.83 (q, *J* = 3.1 Hz), 131.42, 130.19, 127.52, 124.85 (q, *J* = 4.3 Hz), 123.61 (q, *J* = 271.0 Hz), 118.89 (q, *J* = 34.4 Hz), 116.65, 116.00, 66.46, 58.37, 46.05; HRMS (ESI+) for $C_{17}H_{19}F_3N_3O_3$ ($[M+H]^+$) calculated 370.1373 found 370.1368

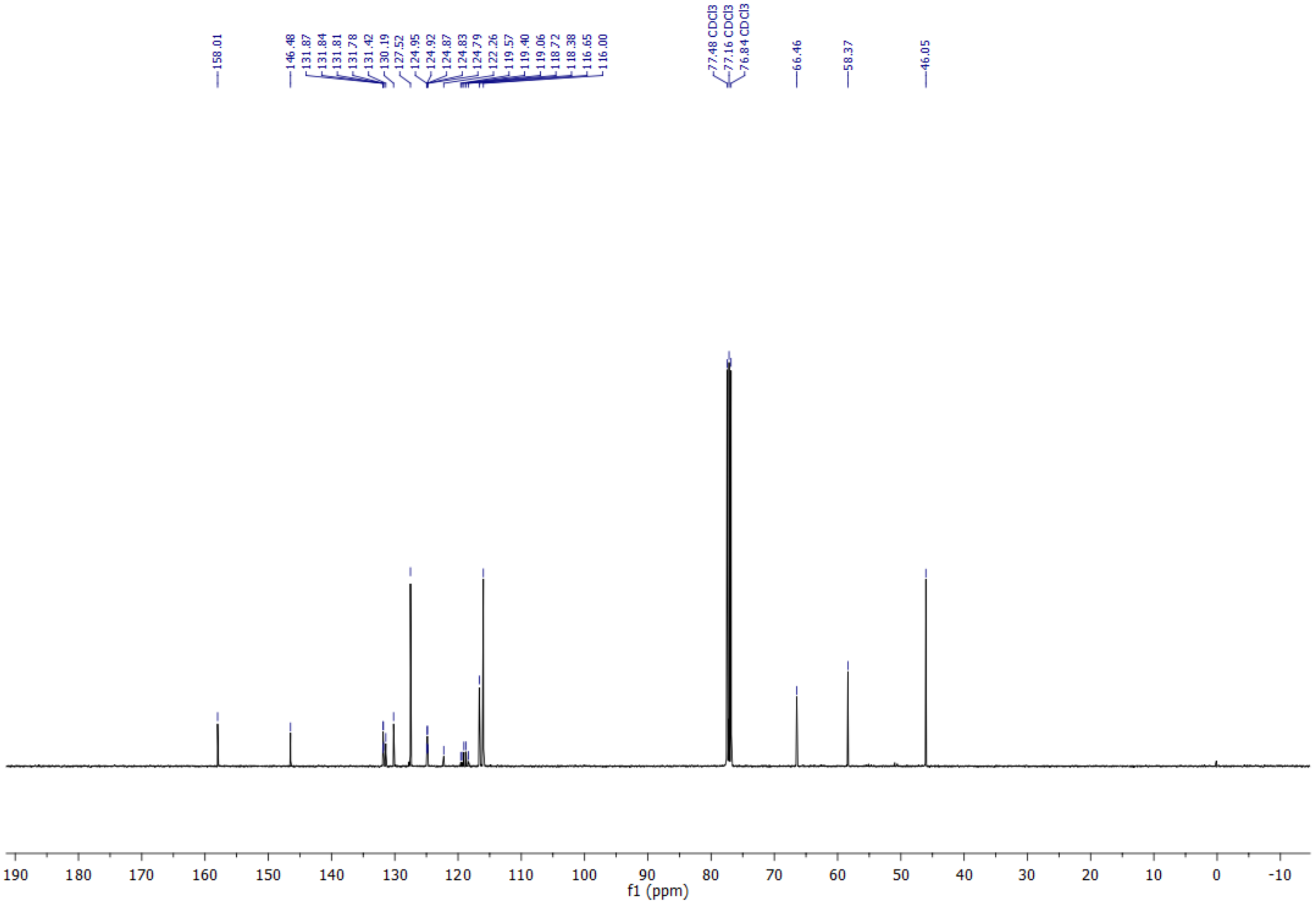

3,5-Dibromo-4-(2-(dimethylamino)ethoxy)-N-(2-nitro-4-(trifluoromethyl)phenyl)aniline (18b)

^{1}H NMR (400 MHz, $CDCl_3$) δ 9.54 (s, 1H), 8.51 (d, *J* = 1.3 Hz, 1H), 7.62 (dd, J_1 = 9.0 Hz, J_2 = 2.0 Hz, 1H), 7.47 (s, 2H), 7.20 (d, *J* = 9.0 Hz, 1H), 4.14 (t, *J* = 5.9 Hz, 2H), 2.85 (t, *J* = 5.9 Hz, 2H), 2.39 (s, 6H)

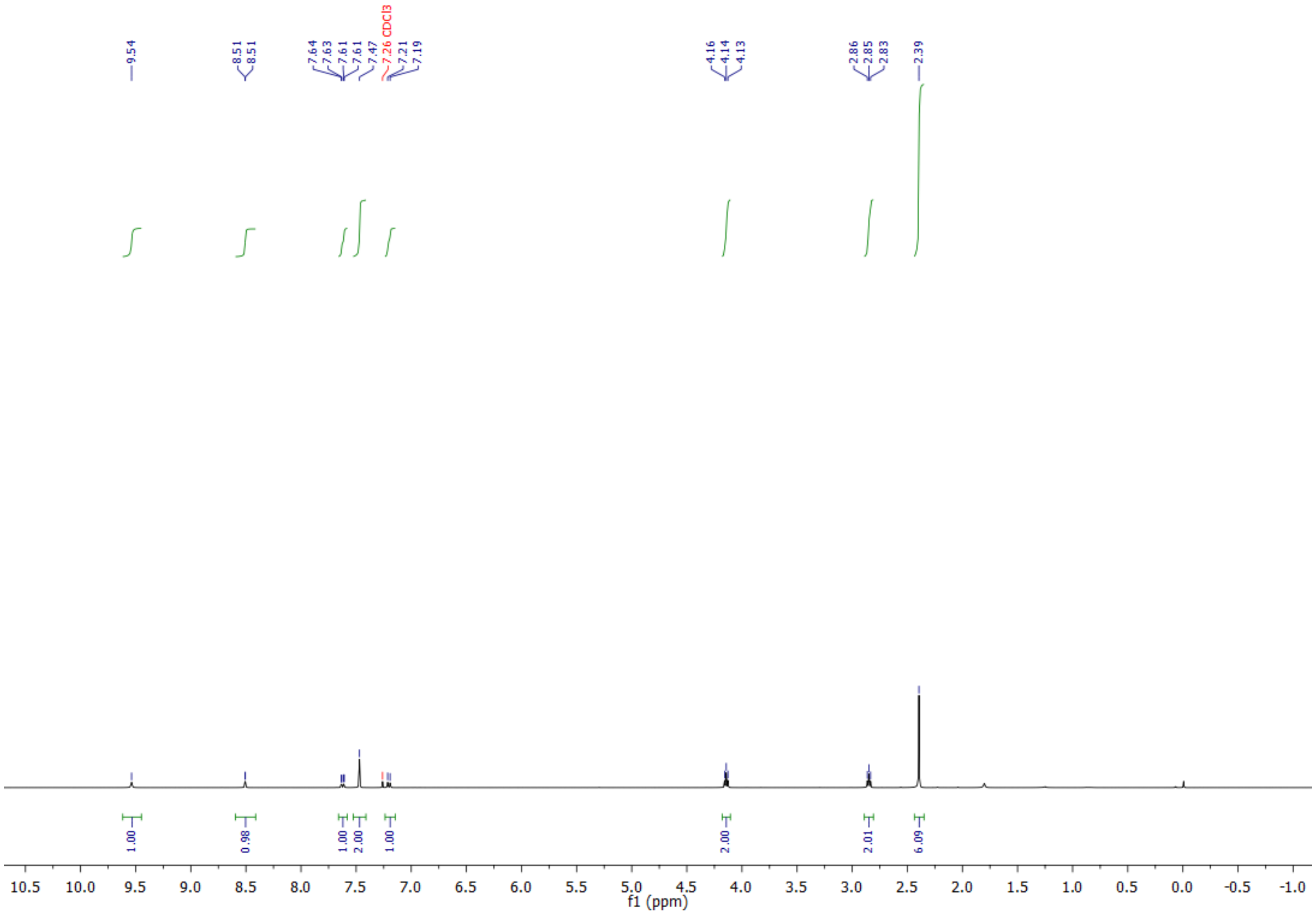

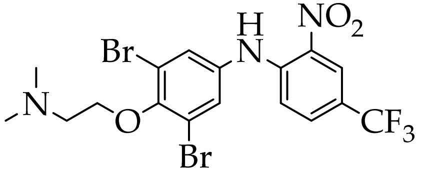

^{13}C NMR (101 MHz, $CDCl_3$) δ 152.41, 144.49, 135.27, 132.54, 132.27 (q, *J* = 3.1 Hz), 129.30, 124.93 (q, *J* = 4.1 Hz), 123.34 (d, *J* = 271.2 Hz), 120.59 (q, *J* = 34.6 Hz), 119.31, 116.61, 71.35, 58.87, 46.00; HRMS (ESI$^+$) for $C_{17}H_{16}Br_2F_3N_3O_3$ ([M+H]$^+$) calculated 525.9583 found 525.9580

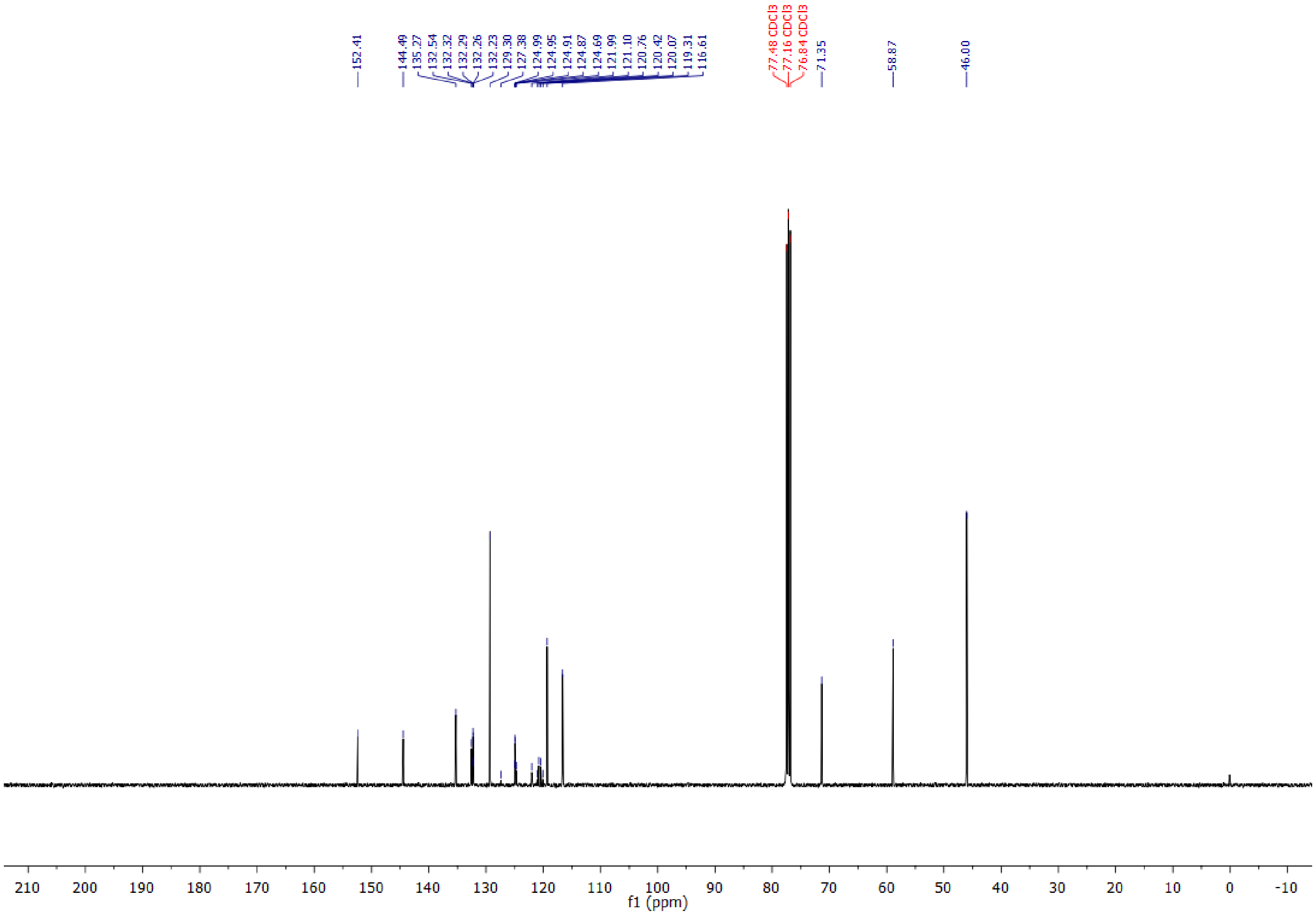

3,5-Dichloro-4-(2-(dimethylamino)ethoxy)-N-(2-nitro-4-(trifluoromethyl)phenyl)aniline (18c)

^{1}H NMR (400 MHz, $CDCl_3$) δ 9.53 (s, 1H), 8.51 (d, *J* = 1.4 Hz, 1H), 7.62 (dd, J_1 = 9.0 Hz, J_2 = 2.1 Hz, 1H), 7.26 (s, 2H), 7.21 (d, *J* = 9.0 Hz, 1H), 4.15 (t, *J* = 5.8 Hz, 2H), 2.82 (t, *J* = 5.8 Hz, 2H), 2.38 (s, 6H)

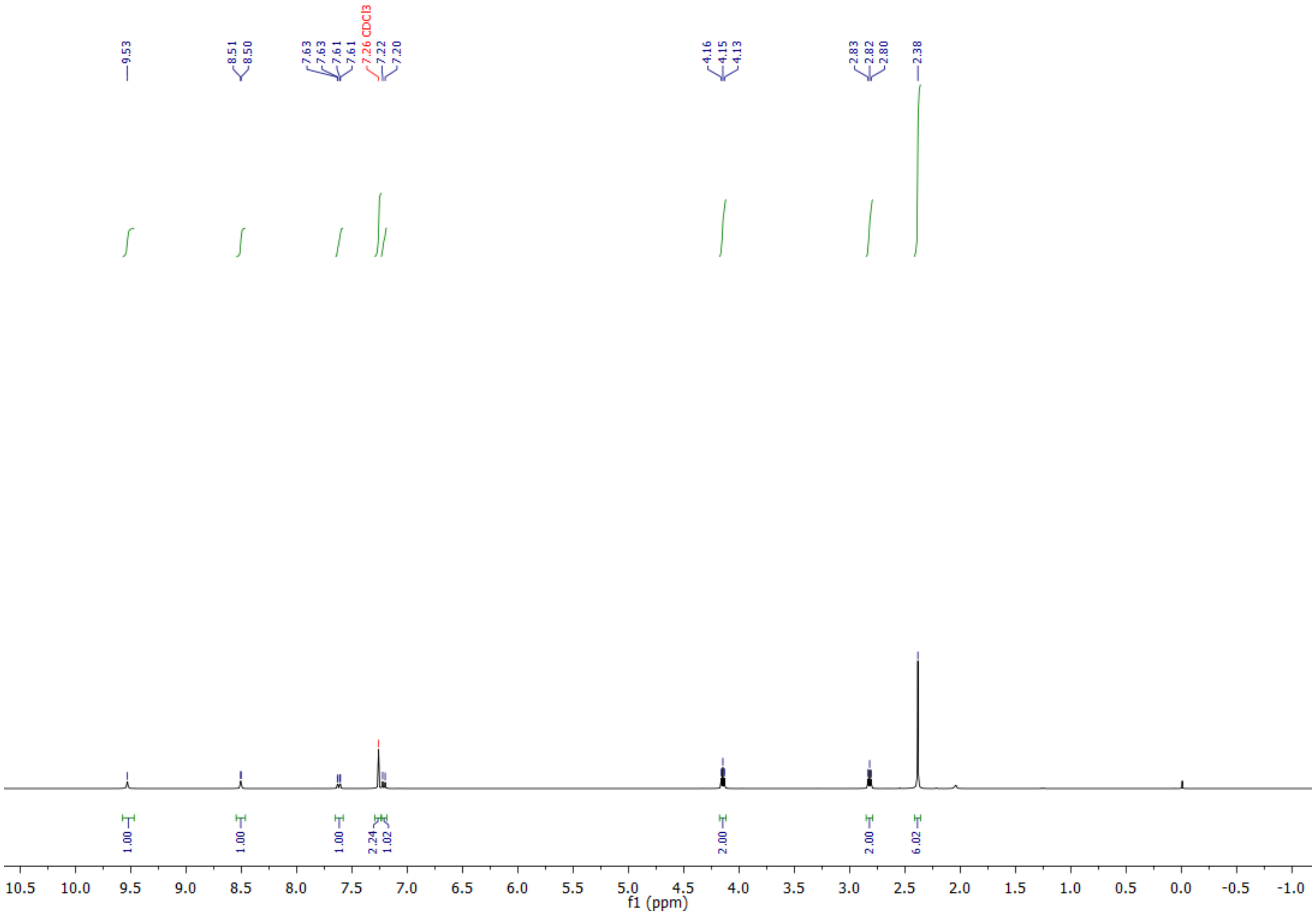

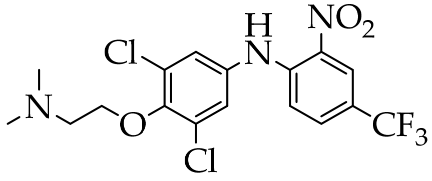

^{13}C NMR (101 MHz, $CDCl_3$) δ 150.46, 144.45, 134.38, 132.55, 132.24 (q, *J* = 3.1 Hz), 130.76, 125.57, 124.92 (q, *J* = 4.2 Hz), 123.33 (q, *J* = 271.3 Hz), 120.56 (q, *J* = 34.6 Hz), 116.63, 71.51, 58.89, 45.93; HRMS (ESI$^+$) for $C_{17}H_{16}Cl_2F_3N_3O_3$ ([M+H]$^+$) calculated 438.0594 found 438.0591

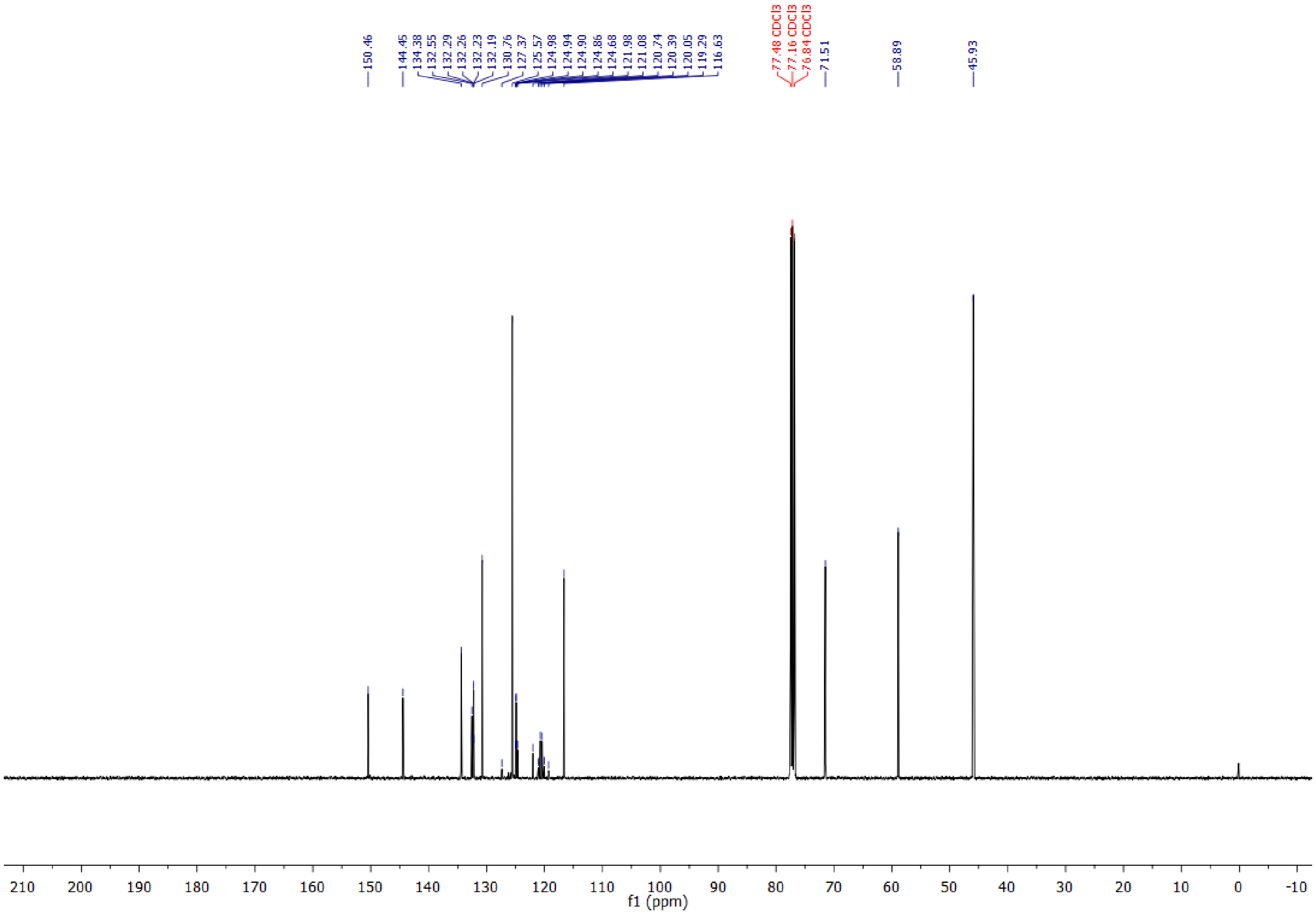

2-Nitro-*N*-(4-(2-(pyrrolidin-1-yl)ethoxy)phenyl)-4-(trifluoromethyl)aniline (19)

^{1}H NMR (400 MHz, $CDCl_3$) δ 9.60 (s, 1H), 8.49 (d, *J* = 1.6 Hz, 1H), 7.48 (dd, J_1 = 9.1 Hz, J_2 = 2.1 Hz, 1H), 7.21 – 7.13 (m, 2H), 7.04 (d, J = 9.1 Hz, 1H), 7.02 – 6.93 (m, 2H), 4.15 (t, *J* = 5.9 Hz, 2H), 2.95 (t, *J* = 5.9 Hz, 2H), 2.67 (t, *J* = 6.6 Hz, 4H), 1.84 (h, *J* = 3.2 Hz, 4H)

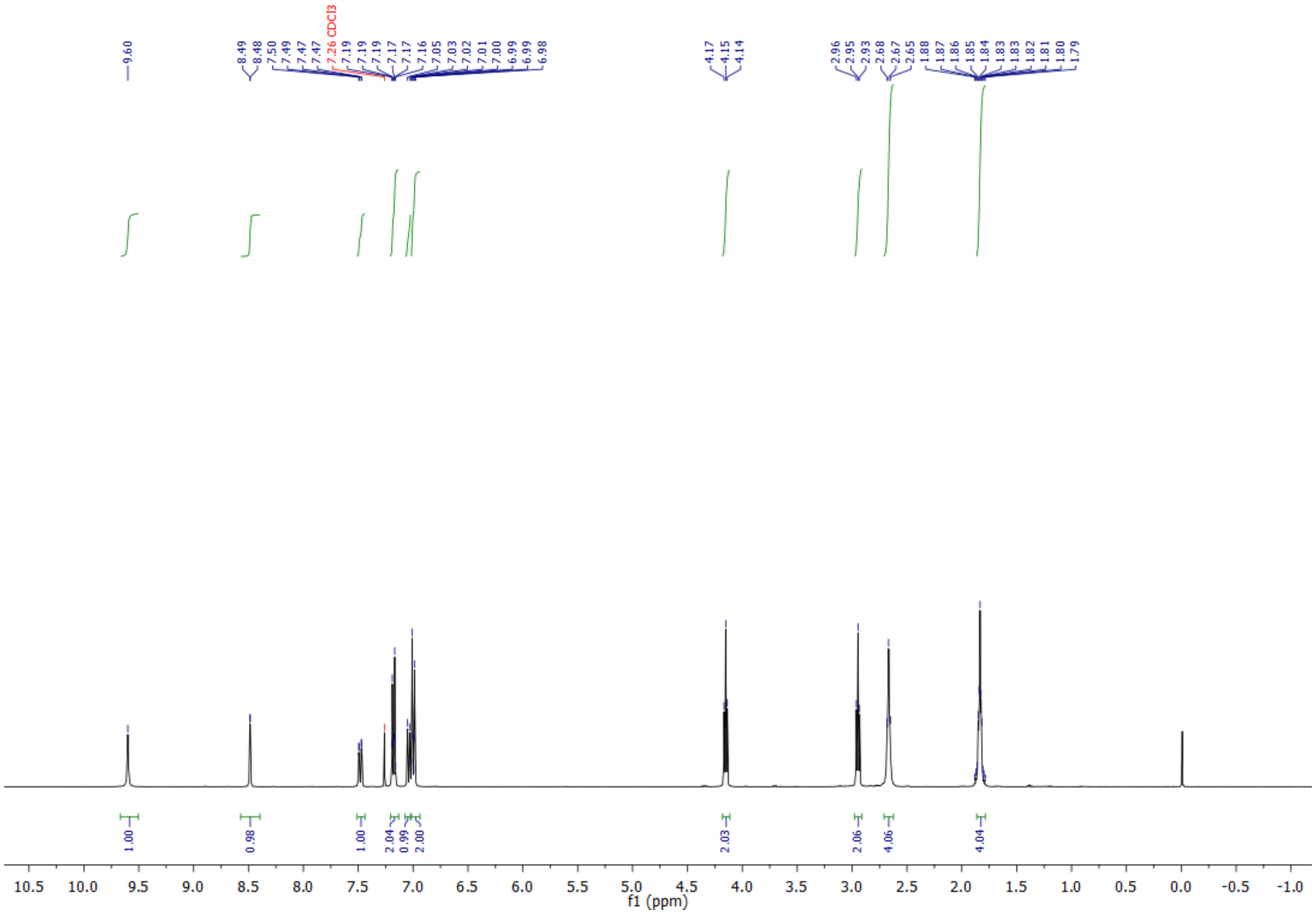

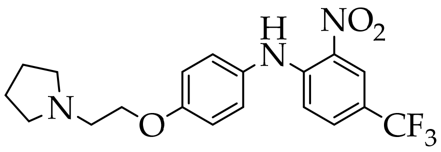

^{13}C NMR (101 MHz, $CDCl_3$) δ 158.00, 146.47, 131.81 (q, *J* = 3.1 Hz), 131.40, 130.14, 127.52, 124.93 (d, *J* = 271.6 Hz), 124.83 (q, *J* = 4.3 Hz), 118.86 (q, *J* = 34.4 Hz), 116.64, 116.00, 67.52, 55.14, 54.90, 23.63; HRMS (ESI+) for $C_{19}H_{20}F_3N_3O_3$ ($[M+H]^+$) calculated 396.15295 found 396.15260

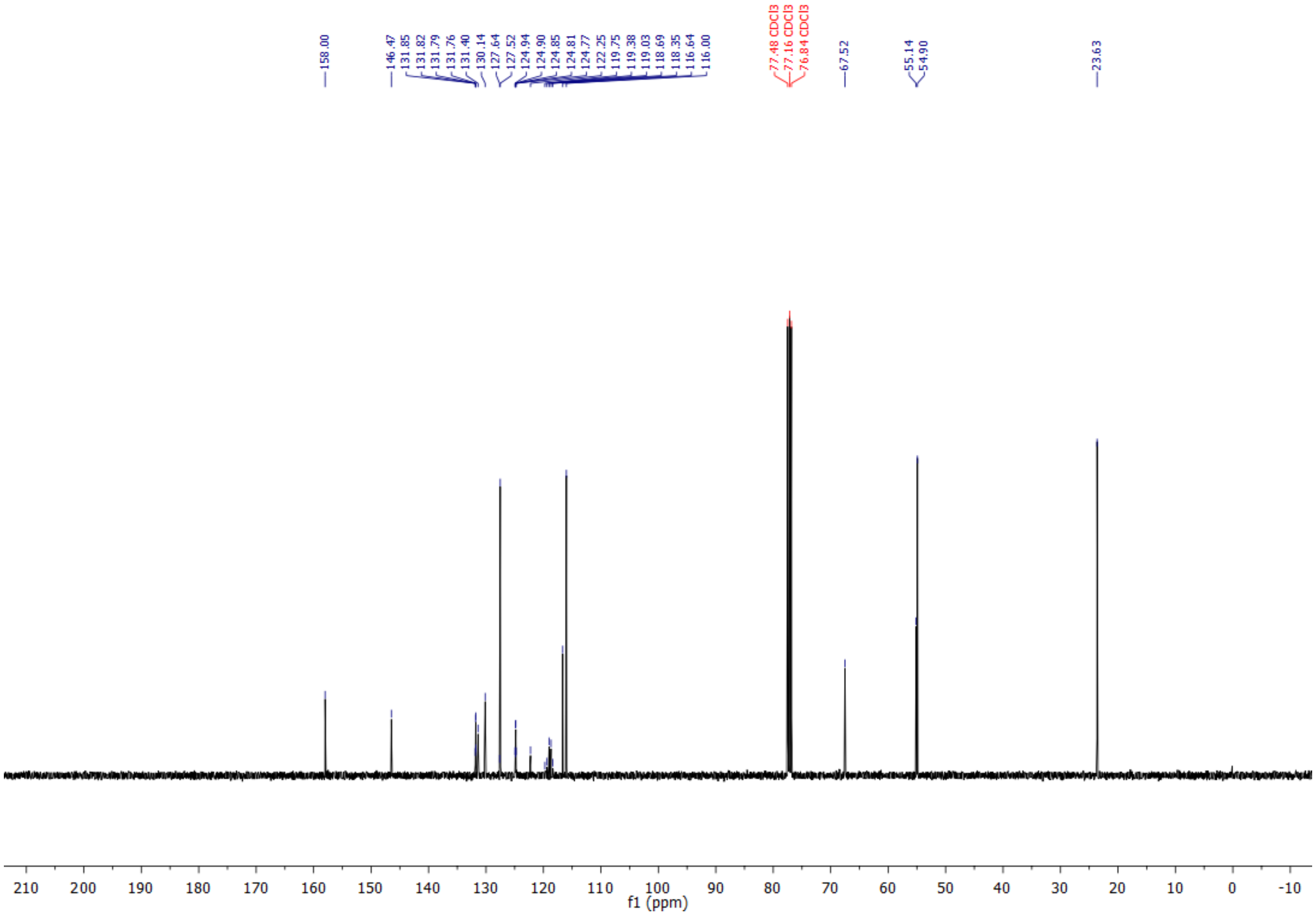

2-Nitro-*N*-(4-(2-(piperidin-1-yl)ethoxy)phenyl)-4-(trifluoromethyl)aniline (20)

^{1}H NMR (400 MHz, $CDCl_3$) δ 9.60 (s, 1H), 8.49 (d, J = *1*.3 Hz, 1H), 7.48 (dd, J_1 = 9.1 Hz, J_2 =2.2 Hz, 1H), 7.22 – 7.13 (m, 2H), 7.04 (d, *J* = 9.1 Hz, 1H), 7.01 – 6.94 (m, 2H), 4.13 (t, *J* = 6.0 Hz, 2H), 2.79 (t, *J* = 6.0 Hz, 2H), 2.52 (s, 4H), 1.62 (p, *J* = 5.6 Hz, 4H), 1.46 (q, *J* = 5.9 Hz, 2H)

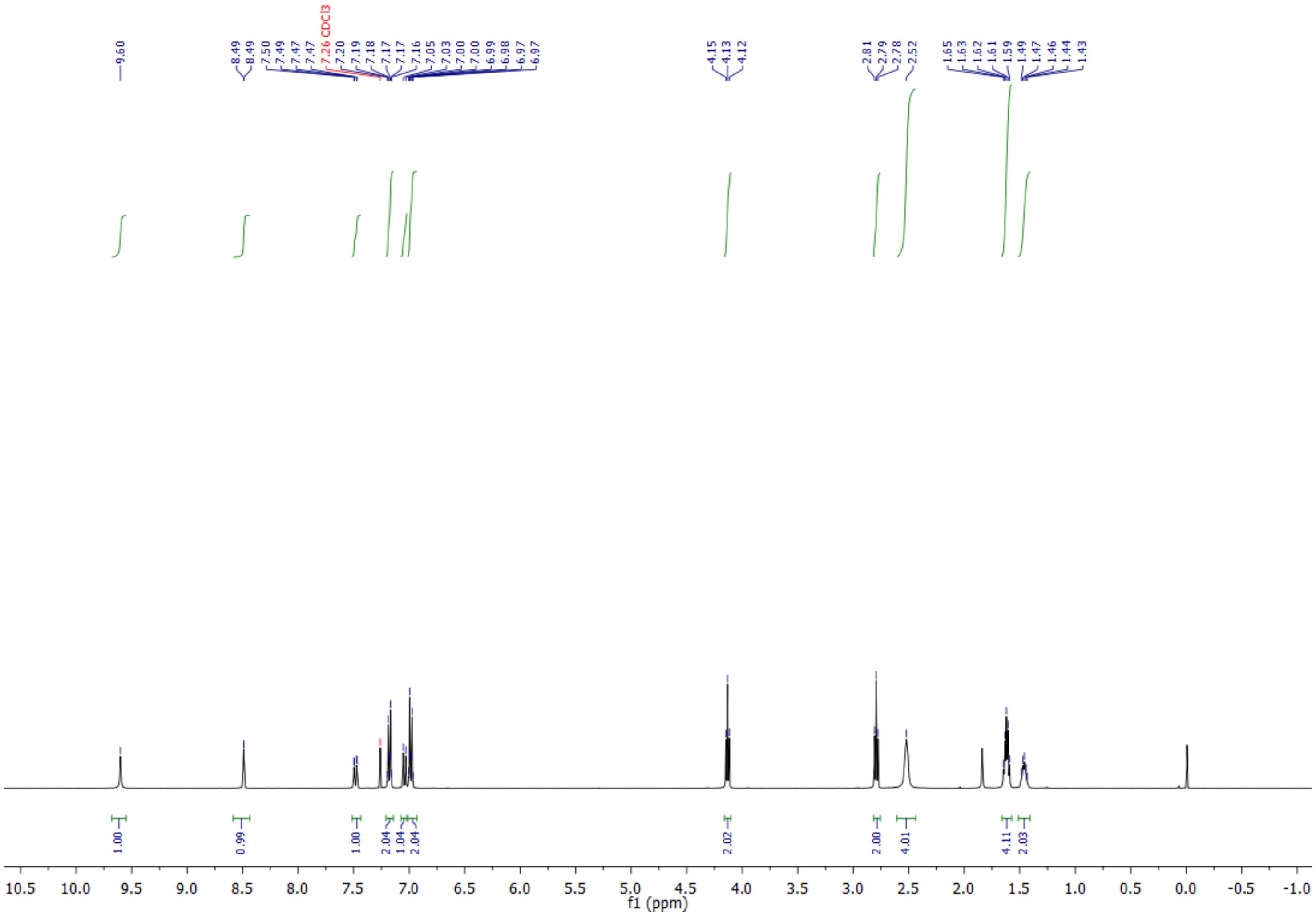

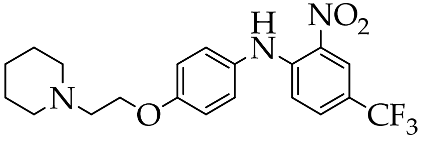

^{13}C NMR (101 MHz, $CDCl_3$) δ 158.04, 146.49, 131.81 (q, *J* = 3.1 Hz), 131.41, 130.11, 127.52, 124.85 (q, *J* = 4.3 Hz), 123.61 (q, *J* = 271.0 Hz), 118.88 (q, *J* = 34.4 Hz), 116.65, 116.02, 66.53, 58.03, 55.26, 26.09, 24.32; HRMS (ESI$^+$) for $C_{20}H_{22}F_3N_3O_3$ ([M+H]$^+$) calculated 410.1686 found 410.1682

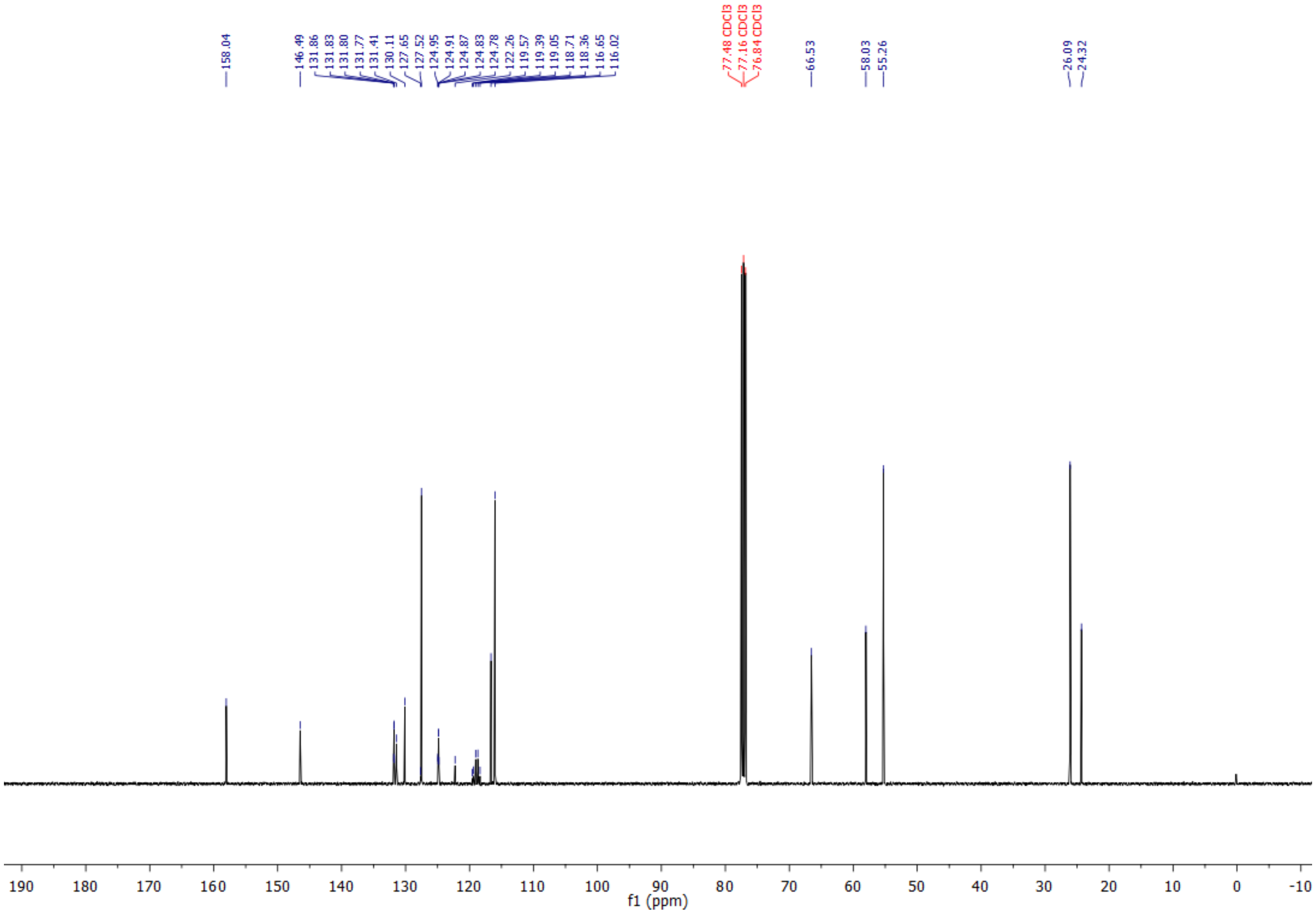

N-(4-(2-(Azepan-1-yl)ethoxy)phenyl)-2-nitro-4-(trifluoromethyl)aniline (21)

^{1}H NMR (400 MHz, $CDCl_3$) δ 9.60 (s, 1H), 8.49 (d, J = 1.4 Hz, 1H), 7.48 (dd, J_1 = 9.1 Hz, J_2 = 2.1 Hz, 1H), 7.21 – 7.15 (m, 2H), 7.04 (d, J = 9.1 Hz, 1H), 7.01 – 6.96 (m, 2H), 4.09 (t, J = 6.2 Hz, 2H), 2.98 (t, J = 6.2 Hz, 2H), 2.84 – 2.74 (m, 4H), 1.75 – 1.57 (m, 8H)

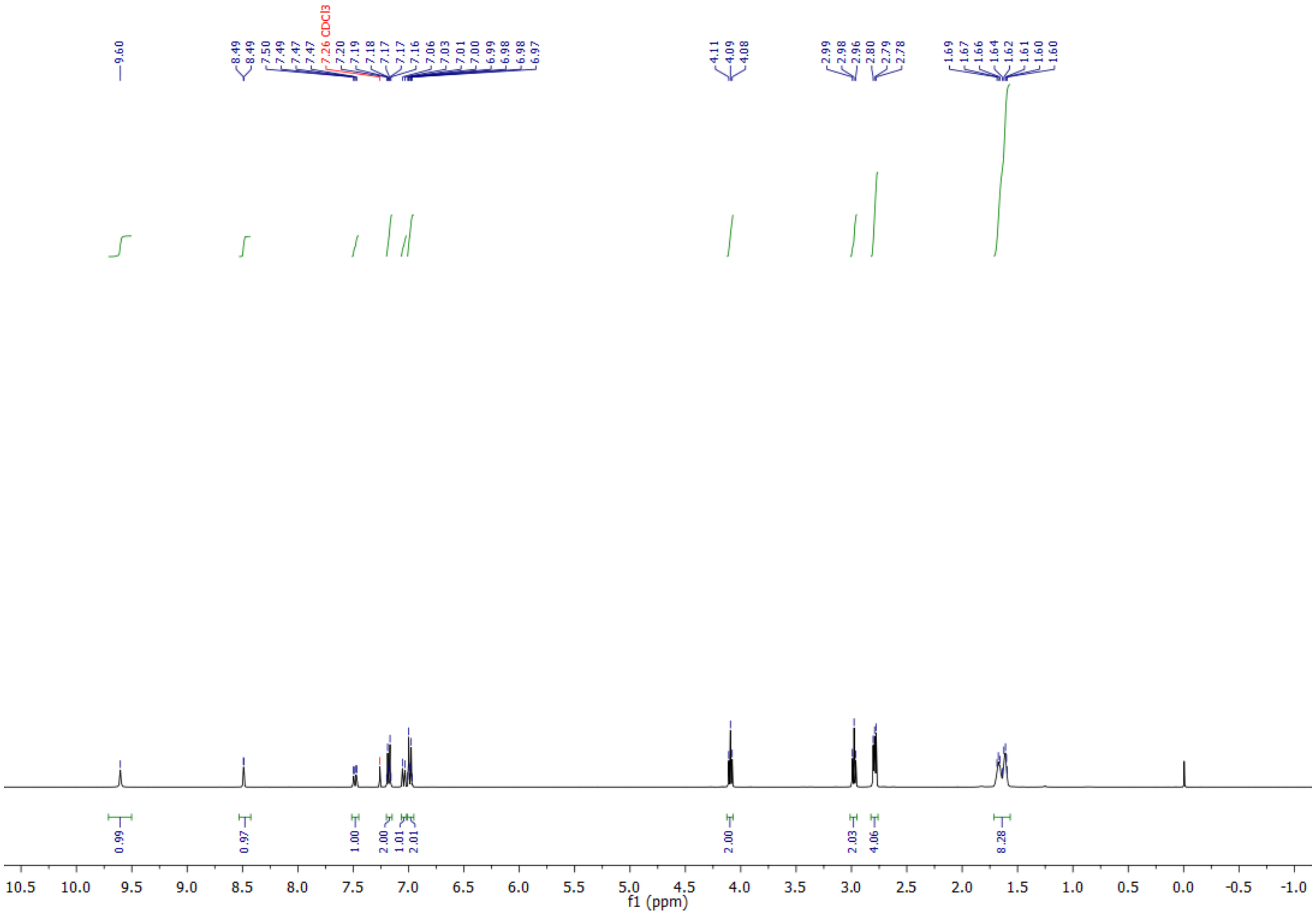

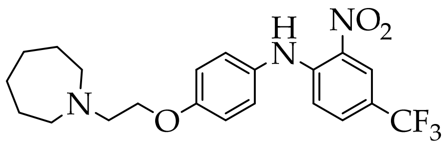

^{13}C NMR (101 MHz, $CDCl_3$) δ 158.14, 146.51, 131.81 (q, *J* = 3.0 Hz), 131.41, 130.05, 127.52, 124.85 (q, *J* = 4.3 Hz), 123.61 (q, *J* = 271.0 Hz), 118.87 (q, *J* = 34.4 Hz), 116.65, 116.02, 67.09, 56.41, 56.05, 28.10, 27.21; HRMS (ESI$^+$) for $C_{21}H_{24}F_3N_3O_3$ ([M+H]$^+$) calculated 424.1843 found 424.1837

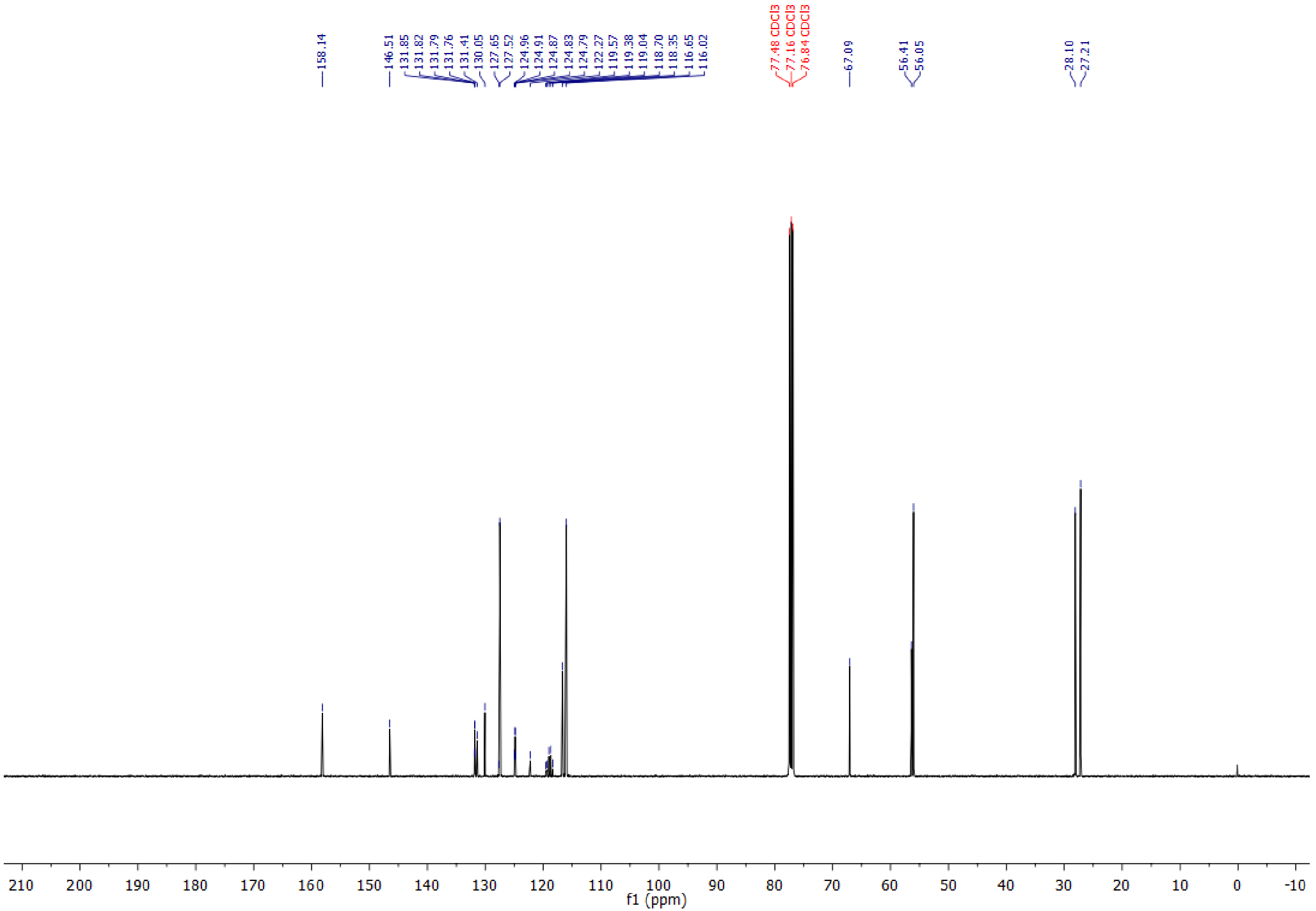

Supplement: Supplementary file 1 [file pharmaceutics-14-01963-s001.zip › pharmaceutics-1904691-supplementary.pdf]
